# Genome-wide analysis of genes encoding core components of the ubiquitin system in soybean (*Glycine max*) reveals a potential role for ubiquitination in host immunity against soybean cyst nematode

**DOI:** 10.1186/s12870-018-1365-7

**Published:** 2018-07-18

**Authors:** Chunyu Zhang, Li Song, Mani Kant Choudhary, Bangjun Zhou, Guangchao Sun, Kyle Broderick, Loren Giesler, Lirong Zeng

**Affiliations:** 1 Department of Plant Pathology, University of Nebraska, Lincoln, NE 68583 USA; 2 Center for Plant Science Innovation, University of Nebraska, Lincoln, NE 68588 USA; 3 Department of Information Science, University of Arkansas, Little Rock, AR 72204 USA; 4 Department of Agronomy and Horticulture, University of Nebraska, Lincoln, NE 68583 USA

**Keywords:** Soybean, Ubiquitin system (UBS), Ubiquitin-activating enzyme (E1), Ubiquitin-conjugating enzyme (E2), RING domain, U-box domain, F-box domain, Soybean cyst nematode, Immunity

## Abstract

**Background:**

Ubiquitination is a major post-translational protein modification that regulates essentially all cellular and physiological pathways in eukaryotes. The ubiquitination process typically involves three distinct classes of enzymes, ubiquitin-activating enzyme (E1), ubiquitin-conjugating enzyme (E2) and ubiquitin ligase (E3). To date, a comprehensive identification and analysis of core components comprising of the whole soybean (*Glycine max*) ubiquitin system (UBS) has not been reported.

**Results:**

We performed a systematic, genome-wide analysis of genes that encode core members of the soybean UBS in this study. A total of 1431 genes were identified with high confidence to encode putative soybean UBS components, including 4 genes encoding E1s, 71 genes that encode the E2s, and 1356 genes encoding the E3-related components. Among the E3-encoding genes, 760 encode RING-type E3s, 124 encode U-box domain-containing E3s, and 472 encode F-box proteins. To find out whether the identified soybean UBS genes encode active enzymes, a set of genes were randomly selected and the enzymatic activities of their recombinant proteins were tested. Thioester assays indicated proteins encoded by the soybean E1 gene *GmUBA1* and the majority of selected E2 genes are active E1 or E2 enzymes, respectively. Meanwhile, most of the purified RING and U-box domain-containing proteins displayed E3 activity in the in vitro ubiquitination assay. In addition, 1034 of the identified soybean UBS genes were found to express in at least one of 14 soybean tissues examined and the transcript level of 338 soybean USB genes were significantly changed after abiotic or biotic (*Fusarium oxysporum* and *Rhizobium* strains) stress treatment. Finally, the expression level of a large number of the identified soybean UBS-related genes was found significantly altered after soybean cyst nematode (SCN) treatment, suggesting the soybean UBS potentially plays an important role in soybean immunity against SCN.

**Conclusions:**

Our findings indicate the presence of a large and diverse number of core UBS proteins in the soybean genome, which suggests that target-specific modification by ubiquitin is a complex and important part of cellular and physiological regulation in soybean. We also revealed certain members of the soybean UBS may be involved in immunity against soybean cyst nematode (SCN). This study sets up an essential foundation for further functional characterization of the soybean UBS in various physiological processes, such as host immunity against SCN.

**Electronic supplementary material:**

The online version of this article (10.1186/s12870-018-1365-7) contains supplementary material, which is available to authorized users.

## Background

Ubiquitination is a major post-translational protein modification that plays an important role in many cellular and physiological processes in eukaryotes [1]. It involves covalently attaching ubiquitin, a highly conserved small protein, to substrate through sequential reactions that are catalyzed by three classes of enzymes: ubiquitin-activating enzyme (E1), ubiquitin-conjugating enzyme (E2), and ubiquitin ligase (E3) [2]. In the enzymatic cascade, the E1 enzyme first activates free ubiquitin in presence of ATP hydrolysis, leading to the formation of a thioester-linkage in which the C-terminal glycine of the ubiquitin molecule is linked with the cysteine residue at the active center of E1. The activated ubiquitin is then transferred to a conserved cysteine residue of the E2 enzyme. In the final step, the ubiquitin molecule is transferred from the E2-ubiquitin intermediate to the substrate protein with the assistance of an E3 ligase. The ubiquitin molecule is usually attached to the ε-amino group of lysine residues of a substrate [3]. The enzymatic cascade can be repeated after the first ubiquitin is attached to the substrate protein, resulting in a polymeric ubiquitin chain being linked to the substrate protein where the linkage between ubiquitin moieties determines the substrate’s fate in the cell [3].

As the enzyme catalyzing the first step of the ubiquitin conjugation cascade, E1s regulate the rate of ubiquitination thus govern the overall ubiquitin function inside the cell [4]. So far, E1 genes and their proteins have been isolated and characterized from rabbit [5], yeast [6], wheat [7], mice [8], human [9], *Arabidopsis thaliana* [4] and tobacco [10]. Multiple E1 genes have been identified in each of the plant and animal genomes analyzed, whereas the yeast genome contains only a single E1 gene. The E1 proteins from all kingdoms possess a similar size ranging from 110 to 125 kDa and share regions of high homology that generally contain four different characteristic structural units: the adenylation domain composed of two ThiF-homology motifs [11]; the catalytic cysteine domain composed of the FCCH and SCCH half-domain (for “first” and “second” catalytic cysteine half-domain, respectively) [12]; a four-helix bundle (4HB) that immediately follows the FCCH; and the C-terminal ubiquitin-fold domain (UFD) [11, 13]. The specificity of an E1 towards E2s depends in part on the UFD, which is responsible for recruiting cognate E2s [14].

The E2 enzymes were originally defined as proteins capable of accepting ubiquitin from an E1 through thioester linkage with a cysteinyl sulfhydryl group [15]. All E2s possess a highly conserved domain of about 140–150 amino acids called the ubiquitin-conjugating (UBC) domain where the cysteinyl residue of the active site resides [16]. Currently, 11, 50 and 40 ubiquitin E2 proteins are known to exist in the yeast (*Saccharomyces cerevisiae*), human (*Homo sapiens*) and tomato (*Solanum lycopersicum*), respectively [17–19]. In addition to 37 ubiquitin E2 proteins [17, 20], a UBC domain is also identified in two RUB-conjugating enzymes (RCE1, At4g36800 and RCE2, At2g18600) and a SUMO-conjugating enzyme (SCE1, At3g57870) in *Arabidopsis thaliana* [21]. Additionally, there are eight other Arabidopsis UBC proteins that lack the active site cysteinyl residue required for thioester formation [2]. Previously, the E2s were often considered as ‘ubiquitin carriers’ with auxiliary roles. However, recent studies have suggested that E2s control the switch from chain initiation to elongation and govern the topology of ubiquitin chains formed, thereby determine the fate of the substrate proteins being modified [22].

The E3 ubiquitin ligases are the largest and most diverse group among the three classes of enzyme that catalyze the ubiquitination cascade. They recruit the target proteins for ubiquitination and are the main factor that determines the specificity of ubiquitination [23]. In the Arabidopsis and human genome, more than 1300 and 600 genes are predicted to encode E3-related components, respectively [24, 25]. The E3 ligases can be either single-polypeptide proteins or multi-subunits complexes. Based on the mechanism of action and the presence of different protein domains responsible for E3 ligase activity, the single-polypeptide ubiquitin ligases can be divided into three defined types, the homology to the E6-associated protein C-terminus (HECT)-, really interesting new gene (RING)-, or U-box-domain containing E3s. The HECT-type E3s are single-subunit proteins characterized by having a C-terminal, approximately 350-amino-acid HECT domain that is involved in both accepting ubiquitin from an E2 protein and transferring it to the substrate protein [26]. A unique feature of the HECT-type E3 ligases is a conserved and catalytic cysteine residue that serves as the site for forming a thioester-linked ubiquitin-E3 intermediate. In these E3 ligases, E2 charges the cysteine residue with ubiquitin prior to it being transferred to the substrate. To date, plant HECT-type E3s have been analyzed in *Arabidopsis thaliana* only, which contains seven HECT genes named *UPL1* - *UPL7* [27]. Evolution analysis indicated the number of HECT genes has been kept quite constant in different plant genomes [28]. Unlike the HECT-type E3s, RING and U-box proteins noncovalently interact with E2 carrying thioester-linked ubiquitin via the conserved RING or U-box domain to facilitate the transfer of ubiquitin to the substrate [2]. RING and U-box ligases are structurally related and use zinc-chelating domain and hydrogen bonds /salt bridges, respectively to transfer ubiquitin [20, 29]. The RING-type E3s are the most abundant among single-subunit ubiquitin ligases [30, 31]. The U-box domain is a modified RING domain that lacks conserved Zn-coordinating residues [32]. The U-box-type E3 ubiquitin ligases are characterized by the conserved ~ 70 amino acid U-box domain originally identified in the yeast UFD2 protein [33]. In addition to being typically single-polypeptide E3s, the RING domain-containing proteins can also be a subunit of complex, multi-subunits E3s, including the Skp1-Cullin-F-box (SCF), the anaphase-promoting complex/cyclosome (APC/C) and the Cullin-Elongin-BC-VHL (CBC VHL)-type E3 ligases [34]. In the well-studied SCF-type multi-subunits ligase, the RING domain-containing protein RBX/ROC/HRT is responsible for binding to E2, whereas the F-box protein confers the substrate recognition [35]. A F-box protein contains at least one F-box domain that spans about 40 amino acids at their N-terminus, which binds the SKP1 to create the SCF complex [36]. F-box proteins have been identified in both prokaryotes [37] and eukaryotes. In plants, the F-box gene family is also one of the largest gene families, suggesting they may regulate many important biological processes [38, 39].

Ubiquitination was originally identified as a principal signal for selective protein degradation in the cell. However, the functions of ubiquitination have extended far beyond that since its discovery over three decades ago. The importance of ubiquitination in the regulation of myriad cellular and physiological processes in animal, human and plant has been increasingly appreciated in the past three decades [31, 40]. Soybean (*Glycine max*) is a major crop and the dominant oil-seed in world. Diseases have been a major constraint on soybean yield [41]. Soybean cyst nematode (SCN, *Heterodera glycines* Ichinohe) has consistently been the most economically important pathogen of soybean worldwide, and causes approximately $1 billion in annual yield loss in the United States [42]. Although a few subfamilies of E3 ligases have been studied individually in soybean [43–46], a comprehensive knowledge on core components of the whole ubiquitin system (UBS) has not been reported yet. In the present study, a genome-wide analysis of core components of the soybean UBS was performed. Through an array of bioinformatics analyses, 4 ubiquitin E1 genes, 71 ubiquitin E2 genes, 1356 genes encoding ubiquitin E3s including 760 RING domain-, 124 U-box domain- and 472 F-box domain-containing E3s were identified with high confidence from the soybean genome [47]. Dozens of components of soybean UBS were cloned, and their enzymatic activities were examined. Moreover, analyses of RNA-seq data and real time quantitative PCR (real time qPCR) results indicated the expression patterns of many components in the soybean UBS were significantly changed under the soybean cyst nematode (SCN) treatment, which supports the notion that soybean UBS may play a role in host immunity against SCN. These results provide a valuable foundation for further functional characterizations of key components of soybean UBS in various physiological processes including their roles in soybean immunity against SCN.

## Results

### The soybean genome possesses four ubiquitin E1 genes

All eukaryotic E1s contain an adenylation domain composed of two ThiF-homology motifs that are derived from the bacterial ThiF proteins [48]. The ThiF motif is considered to be a minimal module for ubiquitin- and ubiquitin-like protein (UBL)-E1 recognition and adenylation activities [49]. Thus, the consensus sequence of the ThiF motif (PF00899) from NCBI conserved domain database (CDD) was employed as query to perform BLAST search against the Phytozome v.12.1 database of the soybean genome (*Glycine max* Wm82.a2.v1). A total of 37 transcripts from 20 genes encoding ThiF motif-containing proteins were identified, apparently due to some of the genes have multiple annotated transcripts (Additional file 1: Table S1). Among them, seven transcripts from four genes encode proteins with more than 1000 amino acids and a molecular weight (Mw) around 120 kD (Additional file 1: Table S1), similar to the ubiquitin E1 proteins that have been characterized in other plant species [4, 7].

**Fig. 1:**
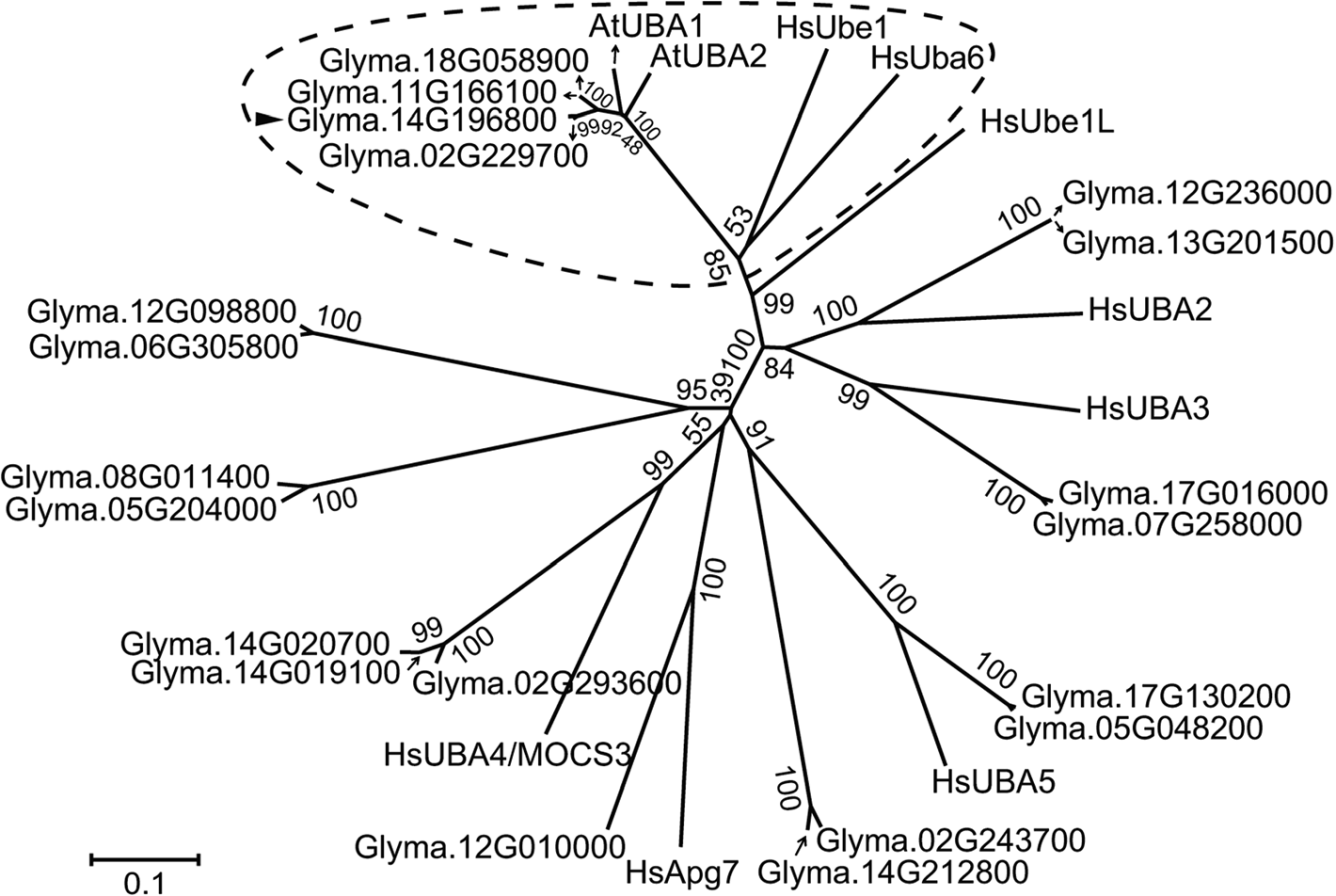
Phylogenetic tree of soybean and human ThiF motif-containing proteins and two Arabidopsis ubiquitin E1 proteins. The unrooted phylogenetic tree was constructed by the neighbor-joining (NJ) method using MEGA 6.0 with 1000 bootstrap replicates. Arrowhead marks the gene that was cloned for verification of enzymatic activity and subsequent assays. Four soybean ThiF motif-containing proteins, Arabidopsis ubiquitin E1s and human UBE1 and UBA6 were clustered in the same clade which was encircled by broken line

In human eight E1s are known to be responsible for initial ubiquitin and UBL recognition and acyl-adenylation, while only two distinct E1s, UBE1 and UBA6, specifically initiate conjugation of ubiquitin rather than other UBLs [14]. To examine the evolutionary relationship between ThiF motif-containing proteins from soybean and human, we generated a neighbor-joining (NJ) phylogenetic tree (Fig. 1). For those genes that have multiple annotated transcripts, only the protein of the primary transcript specified by the database was included in the analyses. Four ThiF motif-containing proteins encoded by the loci *Glyma.02G229700*, *Glyma.11G166100*, *Glyma.14G196800* and *Glyma.18G058900*, respectively were more closely related to human UBE1 and UBA6, and cluster in the same clade with the Arabidopsis ubiquitin E1 AtUBA1 and AtUBA2 in the tree (Fig. 1).

**Fig. 2:**
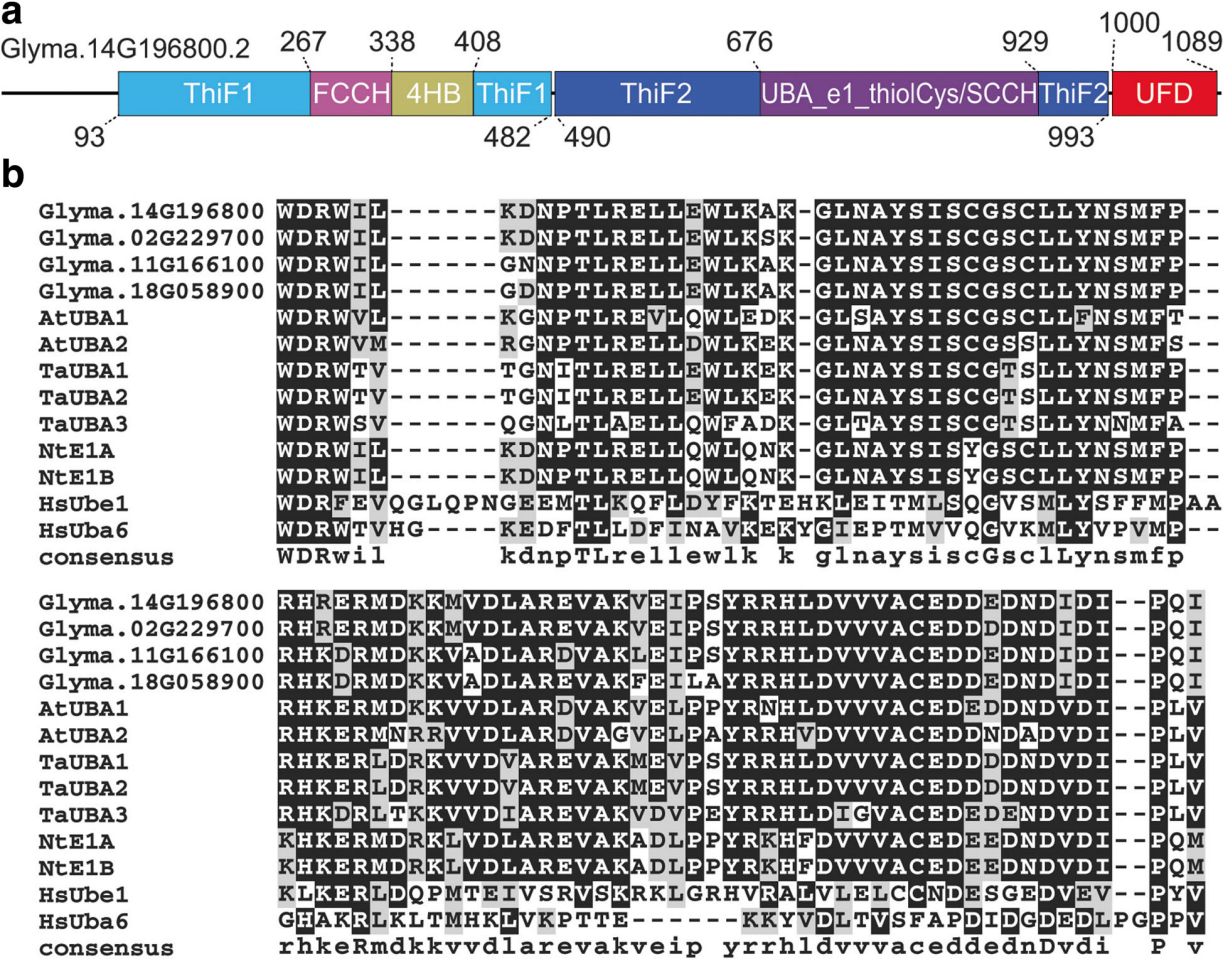
Sequences analysis of ThiF motif -containing proteins in soybean. **a** Structure of soybean ThiF motif -containing proteins as represented by Glyma.14G196800.2. The protein sequences of soyben ThiF motif -containing proteins were analyzed by Pfam (http://pfam.xfam.org/). FCCH: first catalytic cysteine half-domain; 4HB: a four-helix bundle; UFD: ubiquitin-fold domain. The UBA_e1_thiolCys domain that is also called SCCH contains a cysteine residue responsible for ubiquitin thioester linkage. **b** Multiple sequence alignment of the UFD (ubiquitin fold domain) domain of E1 proteins from soybean (*Glycine max,* Glyma), Arabidopsis (*Arabidopsis thaliana*, At), wheat (*Triticum aestivum,* Ta), tobacco (*Nicotiana tabacum*, Nt), and human (*Homo sapiens*, Hs). The UFD was analyzed by Pfam. The multiple sequence alignment was implemented by MUSCLE program using MEGA6, and the image was generated by BoxShade [91]. Conserved and similar residues are shaded in black and grey. The sequence below the alignment indicates the consensus sequence of the aligned UFDs

All the soybean ThiF motif-containing proteins were further subjected to domain analysis using the Pfam database [50]. The four proteins that cluster with the Arabidopsis ubiquitin E1s in the phylogenetic analysis contain in each of them two ThiF motifs (PF00899), an UFD (PF09358) as well as a FCCH (PF16190) and 4HB (PF16191) inserted into the first ThiF motif and an UBA_e1_thiolCys (SCCH) (PF10585) inserted into the second ThiF motif (Fig. 2a), which resemble the typical domain organization of an ubiquitin E1 enzyme. Other soybean ThiF motif-containing proteins present either only one ThiF motif or no UFD domain at the C-terminus. The C-terminal UFD is conserved in E1s from different kingdoms and plays an important role in recruiting specific E2s [13]. Sequence alignments revealed the UFDs from the four soybean ThiF motif-containing proteins encoded by the loci *Glyma.02G229700*, *Glyma.11G166100*, *Glyma.14G196800* and *Glyma.18G058900* are similar to those in other plants and human (Fig. 2b). Taken together, we conclude the soybean genome possesses four genes encoding putative ubiquitin E1 proteins.

### Seventy-one ubiquitin E2s encoded by the soybean genome are classified into eleven groups

To pinpoint soybean genes that encode ubiquitin E2, the hidden Markov model (HMM) profile of ubiquitin-conjugating (UBC) domain (PF00179) (Additional file 2: Table S2) from the Pfam database was used as query to search against the soybean protein database by employing the HMMER 3.1 program [51]. Similar to the E1 genes, we found many putative E2 genes have multiple annotated transcripts (isoforms) and only the primary transcript (i.e. the major transcript) specified by the database for these genes was used for subsequent analyses. A total of 107 genes that encode UBC domain-containing proteins were identified by the HMMER analysis. The Pfam database and NCBI CCD database were further used to validate the 107 proteins. A putative UBC domain was identified in 99 and 106 proteins by BLAST against the two databases, respectively (Table 1). By combining these results, we preliminarily predicted 106 genes encoding putative UBC domain-containing proteins in the soybean genome (Table 1).

**Table 1:** Summary of the number of soybean UBS components identified after HMMER analysis, BLAST search against the Pfam and NCBI databases, and manual validation

Methods	UBC genes	RING genes	U-box genes	F-box genes
HMMER search^a^	107	1234	158	579
Pfam Search	99	826	127	572
NCBI Search	106	1034	145	470
Preliminary Hits^b^	106	1034	145	547
Manual Validation	91	760	124	472

^a^Multiple annotated proteins corresponding to alternative spliced transcripts of the same gene were found in HMMER search. The sequence of the primary transcript for these genes as specified by the database was used

^b^The number of Preliminary Hit denote the number of corresponding UBS component identified after combining the Pfam and NCBI searches

**Fig. 3:**
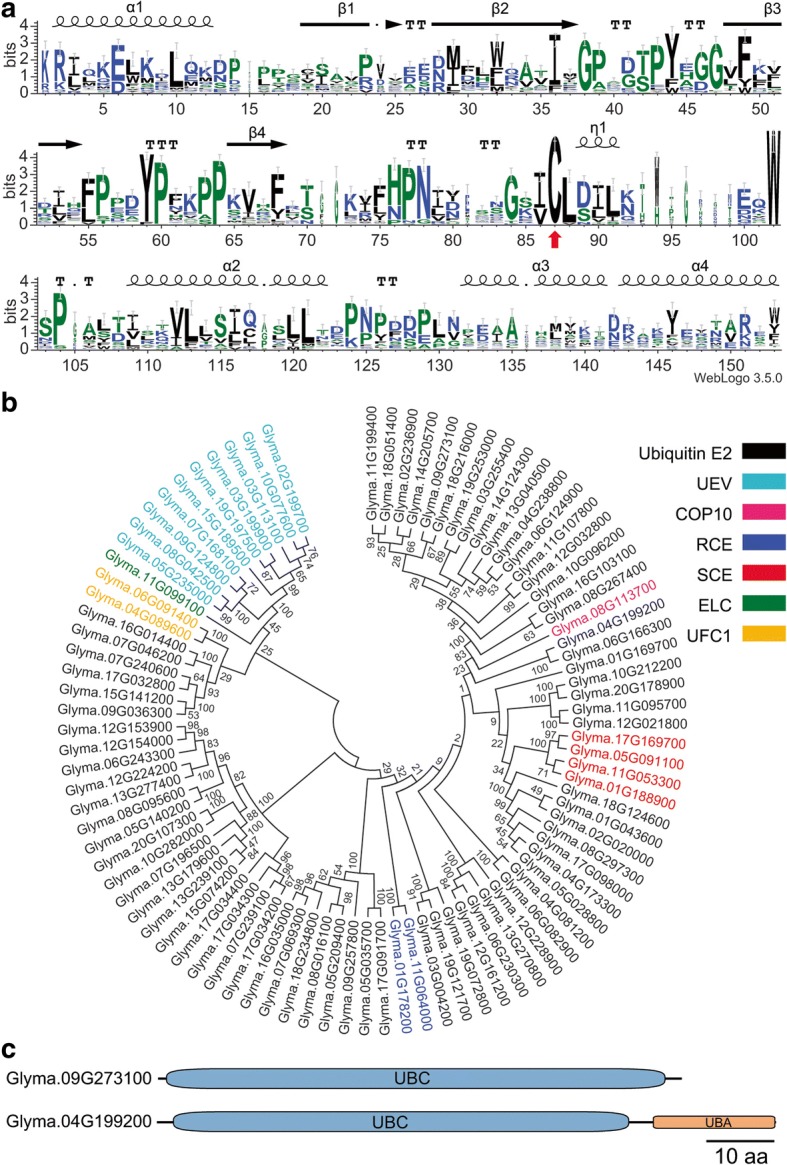
Structure and phylogenetic analysis of UBC domain-containing proteins in soybean. **a** Graphical sequence logo representing sequence of the UBC domains from 91 soybean UBC domain-containing proteins. The overall height of each stack indicates the degree of sequence conservation, while the height of the letters within each stack indicates the relative frequency of corresponding amino acid at the location. The width of the letters is proportional to the fraction of valid letters in that position. Error bars indicate a Bayesian 95% confidence interval. Blue, green and black letters show hydrophilic, neutral and hydrophobic residue, respectively. The bit score of the Y-axis denotes the information content of a given sequence position. Red arrow indicates the active cysteine residue. Positions of secondary structure elements are marked by α for α-helices, β for β-sheets, η for 3_10_-helix, TTT for α-turns and TT for α- and β-turns on top of the logo, respectively. **b** Phylogenetic tree of 91 UBC domain-containing proteins based on the full-length protein sequences. Different types of E2 proteins are indicated with different colors. UEV: ubiquitin-conjugating enzyme variant, RCE: RUB-conjugating enzyme, SCE: SUMO-conjugating enzyme, ELC: ELCH, UFC1: Ubiquitin-fold modifier 1-conjugating enzyme. **c** Two representative domain organizations of the 71 soybean ubiquitin E2 proteins. UBC: ubiquitin-conjugating domain, UBA: ubiquitin-associated domain

To further examine these putative UBC domain protein-coding genes, we performed manual validation. The core of the UBC domain fold forms a four-stranded β-sheet [52]. Beyond this basic core, the UBC domain fold contains two small elements within a C-terminal flap-like structure, and there is also a helix at the N-terminus and 1–2 helices at the C-terminus [53]. Additionally, a catalytic cysteine residue at the active center that is typically located at the C-terminus of the flap is highly conserved in the UBC domain [16, 53]. Sequence analysis eventually determined 91 genes encoding typical UBC domain-containing proteins out of the 106 candidate genes (Additional file 3: Table S3). A graphical sequence logo representing the sequence patterns based on the alignment of the UBC domains from the above 91 proteins was generated using Weblogo3 online (Fig. 3a) [54], and the complete alignment information was shown in Additional file 4: Figure S1.

Besides ubiquitin E2, other proteins such as ubiquitin-conjugating enzyme variant proteins (UEV), Related to Ubiquitin (RUB)-conjugating enzymes (RCE), Small Ubiquitin-like Modifier (SUMO)-conjugating enzyme (SCE), ELCH (ELC homolog) and Ubiquitin-fold modifier 1-conjugating enzyme (UFC1) also contain the UBC domain [19]. To distinguish ubiquitin E2 from those proteins, we generated the phylogeny of soybean and Arabidopsis UBC domain-containing proteins (Additional file 5: Figure S2). The phylogenetic analysis indicated that, of the 91 genes, 71 encode ubiquitin E2 proteins, 11 encode UEV proteins (including homolog of the AtCOP10), two encode RUB E2 proteins (RCE), four encode putative SUMO E2, one encodes ELC and two encode UFC1 E2 proteins (Fig. 3b).

The Arabidopsis ubiquitin E2 proteins were largely subdivided into 12 groups [55]. In addition, the AtUBC37 was assigned to group XIII due to its homology to tomato UBC37 [19]. Based on the phylogenetic analysis of UBC domain-containing proteins in Arabidopsis and soybean, the soybean does not have close homologs to the group V E2s and AtUBC37 in Arabidopsis (Additional file 5: Figure S2)*.* Therefore, the 71 soybean ubiquitin E2 proteins were classified into 11 groups (Additional file 6: Figure S3). Domain organization analysis using the Pfam and the NCBI database indicated that, except for Glyma.04G199200 and Glyma.06G166300, the 71 ubiquitin E2 proteins contain a UBC domain only (Fig. 3c). Both of Glyma.04G199200 and Glyma.06G166300 also contain an additional domain called ubiquitin-associated (UBA) domain at their C-terminuses (Fig. 3c). The UBA domain has been found to mediate protein-protein interactions through binding of ubiquitin molecules [56].

### Identification of genes encoding RING-, U-box- and F-box-domain containing E3s in soybean genome

To identify genes that encode RING-, U-box- and F-box-type E3 ligases in soybean, the HMM profiles of these domains (Additional file 2: Table S2) from Pfam were used as the query files. A total of 1234, 158 and 579 homologs of RING, U-box and F-box proteins, respectively were identified in soybean genome by HMMER analysis (Table 1). To verify these identified proteins, all sequences in FASTA format were uploaded and searched against the Pfam and NCBI databases for detection of the RING, U-box, and F-box domain, respectively. Combined the BLAST results against the Pfam and NCBI databases, 1034, 145, and 547 genes that encode putative RING domain-, U-box domain- and F-box domain-containing proteins were obtained after removing redundant sequences (Table 1).

**Fig. 4:**
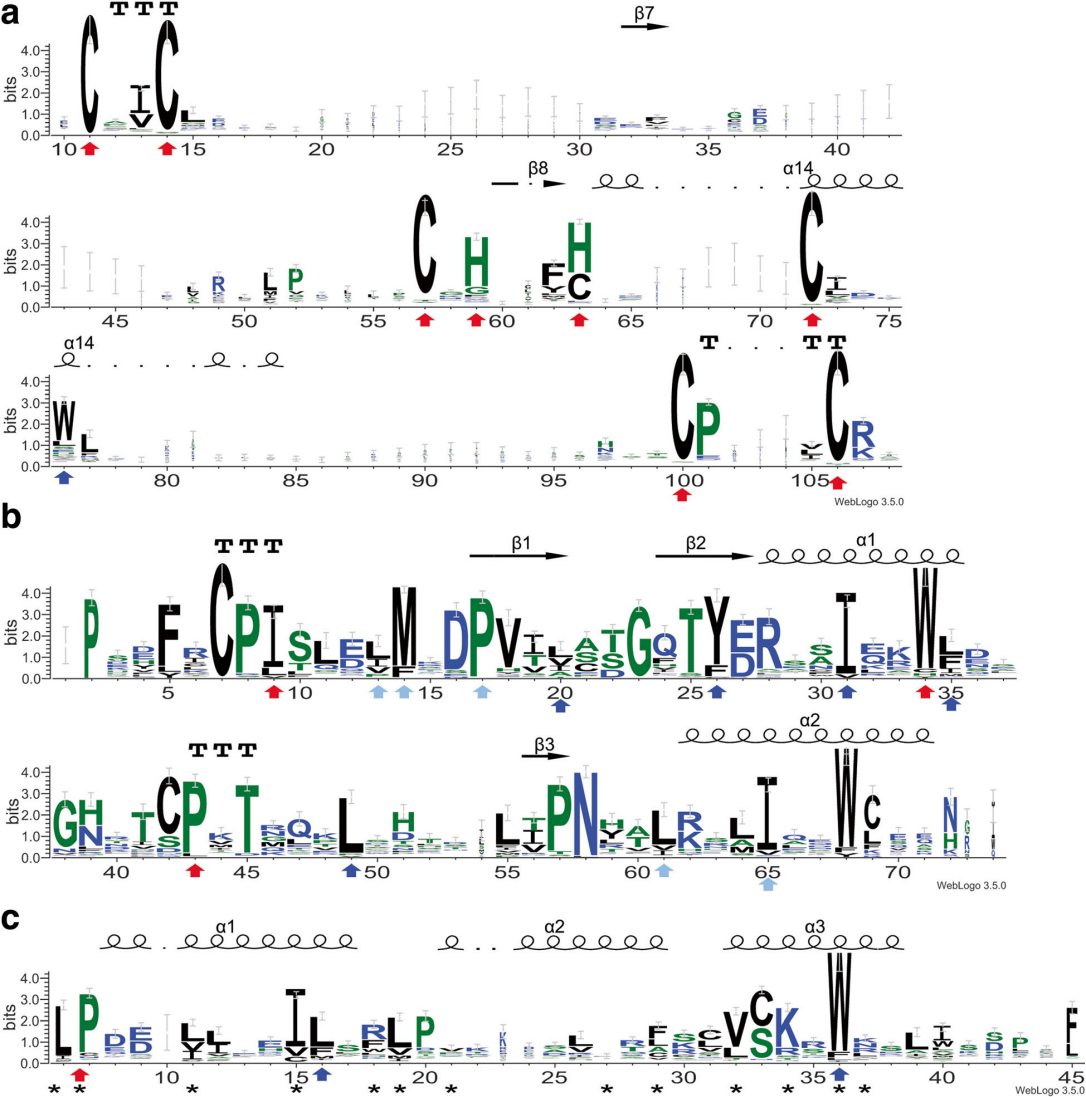
Graphical sequence logo representing the sequence patterns of RING, U-box and F-box domain in identified corresponding type of soybean E3 proteins. Weblogos are generated based on the multiple sequence alignment of RING (**a**), U-box (**b**) and F-box (**c**) domains. Red and blue arrowheads in (**a**) indicate the zinc binding residues of RING domains and the conserved hydrophobic residues that interact with cognate E2s. Red arrowheads in (**b**) indicate the hydrophobic E2-binding residues whereas blue and light blue ones indicate conserved residues in two cores of hydrophobic interactions. Red and blue arrowheads in (**c**) mark the conserved residues that contribute to the formation of α1-helix and the packing of the F-box helices whereas residues marked with star sign are the positions important for human Skp2 contacting with Skp1

Previous structural and biochemical studies have identified key amino acid residues and corresponding secondary structures of RING [30, 57–59], U-box [32, 60], and F-box [61, 62] domain. The information was employed for further validating each of the proteins encoded by the 1034 RING, 145 U-box and 547 F-box genes, respectively. Such manual validation led to the identification of 760 RING, 124 U-box and 472 F-box genes in soybean genome with high confidence (Table 1). The detailed information of these genes is listed in Additional file 3: Table S3. The representative sequences of the respective domains were aligned (Additional file 7: Figure S4, Additional file 8: Figure S5 and Additional file 9: Figure S6) and graphical sequence consensus logos were generated using Weblogo3 online [54] (Fig. 4a, b and c).

A typical RING has the consensus, 40–60 amino acids linear sequence of C-X_2_-C-X_[9–39]_-C-X_[1–3]_-H-X_[2–3]_-C-X_2_-C-X_[4–48]_-C-X_2_-C where the highly conserved Cysteine (C) and Histidine (H) residues form two cross-brace structure to bind two zinc ions and X can be any of the twenty amino acids [57]. Two canonical RING-types (C3H2C3 and C3HC4) that differ in the presence of either a Cys or His at the fifth Cys residue were well characterized [58]. We extracted the sequence of the RING domain from all soybean RING domain-containing proteins that were manually validated. We then performed the alignment of the sequences and generated graphical sequence consensus logos using the Weblogo3 algorithm online (Fig. 4a) [54]. The conserved Cys and His residues that have been known to be responsible for stabilizing two loop regions through coordinating the two zinc ions, as well as a central conserved α-helix that connects the first and second loops are presented in the sequence consensus logos [30, 59] (Fig. 4a). A Trp or other hydrophobic residue that is often found at the α-helix region and has been implicated in interaction with E2s is also presented [30] (Fig. 4a). Unlike the RING domain, the U-box domain lacks the zinc-binding sites. The hydrogen-bonding networks that contain hydrophobic and polar amino acids are proposed to maintain the U-box scaffold [32]. The consensus sequence generated by the Weblogo3 algorithm using sequences of the identified soybean U-box domains displays two α-helices and three β-strands in its secondary structure, which is consistent to the consensus structure of known plant U-box domains, as manifested by the Arabidopsis U-box protein AtPUB14 [60] (Fig. 4b). In the U-box domain, three hydrophobic E2 binding sites and two hydrophobic cores have been shown to be essential for the function of U-box domain [60]. These amino acid residues are identified in the soybean U-box domain consensus sequence generated by Weblogo3 as well (Fig. 4b).

The F-box domain is the signature structure of F-box proteins that act as a subunit of the SCF catalytic core through interacting with Skp1 [62]. Several conserved residues that are known for contributing to protein-protein interaction and structure stability were used for verifying the soybean F-box proteins. In human Skp2 protein, Pro113, a hallmark amino acid residue of F-box domain, assists to launch α-helix while Leu124 and Try139 contribute to the packing of the F-box helices [61]. These amino acid residues are highly conserved in soybean F-box proteins (Fig. 4c). In addition, the Skp1 binding residues of the Skp2 in human were conserved in F-box domain-containing proteins from soybean and other plant species [63–65] (Fig. 4c).

**Fig. 5:**
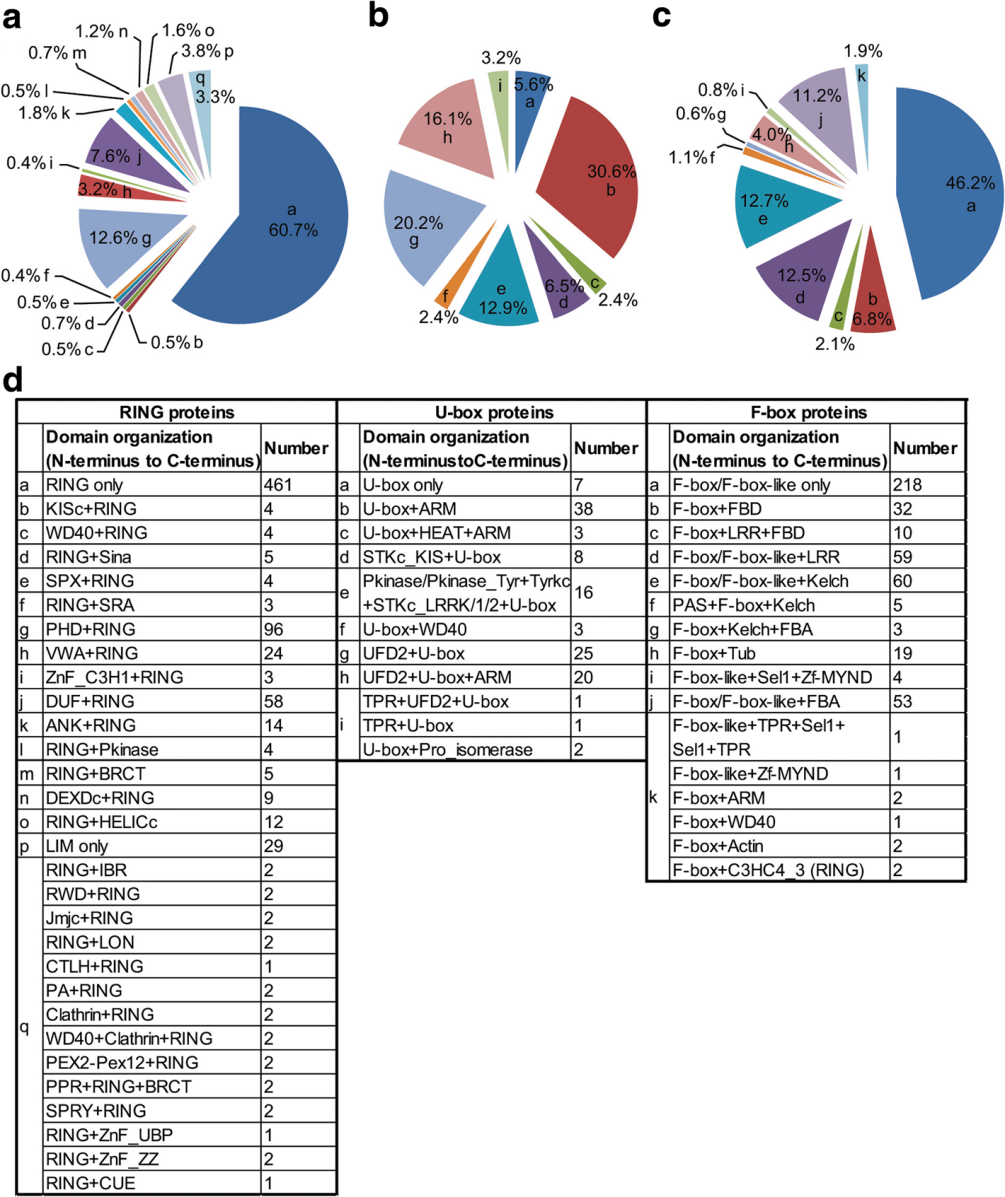
Domain organizations of soybean E3 proteins. Categories and corresponding percentage of soybean RING-, U-box- and F-box- domain-containing proteins with different domain organizations are represented as schematic pie chart in (**a**), (**b**) and (**c**), respectively. The annotations of different categories (in low case alphabetical letters) in (**a**), (**b**) and (**c**) are shown in (**d**)

A protein domain is a conserved structure of a given protein that can evolve, function, and exist independently of the rest of the protein. Analysis of domain organization will thus provide important information to predict the putative function of a protein. To further understand the diversification of RING, U-box and F-box genes in soybean, the corresponding proteins of these genes were also BLAST against the Pfam and the NCBI databases to identify other domains presented in these proteins. The results showed that approximately half of the RING proteins (60.7%) and F-box proteins (46.2%) did not contain other known domains, while the U-box proteins were only 5.6% (Fig. 5). In addition to the RING, U-box and F-box domain, 28, 10 and 13 additional types of protein domain were found in RING, U-box and F-box domain-containing proteins, respectively (Fig. 5d). Our analyses revealed that an additional known domain may appear in different types of E3s whereas a specific E3 protein may contain multiple known domains. For instance, the WD40 repeats domain was found in RING, U-box and F-box proteins (Fig. 5d). To understand the evolutionary relationship/homology of the identified E3 proteins, phylogenetic tree was constructed for the soybean RING, U-box and F-box proteins, respectively using their full-length protein sequences (Additional file 10: Figure S7, Additional file 11: Figure S8 and Additional file 12: Figure S9). Next, gene duplication events in the gene family encoding RING- and F-box-type E3 ligases, respectively were analyzed using MCScanX [66]. The analyses revealed 543 RING genes (71.4% of total RING genes) are in homologous chromosomal regions derived from whole genome duplications (WGD) /segmental duplications whereas only 24 (3.2% of total RING genes) from tandem duplications. For F-box genes, 181 (38.3% of total F-box genes) were found to be WGD/segmental duplications, while 86 (18.2% of total F-box genes) are tandem duplications (Additional file 13: Figure S10 and Additional file 14: Figure S11 and Additional file 15: Table S4). These results suggest WGD/segmental duplications contribute mainly to gene expansion in these soybean gene families.

### The soybean ubiquitin E1 protein and majority of the ubiquitin E2s examined are enzymatically active

**Fig. 6:**
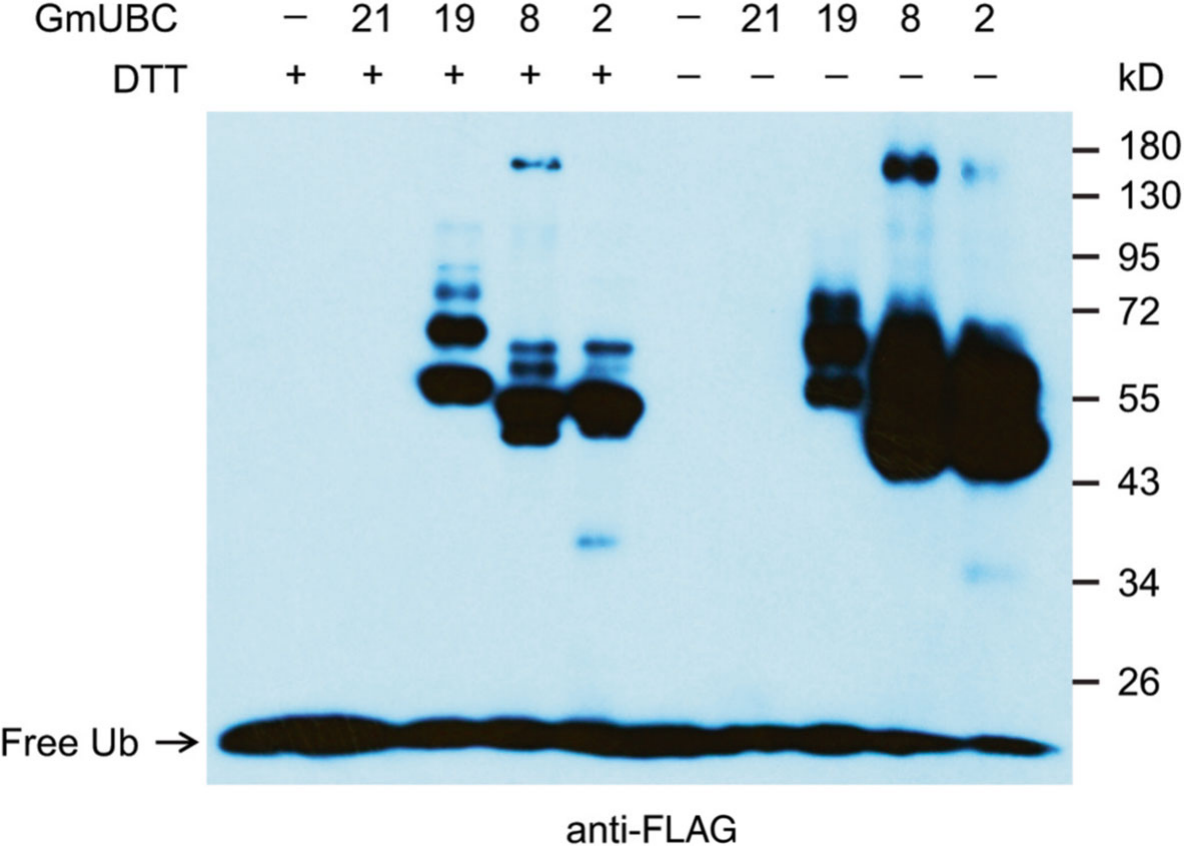
Examination of the enzymatic activity of soybean E2s by thioester assay in the presence of soybean E1, GmUBA1. Immunoblot with anti-FLAG antibody was performed following thioester formation assay. Recombinant soybean E2 (GmUBC) proteins were incubated in the presence of GmUBA1 and ubiquitin. Each reactions was split and treated with 100 mM DTT (+) or 4 M urea (-). The numbers at the right indicate the molecular masses of marker proteins in kilo-Dalton (kD)

To determine whether the identified soybean E1 and E2 genes encode active ubiquitin-activating and ubiquitin-conjugating enzymes, we cloned an E1 gene, *Glyma.14G196800*, and four E2 genes, *Glyma.17G098000*, *Glyma.09G273100*, *Glyma.12G021800* and *Glyma.04G081200* (Fig. 1; Additional file 5: Figure S2) and expressed their recombinant proteins in *Escherichia coli* (*E. coli*). We named the E1 gene as *GmUBA1* because it is the first ubiquitin E1 that is identified and characterized in soybean. The E2 genes were named *GmUBC2* (*Glyma.17G098000*), *GmUBC8* (*Glyma.09G273100*), *GmUBC19* (*Glyma.12G021800*) and *GmUBC21* (*Glyma.04G081200*) based on their homology to Arabidopsis *UBC2*, *UBC8*, *UBC19* and *UBC21* gene, respectively [20] (Additional file 5: Figure S2). We successfully purified recombinant proteins for all the cloned genes (Additional file 16: Figure S12). The purified E1 and E2 proteins were then used in an in vitro thioester assay to detect their enzymatic activities [19]. In the thioester assay, the E1 enzyme activates free ubiquitin molecule to form a thioester-linked ubiquitin in an ATP-dependent manner. Thioester-linked ubiquitin is then transferred to an active E2 enzyme to form E2-ubiquitin adduct that is sensitive to reducing agent dithiothreitol (DTT) [20]. As shown in Fig. 6, except for GmUBC21, GmUBC2, 8 and 19 formed adducts with ubiquitin that were sensitive to 100 mM DTT, indicating that a thioester linkage was formed in the presence of GmUBA1. These results demonstrated that GmUBA1 is active ubiquitin E1 enzyme, and GmUBC2, 8 and 19 possess ubiquitin-conjugating activity.

### Randomly selected soybean RING and U-box genes encode proteins that possess E3 ubiquitin ligase activity

**Fig. 7:**
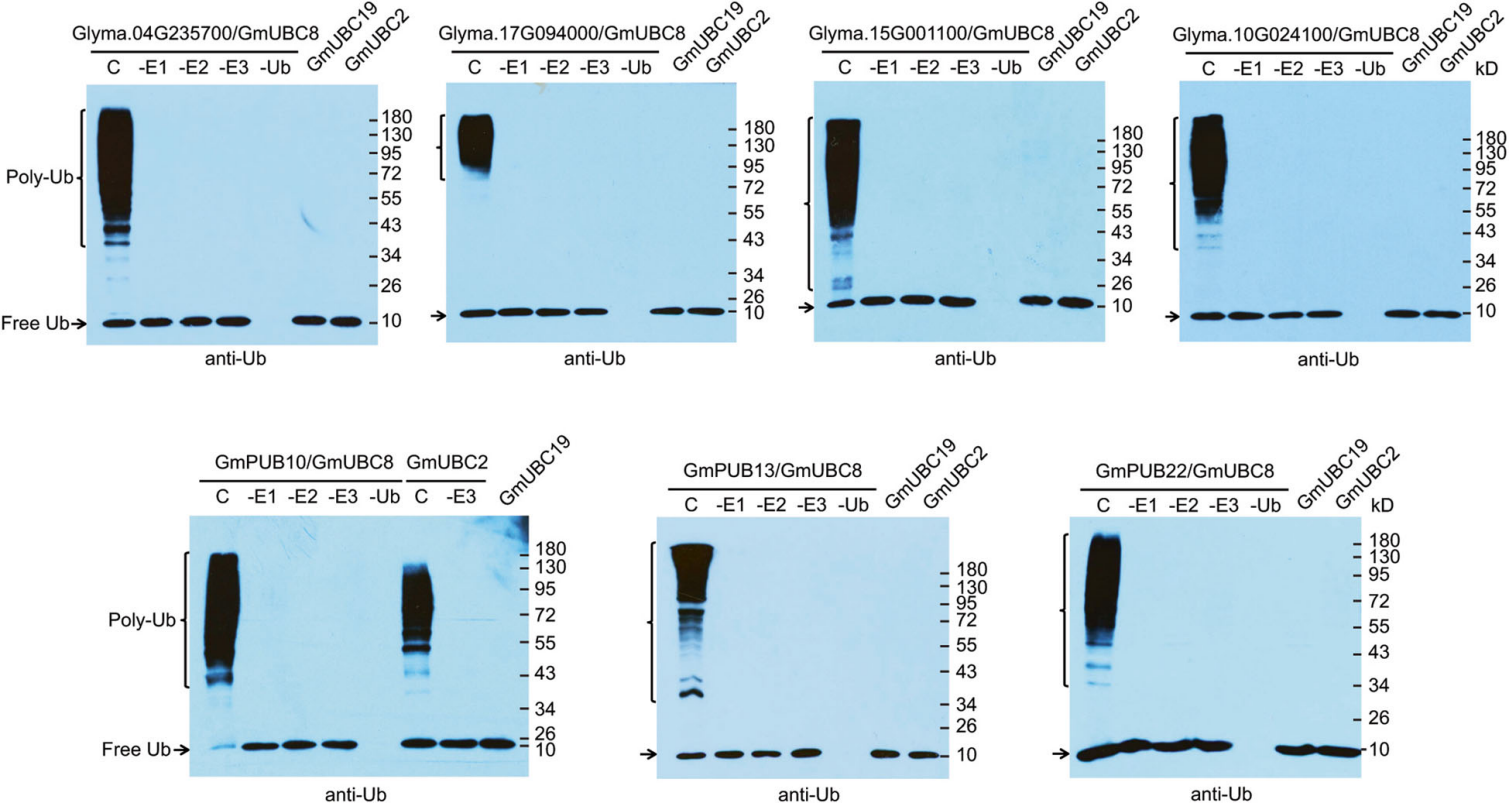
Analysis of E3 ligase activity for selected soybean E3 proteins by in vitro ubiquitination assay. Each of the E3 proteins was tested against active E2s, GmUBC8, GmUBC19 and GmUBC2 in the presence of GmUBA1and ubiquitin as shown in lanes that are labeled as C (complete reaction), GmUBC19 and GmUBC2. The lanes that are marked as -E1, −E2, −E3 and -Ub denote E1, E2, E3 and ubiquitin, respectively was absent in the corresponding reaction. Immunoblot with anti-Ubiquitin antibody (anti-Ub) was performed following in vitro ubiquitination assays. The numbers on the right indicate the molecular masses of marker proteins in kilo-Dalton (kD)

Previous studies have demonstrated RING and U-box domain-containing proteins generally function as E3 ubiquitin ligases [20, 29, 67]. The F-box protein serves as a subunit of the SCF (Skp1-Cullin-F-box) ubiquitin ligase complex. Unfortunately, method for examining E3 ligase activity of SCF complex in vitro is currently unavailable. To determine if the putative soybean RING and U-box proteins are capable of catalyzing protein ubiquitination, we performed in vitro ubiquitination assays. To this end, four RING protein-coding genes, *Glyma.04G235700*, *Glyma.17G094000*, *Glyma.15G001100* and *Glyma.10G24100*, and four U-box protein-coding genes, *Glyma.20G013200*, *Glyma.11G140100*, *Glyma.19G199300*, and *Glyma.04G179300* were randomly selected and cloned and their recombinant proteins were expressed and purified from *E. coli*. The U-box genes were named *GmPUB10* (*Glyma.20G013200*), *GmPUB13* (*Glyma.11G140100*), *GmPUB22* (*Glyma.19G199300*) and *GmPUB38* (*Glyma.04G179300*) based on their homology to *Arabidopsis PUB10* [68], *PUB13* [69], *PUB22* [70] and *PUB38* [67] gene, respectively (Additional file 17: Figure S13). Each RING or U-box protein cloned was examined in an in vitro ubiquitination assay consisting of soybean E1 GmUBA1, soybean E2 (GmUBC8, GmUBC2 or GmUBC19) and ubiquitin. As shown in Fig. 7, except for GmPUB38, all the tested putative RING and U-box E3 ligases catalyzed formation of high molecular weight polyubiquitin chains in the complete reaction that contained soybean E1 GmUBA1, E2 GmUBC8, free ubiquitin and necessary co-factors in the buffer, whereas no signal was detected in the control reactions that lacked either of the E1, E2, E3 and ubiquitin. Additionally, the U-box protein GmPUB10 was also able to work with GmUBC2 to catalyze ubiquitination, suggesting the E2-E3 specificity between GmUBC2 and GmPUB10 (Fig. 7). However, no polyubiquitin chain was detected in any complete reaction that GmUBC19 served as the E2, likely due to none of the tested E3s was able to work with this E2 enzyme to catalyze ubiquitination (Fig. 7). Similarly, the failure of GmPUB38 to catalyze in vitro ubiquitination likely due to none of GmUBC8, GmUBC2 or GmUBC19 is the bona fide cognate E2 for its E3 activity. These results demonstrated that the majority of the RING and U-box proteins we examined displayed E3 ubiquitin ligase activity, which validates the algorithms and protocols we used herein for the identification of core components of soybean UBS at the genome scale.

### Expression profile of soybean UBS genes during plant development and after stress treatments

**Fig. 8:**
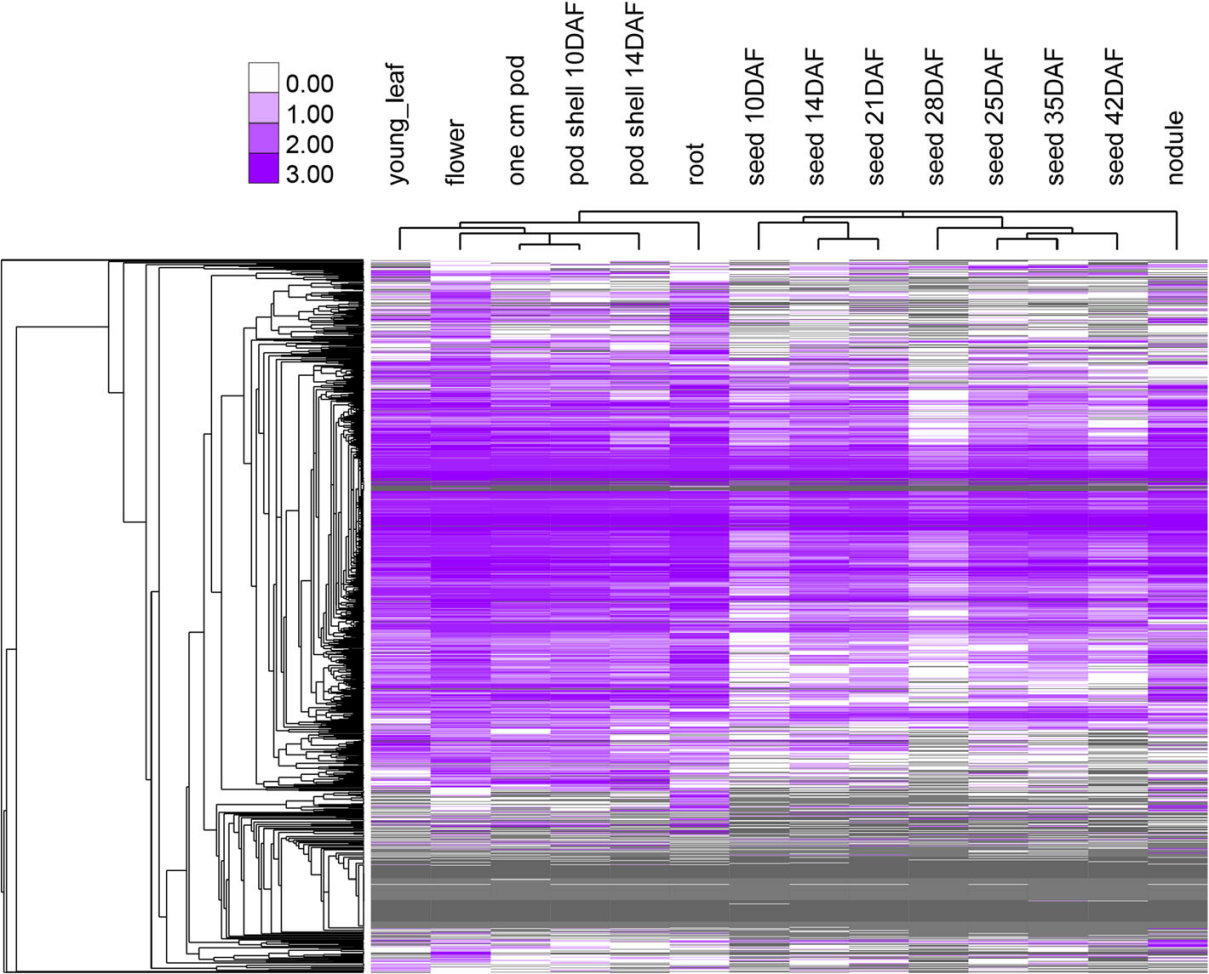
Heatmap of the expression profile for soybean UBS genes in fourteen tissues. The RNA-seq data were downloaded from the Soybase databases [71]. The color scale in the heatmap represents the log-RPKM (Reads Per Kilobase Million) values nomorlized in SoyBase. A complete list of the expression pattern of UBS genes is included in Additional file 18: Table S5

Gene expression patterns can provide important information for gene functions. We therefore explored the expression patterns of the soybean UBS genes using previous RNA-seq data that are publicly-available at Soybase [71]. Based on the dataset, we found the transcript of 1034 out of 1431 soybean UBS genes (72.3%) were detected in at least one of the 14 soybean plant tissues examined. Our analyses also showed 564 UBS genes were constitutively expressed in all 14 tissues, including 2 UBA genes (50% of total UBA genes), 50 UBC genes (70.4% of total UBC genes), 305 RING genes (40.1% of total RING genes), 30 U-box genes (24.2% of total U-box genes) and 177 F-box genes (37.5% of total F-box genes) (Fig. 8 and Additional file 18: Table S5). These results suggest that many UBS genes may be involved in multiple developmental processes in soybean.

To elucidate the potential roles of soybean UBS genes under biotic or abiotic stress, we analyzed the gene expression using previous RNA-seq datasets that were generated from experiments in which soybean plants were treated by various stresses [72–74]. Genes were considered differentially expressed if the fold changes are ≥2 or ≤ 0.5 between treated and control plants at a *P*-value of less than 0.05 (or false discovery rate ≤ 0.001 in the dataset that rhizobium strains were inoculated). The identified up-regulated and down-regulated genes are shown in Additional file 19: Figure S14 and Additional file 20: Table S6. The transcript level of 196, 45 and 112 soybean USB genes were significantly altered after abiotic stress (i.e. drought and salt) treatment, *Fusarium oxysporum* and rhizobium strains inoculation, respectively. Among them, the expression of 41 genes was significantly affected under both drought and salt, and 12 were in response to both *F. oxysporum* and rhizobium strains. Further analysis of the overlap between the biotic and abiotic stresses revealed that the transcript level of 3 RING-type E3 ligases (*Glyma.03G215500*, *Glyma.06G150400* and *Glyma.12G112000*) were significantly changed under all stresses tested.

### The expression level of many soybean UBS genes change significantly upon treatment with SCN

**Fig. 9:**
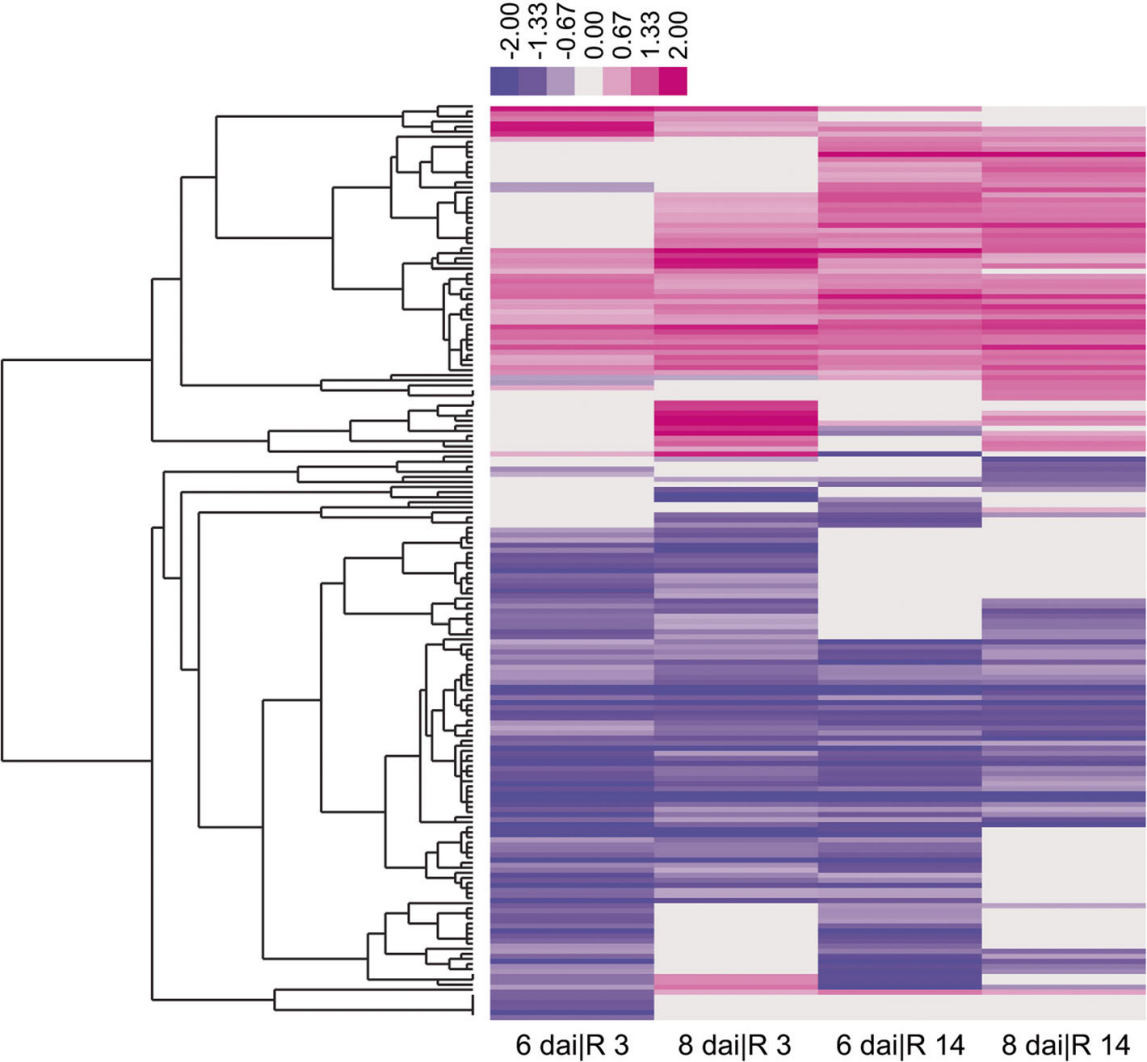
Heatmap of differentially expressed UBS genes after SCN treatment. Analysis of the expression of soybean UBS genes was performed using previous RNA-seq datasets [78]. Soybean whole root 6 and 8 days after independent inoculation (dai) with the SCN populations NH1-RHg (Race 3) and TN8 (Race 14), are denoted as 6 dai|R 3, 8 dai|R 3, 6 dai|R 14 and 8 dai|R 14 respectively. The color scale indicates the log-ratio calculated by comparing the expression value of the gene in inoculated sample to that in uninoculated sample. A complete list of these differentially expressed UBS genes is included in Additional file 21: Table S7

Ubiquitination has emerged in recent years as a key regulatory mechanism underlying plant immunity against many different pathogens [75–77]. To elucidate the possible role and mechanistic basis in the regulation of host immunity by soybean UBS, we explored publicly-available RNA-seq datasets to examine the transcriptional profiles of the soybean UBS genes in response to SCN treatments [78]. Such analysis would facilitate the identification of key candidates of the soybean UBS that are involved in host immunity. In the study from which the RNA-seq data was generated [78], the soybean root transcriptome at 6 and 8 days after inoculation (dai) with virulent (Race 3, R3) and avirulent (Race 14, R14) SCN races that led to a susceptible and resistant reaction of the host, respectively were sequenced and was subsequently compared to the transcriptome created from soybean roots uninoculated with SCN (as baseline control). There are thus four sets of data from four different treatments (6 dai|R3, 8 dai|R3, 6 dai|R14, 8 dai|R14) were generated by comparing experimental and uninoculated samples using the parameter Reads Per Kilobase Million (RPKM) [78]. We defined the significantly differentially expressed soybean UBS genes as those with a *log*_2_RPKM ≥ 1 or ≤ − 1 (i.e. more than 2-fold change in RPKM) in any of the dataset. Using the cutoff of 2*-*fold in RPKM, we found the transcription level of 180 soybean UBS genes were significantly altered after inoculation with SCN, accounting for approximately 12.6% of UBS genes in soybean (Fig. 9; Additional file 21: Table S7). These genes include 22 UBC genes (31.0% of total UBC genes), 91 RING genes (12.0% of total RING genes), 36 U-box genes (29.0% of total U-box) and 31 F-box genes (6.6% of total F-box genes).

**Fig. 10:**
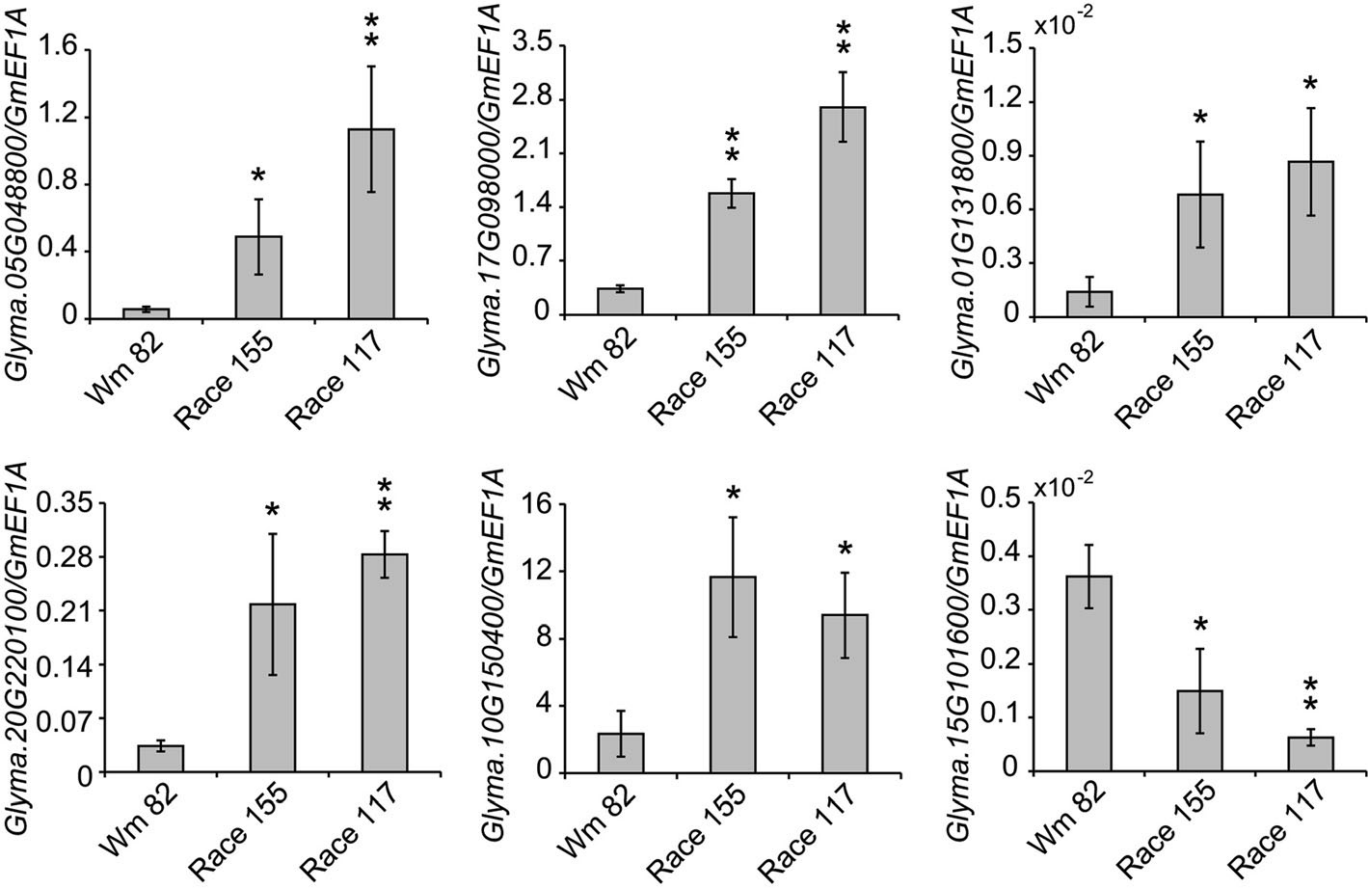
The differential expression patterns of soybean UBS genes revealed by RNA-seq analysis are validated by real-time quantitative PCR (qPCR). Roots from three-week-old soybean Williams 82 (Wm 82) plants were independently inoculated with two soybean cyst nematode (SCN, *Heterodera glycines* Ichinohe) populations, race 155 (HG Type 2.5.7) and race 117 (HG Type 1.2.3.5.6.7) for one week, and then collected for RNA extraction. The root of uninoculated Wm82 plants was used as control. Real time qPCR experiments were performed using the soybean *EF1a* gene (*GmEF1a*) as the internal control and mean values ± SD of three independent experiments were shown. Statistically significant differences were analyzed using Student’s t-test (* *p* < 0.05, ** *p* < 0.01)

To confirm the reliability of the RNA-seq results, we randomly selected ten genes out of the 180 soybean UBS genes and further examined their expression after SCN treatment using real time quantitative PCR (real time qPCR) analysis. Total RNA was generated from SCN-susceptible soybean cv. Williams 82 roots after independently inoculating with two soybean cyst nematode (SCN, *Heterodera glycines* Ichinohe) populations, race 155 (HG Type 2.5.7) and race 117 (HG Type 1.2.3.5.6.7). As shown in Fig. 10, the expression level of six soybean UBS genes were significantly altered after SCN treatment as detected by real time qPCR. The trends of the change (i.e. increase or decrease) in their expression were in consistence with the RNA-seq data, though the exact fold of change was different. However, we observed no obvious change in the expression level for the other four genes by real time qPCR after SCN treatment (Additional file 22: Figure S15). Based on these results, we postulate that the expression levels of approximately 100 soybean UBS gene (approximately 60% of the 180 genes) may be significantly altered during the soybean-SCN interactions. Taken together the results from RNA-seq and real time qPCR analyses, it is not illogical to conclude that dozens of the soybean UBS genes may be involved in the regulation of host immunity or susceptibility to the SCN infection.

## Discussion

A genome-wide identification of genes that encode core components of the soybean UBS would be an essential step towards further functional characterizations of these genes in soybean. Previous studies have reported identification of a few individual ubiquitin E3 ligase gene families in soybean, including the HECT [45], the RBR (a subset of RING) [46], the U-box [44] and the F-box [43] genes. However, a systematic identification and analysis of genes that encode the core components of the entire soybean (*Glycine max*) ubiquitin system (UBS) in soybean have hitherto not been performed. To address this knowledge gap, we performed a comprehensive identification and analysis of soybean UBS genes in this study. Through an array of bioinformatics protocols for gene identification and analyses of their corresponding proteins, we pinpointed with high confidence 4 ubiquitin E1-encoding genes, 71 ubiquitin E2-encoding genes and 1356 genes encoding components of three families of ubiquitin E3 ligases (including 760 RING genes, 124 U-box genes and 472 F-box genes) from the soybean genome using the latest soybean genome database Wm82.a2.v1.

Generally, the ubiquitin E1 enzymes are monomeric proteins with a molecular weight of 110–125 kDa and contain two ThiF motifs that is involved in adenylation [79]. In the present study, we identified 20 genes that encode ThiF motif-containing proteins from the soybean genome. However, only four of these genes encode proteins with a predicted molecular weight of more than 110 kDa and the presence of E1-specific catalytic Cys domain (UBA_e1_thiolCys) and ubiquitin-fold domain (UFD). The UBA_e1_thiolCys domain that is also called SCCH [12] contains a cysteine residue responsible for ubiquitin thioester linkage, while UFD confers specificity of E1 in recruiting ubiquitin E2s [20]. Consistent with the domain organization information, phylogenetic analysis indicated the ThiF motif-CCD-UFD domain-containing proteins encoded by the four soybean genes also fall into the same clade of known ubiquitin E1s in Arabidopsis and human. We also identified 71 ubiquitin E2 genes out of 91 UBC domain-containing genes in soybean. The 71 ubiquitin E2s were classified into 11 groups, I-XII except V according to the grouping of Arabidopsis counterparts [55]. We did not identify the homolog of AtUBC37 in the soybean genome. The absence of UBC37 and group V E2s in the list of soybean E2s identified in present study can either due to the genome is not completely sequenced thus the sequence for those genes are not presented in the soybean genome database or there are indeed no such E2s exist in the genome. The proteins encoded by a soybean ubiquitin E1 gene (*GmUBA1*) and four ubiquitin E2 genes (*GmUBC2*, *8*, *19* and *21*) were used to perform in vitro thioester assay. The results provide proofs that GmUBA1 functions as an active E1 enzyme to activate free ubiquitin to form thioester-linked ubiquitin. Thioester-linked ubiquitin is then transferred to the active E2 enzymes (GmUBC2, 8 and 19) to generate a thioester-linked E2-ubiquitin adduct.

The ubiquitin E3 ligases are the most diverse group in the ubiquitin system and are responsible for the substrate specificity of ubiquitination. Based on the mechanism of action and their structural features, E3 ligases can be grouped into single-subunit including HECT, RING and U-box-types [31] and multi-subunit including SCF (Skp1-Cullin-F-box), Cullin-Elongin-BC-VHL (CBC VHL) and the APC (Anaphase Promoting Complex) types [38]. We did not include in this study the identification of HECT-type of soybean E3s as previous evolutionary analysis indicated the number of HECT genes has been kept quite constant in different plant genomes and 19 were predicted in the soybean genome [28, 45]. Instead, we focused on the three types of E3 (RING, U-box and F-box) that constitute the largest groups of E3 in plant genomes. In present study, 760 RING genes were identified in the soybean genome, which is almost 2 times of the numbers found in other eukaryotes such as Arabidopsis, human and mouse genomes that encode for 469, 385 and 305 RING proteins, respectively [80, 81]. Recently, 24 genes encoding RBR (RING1-IBR-RING2; a subset of RING proteins) domain-containing proteins were identified from the soybean genome [46]. These RBR genes were among the 1234 RING genes identified by our HMMER3.1 analysis (Additional file 3: Table S3). However, only five of these RBR genes were among the list generated by BLAST against the NCBI & Pfam databases and none of them fall into the list after manual validation due to their highly atypical RING domain. The RING proteins that serve as a subunit of the Cullin-RING-like (CRL)-type of multiple-subunit ubiquitin E3s and the RBR proteins were also excluded from our final list of soybean RING E3s after manual validation. The Arabidopsis and rice (*Oryzae sativa*) genomes contain 64 and 77 predicted U-box proteins, respectively [29, 82]. Recently, 125 U-box genes were identified in the soybean genome [44]. Our analysis identified 124 U-box genes, of which 119 (96%) are among the list that was reported in that study [44], five extra U-box genes were revealed by our study but were missed in the former study, and three other U-box genes reported by the that study were eliminated from our list after manual validation (Additional file 3: Table S3) [44]. A close look of the three eliminated U-box genes revealed that the highly conserved amino acid residues at the N-terminus of the U-box domain were missed in the proteins encoded by those genes (data not shown). In plants, 694, 687, 337 and 156 F-box genes have been identified in Arabidopsis, rice, popular (*Populus trichocarpa*) and grape (*Vitis vinifera*), respectively [39, 83]. In present study, 472 F-box genes were identified, of which 440 (93%) were also identified by another group in a recent study [43]. However, 32 F-box genes revealed in present study were missed in that study whereas 64 F-box genes that were reported in that study were eliminated from our final list after manual validation in this study even though they are actually on the list after HMMER 3.1 analysis (Additional file 3: Table S3). The overlap of the vast majority of the E3 genes we identified in present study with the previous reports indicates the effectiveness of the algorithms we used for our genome-wide gene identification. On the other hand, we combined in present study the HMMER 3.1 analysis, protein domain detection tools in the NCBI and, Pfam databases, and manual validation for the identification of genes of interest, which is more stringent than previous studies that involved HMMER analysis and/or BLAST only and may explain why some of the genes identified in those studies are not on our final list.

So far, a few soybean RING and U-box proteins have been shown to possess E3 ubiquitin ligase activity [44, 84, 85]. However, commercially available, non-soybean E1 and E2s were used for the in vitro ubiquitination assays in those studies to examine the E3 activity. In this study, a ubiquitin E1 gene *GmUBA1* and three E2 genes *GmUBC2*, *8* and *19* are proved to encode active ubiquitin E1 and E2 enzymes by thioester assay but GmUBC21 failed to form adducts with ubiquitin in the assay. Similarly, the Arabidopsis homologs of GmUBC2, 8 and 19, AtUBC2, 8 and 19 have also been shown to carry thioester-linked ubiquitin [20] but AtUBC21 did not show E2 activity in thioester assay [20]. Using components of the soybean ubiquitin system, four RING proteins and three U-box proteins were tested to be true E3 ubiquitin ligases when GmUBC8 was employed as the cognate ubiquitin E2 enzyme. Similar to the Arabidopsis AtPUB10 that is capable of performing autoubiquitination using AtUBC2 as the cognate E2 enzyme [68], the soybean GmPUB10 was also found to display E3 activity in the presence of soybean E2 GmUBC2. Demonstration of these randomly selected proteins of the soybean UBS as enzymatically active validates the algorithms we used for the identification at genome scale of components that constitute the soybean UBS.

Gene expression analyses can provide key information about the potential functions of soybean UBS genes. Accordingly, we analyzed the expression profile of UBS genes during plant development and under abiotic and biotic stresses using publicly-available RNA-seq datasets. The transcript of 1034 UBS genes could be detected in at least one of 14 soybean tissues examined, further suggesting the effectiveness of the algorithms we used for our genome-wide gene identification. Meanwhile, the expression level of 338 soybean USB genes were significantly changed after either abiotic (drought and salt) or biotic (*F. oxysporum* and rhizobium strains) stress treatment, implying they may play a role in these processes. Among biotic stresses, SCN (*Heterodera glycines* Ichinohe) has consistently been a major pest on soybean worldwide, which cause soybean yield loss of 15–30% yearly. Breeding and planting SCN-resistant cultivars is the most effective strategy to control SCN [86]. There has hitherto been very limited study on the ubiquitin system (UBS) in soybean immunity against SCN and other pathogens. To expand our understanding of the functions of ubiquitination-related genes in soybean immunity, we examined their expression profiles after SCN treatment by employing publicly-available RNA-seq datasets [78]. Based on the analysis of the RNA-seq datasets, 180 soybean UBS genes including 22 E2 genes and 158 E3 genes were found to have significantly altered their abundance in transcripts after incubation with SCN. Among these genes identified by RNA-seq analysis, six out of ten randomly selected ones were validated by real time qPCR using the SCN-susceptible soybean cv. Williams 82 after incubation with SCN. These results support the notion that UBS likely plays an important role in soybean immunity against SCN. Until now most soybean cultivars being resistant to SCN are derived from limited resistance sources and SCN race has begun evolving to overcome the resistance [86]. Therefore, engineering novel SCN resistance may serve as an intriguing strategy for the management of SCN infection. To this end, pinpointing and characterizing members of the soybean UBS identified by present study that play key roles in soybean immunity should be the next experiments. Considering the omnipresence of ubiquitination in the regulation of plant growth, development, and biotic and abiotic stress responses, further functional characterization of the soybean UBS components identified in present study would also facilitate in-depth understanding of many other plant physiological processes.

## Conclusion

In this study, genes encoding core components of the soybean ubiquitin system (UBS) were systematically identified by an array of bioinformatics protocols. A total of 4 ubiquitin E1 genes, 71 ubiquitin E2 genes and 1356 E3 ligase genes were identified from the soybean genome. The presence of such a large and diverse number of UBS proteins suggests that target-specific modification by ubiquitin is a complex and important part of cellular and physiological regulation in soybean. More than a dozen of proteins encoded by the identified soybean E1, E2 and E3 genes were randomly selected for biochemical tests and the enzymatic activity was validated for the majority of them. Combined the analysis of RNA-seq data and real time qPCR results indicate that the expression level of a large number of soybean UBS genes changed significantly after the SCN treatment, which suggests the involvement of UBS components in the soybean-SCN interactions. The present study has built a foundation and presented an essential framework for further functional characterization of soybean UBS genes in various physiological processes, including their role and the underlying molecular mechanism in the regulation of soybean immunity against SCN.

## Methods

### Identification of soybean UBS genes

The search for ubiquitin E1 enzyme-coding genes in soybean was performed using a consensus sequence of ThiF motif as query and the BLASTP algorithm against the latest soybean proteome database (Phytozome 12.1, https://phytozome.jgi.doe.gov/pz/portal.html#!info?alias=Org_Gmax). The consensus sequence for the ThiF motif (PF00899) was downloaded from the NCBI CDD database (http://www.ncbi.nlm.nih.gov/cdd/). To confirm the obtained proteins, the Pfam database (http://pfam.xfam.org/) [50] was used to further examine the presence of ThiF motif in the candidate proteins.

To identify potential members of ubiquitin E2 enzymes and E3 ligases in soybean, the HMM profiles (Additional file 2: Table S2) of corresponding domains were downloaded from the Pfam database. The HMMER3.1 [87] program was then employed to search against the soybean proteome database (Wm82.a2.v1) at the Soybase (http://www.soybase.org/) [47, 71] using these HMM profiles as queries. The complete protein sequences were extracted from Soybase based on the HMMER search results, and then submitted to the Pfam and NCBI CDD databases to validate the presence of domains of interest. To finally determine these predicted proteins, we processed manual validation based on alignment of the sequence of domain of interest in candidate proteins and their corresponding consensus sequences that are downloaded from CDD database. Those proteins that lack the highly conserved key amino acids or secondary structures were excluded from the final dataset.

On the basis of the results of BLASTP searches in the soybean genome database of Phytozome, we obtained information on the chromosomal locations, cDNA sequences, CDS sequences, protein sequences, and alternative splicing events. The molecular weight was calculated using ProtParam (http://web.expasy.org/protparam/). The expressed sequence tag (EST) was identified by NCBI blast. If more than one transcript existed for a gene in the Soybase, the primary transcript was used for all subsequent analyses.

### Phylogenetic, sequence conservation and gene duplication analysis

The phylogenetic trees were constructed using MUSCLE aligned full-length amino acids sequences and the Neighbor-joining (NJ) method in the MEGA6 program with parameters of *p*-distance, gaps treated by partial deletion, and 1000 bootstrap replicates [88].

To analyze the sequence features of the domain of interest, the sequences of the corresponding domain in the predicted proteins were extracted based on NCBI blast results, and the consensus sequences of the UBC, RING, U-box, and F-box domain were downloaded from CDD database. The multiple sequence alignments were performed by CLUSTAL2.1 [89], and visualized using the ESPript3 (http://espript.ibcp.fr/ESPript/cgi-bin/ESPript.cgi) [90] and BoxShade (http://www.ch.embnet.org/software/BOX_form.html) [91]. The secondary structures were also generated by the ESPript3 according to the reference sequences. The sequence logos were produced from the multiple sequence alignment using the online program WebLogo3 (http://weblogo.threeplusone.com/create.cgi) [54] with the default parameters.

To inspect domain organization of the identified proteins, the amino acid sequences of the proteins with FASTA format were searched against the Pfam and CDD database. The information of conserved domains was extracted for analyzing the domain organization.

To analyze RING and F-box collinear paralogues, MCScanX was employed as previous described [92]. The highest scoring path was identified by dynamic programming with standard settings. Gene loci were classified as whole genome duplications (WGD) /segmental, tandem, proximal or dispersed duplications based on the number of matching hits and positions in chromosomes and scaffolds.

### Proteins expression and purification

The full-length coding sequences of the selected genes were cloned into the pDEST15 vector using the Gateway cloning system (Invitrogen), and transformed into the *E. coli* strain BL21 (DE3). The primers used for this assay are listed in Additional file 18: Table S5. GST-tagged fusion proteins were expressed in BL21 and purified using Glutathione Sepharose 4 Fast Flow beads (GE Healthcare) by following the protocol provided by the manufacturer. Briefly, the *E. coli* cells were harvested by centrifugation, suspended with lysis buffer (50 mM Tris-HCl (pH 7.5), 100 mM NaCl, 1 mM EDTA, 1% Triton X-100, 1 mg/mL lysozyme and cocktail), and disrupted using sonicator. For purification, 200 μL Glutathione Sepharose 4 Fast Flow beads was added to cleared supernatant and incubated on a rotator overnight at 4 °C. Beads were washed 3 times with 10 mL washing buffer (1 × PBS, 1 mM EDTA and 0.5% Triton X-100), and then eluted with 4 mL of elution buffer (10 mM reduced glutathione in 50 mM Tris-HCl pH 8.8). The purified proteins were further desalted and concentrated in the protein storage buffer (50 mM Tris-HCl, pH 8, 50 mM KCl, 0.1 mM EDTA, 1 mM DTT, and 0.5 mM PMSF) using the Amicon Centrifugal Filter (Millipore). Glycerol was added to the recombinant protein to a final concentration of 40% for storage at − 80 °C until being used. The concentration of purified protein was measured using protein assay agent (Bio-Rad).

### Thioester assay

The E1 ubiquitin-activating activity and E2 ubiquitin-conjugating activity were detected by in vitro thioester assays as previously described [19]. The assays were conducted in a total reaction volume of 20 μL, consisting of 20 mM Tris-HCl (pH 7.5), 10 mM MgCl_2_, and 1 mM ATP. 40 ng of soybean E1 (GST-GmUBA1) was preincubated with 2 μg of FLAG-ubiquitin in the 20 μL reaction at 28 °C for 10 min. An approximate 100 ng of GST-fused E2 protein was added into the reaction and continued for 15 min. The reactions were split into two half-volume after incubation and terminated by the addition of SDS sample buffer with 100 mM dithiothreitol (DTT) or 4 M urea sample buffer without DTT (−). The reactions were probed with mouse monoclonal anti-FLAG M2-peroxidase-conjugated antibody (Sigma-Aldrich) before being detected using an ECL kit (Pierce, now Thermo Fisher).

### In vitro ubiquitination assay

The in vitro ubiquitination assay was performed as described previously [19]. In briefly, in a total of 30 μL, 40 ng of soybean E1 (GST-GmUBA1), an approximate 100 ng of GST-fused E2, 2 μg of GST-E3 ligase and 2 μg of ubiquitin were combined in ubiquitination buffer (50 mM Tris-HCl (pH 7.5), 5 mM ATP, 5 mM MgCl_2_, 2 mM DTT, 3 mM creatine phosphate, and 5 μg/mL creatine phosphokinase). After 1.5 h at 30 °C, the reactions were terminated by adding SDS sample loading buffer with 100 mM DTT, and boiled at 100 °C for 5 min. Products of the reactions were separated by 10% SDS-PAGE gel and detected by immunoblot using mouse monoclonal anti-ubiquitin M2-peroxidase-conjugated (horseradish peroxidase) antibody (Sigma-Aldrich).

### Plant materials and gene expression analysis after SCN inoculation

Roots from three-week-old soybean Williams 82 plants were independently inoculated with two soybean cyst nematode (SCN, *Heterodera glycines* Ichinohe) populations, race 155 (HG Type 2.5.7) and race 117 (HG Type 1.2.3.5.6.7). One week after SCN inoculation, roots from three soybean plants were collected and immediately frozen in liquid nitrogen and ground to a fine powder for RNA extraction. Roots of three uninoculated Williams 82 plants were collected for the negative control.

Total RNA was extracted using the RNeasy Plant Mini Kit with DNase treatment (Qiagen) following the manufacturer’s procedure. Two micrograms of total RNA was then used as template for the first-strand cDNA synthesis in the presence of SuperScript III reverse transcriptase and oligo (dT) primer (Life Technologies). The cDNA population were diluted 10 times with sterilized ddH_2_O before being used for real time quantitative PCR (qPCR). The real time-qPCR was conducted on the LightCycler 480 Instrument II (Roche) with SYBR Green (Life Technologies) and gene-specific primers. The soybean *EF1a* gene, *GmEF1a* (*Glyma.19G052400*) was used as an internal control (Additional file 23: Table **S**8).

## Additional files

Additional file 1: **Table S1.** List of soybean ThiF motif-containing proteins. (DOCX 124 kb)

Additional file 2: **Table S2.** The HMM profiles used for present study. (DOCX 191 kb)

Additional file 3: **Table S3.** List of soybean UBC, RING, U-box and F-box domain-encoding genes identified. (XLSX 62 kb)

Additional file 4: **Figure S1.** Multiple sequence alignment of the UBC domain from the soybean UBC domain-containing proteins. (PDF 28 kb)

Additional file 5: **Figure S2.** Phylogenetic tree of the Arabidopsis and soybean UBC domain-containing proteins. (JPG 1759 kb)

Additional file 6: **Figure S3.** Phylogenetic tree of the soybean ubiquitin E2 proteins. (JPG 905 kb)

Additional file 7: **Figure S4.** Alignment of sequences of the RING domain from soybean RING domain-containing proteins. (PDF 88 kb)

Additional file 8: **Figure S5.** Alignment of sequences of the U-box domain from soybean U-box domain-containing proteins. (PDF 27 kb)

Additional file 9: **Figure S6.** Multiple sequence alignments of the F-box domain from the soybean F-box domain-containing proteins. (PDF 52 kb)

Additional file 10: **Figure S7.** Phylogenetic analysis of soybean RING domain-containing proteins. (PDF 187 kb)

Additional file 11: **Figure S8.** Phylogenetic analysis of soybean U-box domain-containing proteins. (PDF 31 kb)

Additional file 12: **Figure S9.** Phylogenetic analysis of soybean F-box domain-containing proteins. (PDF 118 kb)

Additional file 13: **Figure S10.** Duplication events of soybean RING genes. (PPTX 1668 kb)

Additional file 14: **Figure S11.** Duplication events of soybean F-box genes. (PPTX 1064 kb)

Additional file 15: **Table S4.** Summary of the number of soybean UBS genes underwent different types of duplications. (XLSX 9 kb)

Additional file 16: **Figure S12.** Examination of purified soybean E1 and E2 proteins using SDS-PAGE. (JPG 992 kb)

Additional file 17: **Figure S13.** Phylogenetic analysis of cloned soybean U-box domain-containing proteins and their homologs in Arabidopsis. (JPG 1220 kb)

Additional file 18: **Table S5.** Expression pattern of UBS genes as revealed by RNA-seq analysis. (XLSX 213 kb)

Additional file 19: **Figure S14.** Heatmap of differentially expressed soybean UBS genes after abiotic and biotic stress treatment. (JPG 1112 kb)

Additional file 20: **Table S6.** Soybean UBS genes that are differetially expressed under stresses as revealed by RNA-seq analysis. (XLSX 34 kb)

Additional file 21: **Table S7.** UBS genes that are differetially expressed after SCN treatment as revealed by RNA-seq analysis. (XLSX 29 kb)

Additional file 22: **Figure S15.** The expression level of selected soybean UBS genes identified in the RNA-seq analysis are not changed after SCN treatment. (JPG 1032 kb)

Additional file 23: **Table S8.** PCR primers used in this study. (DOCX 18 kb)
