# Supplementary material for: Genome-wide analysis of genes encoding core components of the ubiquitin system in soybean (Glycine max) reveals a potential role for ubiquitination in host immunity against soybean cyst nematode
Source: BMC Plant Biol. 2018 Jul 18;18:149. doi: 10.1186/s12870-018-1365-7 (PMC6052599; doi:10.1186/s12870-018-1365-7)
Supplement: Supplementary file 1 — Table S1. List of soybean ThiF motif-containing proteins. (DOCX 124 kb) [file 12870_2018_1365_MOESM1_ESM.docx]

**Supplemental Table 1. List of soybean ThiF motif-containing proteins.**

| **Gene Locus (Wm82.a2.v1)** | **Chromosome** | **mRNA** | **CDS (bp)** | **Protein length**  **(aa)** | **Molecular Weight (Da)** | **Alternative splicing events** |
| --- | --- | --- | --- | --- | --- | --- |
| Glyma.02G229700.1 | Chr02 | 4046 | 3279 | 1092 | 120541.68 | 2 |
| Glyma.02G229700.2 | Chr02 | 3970 | 3279 | 1092 | 120541.68 |  |
| Glyma.14G196800.1 | Chr14 | 3665 | 3402 | 1133 | 121018.28 | 3 |
| Glyma.14G196800.2 | Chr14 | 3897 | 3285 | 1094 | 121018.28 |  |
| Glyma.14G196800.3 | Chr14 | 3671 | 3285 | 1094 | 121018.28 |  |
| Glyma.18G058900.1 | Chr18 | 3751 | 3339 | 1112 | 123808.42 | 2 |
| Glyma.18G058900.2 | Chr18 | 3154 | 2403 | 800 | 89831.53 |  |
| Glyma.11G166100.1 | Chr11 | 3783 | 3285 | 1094 | 121892.90 | 1 |
| Glyma.05G204000.1 | Chr05 | 1645 | 996 | 331 | 36846.33 | 5 |
| Glyma.05G204000.2 | Chr05 | 1325 | 990 | 329 | 36647.18 |  |
| Glyma.05G204000.3 | Chr05 | 1552 | 957 | 318 | 35254.38 |  |
| Glyma.05G204000.5 | Chr05 | 1561 | 966 | 321 | 35632.87 |  |
| Glyma.05G204000.6 | Chr05 | 1863 | 675 | 224 | 25145.85 |  |
| Glyma.06G305800.1 | Chr06 | 2237 | 1572 | 523 | 57868.02 | 1 |
| Glyma.12G098800.1 | Chr12 | 2124 | 1572 | 523 | 57980.26 | 3 |
| Glyma.12G098800.2 | Chr12 | 2398 | 1494 | 497 | 55047.83 |  |
| Glyma.12G098800.3 | Chr12 | 2302 | 1494 | 497 | 55047.83 |  |
| Glyma.17G016000.1 | Chr17 | 3438 | 1341 | 446 | 49434.89 | 2 |
| Glyma.17G016000.2 | Chr17 | 3352 | 1341 | 446 | 49434.89 |  |
| Glyma.12G236000.1 | Chr12 | 2598 | 1191 | 636 | 70923.82 | 2 |
| Glyma.12G236000.2 | Chr12 | 2620 | 1785 | 594 | 66609.68 |  |
| Glyma.13G201500.1 | Chr13 | 2473 | 1917 | 638 | 70978.72 | 1 |
| Glyma.02G293600.1 | Chr02 | 1629 | 1344 | 447 | 48529.89 | 1 |
| Glyma.14G019100.1 | Chr14 | 1791 | 1374 | 457 | 49467.70 | 2 |
| Glyma.14G019100.2 | Chr14 | 1798 | 1008 | 335 | 36476.99 |  |
| Glyma.17G130200.1 | Chr17 | 1626 | 1281 | 426 | 46732.04 | 1 |
| Glyma.05G048200.1 | Chr05 | 1710 | 1281 | 426 | 46633.88 | 2 |
| Glyma.05G048200.2 | Chr05 | 1938 | 1068 | 355 | 38875.75 |  |
| Glyma.14G020700.1 | Chr14 | 1050 | 1050 | 349 | 37980.67 | 1 |
| Glyma.12G010000.1 | Chr12 | 2380 | 2061 | 686 | 75509.45 | 1 |
| Glyma.02G243700.1 | Chr02 | 1700 | 1515 | 504 | 56178.72 | 4 |
| Glyma.02G243700.2 | Chr02 | 1643 | 1458 | 485 | 54165.57 |  |
| Glyma.02G243700.3 | Chr02 | 1667 | 1482 | 493 | 55022.50 |  |
| Glyma.02G243700.4 | Chr02 | 1467 | 1209 | 402 | 45040.56 |  |
| Glyma.14G212800.1 | Chr14 | 1779 | 1377 | 458 | 51051.68 | 1 |
| Glyma.07G258000.1 | Chr07 | 1746 | 1341 | 446 | 49517.01 | 1 |
| Glyma.08G011400.1 | Chr08 | 1582 | 846 | 281 | 31136.90 | 1 |
