# Supplementary material for: Genome-wide analysis of genes encoding core components of the ubiquitin system in soybean (Glycine max) reveals a potential role for ubiquitination in host immunity against soybean cyst nematode
Source: BMC Plant Biol. 2018 Jul 18;18:149. doi: 10.1186/s12870-018-1365-7 (PMC6052599; doi:10.1186/s12870-018-1365-7)
Supplement: Supplementary file 2 — Table S2. The HMM profiles used for present study. (DOCX 191 kb) [file 12870_2018_1365_MOESM2_ESM.docx]

**Supplemental Table 2. The HMM profiles used for present study**

| **Proteins** | **The HMM files**  **of domain** | **The websites for downloading** | **Numbers for**  **seed alignments** |
| --- | --- | --- | --- |
| **UBC** | UBC domain (PF00179) | <http://pfam.xfam.org/family/PF00179/hmm> | 65 |
| **RING** | zf-C3HC4 (PF00097) | <http://pfam.xfam.org/family/PF00097/hmm> | 35 |
|  | zf-RING_2 (PF13639) | <http://pfam.xfam.org/family/PF136397/hmm> | 322 |
|  | zf-C3HC4 _2 (PF13923) | <http://pfam.xfam.org/family/P13923/hmm> | 341 |
|  | zf-C3HC4 _3(PF13920) | <http://pfam.xfam.org/family/PF13920/hmm> | 406 |
|  | zf-C3HC4 _4 (PF15227) | <http://pfam.xfam.org/family/PF15527/hmm> | 11 |
|  | zf-C3HC4 _5 (PF17121) | <http://pfam.xfam.org/family/PF17121/hmm> | 7 |
|  | zf-RING_4 (PF14570) | <http://pfam.xfam.org/family/PF14570/hmm> | 54 |
|  | zf-RING_5 (PF14634) | <http://pfam.xfam.org/family/PF14634/hmm> | 90 |
|  | zf-RING_6 (PF14835) | <http://pfam.xfam.org/family/PF14835/hmm> | 1 |
|  | zf-RING_9 (PF13901) | <http://pfam.xfam.org/family/PF13901/hmm> | 126 |
|  | zf-RING_10 (PF16685) | <http://pfam.xfam.org/family/PF16685/hmm> | 17 |
|  | zf-RING_11 (PF17123) | <http://pfam.xfam.org/family/PF17123/hmm> | 150 |
|  | RING_like (PF08746) | <http://pfam.xfam.org/family/PF08746/hmm> | 114 |
|  | RING_UBOX (PF13445) | <http://pfam.xfam.org/family/PF13445/hmm> | 77 |
|  | RINGv (PF12906) | <http://pfam.xfam.org/family/PF12906/hmm> | 21 |
|  | LIM (PF00412) | <http://pfam.xfam.org/family/PF100412/hmm> | 34 |
|  | PHD (Cys4-His-Cys3 )  (PF00628) | <http://pfam.xfam.org/family/PF00628/hmm> | 74 |
|  | zf-C3H2C3 (PF17122) | http://pfam.xfam.org/family/PF17122/hmm | 3 |
| **U-box** | U-box domain (PF04564) | <http://pfam.xfam.org/family/PF04564/hmm> | 16 |
| **F-box** | F-box domain (PF00646) | <http://pfam.xfam.org/family/PF00646/hmm> | 444 |
|  | F-box associated region  (FBA, PF04300) | <http://pfam.xfam.org/family/PF04300/hmm> | 80 |
|  | F-box associated region  (FBA_1, PF07734) | <http://pfam.xfam.org/family/PF07734/hmm> | 99 |
|  | F-box associated  (FBA_2, PF07735) | <http://pfam.xfam.org/family/PF07735/hmm> | 558 |
|  | F-box associated domain  (FBA_3, PF08268) | <http://pfam.xfam.org/family/PF08268/hmm> | 50 |
|  | FBD (PF08387) | <http://pfam.xfam.org/family/PF08387/hmm> | 329 |
|  | F-box-like (PF12937) | <http://pfam.xfam.org/family/PF12937/hmm> | 336 |
|  | F-box-like­2 (PF13013) | <http://pfam.xfam.org/family/PF13013/hmm> | 6 |
