# Supplementary material for: Genome-wide analysis of genes encoding core components of the ubiquitin system in soybean (Glycine max) reveals a potential role for ubiquitination in host immunity against soybean cyst nematode
Source: BMC Plant Biol. 2018 Jul 18;18:149. doi: 10.1186/s12870-018-1365-7 (PMC6052599; doi:10.1186/s12870-018-1365-7)
Supplement: Supplementary file 4 — Figure S1. Multiple sequence alignment of the UBC domain from the soybean UBC domain-containing proteins. (PDF 28 kb) [file 12870_2018_1365_MOESM4_ESM.pdf]

```
                  α1                      β1                β2
UBE2D2            ℓℓℓℓℓℓℓℓℓℓ.ℓ          ..........TT              TT   TT
                  1        10          20                 30          40
UBE2D2                      .RIHKELNDLA.RDPP.AQCSAGP..........VGDDMFHWQATIMGPNDSPYQGGVFF
consensus                   .RLLKELKELR.KDPP.PGFTAYP..........VDDENLLEWTGTIVGPPGTPYEGGVFK
lcl|Glyma.04G089600_33-128  ..LKEEYKALI.......AYTQMNK..........SNDNDWFRISAANPEGTRWTGKCWY
lcl|Glyma.06G091400_33-128  ..LKEEYKALI.......AYTQMNK..........SNDNDWFRISAANPEGTRWTGKCWY
lcl|Glyma.02G199700_17-143  ..LLEELERGEKGIGDGTVSYGMDD........GDDIYMRSWTGTIIGPHNTVHEGRIYQ
lcl|Glyma.10G077600_17-143  ..LLEELERGEKGIGDGTVSYGMDD........GDDIYMRSWTGTIIGPHNTVHEGRIYQ
lcl|Glyma.03G199900_17-146  ..LLEELEKGEKGIGDGTVSYGMDD........GDDIYMRSWTGTIIGPYNTVHEGRIYQ
lcl|Glyma.19G197500_17-154  ..LLEELEKGEKGIGDGTVSYGMDD........GDDIYMRSWTGTIIGPYNTVHEGRIYQ
lcl|Glyma.03G113100_5-108   ..LLKEIERGEKGIGDGTVNYGMDD........GDDIYMRSWT...IGPHNAVHEGRIYQ
lcl|Glyma.15G189500_8-136   ..LLEELERGEKGIRDGTISYVMDD........GDDIYMRSRTGTIIGPQNTIHEGKIYQ
lcl|Glyma.05G235000_15-150  ..LLEELERGEKGIGDGTVSYGMDD........ADDIYMQSWTGTIIGPPGTVHEGRIYQ
lcl|Glyma.08G042500_15-150  ..LLEELERGEKGIGDGTVSYGMDD........ADDIYMQSWTGTIIGPPGGVHEGRIYQ
lcl|Glyma.07G168100_15-150  ..LLEELERGEKGIGDGTVSYGMDD........ADDIYMQSWTGTIIGPPSTVHEGRIYQ
lcl|Glyma.09G124800_15-150  ..LLQELERGEKGIGDGTVSYGMDD........ADDIYMQSWTGTIIGPPGTVHEGRIHQ
lcl|Glyma.01G178200_33-167  ..LHKDISELNLPKSCTMQFPNGK............DDLMNFEVSIR.PDDGYYLGGTFL
lcl|Glyma.11G064000_33-167  ..LHKDISELNLPKSCTMQFPNGK............DDLMNFEVSIR.PDDGYYLGGTFL
lcl|Glyma.01G043600_7-145   .RLMRDFKRLQ.LDP.PAGISGAPQ..........DNNIMLWNAVIFGPDDTPWDGGTFK
lcl|Glyma.02G020000_7-145   .RLMRDFKRLQ.LDP.PAGISGAPQ..........DNNIMLWNAVIFGPDDTPWDGGTFK
lcl|Glyma.04G173300_7-145   .RLMRDFKRLQ.QDP.PAGISGAPQ..........DNNIMLWNAVIFGPDDTPWDGGTFK
lcl|Glyma.05G028800_7-145   .RLMRDFKRLQ.QDP.PAGISGAPQ..........DNNIMLWNAVIFGPDDTPWDGGTFK
lcl|Glyma.17G098000_7-145   .RLMRDFKRLQ.QDP.PAGISGAPQ..........DNNIMLWNAVIFGPDDTPWDGGTFK
lcl|Glyma.08G297300_7-145   .RLMRDFKRLQ.QDP.PAGISGAPQ..........DNNIMLWNAVIFGPDDTPWDGGTFK
lcl|Glyma.18G124600_7-111   .RLMRDFKRLQ.QDP.PAGISGAPQ..........DNNIMLWNAVIFGPDDTPWDGGTFK
lcl|Glyma.10G212200_39-178  QRLQKELMALM.MSGGDLGVSAFPD..........GESIFTWIGTIEGGKGTQYEGLSYK
lcl|Glyma.20G178900_39-174  QRLQKELMTLM.MSGGDLGVSAFPD..........GESIFTWIGTIEGGKGTLYEGLSYK
lcl|Glyma.11G095700_39-155  .RLQSELMALM.MSG.DSGISAFPE..........EDNIFFWKGTITGSKDTVFEGTEYK
lcl|Glyma.12G021800_39-176  .RLQSELMALM.MSG.DSGISAFPE..........EDNIFFWKGTITGSKDTVFEGTEYK
lcl|Glyma.05G091100_9-153   ..LTEERKSWR.KNH.PHGFVAKPETL.....PDGTVNLMVWHCTIPGKTGTDWEGGYFP
lcl|Glyma.17G169700_9-153   ..LTEERKSWR.KNH.PHGFVAKPETL.....PDGTVNLMVWHCTIPGKTGTDWEGGYFP
lcl|Glyma.11G053300_8-150   ..LAEERKSWR.KNH.PHGFVAKPETL.....PDGTVNLMVWHCTIPGKTGTDWEGGYFP
lcl|Glyma.01G188900_8-150   ..LAEERKSWR.KNH.PHGFVAKPETL.....PDATVNLMVWHCTIPGKAGTDWEGGYFP
lcl|Glyma.01G169700_17-117  NRLQKELVEWQ.VNP.PTGFKHK...........VTDNLQRWVVEVTGAPGTLYANETYQ
lcl|Glyma.04G199200_6-145   ..VQKELVECS.KDAEGSGIKVCP..........KSD.NLVLLIGTIPGPVGTPYEGGIFQ
lcl|Glyma.06G166300_6-145   ..VQKELVECS.KDAEGSGIKVSP..........KND.NLVLLIGTIPGPVGTPYEGGIFQ
lcl|Glyma.06G230300_7-146   RRIIKETQRLL.SEPA.PGISASP..........SEE.NMRYFNVMILGPTQSPYEGGVFK
lcl|Glyma.12G161200_7-146   RRIIKETQRLL.SEPA.PGISASP..........SEE.NMRYFNVMILGPTQSPYEGGVFK
lcl|Glyma.13G270800_7-146   RRIIKETQRLL.SEPA.PGISASP..........SED.NMRYFNVMILGPTQSPYEGGVFK
lcl|Glyma.12G228900_7-108   RRIIKETQRLL.SEPA.PGISASP..........SED.NMRYFNVMILGPTQSPYEGGVFK
lcl|Glyma.04G238800_3-142   KRILKELKDLQ.KDPP.TSCSAGP..........VAEDMFHWQATIMGPPDSPYAGGVFL
lcl|Glyma.13G040500_3-143   KRILKELKDLQ.KDPP.TSCSAGPV.........VAEDMFHWQATIMGPPDSPYAGGVFL
lcl|Glyma.06G124900_3-142   KRILKELKDLQ.KDPP.TSCSAGP..........VAEDMFHWQATIMGPPDSPYAGGVFL
lcl|Glyma.14G124300_3-142   KRILKELKDLQ.KDPP.TSCSAGP..........VHEDMFHWQATIMGPPDSPYAGGVFL
lcl|Glyma.03G255400_3-142   KRILKELKDLQ.KDPP.TSCSAGP..........VAEDMFHWQATIMGPPDSPYAGGVFL
lcl|Glyma.19G253000_3-142   KRILKELKDLQ.KDPP.TSCSAGP..........VAEDMFHWQATIMGPPDSPYAGGVFL
lcl|Glyma.02G236900_3-142   KRILKELKDLQ.KDPP.TSCSA..........GPVAEDMFHWQATIMGPADSPYAGGVFL
lcl|Glyma.14G205700_3-142   KRILKELKDLQ.KDPP.TSCSA..........GPVAEDMFHWQATIMGPADSPYAGGVFL
lcl|Glyma.08G267400_3-142   KRINKELKDLQ.KDPP.TSCSA..........GPVADDMFHWQATIMGPADSPFAGGVFL
lcl|Glyma.16G103100_4-142   .RINTELKDLQ.KDPP.VSCSA..........GPVTNDMFHWQATIMGPANSPFAGGVFL
lcl|Glyma.11G199400_3-142   KRIVKELKDLQ.KDPP.TSCSA..........GPVAEDMFHWQATIMGPPDSPYTGGVFL
lcl|Glyma.18G051400_3-142   KRIVKELKDLQ.KDPP.TSCSA..........GPVAEDMFHWQATIMGPPDSPYTGGVFL
lcl|Glyma.09G273100_3-142   KRILKELKDLQ.KDPP.TSCSA..........GPVAEDMFHWQATIMGPADSPYAGGVFL
lcl|Glyma.18G216000_3-152   KRILKELKDLQ.KDPP.TSCSAVCANTNVYFVGPVAEDMFHWQATIMGPADSPYAGGVFL
lcl|Glyma.11G107800_3-142   KRILKELKDLQ.RDPP.TSCSAGP..........VGEDMFHWQATIIGPNDSPYAGGVFL
lcl|Glyma.12G032800_3-142   KRILKELKDLQ.RDPP.TSCSAGP..........VGEDMFHWQATIIGPNDSPYAGGVFL
lcl|Glyma.10G096200_3-142   KRITKELKDLQ.QDPP.VSCSAGP..........VGDDMFHWQATIMGPTDSPYAGGVFL
lcl|Glyma.08G113700_33-171  .RIQREMVELN.NDPP.PDCSAGP..........KGDNLYHWIATIIGTPETPYQGGIFF
lcl|Glyma.03G004200_12-151  KQLAKELKSLD..ESPPEGIKVVVN..........DDDFSTIFSDIEGPAGTPYENGVFR
lcl|Glyma.19G121700_12-151  KQLAKELKNLD..ESPPEGIKVVVN..........DDDFSTIFADIEGPAGTPYENGVFR
lcl|Glyma.19G072800_12-151  KQLAKELKNID..ESPPEGIKVVVN..........DDDFSIIYADIEGPAGTPYDNGVFR
lcl|Glyma.04G081200_7-148   ..LFKEYKEVQREKAVDLDIQLVCD..........DSNIFKWTALIKGPSETPYEGGVFQ
lcl|Glyma.06G082900_7-148   ..LFKEYKEVQREKAVDPDIQLVCD..........DSNIFKWTALIKGPSETPFEGGVFQ
lcl|Glyma.05G035700_7-143   ..REMDVMKLMMSDYTVETINDG................LNEFNVEFHGPKESLYEGGVWK
lcl|Glyma.17G091700_7-143   ..REMDVMKLMMSDYAVETINDG................LNEFNVEFHGPKESLYEGGVWK
lcl|Glyma.05G209400_10-143  .....DLMKLMMSDYKVETINDG................MQEFFVEFHGPKDSPYQGGVWK
lcl|Glyma.08G016100_10-143  .....DLMKLMMSDYKVETVNDG................MQEFFVEFHGPKDSPYQGGVWK
lcl|Glyma.07G069300_7-143   ..REMDLMKLMMSDYKVEMINDG................MQEFYVHFHGPNESPYHGGVWK
lcl|Glyma.16G035000_7-143   ..REMDLMKLMMSDYKVEMINDG................MQEFYVHFHGPNESPYHGGVWK
lcl|Glyma.09G257800_18-145  ..........MSDYKVEMINDG................MQEFYVQFHGPNDSPYHGGVWK
lcl|Glyma.18G234800_7-143   ..REMDLMKLMMSDYKVEMINDG................MQEFYVQFHGPNDSPYHGGVWK
lcl|Glyma.05G140200_172-318 .KIQEEWKILEEN.LPETIFVRVC...........ESRMELLRAVIIGPQGTPYHDGLFF
lcl|Glyma.08G095600_194-340 .KIQEEWKILEEN.LPETIFVRVS...........ESRMELLRAVIIGPQGTPYHDGLFF
lcl|Glyma.12G153900_92-241  .RIQGEWKSLEKD.LPDSIFVRVY...........ESRIDLLRAVIIGAKGTPYHDGLFF
lcl|Glyma.12G154000_8-156   .KIQGEWKSLEKD.LPDSIFVRVY...........ESRIDLLRAVIIGAEGTPYHDGLFF
lcl|Glyma.06G243300_493-619 .RIQGEWKSLEKD.LPDSIFVRVY...........ESRIDLLRAVIIAAEGTPYHDGLFF
lcl|Glyma.12G224200_443-569 .KIQEEWRILEKD.LPASIFVRVF...........ESRMDLLRAVIIGAEGTPYHDGLFF
lcl|Glyma.13G277400_462-588 .KIQEEWRILEKD.LPASIFVRVY...........ESRMDLLRAVIIGAEGTPYHDGLFF
lcl|Glyma.13G239100_672-825 KKVQQEWSILEKN.LPETIYVRVF...........EERMDLMRAAIVGASGTPYHDGLFF
lcl|Glyma.15G074200_677-830 KKLQQEWSILEKN.LPETIYVRVF...........EERMDLMRAAIVSASGTPYHDGLFF
lcl|Glyma.13G179600_679-832 KKVQQEWSILEKN.LPETIYVRVF...........EERMDLMRAAIVGASGTPYHDGLFF
lcl|Glyma.07G196500_680-833 KKVQQEWSILEKN.LPETIYVCAF...........EERMDLMRAAIVGASGTPYHEGLFF
lcl|Glyma.10G282000_880-1030 .KVQQDWSILQNN.LPEEIYVRVY...........EDRMDLLRAVIVGPYGTPYQDGLFF
lcl|Glyma.20G107300_876-1026 .KVQQDWSILQNN.LPEEIYVRVY...........EDRMDLLRAVIVGPYGTPYQDGLFF
lcl|Glyma.07G239100_38-188  RTIMKEWKILEQN.LPESIYVRVY...........ERRIDLMRAVIVGAAGTPYHDGLFF
lcl|Glyma.17G034200_47-149  RTIMREWRILEHN.LPESIYVRVY...........ERRIDLMRAVIVGAAGTPYHDGLFF
lcl|Glyma.17G034300_1-145   ....KEWKILEQS.LPESIYVRVY...........ERRIDLMRAVIVGTAGTPYHDGLFF
lcl|Glyma.17G034400_40-142  KRIMMEWKILANNNLPESIYVRVY...........ANRIDLLRAVIVGAAGTPYHDGLFF
lcl|Glyma.07G046200_7-157   KRLQKEYRALC..KEPVSHVVARPS..........PSDILEWHYVLEGSEGTPFAGGYYY
lcl|Glyma.16G014400_7-157   KRLQKEYRALC..KEPVSHVVARPS..........PSDILEWHYVLEGSEGTPFAGGYYY
lcl|Glyma.07G240600_12-127  KRILQEVKEMQ..SNPSDDYMSLPL..........EENIFEWQFAIRGPRDTEFEGGIYH
lcl|Glyma.17G032800_12-151  KRILQELKEMQ..SNPSDDYLSLPL..........EENIFEWQFAIRGPRDTEFEGGIYH
lcl|Glyma.15G141200_12-127  KRILQEVKEMQ..SNPSDDFMSLPL..........EENIFEWQFAIRGPRDTEFEGGIYH
lcl|Glyma.09G036300_12-127  KRILQELKEMN..SNPSDDFMSLPL..........EENIFEWQFAIRGPRDTEFEGGIYH
lcl|Glyma.11G099100_34-153  RQHLVALTTAFPSLEPKTASFTHND..........GRSVNLLQADGTIPMTFQGVTYNIP
                                      1
```

```
                              β3            β4                          η1
UBE2D2                        --->    TTT   --->            TT  T.T    ~~~            TT
                               50       60               70         80                90
UBE2D2                        LTIHFPTDYPFKPPKVAFTT......RIYHPNINS.NGSICLDILR.SQ......WSPAL
consensus                     LTIEFPEDYPFKPPKVKFIT......KIYHPNVDS.SGEICLDILKQEK......WSPAL
lcl|Glyma.04G089600_33-128    VYNLLKYEFD.....LQFDV......PVTYPSTAP...ELELPQLDGKT.......QKMY
lcl|Glyma.06G091400_33-128    VHNLLKYEFD.....LQFDV......PVTYPSTAP...ELELPQLDGKT.......QKMY
lcl|Glyma.02G199700_17-143    LKLFCDKDYPEKPPSVRFHS......RINMTCVNHETGVVEPKKFGLLAN.....WQREY
lcl|Glyma.10G077600_17-143    LKLFCDKDYPEKPPSVRFHS......RVNMTCVNHETGVVEPKKFGLLAN.....WQREY
lcl|Glyma.03G199900_17-146    LKLFCDKDYPEKPPSVRFHS......RINMTCVNHETGVVESKKFGLLAN.....WQRRF
lcl|Glyma.19G197500_17-154    LKLFCDKDYPEKPPSVRFHS......RINMTCVNHETGVVESKKFGLLAN.....WQQRF
lcl|Glyma.03G113100_5-108     LKLFCDKDYPEKSPSVRFHS......RINMTGVNHETEVVEPKKFGLLEN.....WQREY
lcl|Glyma.15G189500_8-136     LKLFGDKDYPEKPPSVRFHS......RINMTCVNHETGEVEPKKFGLLAN.....WQREY
lcl|Glyma.05G235000_15-150    LKLFCGKDYPDNPPTVRFQT......RINMTCVNQETGVVEPHLFPMLAN.....WQREC
lcl|Glyma.08G042500_15-150    LKLFCGKDYPDNPPTVRFQT......RINMTCVNQETGVVEPHLFPMLAN.....WQREC
lcl|Glyma.07G168100_15-150    LKLFCSRDYPDNPPSVRFQT......RINMTCVNQETGVVEPHLFPMLAN.....WKRER
lcl|Glyma.09G124800_15-150    LKLFCGKDYPDNPPSVRFQT......RINMTCVNQETGAVEPHLFPMLAN.....WKREH
lcl|Glyma.01G178200_33-167    FSFQVSPIYPHEAPKVKCKT......KVYHPNIDL.EGNVCLNILR..ED.....WKPVL
lcl|Glyma.11G064000_33-167    FSFQVSPIYPHEAPKVKCKT......KVYHPNIDL.EGNVCLNILR..ED.....WKPVL
lcl|Glyma.01G043600_7-145     LTLQFIEDYPNKPPAVRFVS......QMFHPNIYA.DGSICLDILQNQ.......WSPIY
lcl|Glyma.02G020000_7-145     LTLQFTEDYPNKPPTVRFVS......QMFHPNIYA.DGSICLDILQNQ.......WSPIY
lcl|Glyma.04G173300_7-145     LTLQFTEDYPNKPPTVRFVS......RMFHPNIYA.DGSICLDILQNQ.......WSPIY
lcl|Glyma.05G028800_7-145     LTLQFTEDYPNKPPTVRFVS......RMFHPNIYA.DGSICLDILQNQ.......WSPIY
lcl|Glyma.17G098000_7-145     LILQFTEDYPNKPPTVRFVS......RMFHPNIYA.DGSICLDILQNQ.......WSPIY
lcl|Glyma.08G297300_7-145     LTLQFTEDYPNKPPTVRFVS......RMFHPNIYA.DGSICLDILQNQ.......WSPIY
lcl|Glyma.18G124600_7-111     LTLQFTEDYPNKPPTVRFVS......RMFHPNIYA.DGSICLDILQNQ.......WSPIY
lcl|Glyma.10G212200_39-178    LSLRFPLDYPFKPPQVKFET......MCFHPNVDQ.FGNICLDILQDK.......WSSAY
lcl|Glyma.20G178900_39-174    LSLRFPLDYPFKAPQVKFET......MCFHPNIDQ.FGNICLDILQDK.......WSSAY
lcl|Glyma.11G095700_39-155    LSLSFPNDYPFKPPKVKFET......TCFHPNFDV.HGNICLDILQDK.......WSSAY
lcl|Glyma.12G021800_39-176    LSLSFPNDYPFKPPKVKFET......TCFHPNFDV.HGNICLDILQDK.......WSSAY
lcl|Glyma.05G091100_9-153     LTLHFSEDYPSKPPKCKFPQ......GFFHPNVYP.SGTVCLSILNEDSG.....WRPAI
lcl|Glyma.17G169700_9-153     LTLHFSEDYPSKPPKCKFPQ......GFFHPNVYP.SGTVCLSILNEDSG.....WRPAI
lcl|Glyma.11G053300_8-150     LTMHFSEDYPSKPPKCKFPQ......GFFHPNVYP.SGTVCLSILNEDSG.....WRPAI
lcl|Glyma.01G188900_8-150     LTMHFSEDYPSKPPKCKFPQ......GFFHPNVYP.SGTVCLSILNEDSG.....WRPAI
lcl|Glyma.01G169700_17-117    LQVDFPENYPMEAPQVIFLHP.....APLHPHIYS.NGHICLDILYDS.......WSPAM
lcl|Glyma.04G199200_6-145     IDITLPDGYPFEPPKMKFKT......KVWHPNISSQSGAICLDILK.DQ......WSPAL
lcl|Glyma.06G166300_6-145     IDITLPDGYPFEPPKMQFKT......KVWHPNISSQSGAICLDILK.DQ......WSPAL
lcl|Glyma.06G230300_7-146     LELFLPEEYPMAAPKVRFLT......KIYHPNIDK.LGRICLDILK.DK......WSPAL
lcl|Glyma.12G161200_7-146     LELFLPEEYPMAAPKVRFLT......KIYHPNIDK.LGRICLDILK.DK......WSPAL
lcl|Glyma.13G270800_7-146     LELFLPEEYPMAAPKVRFLT......KIYHPNIDK.LGRICLDILK.DK......WSPAL
lcl|Glyma.12G228900_7-108     LELFLPEEYPMAAPKVRFLT......KIYHPNIDK.LGRICLDILK.DK......WSPAL
lcl|Glyma.04G238800_3-142     VTIHFPPDYPFKPPKVAFRT......KVFHPNINS.NGSICLDILK.EQ......WSPAL
lcl|Glyma.13G040500_3-143     VTIHFPPDYPFKPPKVAFRT......KVFHPNINS.NGSICLDILK.EQ......WSPAL
lcl|Glyma.06G124900_3-142     VTIHFPPDYPFKPPKVAFRT......KVFHPNINS.NGSICLDILK.EQ......WSPAL
lcl|Glyma.14G124300_3-142     VTIHFPPDYPFKPPKVAFRT......KVFHPNINS.NGSICLDILK.EQ......WSPAL
lcl|Glyma.03G255400_3-142     VTIHFPPDYPFKPPKVAFRT......KVFHPNINS.NGSICLDILK.EQ......WSPAL
lcl|Glyma.19G253000_3-142     VTIHFPPDYPFKPPKVAFRT......KVFHPNINS.NGSICLDILK.EQ......WSPAL
lcl|Glyma.02G236900_3-142     VSIHFPPDYPFKPPKVAFRT......KVFHPNINS.NGSICLDILK.EQ......WSPAL
lcl|Glyma.14G205700_3-142     VSIHFPPDYPFKPPKVAFRT......KVFHPNINS.NGSICLDILK.EQ......WSPAL
lcl|Glyma.08G267400_3-142     VSIHFPPDYPFKPPKVSFCT......KVFHPNINS.NGSICLDILK.EQ......WSPAL
lcl|Glyma.16G103100_4-142     VSIHFPPDYPFKPPKVSFRT......KVFHPNINS.NGSICLDILK.EQ......WSAAL
lcl|Glyma.11G199400_3-142     VSIHFPPDYPFKPPKVAFRT......KVFHPNINS.NGSICLDILK.EQ......WSPAL
lcl|Glyma.18G051400_3-142     VSIHFPPDYPFKPPKVAFRT......KVFHPNINS.NGSICLDILK.EQ......WSPAL
lcl|Glyma.09G273100_3-142     VTIHFPPDYPFKPPKVAFRT......KVFHPNINS.NGSICLDILK.EQ......WSPAL
lcl|Glyma.18G216000_3-152     VTIHFPPDYPFKPPKVAFRT......KVFHPNINS.NGSICLDILK.EQ......WSPAL
lcl|Glyma.11G107800_3-142     VTIHFPPDYPFKPPKVAFRT......KVFHPNINS.NGNICLDILK.EQ......WSPAL
lcl|Glyma.12G032800_3-142     VTIHFPPDYPFKPPKVAFRT......KVFHPNINS.NGNICLDILK.EQ......WSPAL
lcl|Glyma.10G096200_3-142     VTIHFPPDYPFKPPKVSFRT......KVFHPNINS.NGSICLDILK.EQ......WSPAL
lcl|Glyma.08G113700_33-171    LDIKFPIDYPFKPPEVVFKT......RIYHCNVDP.DGRVSMGILK.DD......WSPAL
lcl|Glyma.03G004200_12-151    MKLLLSHDFPHSPPKGFFLT......KIFHPNIANN.GEICVNTLKKD.......WNPSL
lcl|Glyma.19G121700_12-151    MKLLLSHDFPHSPPKGFFLT......KIFHPNIATN.GEICVNTLKKD.......WNPSL
lcl|Glyma.19G072800_12-151    MKLLLSRDFPHSPPKGFFLT......KIFHPNIATN.GEICVNTLKKD.......WNPNL
lcl|Glyma.04G081200_7-148     LAFSVPEQYPLQPPQVRFLT......KIFHPNVHFKTGEICLDILKNA.......WSPAW
lcl|Glyma.06G082900_7-148     LAFSVPEQYPLQPPQVRFLT......KIFHPNVHFKTGEICLDILKNA.......WSPAW
lcl|Glyma.05G035700_7-143     IRVELPDAYPYKSPSIGFVN......KIFHPNVDELSGSVCLDVIN..QS.....WSPMF
lcl|Glyma.17G091700_7-143     IRVELPDAYPYKSPSIGFVN......KIFHPNVDELSGSVCLDVIN..QS.....WSPMF
lcl|Glyma.05G209400_10-143    VRVELPDAYPYKSPSIGFVN......KIFHPNVDEVSGSVCLDVIN..QT.....WSPMF
lcl|Glyma.08G016100_10-143    VRVDLPDAYPYKSPSIGFVN......KIFHPNVDEMSGSVCLDVIS..QT.....WSPMF
lcl|Glyma.07G069300_7-143     VRVELPDAYPYKSPSIGFIN......KIYHPNVDEMSGSVCLDVIN..QT.....WSPMF
lcl|Glyma.16G035000_7-143     VRVELPDAYPYKSPSIGFIN......KIYHPNVDEMSGSVCLDVIN..QT.....WSPMF
lcl|Glyma.09G257800_18-145    VRVELPDAYPYKSPSIGFIN......KIYHPNVDEMSGSVCLDVIN..QT.....WSPMF
lcl|Glyma.18G234800_7-143     VRVELPDAYPYKSPSIGFIN......KIYHPNVDEMSGSVCLDVIN..QT.....WSPMF
lcl|Glyma.05G140200_172-318   FDCLFPSTYPAGPPKVHYHSG....GLRLNPNLYQ.CGKVCLSLLGTWHGRNSENWIPEK
lcl|Glyma.08G095600_194-340   FDCLFPSTYPAGPPKVHYHSG....GLRLNPNLYN.CGKVCLSLLGTWHGSNSENWIPEK
lcl|Glyma.12G153900_92-241    FDVFFSSGYPHVPPQVHYHSG....GLRLNPNLYS.SGKVCLSLLNTWTGHQNEQWIPGV
lcl|Glyma.12G154000_8-156     FDVFFPSGYSHVPPQVHYHSG....GLRLNPNLYS.SGKVCLSLLNTWTGHQNEQWIPGV
lcl|Glyma.06G243300_493-619   FDVYFPSGYPHVPPNVHYHSG....GLRLNPNLYN.CGKVCLSLLNTWTGHQNEQWISGV
lcl|Glyma.12G224200_443-569   FDVFFPSAYPNVPPKVHYHSG....GLRLNPNLYA.CGKVCLSLLNTWSGSKNEKWLPGV
lcl|Glyma.13G277400_462-588   FDVFFPSAYPNVPPKVHYHSG....GLRLNPNLYA.CGKVCLSLLNTWSGSKNEKWVPGM
lcl|Glyma.13G239100_672-825   FDICFPPEYPSEPPMVHYNSG....GLRLNPNLYE.SGKVCLSLLNTWTGTDTEVWNPGA
lcl|Glyma.15G074200_677-830   FDICFPPEYPSEPPMVHYNSG....GLQLNPNLYE.SGKVCLSLLNTWTGTDTEVWNPGA
lcl|Glyma.13G179600_679-832   FDICFPPEYPNEPPMVHYNSA....GLRLNPNLYE.SGKICLSLLNTWTGTDTEVWNPGA
lcl|Glyma.07G196500_680-833   FDICFPPEYPNKPPMVHYNSG....GLRLNPNLYE.SGKICLSLLNTWTGTGTEVWNPGA
lcl|Glyma.10G282000_880-1030  FDFHLPPEYPDVPPSAYYHSG....GWRINPNLYE.EGKVCLSLLNTWTGRGNEVWDPKS
lcl|Glyma.20G107300_876-1026  FDFHLPPEYPDVPPSAYYHSG....GWRINPNLYE.EGKVCLSLLNTWTGRGNEVWDPKS
lcl|Glyma.07G239100_38-188    FDIGFPSDYPKNPPKLHYHSF....GYRHNPNLYS.SGRVCLSLLNTWTGRKSEKWDPSG
lcl|Glyma.17G034200_47-149    FDILFPSDYPKHPPKLHFDSF....GLQVNPNLHP.SGEVCLSLLNTWYGKKREKWDPS.
lcl|Glyma.17G034300_1-145     FDIMFPSDYPKHPPMLHFHSF....GYRINPNLYI.NGKVCLSLLNTWNGKGTEMWDPSK
lcl|Glyma.17G034400_40-142    FDIAFPHDYPFHPPEVHYRSY....GFSLNPNLYN.NGHVCLSLINTWVGKSTEKWDP..
lcl|Glyma.07G046200_7-157     GKIKFPPEYPYKPPGISMTTP....NGRFMT.....QKKICLSMSDFHPES....WNPMW
lcl|Glyma.16G014400_7-157     GKIKFPPEYPYKPPGISMTTP....NGRFMT.....QKKICLSMSDFHPES....WNPMW
lcl|Glyma.07G240600_12-127    GRIQLPSEYPFKPPSFMLLTP....SGRFET.....QTKICLSISNHHPEH....WQPSW
lcl|Glyma.17G032800_12-151    GRIQLPSEYPFKPPSFMLLTP....SGRFET.....QTKICLSISNHHPEH....WQPSW
lcl|Glyma.15G141200_12-127    GRIQLPSEYPFKPPSFMLLTP....NGRFET.....QTKICLSISNHHPEH....WQPSW
lcl|Glyma.09G036300_12-127    GRIQLPSEYPFKPPSFMLLTP....NGRFET.....QTKICLSISNHHPEH....WQPSW
lcl|Glyma.11G099100_34-153    VVIWLMESYPRHPPCVYVNPTRDMIIKRPHPHVNP.SGLVSVPYLQN........WTYPS
```

```
                                     α2                          α3               α4
UBE2D2                                                   TT
                                   100          110          120          130

UBE2D2                        T.ISKVLLSIC.SLLCDPNPDDPL.VPEIARIYKTDREKYNRIAREW.........
consensus                     T.LETVLLSLQ.SLLSEPNPDSPL.NADAAELYKKNREEFKKKAREWTKKYAE...
lcl|Glyma.04G089600_33-128    R.GGKICLTVHFKPLW........................................
lcl|Glyma.06G091400_33-128    R.GGKICLTVHFKPLW........................................
lcl|Glyma.02G199700_17-143    T.MEDILTQLKKEMAAPHNRKLVQPPEV............................
lcl|Glyma.10G077600_17-143    T.MEDILTQLKKEMAAPHNRKLVQPPEG............................
lcl|Glyma.03G199900_17-146    T.MEDILTQLKKEMAASHNRKLVQPPEGTHY.........................
lcl|Glyma.19G197500_17-154    T.MEDILTQLKKEMAASHNRKLVQPPEVGAYVMFLNVNV.................
lcl|Glyma.03G113100_5-108     T.MEDILT................................................
lcl|Glyma.15G189500_8-136     T.MEDILTQLKKEMAAPHNRKLVQPPEVSH..........................
lcl|Glyma.05G235000_15-150    T.MEDILMQLKKEMISPQNRKLAQPPEGNEDGRIDQK...................
lcl|Glyma.08G042500_15-150    T.MEDILMQLKKEMMSPQNRKLAQPPEGNEDGRIDQK...................
lcl|Glyma.07G168100_15-150    T.MEDILIQLKREMMSPQNRKLTQPPEGNEESRVDQK...................
lcl|Glyma.09G124800_15-150    T.MEDVLIQLKREMMSPQNRKLTQPPEGNEEARVDQK...................
lcl|Glyma.01G178200_33-167    N.INTVIYGLY.HLFTEPNYEDPL.NHDAAAVLRENPKMFESNVRR..........
lcl|Glyma.11G064000_33-167    N.INTVIYGLY.HLFTEPNYEDPL.NHDAAAVLRENPKMFESNVRR..........
lcl|Glyma.01G043600_7-145     D.VAAILTSIQ.SLLCDPNPNSPA.NSEAARIFSENKREYNRRVRDIV........
lcl|Glyma.02G020000_7-145     D.VAAILTSIQ.SLLCDPNPNSPA.NSEAARMFSENKREYNRRVRDIV........
lcl|Glyma.04G173300_7-145     D.VAAILTSIQ.SLLCDPNPNSPA.NSEAARMFSENKREYNRRVREIV........
lcl|Glyma.05G028800_7-145     D.VAAILTSIQ.SLLCDPNPNSPA.NSEAARMFSENKREYNRRVREIV........
lcl|Glyma.17G098000_7-145     D.VAAILTSIQ.SLLCDPNPNSPA.NSEAARMFSENKREYNRRVREIV........
lcl|Glyma.08G297300_7-145     D.VAAILTSIQ.SLLCDPNPNSPA.NSEAARMFSENKREYNRKVREIV........
lcl|Glyma.18G124600_7-111     D.VAAILTSIQ.V...........................................
lcl|Glyma.10G212200_39-178    D.CRTILLSIQ.SLLEEPNLESPL.NSYAAALWN.DKEDYRRMVHKQY........
lcl|Glyma.20G178900_39-174    D.CRTILLSIQ.SLLEEPNLESPL.NSYAAALWN.DKEDYRRMV............
lcl|Glyma.11G095700_39-155    D.VRTILLSIQ.SLLGGTSLEKAL.N..............................
lcl|Glyma.12G021800_39-176    D.VRTILLSIQ.SLLGEPNISSPL.NQQAAQLWS.NQEEYRKMVEKLY........
lcl|Glyma.05G091100_9-153     T.VKQILVGIQ.DLLDQPNPADPA.QTEGYHLFIQDAAEYKRRVRQQA........
lcl|Glyma.17G169700_9-153     T.VKQILVGIQ.DLLDQPNPADPA.QTEGYHLFIQDAAEYKRRVRQQA........
lcl|Glyma.11G053300_8-150     T.VKQILVGIQ.DLLDQPNPADPA.QTEGYHLFIQDAAEYKRRVRQ..........
lcl|Glyma.01G188900_8-150     T.VKQILVGIQ.DLLDQPNPADPA.QTEGYHLFIQDAAEYKRRVRQ..........
lcl|Glyma.01G169700_17-117    T.VSSIC.................................................
lcl|Glyma.04G199200_6-145     T.LKTALLSVQ.ALLSAPQPDDPQ.DAVVAQQYLKDYQTFVNTARYWT........
lcl|Glyma.06G166300_6-145     T.LKTALLSVQ.ALLSAPQPDDPQ.DAVVAQQYLKDYQTFVNTARYWT........
lcl|Glyma.06G230300_7-146     Q.IRTVLLSIQ.ALLSAPNPDDPL.SENIAKHWKSNEAEAVETAKEWT........
lcl|Glyma.12G161200_7-146     Q.IRTVLLSIQ.ALLSAPNPDDPL.SENIAKHWKSNEAEAVETAKEWT........
lcl|Glyma.13G270800_7-146     Q.IRTVLLSIQ.ALLSAPNPDDPL.SENIAKHWKSNEAEAVETAKEWT........
lcl|Glyma.12G228900_7-108     G.IRTVLL................................................
lcl|Glyma.04G238800_3-142     T.ISKVLLSIC.SLLTDPNPDDPL.VPEIAHMYKTDRNKYESTARSWT........
lcl|Glyma.13G040500_3-143     T.ISKVLLSIC.SLLTDPNPDDPL.VPEIAHMYKTDRNKYESNARSWT........
lcl|Glyma.06G124900_3-142     T.ISKVLLSIC.SLLTDPNPDDPL.VPEIAHMYKTDRNKYESTARSWT........
lcl|Glyma.14G124300_3-142     T.ISKVLLSIC.SLLTDPNPDDPL.VPEIAHMYKTDRNKYESTARSWT........
lcl|Glyma.03G255400_3-142     T.ISKVLLSIC.SLLTDPNPDDPL.VPEIAHMYKTDRSKYETTARSWT........
lcl|Glyma.19G253000_3-142     T.ISKVLLSIC.SLLTDPNPDDPL.VPEIAHMYKTDRSKYETTARSWT........
lcl|Glyma.02G236900_3-142     T.ISKVLLSIC.SLLTDPNPDDPL.VPEIAHMYKTDRAKYEATARSWT........
lcl|Glyma.14G205700_3-142     T.ISKVLLSIC.SLLTDPNPDDPL.VPEIAHMYKTDRAKYEATARSWT........
lcl|Glyma.08G267400_3-142     T.ISKVLLSIC.SLLTDPNPDDPL.VPEIAHMYKTDRAKYEATARSWT........
lcl|Glyma.16G103100_4-142     T.ISKVLLSIC.SLLTDPNPDDPL.VPEIAQMYKTNRTKYEATARSWT........
lcl|Glyma.11G199400_3-142     T.ISKVLLSIC.SLLTDPNPDDPL.VPEIAHMYKTDRAKYEATARSWT........
lcl|Glyma.18G051400_3-142     T.ISKVLLSIC.SLLTDPNPDDPL.VPEIAHMYKADKAKYEATARSWT........
lcl|Glyma.09G273100_3-142     T.ISKVLLSIC.SLLTDPNPDDPL.VPEIAHMYKTDRAKYEATARSWT........
lcl|Glyma.18G216000_3-152     T.ISKVLLSIC.SLLTDPNPDDPL.VPEIAHMYKTDRAKYEATARSWT........
lcl|Glyma.11G107800_3-142     T.ISKVLLSIC.SLLTDPNPDDPL.VPEIAHLCKTDKFKYESTARSWT........
lcl|Glyma.12G032800_3-142     T.ISKVLLSIC.SLLTDPNPDDPL.VPEIAHMCKTDKVKYESTARSWT........
lcl|Glyma.10G096200_3-142     T.VSKVLLSIC.SLLTDPNPDDPL.VPDIAHMYKTDRDKYESTARSWT........
lcl|Glyma.08G113700_33-171    T.ITKVLLEVR.SIMTNPDPYNAV.VPGIAHLYSGDRAKHDDIAAEWT........
lcl|Glyma.03G004200_12-151    G.LRHVLIVVR.CLLIEPFPESALNEQAGKMLLEN.YEEYARHARLYT........
lcl|Glyma.19G121700_12-151    G.LRHVLIVVR.CLLIEPFPESALNEQAGKMLLEN.YEEYARHARLYT........
lcl|Glyma.19G072800_12-151    G.LRHVLIVVR.CLLIEPFPESALNEQAGKLLLEN.YEEYARHARLYT........
lcl|Glyma.04G081200_7-148     T.LQSVCRAII.ALMAHPEPDSPLNCDSGNLLRSGDVRGYQSMARMYT........
lcl|Glyma.06G082900_7-148     T.LQSVCRAII.ALMAHPEPDSPLNCDSGNLLRSGDVRGYQSMARMYT........
lcl|Glyma.05G035700_7-143     D.LLNVFEAFLPQLLLYPNASDPL.NGDAASLMMKDKELYDQKVKEYC........
lcl|Glyma.17G091700_7-143     D.LLNVFEVFLPQLLLYPNASDPL.NGDAASLMMKDKKLYDQKVKEYC........
lcl|Glyma.05G209400_10-143    D.LVNVFEVFLPQLLLYPNASDPL.NGDAAALMIRDHATYEQRVKEYC........
lcl|Glyma.08G016100_10-143    D.LVNVFEVFLPQLLLYPNASDPL.NGDAAALMIRDHATYEQRVKEYC........
lcl|Glyma.07G069300_7-143     D.LVNVFEVFLPQLLLYPNPSDPL.NGEAAALMMRDRATYEQRVKEYC........
lcl|Glyma.16G035000_7-143     D.LVNVFEVFLPQLLLYPNPSDPL.NGEAAALMMRDRATYEQRVKEYC........
lcl|Glyma.09G257800_18-145    D.LVNVFEVFLPQLLLYPNPSDPL.NGEAAALMMRDRPSYEQRVKEYC........
lcl|Glyma.18G234800_7-143     D.LVNVFEVFLPQLLLYPNPSDPL.NGEAAALMMRDRPSYEQRVKEYC........
lcl|Glyma.05G140200_172-318   STMLQVLVSIQALILNEKPFFNEPGYPSTYPGSEGQRRSKEYNE............
lcl|Glyma.08G095600_194-340   STMLQVLVSIQALILNEKPFFNEPGYTSTYPGAEGQRRSKEYNE............
lcl|Glyma.12G153900_92-241    STILQVLVSIQGLILVAKPFFNEPEYAHLSNSQYGEISSLKYNEDTF.........
lcl|Glyma.12G154000_8-156     STILQVLVFIQGLILVAKPFFNEPGYAHLSNSQYGEISSLKYNEDTF.........
lcl|Glyma.06G243300_493-619   STILQVLVSIQGLILVAKPFFNEP................................
lcl|Glyma.12G224200_443-569   STILQVLVSIQGLILNTKPYFNEP................................
lcl|Glyma.13G277400_462-588   STILQVLVSLQGLILNTKPYFNEP................................
lcl|Glyma.13G239100_672-825   STILQVLLSLQALVLNDKPYFNEAGYDQQIGRAEGERNSVSYNENAFLVT......
lcl|Glyma.15G074200_677-830   STVLQVLLSLQALVLNEKPYFNEAGYDQQIGRAEGERNSVSYNENASLVT......
lcl|Glyma.13G177400_679-832   STILQVLLSLQALVLNEKPYFNEAGYDQQIGRAEGEKNSVSYNENAFLVT......
lcl|Glyma.07G196500_680-833   STILQVLLSLQALVLNEKPYFNEAGYDQQIGRAEGEKNSVSYNENALLVT......
lcl|Glyma.10G282000_880-1030  SSILQVLVSLQGLVLNSKPYFNEAGYDKQVGTAEGEKNSLSYNENTFL........
lcl|Glyma.20G107300_876-1026  SSILQVLVSLQGLVLNSKPYFNEAGYDKQVGTAEGEKNSLSYNENTFL........
lcl|Glyma.07G239100_38-188    STMLQVLLSIQALVLNKKPYYNEPGLASIASS...EWRSRAYNENVFLIT......
lcl|Glyma.17G034200_47-149    ........................................................
lcl|Glyma.17G034300_1-145     STMLQVLVSIQGLVLNKNPYFNEP..LRLFDS...ESKSRVYNENAFIIT......
lcl|Glyma.17G034400_40-142    ........................................................
lcl|Glyma.07G046200_7-157     S.VSSILTGLLSFMMDNSPTTGSVNTTTAEKQRLAKSSLSFNCKNATFRKMFPEYV
lcl|Glyma.16G014400_7-157     S.VSSILTGLLSFMMDNSPTTGSVNTTTAEKQRLAKSSLSFNCKNATFRKIFPEYV
lcl|Glyma.07G240600_12-127    S.VRTALVALIAFMPTNP..NGA.................................
lcl|Glyma.17G032800_12-151    S.VRTALVALIAFMPTNP..NGALGSLNY..KKEDRRALAIKSREAPPK.......
lcl|Glyma.15G141200_12-127    S.VRTALVALIAFMPTNP..NGA.................................
lcl|Glyma.09G036300_12-127    S.VRTALVALIAFMPTNP..NGA.................................
lcl|Glyma.11G099100_34-153    SNLVDLLILNLSLHFGRDP.....................................
                                          1
```
