# Supplementary figures and images for: Genome-wide analysis of genes encoding core components of the ubiquitin system in soybean (Glycine max) reveals a potential role for ubiquitination in host immunity against soybean cyst nematode
Source: BMC Plant Biol. 2018 Jul 18;18:149. doi: 10.1186/s12870-018-1365-7 (PMC6052599; doi:10.1186/s12870-018-1365-7)

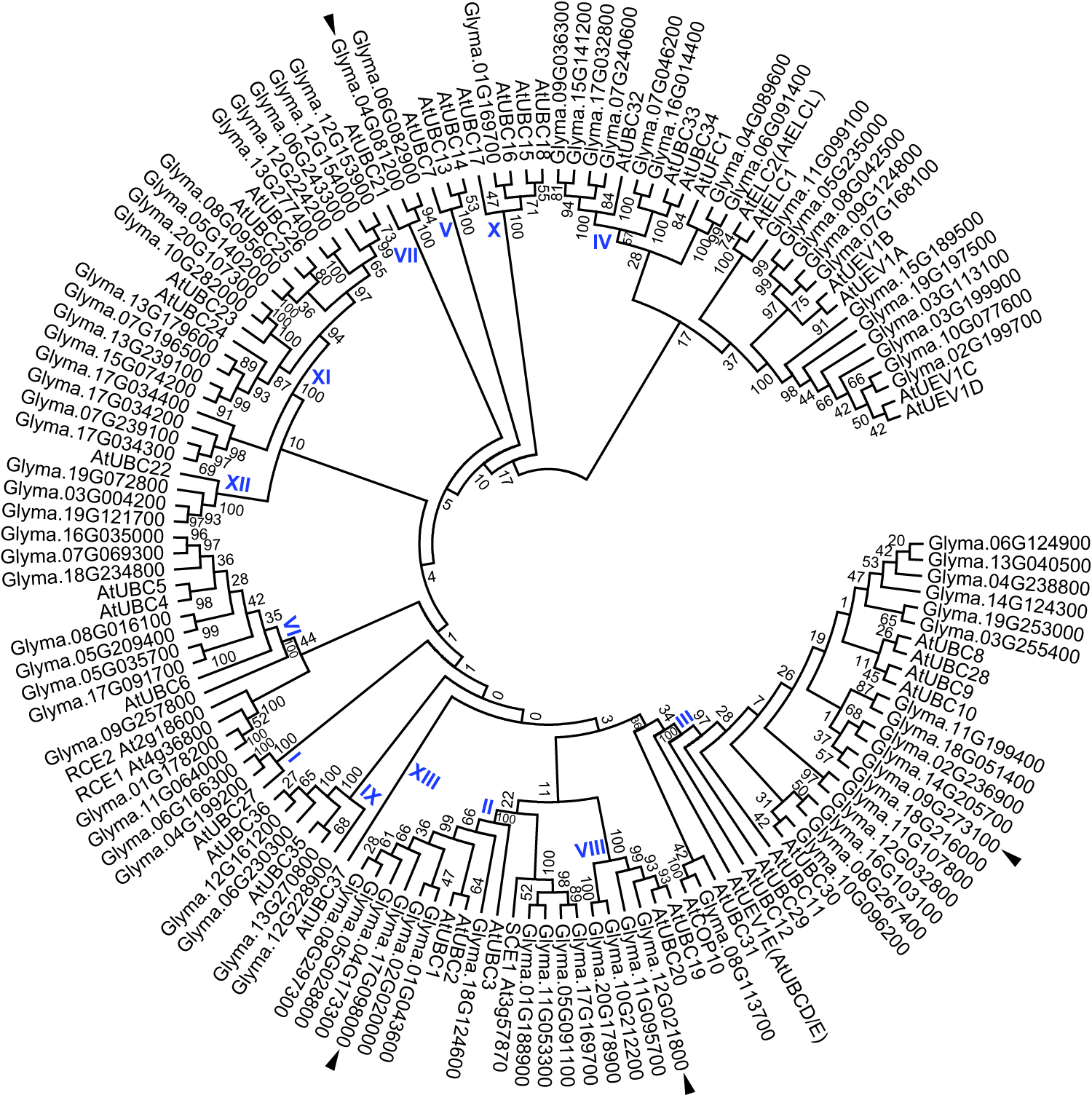

Supplement: Supplementary file 5 — Figure S2. Phylogenetic tree of the Arabidopsis and soybean UBC domain-containing proteins. (JPG 1759 kb) [file 12870_2018_1365_MOESM5_ESM.jpg]

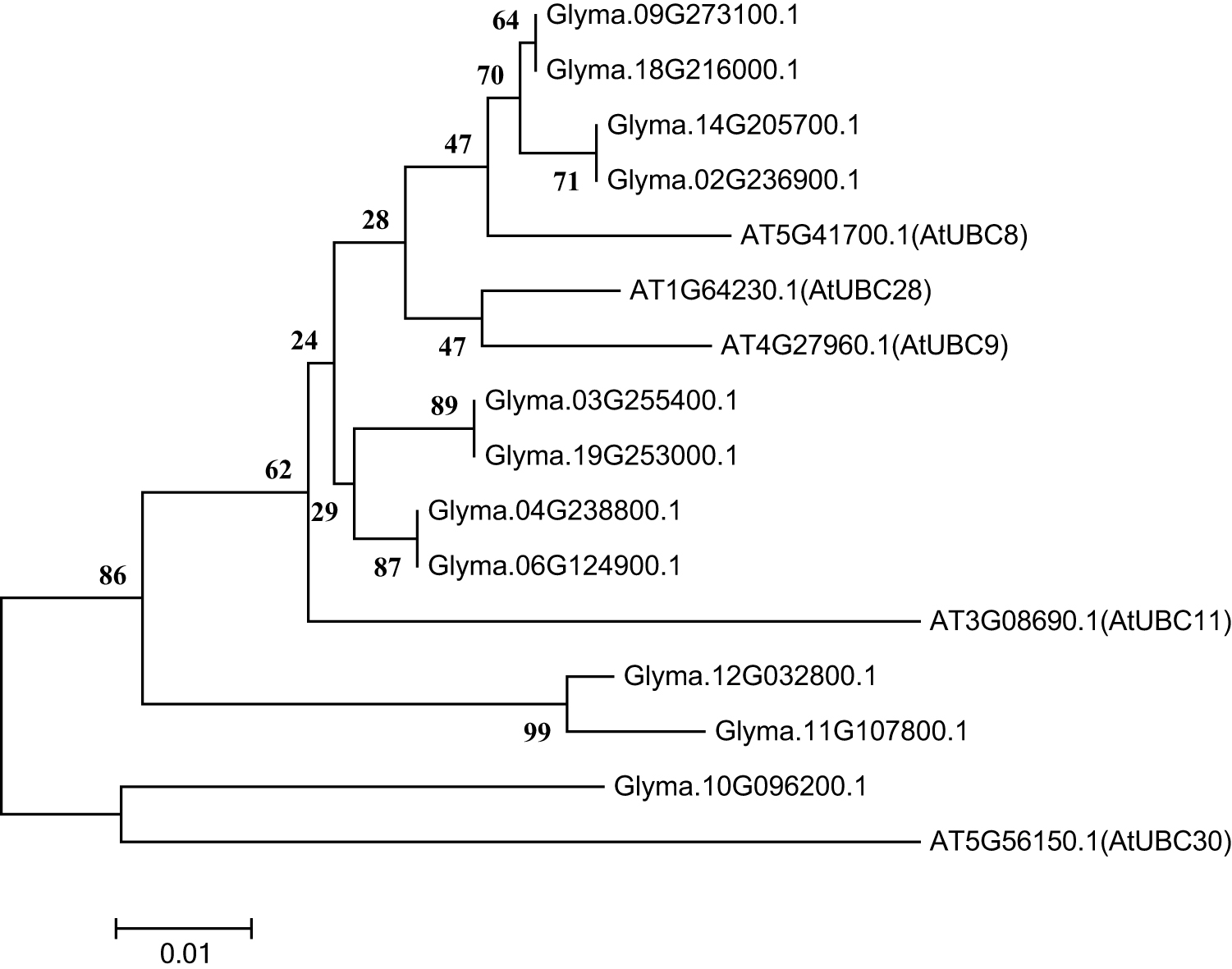

Supplement: Supplementary file 6 — Figure S3. Phylogenetic tree of the soybean ubiquitin E2 proteins. (JPG 905 kb) [file 12870_2018_1365_MOESM6_ESM.jpg]

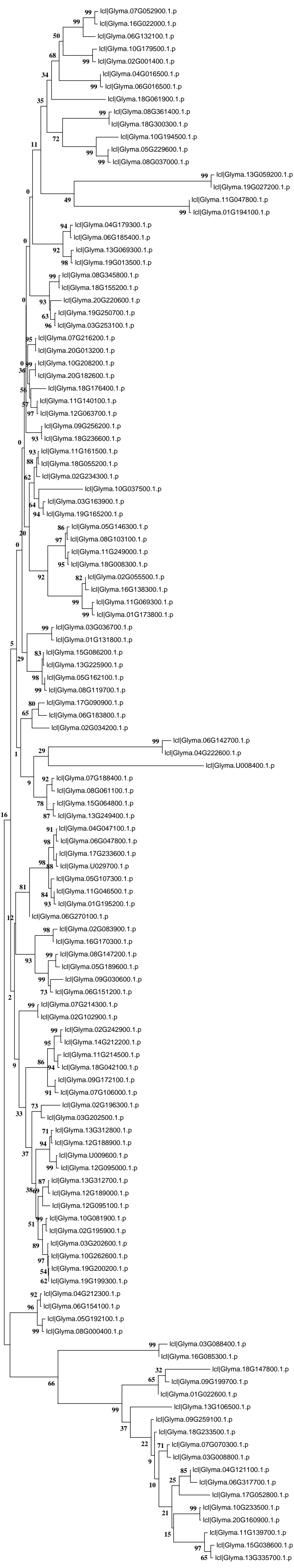

Supplement: Supplementary file 11 — Figure S8. Phylogenetic analysis of soybean U-box domain-containing proteins. (PDF 31 kb) [file 12870_2018_1365_MOESM11_ESM.pdf]

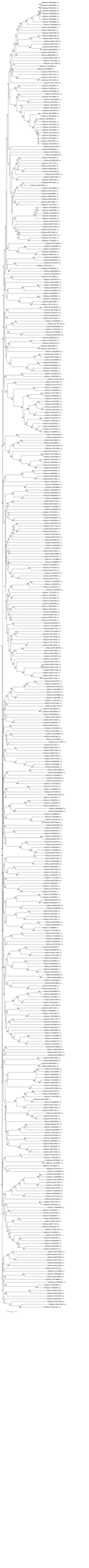

Supplement: Supplementary file 12 — Figure S9. Phylogenetic analysis of soybean F-box domain-containing proteins. (PDF 118 kb) [file 12870_2018_1365_MOESM12_ESM.pdf]

## Slide 1
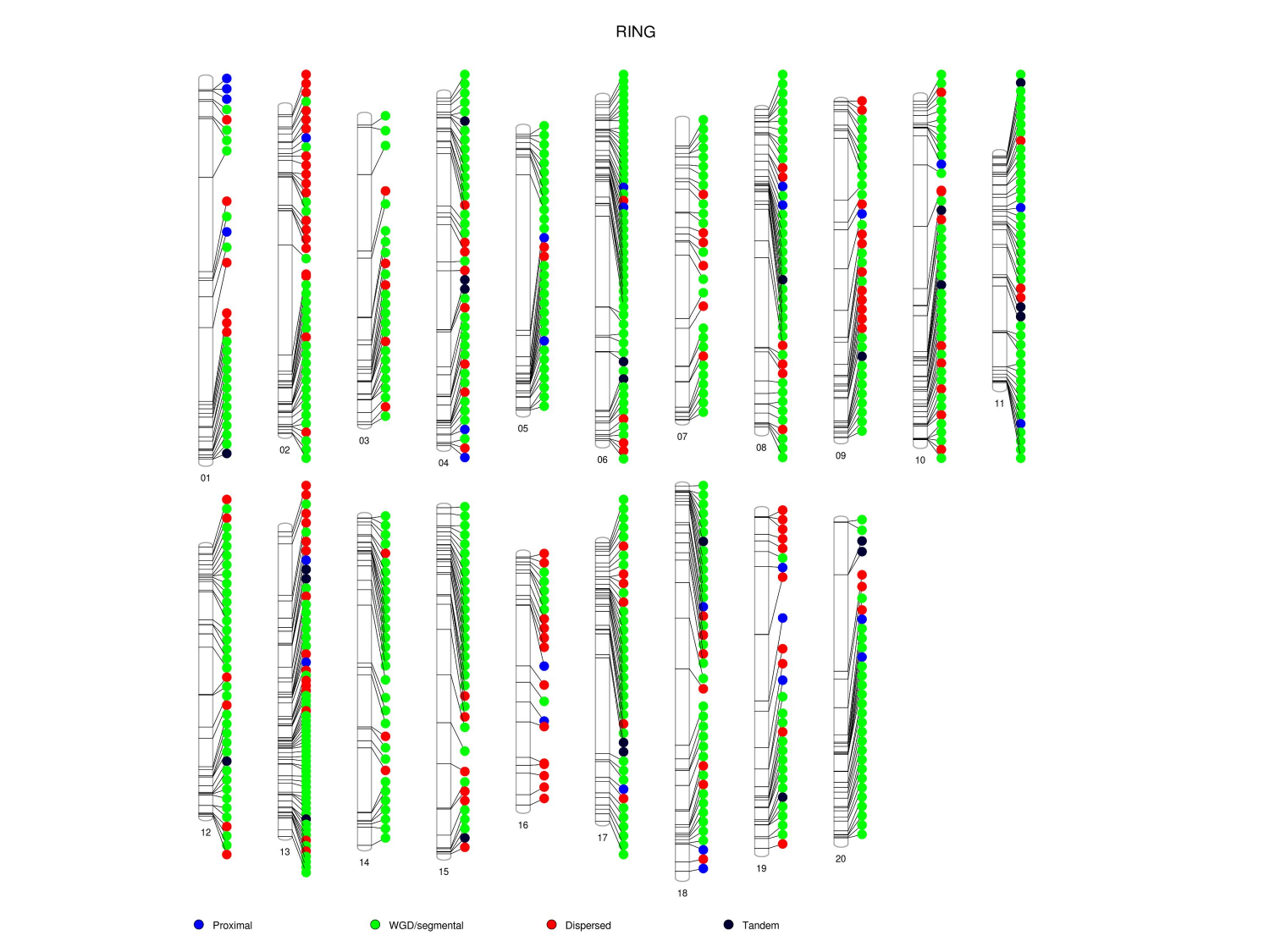

Supplement: Supplementary file 13 — Figure S10. Duplication events of soybean RING genes. (PPTX 1668 kb) [file 12870_2018_1365_MOESM13_ESM.pptx]

## Slide 1
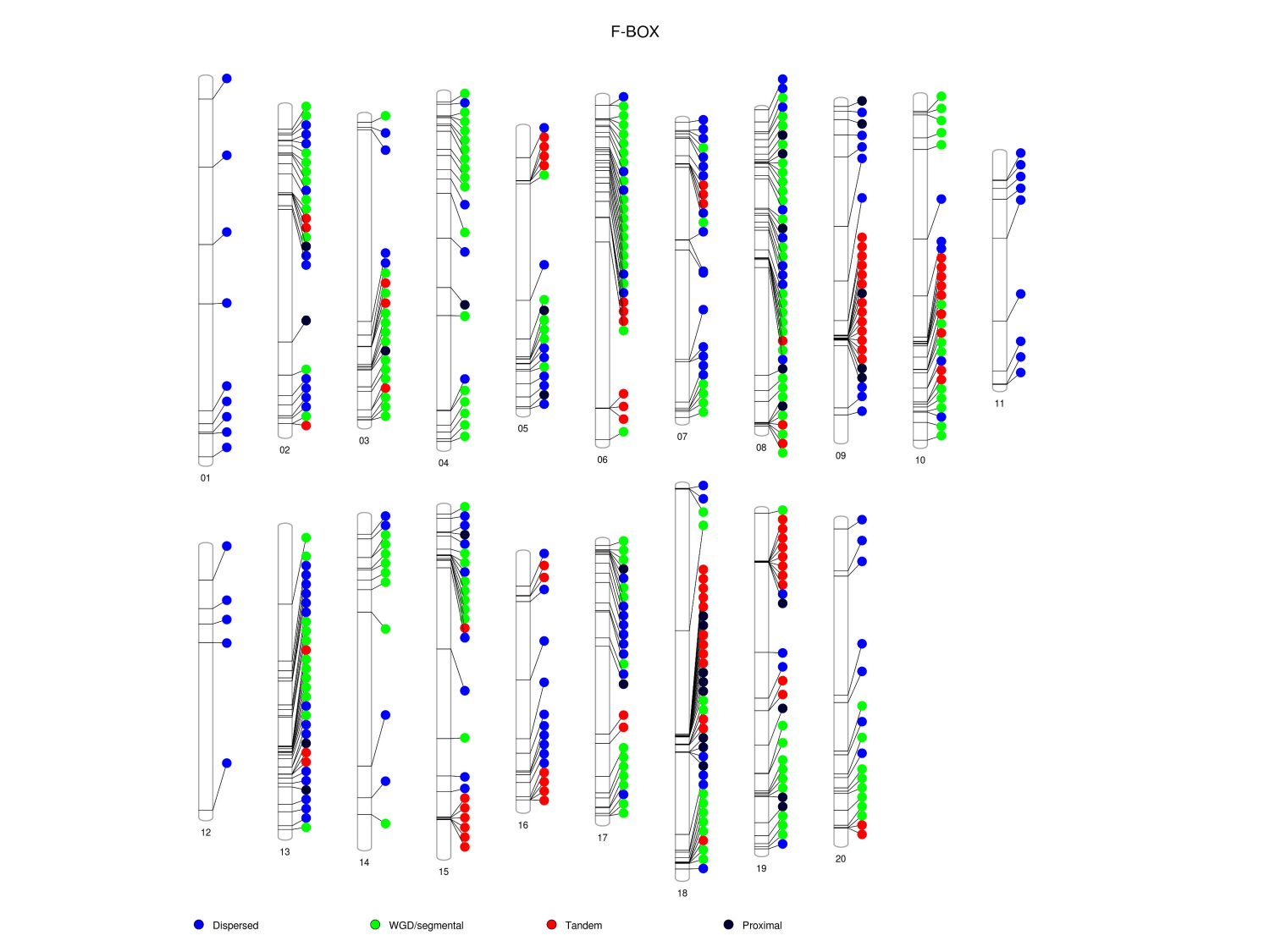

Supplement: Supplementary file 14 — Figure S11. Duplication events of soybean F-box genes. (PPTX 1064 kb) [file 12870_2018_1365_MOESM14_ESM.pptx]

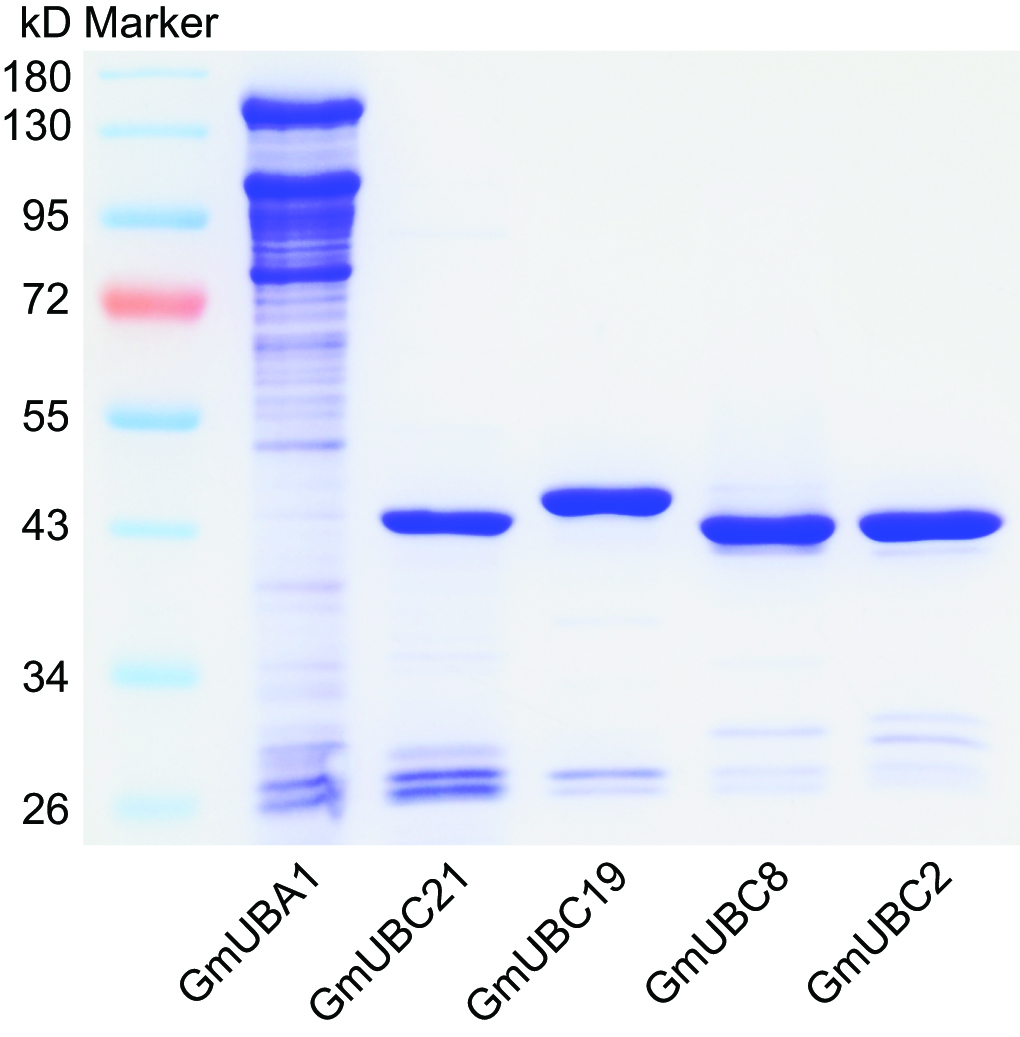

Supplement: Supplementary file 16 — Figure S12. Examination of purified soybean E1 and E2 proteins using SDS-PAGE. (JPG 992 kb) [file 12870_2018_1365_MOESM16_ESM.jpg]

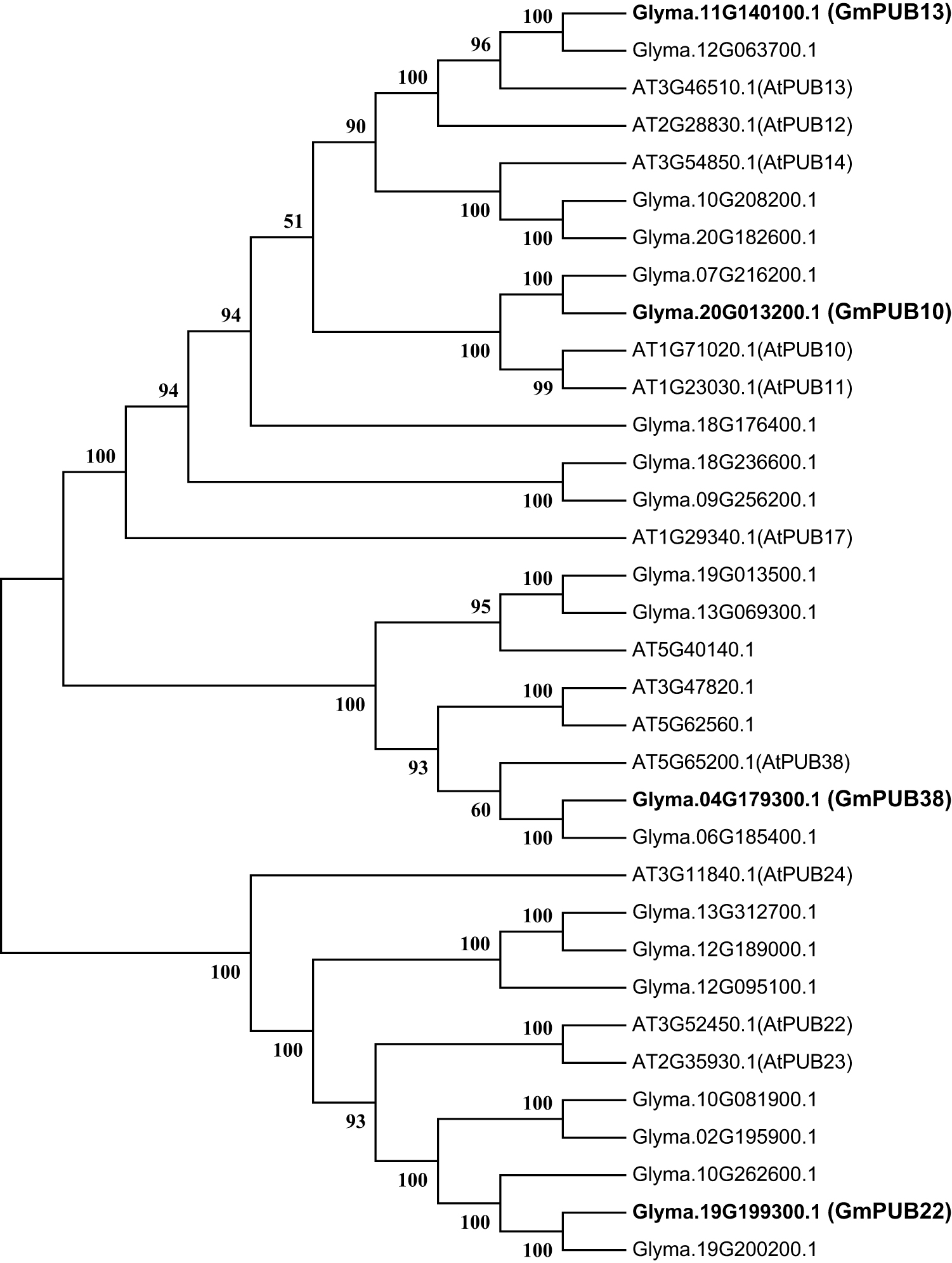

Supplement: Supplementary file 17 — Figure S13. Phylogenetic analysis of cloned soybean U-box domain-containing proteins and their homologs in Arabidopsis. (JPG 1220 kb) [file 12870_2018_1365_MOESM17_ESM.jpg]

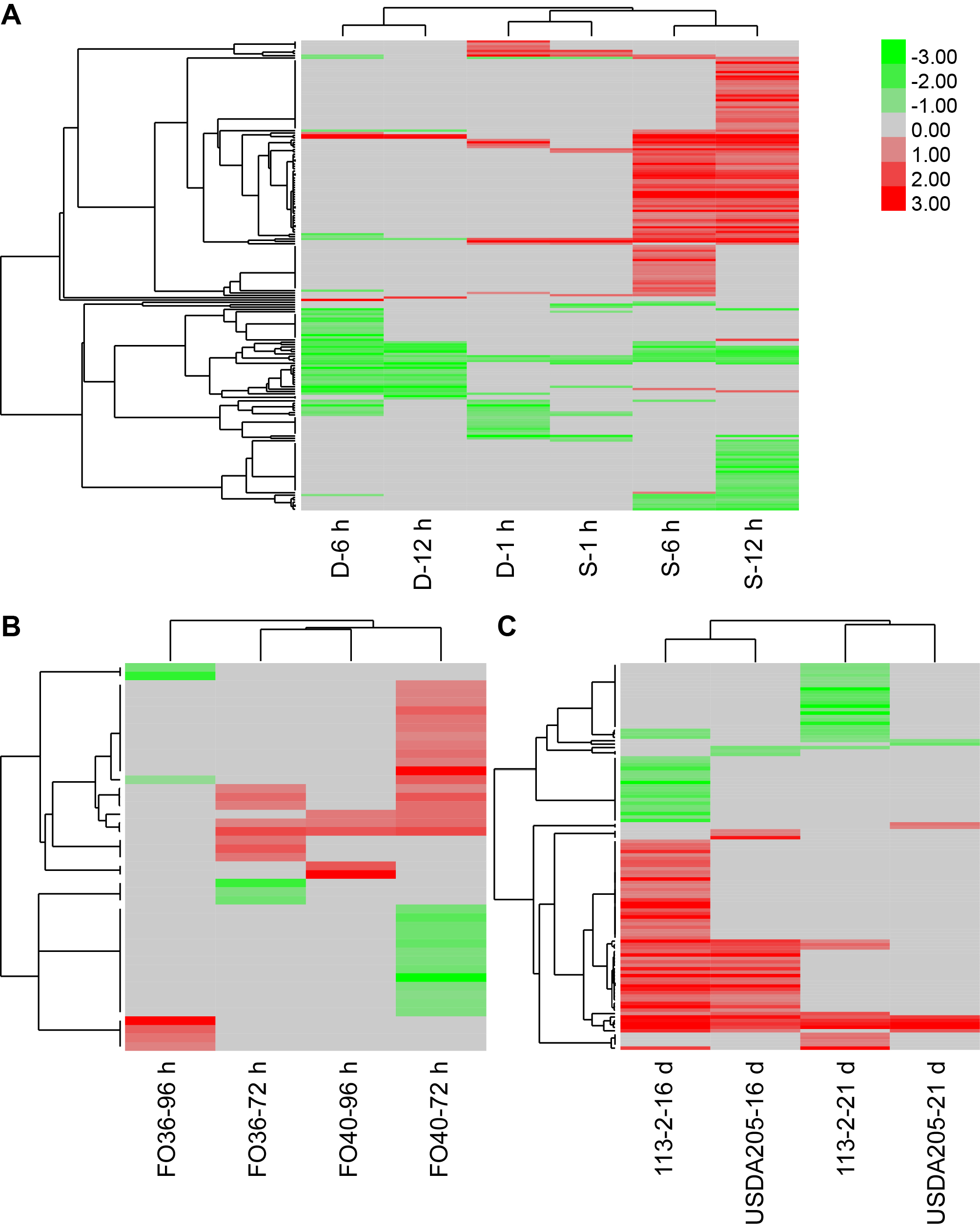

Supplement: Supplementary file 19 — Figure S14. Heatmap of differentially expressed soybean UBS genes after abiotic and biotic stress treatment. (JPG 1112 kb) [file 12870_2018_1365_MOESM19_ESM.jpg]

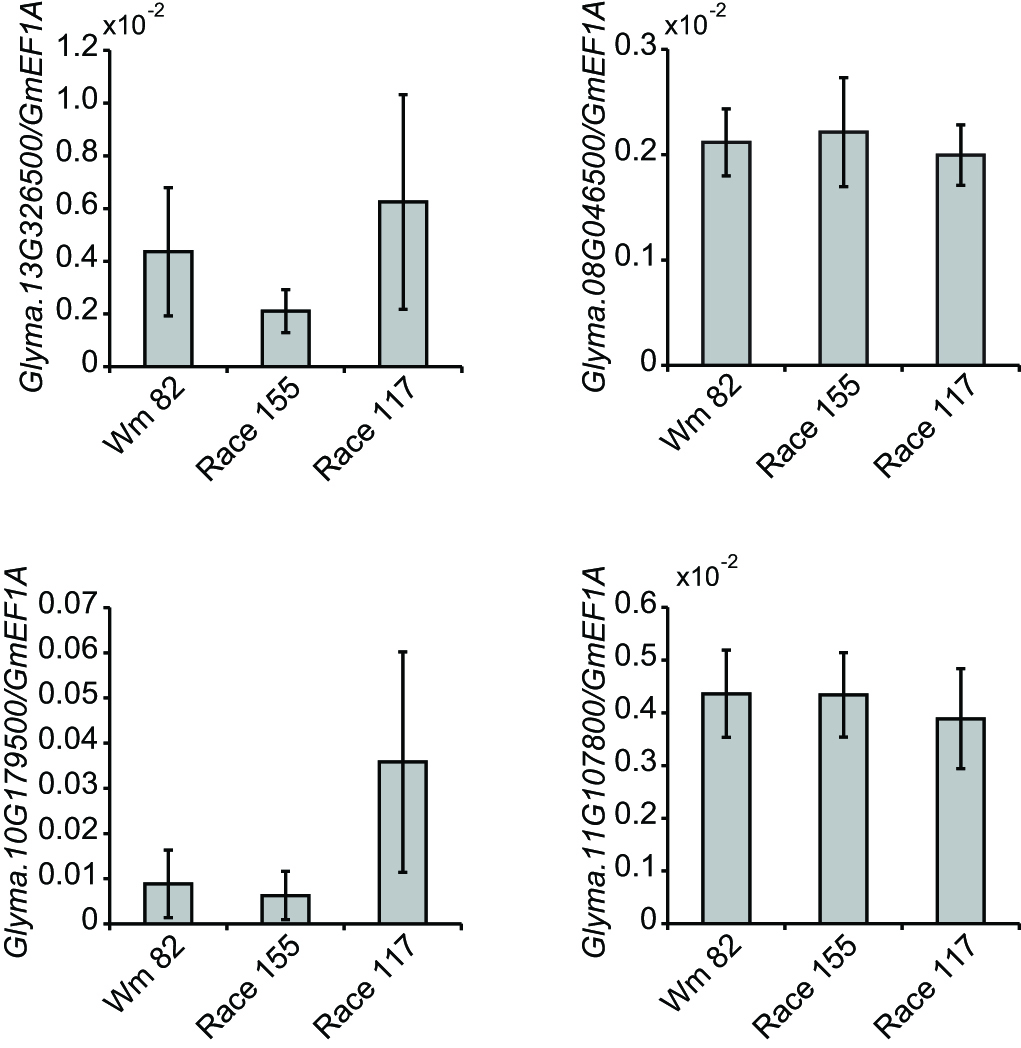

Supplement: Supplementary file 22 — Figure S15. The expression level of selected soybean UBS genes identified in the RNA-seq analysis are not changed after SCN treatment. (JPG 1032 kb) [file 12870_2018_1365_MOESM22_ESM.jpg]
