# Supplementary material for: Genome-wide analysis of genes encoding core components of the ubiquitin system in soybean (Glycine max) reveals a potential role for ubiquitination in host immunity against soybean cyst nematode
Source: BMC Plant Biol. 2018 Jul 18;18:149. doi: 10.1186/s12870-018-1365-7 (PMC6052599; doi:10.1186/s12870-018-1365-7)
Supplement: Supplementary file 7 — Figure S4. Alignment of sequences of the RING domain from soybean RING domain-containing proteins. (PDF 88 kb) [file 12870_2018_1365_MOESM7_ESM.pdf]

```
                                                                     β7
Human_c-Cbl                                              TTT            →                    -
                                                        1                  10
Human_c-Cbl                        ..........CKICAENDK..........DVKI....................EPCGHL
consensus                          ..........CPICLEEYL..........KDPV...................ILPC.GH
lcl|Glyma.09G103000_21-70          ...........QICG..............DNIGNNA.........NGDPFIACDVCAFP
lcl|Glyma.15G275000_20-69          ...........QICG..............DNIGNNV.........NGDPFIACDVCAFP
lcl|Glyma.04G067900_40-89          ...........QICG..............DTVGLTA.........TGDVFVACNECAFP
lcl|Glyma.06G069600_30-98          .PLKSLNGQICQICG..............DTVGLTA.........TGDVFVACNECAFP
lcl|Glyma.13G202500_20-69          ...........QICS..............DGVGKTV.........DGEPFVACDVCAFP
lcl|Glyma.02G080900_38-87          ...........QICG..............DELEVTV.........NGEPFVACNECAFP
lcl|Glyma.16G165900_30-96          ...TELSGQICQICG..............DELEVTV.........NGEPFVACNECAFP
lcl|Glyma.05G187300_39-88          ...........QICG..............DEIEITV.........DGEPFVACNECAFP
lcl|Glyma.08G145600_39-88          ...........QICG..............DEIEITV.........DGEPFVACNECAFP
lcl|Glyma.10G223500_41-90          ...........QICG..............DEIEVTV.........DGEPFVACNECAFP
lcl|Glyma.02G205800_37-86          ...........EICG..............DDVGLTV.........DGDLFVACNECGFP
lcl|Glyma.17G072200_27-95          .PLKNLDGQVCEICG..............DDVGLTV.........DGDLFVACNECGFP
lcl|Glyma.04G153700_37-86          ...........EICG..............DGVGLTV.........DGDLFVACNECGFP
lcl|Glyma.06G225500_37-86          ...........EICG..............DGVGLTV.........DGDLFVACNECGFP
lcl|Glyma.04G142700_37-86          ...........EICG..............DSVGLTV.........DGDLFVACEECGFP
lcl|Glyma.06G225400_46-117         KPVKNLDGQLCEICG..............DSVGLTV.........DGDLFVACEECGFP
lcl|Glyma.06G316800_40-89          ...........QLCG..............DDIGVNE.........DGDLFVACNECAFP
lcl|Glyma.09G051100_38-87          ...........RVCG..............DEIGYKE.........NGELFVACHVCGFP
lcl|Glyma.15G157100_38-87          ...........RVCG..............DEIGYME.........NGELFVACHVCRFP
lcl|Glyma.08G088400_32-81          ...........RVCS..............DEIGYGE.........DGKLFVACHVCRFP
lcl|Glyma.08G302500_39-88          ...........QICG..............DTIGLTA.........TGDVFVACHECGFP
lcl|Glyma.18G117100_39-88          ...........QICG..............DTIGLTA.........TGDLFVACHECGFP
lcl|Glyma.04G063800_9-58           ...........NSCG..............EQIGLDA.........NGELFVACHECYFP
lcl|Glyma.06G065000_6-58           ........HFCNSCG..............EQIGLDA.........NGEVFVACHECYFP
lcl|Glyma.05G160000_385-436        ........PLCNTCG..............EQVGLNA.........NGEVFVACHECNFP
lcl|Glyma.08G117500_6-58           ........PLCNTCG..............EQVGLND.........NGEVFVACHECNFP
lcl|Glyma.12G017600_131-178        ............GCD..............CKVMSDE.........HGEDILPC.ECDFK
lcl|Glyma.02G090900_13-60          ..........CRICHDE............DEES.............NMDTPC..SCCGT
lcl|Glyma.18G292200_14-61          ..........CRICHDE............DEES.............NMDTPC..SCCGT
lcl|Glyma.18G242400_64-112         ..........CRYCQEE............DFIF.............NMESPC..NCNGS
lcl|Glyma.02G103800_77-125         ..........CRICRNPG...........DAEN.............PLRYPC..ACSGS
lcl|Glyma.07G215200_80-128         ..........CRICRNPG...........DAEN.............PLRYPC..ACSGS
lcl|Glyma.13G372600_71-120         ..........CRICLDI............GGE..............DLIAPC..HCKGT
lcl|Glyma.15G001100_69-118         ..........CRICLDI............GGE..............DLIAPC..HCKGT
lcl|Glyma.11G236400_33-82          ..........CRICLET............DGR..............DFIAPC..KCKGT
lcl|Glyma.18G020800_33-82          ..........CRICLET............DGR..............DFIAPC..KCKGT
lcl|Glyma.05G182200_29-78          ..........CRICLET............DGR..............NFIVPC..KCKGT
lcl|Glyma.05G010700_54-89          ...........RICLES............DSDPE...........DELISPC..MCKGT
lcl|Glyma.17G118400_55-107         ..........CRICLES............DSDPE...........DELISPC..MCKGT
lcl|Glyma.02G310000_267-316        ..........CRICLVELG..........EGGNT.............LKMEC..SCKGD
lcl|Glyma.14G002700_268-317        ..........CRICLVELG..........EGGNT.............LKMEC..SCKGD
lcl|Glyma.03G180200_231-280        ..........CRICLIELG..........EGSDT.............LKMEC..SCKGE
lcl|Glyma.19G180900_243-292        ..........CRICLIELG..........EGSDT.............LKMEC..SCKGE
lcl|Glyma.13G005300_253-302        ..........CRICLVELV..........EGGNT.............LRMEC..SCKGE
lcl|Glyma.20G064600_253-302        ..........CRICLVELA..........EGGNT.............LRMEC..SCKGE
lcl|Glyma.11G147100_266-315        ..........CRICLVDLC..........EGGET.............FKLEC..SCKGE
lcl|Glyma.12G068000_266-315        ..........CRICLVDLC..........EGGET.............FKLEC..SCKGE
lcl|Glyma.12G173300_234-283        ..........CRICLVDLC..........EGGET.............LKMEC..SCKGE
lcl|Glyma.13G326500_259-308        ..........CRICLVDLC..........EGGET.............LKMEC..SCKGE
lcl|Glyma.10G051800_240-289        ..........CRICMVELG..........EGGNT.............FKLEC..GCKGD
lcl|Glyma.13G139200_260-309        ..........CRICFVELG..........EGADT.............FKLEC..SCKGE
lcl|Glyma.13G213600_217-265        ..........CRICF.DVC..........DERNT.............FKMEC..SCKGD
lcl|Glyma.15G099100_214-262        ..........CRICF.DVC..........DERNT.............FKMEC..SCKGD
lcl|Glyma.01G107700_99-151         ...........RICHLG...LESD.S.QEESGVP.............IELGC..SCKDD
lcl|Glyma.U027600_104-157          ...........RICHLG...LESDDS.QEESGVP.............IELGC..SCKDD
lcl|Glyma.07G142300_108-159        ...........RICHMG...LESD..SHESGAP.............IQLGC..SCKDD
lcl|Glyma.02G253000_84-135         ...........RICHLS...MDMT..NHESGTP.............IELGC..SCKDD
lcl|Glyma.14G063500_84-135         ...........RICHLS...MDMT..NHESGTS.............IELGC..SCKDD
lcl|Glyma.13G227900_172-233        ...........RICHLTSVQSLDETTVGTASSAKS.A........DLIQLGC..ACKDE
lcl|Glyma.15G084600_183-244        ...........RICHLTSVQSSDETTVGTASSATS.A........DLIQLGC..ACKDE
lcl|Glyma.04G063700_161-222        ...........RICHLTSGQSLNATTVGTVESATS.E........DLIQLGC..ACKDE
lcl|Glyma.06G064900_158-219        ...........RICHLTSGQSSDATTVGTSDSATS.A........DLIQLGC..ACKGK
lcl|Glyma.05G159600_181-243        ...........RICHLASGQPLEAADVGTASSATTNT........DLIQLGC..ACKDE
lcl|Glyma.04G084500_138-184        ..........CPVCLEEMG..........KEQN...............LVACG.TCRNP
lcl|Glyma.06G086100_152-198        ..........CPVCLEEMG..........KEQN...............LVACG.TCRNP
lcl|Glyma.02G232900_1829-1880      ..........CPICYSVIH..........TTNHG............LPRLACK.TCKHK
lcl|Glyma.01G095300_99-145         ..........CAICL..............LEFEDDN.........VLRLLTL...CCHV
lcl|Glyma.08G270800_99-145         ..........CAICL..............LEFEDDN.........MVRLLTL...CCHV
lcl|Glyma.01G017200_110-156        ..........CAICL..............LEFDHDS.........MLRLLTV...CYHV
lcl|Glyma.02G210700_138-184        ..........CAVCL..............NEFLDDE.........TLRLIPK...CCHV
lcl|Glyma.14G178400_137-183        ..........CAVCL..............NEFRDDE.........TLRLIPK...CCHV
lcl|Glyma.04G098100_128-174        ..........CAVCL..............NEFEEDE.........TLRFIPN...CSHV
lcl|Glyma.06G099800_131-177        ..........CAVCL..............NEFEEVE.........TLRFIPN...CSHV
lcl|Glyma.10G156500_116-162        ..........CAVCL..............NEFEDTE.........TLRLIPK...CDHV
lcl|Glyma.20G231900_117-163        ..........CAVCL..............NEFEDTE.........TLRLIPK...CDHV
lcl|Glyma.19G236800_118-164        ..........CAVCL..............CEFEDTE.........TLRLIPK...CDHV
lcl|Glyma.03G239900_110-156        ..........CAVCL..............CEFEDTE.........TLRLLPK...CDHV
lcl|Glyma.09G140700_126-172        ..........CAVCL..............TDFTDKD.........ALRLLPK...CNHV
lcl|Glyma.16G194600_116-162        ..........CAVCL..............TDFTHKD.........SLRLLPK...CNHV
lcl|Glyma.09G140800_108-154        ..........CAVCL..............AEFDDAD.........ALRLLPK...CGHV
lcl|Glyma.11G242800_110-156        ..........CAVCL..............VEFEDSD.........TIKMLPK...CQHV
lcl|Glyma.14G037000_80-126         ..........CAVCL..............GEFEDCD.........VVKMLPK...CEHI
lcl|Glyma.06G118400_62-106         ..........CAVCL..............SEFEECD.........MVKIQPK...CEHI
lcl|Glyma.01G017300_141-187        ..........CSVCL..............SEFQDDE.........SVRLLPK...CSHA
lcl|Glyma.09G205600_131-177        ..........CSVCL..............SEFRDDE.........SVRLLPK...CSHA
lcl|Glyma.01G095600_140-186        ..........CSVCL..............SEFQDDE.........SVRLLPK...CSHV
lcl|Glyma.08G271100_141-187        ..........CSVCL..............SEFEDDE.........SVRLLPK...CSHV
lcl|Glyma.04G128100_143-189        ..........CSVCL..............SEFEENE.........DLRLLPK...CNHA
lcl|Glyma.06G310600_135-181        ..........CSVCL..............IEFQENE.........NLRLLPK...CNHA
lcl|Glyma.02G210400_152-198        ..........CSVCL..............NEFQEEE.........TLRLLPK...CNHA
lcl|Glyma.14G177900_153-199        ..........CSVCL..............NEFQEEE.........TLRLLPK...CNHA
lcl|Glyma.08G070100_155-204        .......GTECSVCL..............SEFQEDE.........SLRLLPK...CNHA
lcl|Glyma.13G042300_156-201        ...........SVCL..............SEFQEDE.........SLRLLPK...CNHA
lcl|Glyma.08G177000_180-226        ..........CAVCL..............SEFQEDE.........NLRLLPK...CQHA
lcl|Glyma.15G056000_166-212        ..........CAVCL..............SEFQEDE.........NLRLLPK...CHHA
lcl|Glyma.05G175400_149-200        .......GTECSVCL..............GEFEHDE.........SLRLLPK...CSHA
lcl|Glyma.18G014500_194-240        ..........CLVCL..............GEFQQEE.........SLRVLPK...CNHA
lcl|Glyma.18G014600_153-202        .......ETECLVCL..............GEFHQEE.........SLRVLPK...CNHA
lcl|Glyma.11G242500_156-205        .......ESECLVCL..............GEFQQEE.........SLRVLPK...CNHA
lcl|Glyma.11G122400_135-179        .........DCAVCL..............LEFEDDD.........YVRTLPI...CSHT
lcl|Glyma.12G047300_133-176        ...........AVCL..............LEFEDED.........YVRTLPV...CSHT
```

```
lcl|Glyma.01G032200_147-191    .........DCAVCL...............CEFLEQD.........KLRLLPM...CNHA
lcl|Glyma.02G033200_149-193    .........DCAVCL...............CEFLEQD.........KLRLLPM...CNHA
lcl|Glyma.13G071600_172-216    .........DCAVCL...............CEFSEKD.........KLRLLPM...CSHA
lcl|Glyma.19G011200_168-212    .........DCAVCL...............CEFSEKD.........KLRLLPM...CSHA
lcl|Glyma.08G289200_148-194    ..........CAVCL...............CQFSEQD.........MLRLLPL...CNHA
lcl|Glyma.18G135300_149-195    ..........CAVCL...............CQFSEQD.........MLRLLPL...CNHA
lcl|Glyma.05G035800_139-185    ..........CAVCL...............CEFSKED.........KLRLLPM...CTHA
lcl|Glyma.17G091600_134-180    ..........CAVCL...............CEFSEDD.........KLRLLPM...CTHA
lcl|Glyma.13G092000_129-175    ..........CAVCL...............CEFEPED.........KLRLLPK...CSHA
lcl|Glyma.17G068300_125-171    ..........CAVCL...............CEFEPED.........KLRLLPK...CSHA
lcl|Glyma.03G103800_137-183    ..........CAVCL...............SKFHHHD.........LLRLLPL...CCHA
lcl|Glyma.07G120300_138-184    ..........CAVCL...............SKFHHHD.........LLRLLPL...CCHA
lcl|Glyma.15G183300_121-167    ..........CAVCL...............SKFEQND.........LLRLLPL...CCHA
lcl|Glyma.01G146500_110-156    .......GLECAVCL...............SKFEDVE.........ILRLLPK...CKHA
lcl|Glyma.09G194100_115-161    .......GLECAVCL...............SKFEDVE.........ILRLVPK...CKHA
lcl|Glyma.04G083400_113-140    ..........CTVCL...............SKFEDTE.........TLRLLP........
lcl|Glyma.06G084800_126-176    .......GLECTVCL...............SKFEDTE.........TLRLLPK...CKHA
lcl|Glyma.14G146700_132-182    .......GLECTVCL...............SQFEDTE.........ILRLLPK...CKHT
lcl|Glyma.02G106400_97-139     ..........CAVCL...............NKFKVAK.........VLRLLSK...CKHA
lcl|Glyma.04G091400_117-163    ..........CAVCL...............NKFEAAE.........VLRLLPK...CKHA
lcl|Glyma.03G215500_93-139     ..........CAVCL...............SEFADGD.........EGRVLPN...CKHA
lcl|Glyma.19G212000_90-136     ..........CAVCL...............SEFSDGD.........EGRVLPN...CKHS
lcl|Glyma.02G016800_86-132     ..........CAVCL...............SEFADGE.........EGRFLPN...CNHA
lcl|Glyma.07G046400_108-154    ..........CAVCL...............SEIVQGE.........KLRLLPK...CNHG
lcl|Glyma.16G014600_107-153    ..........CAVCL...............SEIVEGE.........KLRLLPK...CNHG
lcl|Glyma.03G263000_99-148     .......GLECAVCL...............SEVVEGE.........KARLLPK...CNHG
lcl|Glyma.07G245400_93-139     ..........CAVCL...............SEFENGE.........TGRVLPK...CNHS
lcl|Glyma.17G028400_95-141     ..........CAVCL...............SEFENGE.........TGRVLPK...CNHS
lcl|Glyma.09G042400_118-164    ..........CAVCL...............SEFEPGE.........TGRVLPK...CNHS
lcl|Glyma.13G332800_88-134     ..........CAICL...............GEFEEGE.........WLKLLPN...CTHG
lcl|Glyma.06G284600_119-165    ..........CAVCL...............SALEGEE.........KAKLLPN...CNHF
lcl|Glyma.12G121400_124-171    ..........CAVCL...............SALEGEE.........KAKLLPN...CNHF
lcl|Glyma.12G208600_102-147    ..........CAVCL...............SVLEDGE.........HVRLLPN...CKHS
lcl|Glyma.13G292600_94-142     .......SVECTVCL...............SVLEDGE.........QVRLLPN...CKHS
lcl|Glyma.09G214600_99-144     ..........CAVCL...............GDFEDGE.........ELRTMPE...CMHS
lcl|Glyma.16G075300_86-130     .........TCAVCL...............EEFEEGE.........ELRRLPE...CMHF
lcl|Glyma.13G108500_112-158    ..........CPVCL...............SGFEEGE.........EVRKLPR...CKHW
lcl|Glyma.17G050900_108-154    ..........CPVCL...............SGFEEGE.........EVRKLPR...CKHW
lcl|Glyma.18G176200_157-203    ..........CPVCL...............SVFANGE.........EVRQLSA...CKHS
lcl|Glyma.02G228800_114-163    .......ESECVICL...............SVIEEGE.........IGRGLPK...CCHA
lcl|Glyma.01G157400_104-150    ..........CAICL...............ADFAAGD.........EIRVLPQ...CGHG
lcl|Glyma.11G087600_104-150    ..........CAICL...............AEFAAGD.........EIRVLPQ...CGHG
lcl|Glyma.04G015200_96-142     ..........CAICL...............TEFAAGD.........EIRVLPQ...CGHG
lcl|Glyma.06G015200_96-142     ..........CAICL...............TEFAAGD.........EIRVLPQ...CGHG
lcl|Glyma.09G196700_100-146    ..........CAICL...............TEFGAGD.........EVRVLPQ...CGHG
lcl|Glyma.16G112200_99-145     ..........CAICL...............TEFGAGD.........EIRVLPQ...CGHG
lcl|Glyma.04G215600_139-185    ..........CVICL...............SEFTSGD.........KVRILPK...CNHR
lcl|Glyma.06G150400_141-187    ..........CVICL...............SEFTSGE.........KVRILPK...CNHG
lcl|Glyma.05G188900_144-190    ..........CVICL...............SEFANGD.........KVRILPK...CNHG
lcl|Glyma.08G146600_143-189    ..........CVICL...............SEFANGD.........KVRILPK...CNHG
lcl|Glyma.09G275300_113-159    ..........CPICL...............GEFEKGD.........KVRMLPK...CNHG
lcl|Glyma.18G214200_108-154    ..........CPICL...............GEFEKGD.........RVRMLPK...CNHG
lcl|Glyma.07G173600_79-113     ..........CPICL...............DE..........................CNHG
lcl|Glyma.06G143100_111-157    ..........CPICL...............GEFVDGE.........KVRVLPK...CNHG
lcl|Glyma.08G088100_109-152    ...........VICL...............AEFSDGD.........PIRFLPK...CNHY
lcl|Glyma.15G157400_108-152    .........ICAICL...............TEFSDGD.........RIRFLPN...CNHR
lcl|Glyma.04G182100_194-239    ..........CLICL...............EEFHVGN.........QVRGLP....CAHN
lcl|Glyma.06G183300_233-278    ..........CLICL...............EEFHVGN.........QVRGLP....CAHN
lcl|Glyma.05G037000_234-279    ..........CPICL...............EEFYVGN.........EVRGLP....CAHN
lcl|Glyma.17G090200_234-279    ..........CPICL...............EEFYVGN.........EVRGLP....CAHN
lcl|Glyma.11G191800_129-174    ..........CAICL...............EELREGD.........AVKVIPY...CKHV
lcl|Glyma.01G151700_511-557    ..........CYICL...............VEYEDGD.........SMRVLP....CHHE
lcl|Glyma.09G216900_511-557    ..........CYICL...............VEYEDGD.........SMRVLP....CHHE
lcl|Glyma.16G076000_519-565    ..........CYICL...............VEYEDGD.........NMRVLP....CHHE
lcl|Glyma.16G107700_522-568    ..........CYICL...............VEYEDGD.........NMRVLP....CHHE
lcl|Glyma.13G165500_478-524    ..........CYICL...............ADYEEGD.........QIRVLP....CFHE
lcl|Glyma.17G105500_479-525    ..........CYICL...............ADYEEGD.........QIRVLP....CFHE
lcl|Glyma.01G217300_329-374    ..........CCICL...............SSYDDGV.........ELRELP....CGHH
lcl|Glyma.11G026000_332-377    ..........CCICL...............SSYDDGV.........ELRELP....CGHH
lcl|Glyma.05G048800_325-370    ..........CCICL...............SAYDDGV.........ELRQLP....CGHH
lcl|Glyma.17G130700_323-368    ..........CCICL...............SAYDDGV.........ELRKLP....CSHH
lcl|Glyma.09G265600_314-359    ..........CCICL...............SAYDDGA.........ELRELP....CNHH
lcl|Glyma.18G225500_320-365    ..........CCICL...............SAYDNDA.........ELRELP....CNHH
lcl|Glyma.02G085200_305-350    ..........CCICL...............CSYEDGA.........ELHALP....CNHH
lcl|Glyma.07G170200_304-349    ..........CCICL...............CSYEDGA.........ELHALP....CNHH
lcl|Glyma.05G238900_292-337    ..........CCICI...............SSYEDGA.........ELHVLP....CNHH
lcl|Glyma.08G046000_293-338    ..........CCICI...............SSYEDGA.........ELHVLP....CNHH
lcl|Glyma.03G120200_273-318    ..........CCICL...............CPYVEGA.........ELYRLP....CTHH
lcl|Glyma.19G124800_275-320    ..........CCICL...............CPYVEGE.........ELYRLP....CTHH
lcl|Glyma.02G302400_289-335    .......DPECCICL...............AKYKDEE.........EVRQLP....CSHM
lcl|Glyma.14G011800_289-335    .......DPECCICL...............AKYKDKE.........EVRQLP....CSHM
lcl|Glyma.08G332300_279-325    .......DPECCICL...............AKYKDKE.........EVRQLP....CSHL
lcl|Glyma.18G074600_278-324    .......DPECCICL...............AKYKDKE.........EVRQLP....CSHL
lcl|Glyma.13G361000_365-409    ...........CICL...............AKYADDD.........ELRELP....CSHV
lcl|Glyma.15G012900_392-436    ...........CICL...............AKYADDD.........ELRELP....CSHF
lcl|Glyma.14G129100_343-387    ...........CICL...............AKYENND.........ELRELP....CSHL
lcl|Glyma.17G203800_375-419    ...........CICL...............AKYENND.........ELRELP....CSHL
lcl|Glyma.01G162900_187-232    ...........SVCL...............QDFMLGE.........TVRSLPH...CHHM
lcl|Glyma.11G080500_187-232    ...........SVCL...............QDFMLGE.........TVRSLPH...CHHM
lcl|Glyma.02G044300_186-231    ...........SVCL...............QDFQLGE.........TGRSLPH...CHHI
lcl|Glyma.04G143800_84-127     ..........CSICF...............QDFEDEE.........FVRTLPK...CGHF
lcl|Glyma.08G316400_182-225    ..........CSICF...............QDFEYEE.........FVRTLPK...CGHF
lcl|Glyma.18G096600_201-244    ..........CSICF...............QDFEDEE.........FVRTLPK...CGHF
lcl|Glyma.02G291200_187-233    ..........CSICF...............QDFEDGE.........LVRILPK...CDHL
lcl|Glyma.05G025200_170-216    ..........CAICL...............QDIEVGE.........IARSLPR...CHHT
lcl|Glyma.17G101800_167-213    ..........CAICL...............QDIEVGE.........IARSLPR...CHHT
lcl|Glyma.10G124600_688-735    .........ACAICL...............ETPVQGE.........IIRHLP....CLHK
lcl|Glyma.20G076700_718-765    .........ACAICL...............EIPVQGE.........TIRHLP....CLHK
lcl|Glyma.10G217500_232-277    ..........CAICL...............EDYCVGE.........KLRILP....CCHK
lcl|Glyma.20G174500_247-292    ..........CAICL...............EDYCVGE.........KLRILP....CCHK
lcl|Glyma.11G137400_208-259    .......ELTCTICL...............DQVKRGE.........LVRSLP....CLHQ
lcl|Glyma.12G060800_236-287    .......ELTCTICL...............DQVKRGE.........LVRSLP....CLHQ
lcl|Glyma.11G220200_212-254    ..........CSVCL...............EQVDVGD.........VLRSLP....CLHQ
```

```
lcl|Glyma.18G037200_212-254   ..........CSVCL..............EQVNVGD.........VLRSLP....CLHQ
lcl|Glyma.02G194400_103-147   ...........SICL..............GDYKGSD.........LLRVLPD...CDHV
lcl|Glyma.10G083100_103-147   ...........SICL..............GDYKGSD.........FLRVLPD...CDHV
lcl|Glyma.03G203400_104-149   ..........CSICL..............ADYKGTD.........MLRMLPD...CGHQ
lcl|Glyma.10G121100_107-150   ..........CSICL..............ADYKDTD.........CVKLLSN...CGHL
lcl|Glyma.10G121500_113-160   ........LCCSICL..............ADYKNTE.........WLKLLPD...CGHM
lcl|Glyma.03G017500_84-127    ..........CVICL..............AEYKEKE.........LLRIIPK...CGHT
lcl|Glyma.07G078800_84-127    ..........CVICL..............AEYKEKE.........LLRIIPK...CGHT
lcl|Glyma.09G264300_89-135    ..........CVICL..............ADYKERE.........VLRIMPK...CGHT
lcl|Glyma.18G227700_89-135    ..........CVICL..............ADYRERE.........VLRIMPK...CGHT
lcl|Glyma.10G202500_86-132    ..........CTVCL..............SEYQGED.........MLRILPY...CGHS
lcl|Glyma.20G187900_86-132    ..........CTVCL..............SEYQGED.........MLRILPY...CGHS
lcl|Glyma.09G001600_110-156   ..........CSVCL..............LDYQAED.........RLQQIPA...CGHT
lcl|Glyma.05G217000_334-378   .........TCAICL..............SEYQPKE.........TLRSIPE...CNHY
lcl|Glyma.08G022900_332-375   ..........CAICL..............CEYEAKE.........TLRSIPQ...CNHY
lcl|Glyma.07G056200_314-357   ..........CWICL..............SEYNSKE.........TIRLIPE...CKHC
lcl|Glyma.16G025100_320-363   ..........CWICL..............SEYNSKE.........TIRLIPE...CKHC
lcl|Glyma.19G255700_319-362   ..........CTICL..............SEYKTKD.........TIRCIPE...CAHC
lcl|Glyma.06G127300_326-371   ..........CSICL..............SEYIPKE.........TVKTIPE...CGHC
lcl|Glyma.07G062800_119-162   ..........CSICL..............CEYKDSE.........MLRMMPE...CRHY
lcl|Glyma.16G030900_115-158   ..........CSICL..............CEYKDSE.........MLRMMPE...CRHY
lcl|Glyma.09G253500_112-155   ..........CSICL..............CEYKDSE.........MLRMMPE...CRHY
lcl|Glyma.18G239400_89-132    ..........CSICL..............CEYKDSE.........MLRMMPE...CRHY
lcl|Glyma.09G253400_107-153   ..........CSICI..............EDYEDSE.........MLRMMPQ...CRHY
lcl|Glyma.02G264800_105-151   ..........CCICL..............GVFADGE.........KLKVLPG...CDHS
lcl|Glyma.14G058000_100-146   ..........CCICL..............GAFADGE.........KLKVLPG...CDHS
lcl|Glyma.11G231400_107-153   ..........CCICL..............GEFRDGE.........KVKVLPA...CDHY
lcl|Glyma.18G025700_107-153   ..........CCICL..............GEFRDGE.........KVKVLPA...CDHY
lcl|Glyma.11G169500_92-138    ..........CCICL..............SLFRGNE.........KLKVLIE...CEHV
lcl|Glyma.18G060400_108-154   ..........CCICL..............SLFQSNE.........KLKVLIE...CEHV
lcl|Glyma.01G093800_57-96     ..........CAVCL..............SKFGERD.........EVIRVMR...CEHV
lcl|Glyma.04G153400_57-104    ..........CAVCL..............SKFGEGD.........EVIRVMR...CEHV
lcl|Glyma.04G153500_74-120    .........DCAVCL..............CKMGETE.........ERIITLR...CGHV
lcl|Glyma.13G115900_83-134    ..........CAVCL..............SQLSVED.........EVRELMN...CYHV
lcl|Glyma.17G043900_85-135    ..........CAVCL..............SQLSVED.........EVRELMN...CYHV
lcl|Glyma.01G221300_81-127    ...........AVCL..............YEFEGED.........EIRRLTN...CRHI
lcl|Glyma.11G022400_87-133    ...........AVCL..............YEFEGED.........EIRRLTN...CRHI
lcl|Glyma.05G210000_86-133    ..........CAVCL..............SEFEEND.........EIRRLAN...CRHI
lcl|Glyma.08G016700_85-132    ..........CAVCL..............SEFEEND.........EIRQLAN...CRHI
lcl|Glyma.17G159400_72-117    .........SCAVCL..............YEFEGED.........EIRWLTN...YRHI
lcl|Glyma.02G223600_99-145    ..........CSVCL..............TQFEPES.........EINRLS....CGHL
lcl|Glyma.14G190300_98-144    ..........CSVCL..............TQFEPES.........EINRLS....CGHL
lcl|Glyma.13G009100_96-142    ..........CCVCL..............TKFEPES.........EINCLS....CGHI
lcl|Glyma.05G182000_68-114    ..........CRVCL..............SEFQEGE.........KVRNL.N...CRHT
lcl|Glyma.08G139700_68-114    ..........CRVCL..............SEFQEGE.........KVRNL.N...CRHT
lcl|Glyma.11G236600_73-119    ..........CRVCL..............SEFEEGE.........KVRKL.K...CQHT
lcl|Glyma.18G020400_70-116    ..........CRVCL..............SEFEQGE.........KLRKL.K...CQHT
lcl|Glyma.05G218700_107-152   ...........CVCL..............GEFELKE.........ELLQIPY...CKHV
lcl|Glyma.08G024800_108-153   ...........CVCL..............GEFELNE.........ELLQIPY...CNHV
lcl|Glyma.13G002800_104-153   .......DSLCCVCL..............GEFELKE.........ELVQIPY...CKHV
lcl|Glyma.20G066300_104-153   .......DSLCCVCL..............GEFELKE.........EVLQIPY...CKHV
lcl|Glyma.10G007200_117-163   ..........CSVCL..............SEFEQDE.........KLRVIPN...CSHV
lcl|Glyma.20G088900_139-185   ..........CSVCL..............SEFQQDE.........KLRVIPN...CSHV
lcl|Glyma.10G187100_136-182   ..........CAVCL..............NEFQEDE.........KLRVIPN...CSHV
lcl|Glyma.20G203400_135-181   ..........CAVCL..............NEFQEDE.........KLRIIPN...CCHV
lcl|Glyma.19G163600_128-173   ...........VVCL..............TEFQEHD.........MLKALPI...CKHA
lcl|Glyma.11G243400_93-139    ..........CCVCL..............CRFEDNQ.........EVSELP....CKHY
lcl|Glyma.18G013800_88-132    ..........CCVCL..............CRFEANQ.........EVSELP....CKHY
lcl|Glyma.13G232800_104-150   ..........CSVCL..............GTIVEDT.........ISRVLPN...CKHI
lcl|Glyma.13G232800_105-147   ...........SVCL..............GTIVEDT.........ISRVLPN...CKHI
lcl|Glyma.15G079900_101-151   .......VVECSVCL..............GTIVEDA.........ITRVLPN...CKHI
lcl|Glyma.04G021200_74-120    ..........CAVCL..............DEIESEQ.........PARLVPG...CNHG
lcl|Glyma.06G021400_73-119    ..........CAVCL..............DEIESEQ.........PARVVPG...CNHG
lcl|Glyma.14G221800_70-116    ..........CAVCL..............DEIGTEQ.........PVRVVPG...CNHA
lcl|Glyma.17G260700_70-116    ..........CAVCL..............DHIGTEQ.........PARLVPG...CNHA
lcl|Glyma.04G181300_83-127    ..........CAVCL..............ENFKVGD.........VCRLLPN...CSHS
lcl|Glyma.06G183600_83-127    ..........CAVCL..............ENFKIGD.........VCRLLPN...CGHS
lcl|Glyma.13G170300_69-114    ..........CAVCL..............ENLITGD.........KCRLLPM...CKHS
lcl|Glyma.19G010400_71-116    ..........CAVCL..............ENLITGD.........KCRFLPV...CKHS
lcl|Glyma.13G092100_125-170   ...........VVCL..............DAFRNAQ.........WCRKLAA...CGHV
lcl|Glyma.17G068200_100-145   ...........VVCL..............DAFHNAQ.........WCRKLAA...CGHV
lcl|Glyma.10G244900_121-165   ...........AVCL..............EDLGLEQ.........QVMNLS....CSHK
lcl|Glyma.20G149700_120-164   ...........AVCL..............EDLGQEQ.........QVMNLS....CSHK
lcl|Glyma.01G048700_113-166   .......DLECVVCL..............EEFGVGG.........VAKEMP....CKHR
lcl|Glyma.02G108300_190-234   ..........CVVCL..............EEFGVGG.........VAKEMP....CKHR
lcl|Glyma.02G071200_226-269   .........QCPVCL..............EDVEVGS.........EAKEMP....CMHK
lcl|Glyma.16G152400_225-267   ..........CTVCL..............EDVEVGS.........EAKEMP....CKHK
lcl|Glyma.18G000200_236-278   ..........CSVCL..............DDFEVGS.........EAKEMP....CKHR
lcl|Glyma.02G161600_186-230   ...........PVCK..............DKFEVGS.........EARQMP....CNHL
lcl|Glyma.10G101400_186-230   ...........PVCK..............DKFELGS.........KARQMP....CNHL
lcl|Glyma.02G276700_252-296   ...........PVCK..............EKFELGT.........EAREMP....CNHI
lcl|Glyma.14G039000_202-246   ...........PVCK..............EKFELGT.........EAREMP....CNHI
lcl|Glyma.13G072800_206-250   ...........PVCK..............ERFELGS.........EARKMP....CNHV
lcl|Glyma.13G072900_126-169   .........KCSVCI..............ERFEVGS.........EARKMP....CDHI
lcl|Glyma.04G251600_220-265   ..........CPVCQ..............EEFEVGG.........EARELQ....CKHI
lcl|Glyma.08G185400_202-246   ...........PICK..............DEFLLDM.........EARELP....CKHF
lcl|Glyma.15G047300_199-243   ...........PICK..............DEFELDM.........EARELP....CKHF
lcl|Glyma.09G165200_203-248   ..........CAVCK..............DTFELGE.........TAKQIP....CKHI
lcl|Glyma.16G213500_202-247   ..........CAVCK..............DTFELGE.........TAKQIP....CKHI
lcl|Glyma.10G285300_179-224   ..........CAVCQ..............DEFEKGS.........KVTQMP....CKHA
lcl|Glyma.20G103700_178-223   ..........CAVCQ..............DEFEKGS.........LVTQMP....CKHA
lcl|Glyma.02G249100_188-232   ...........AVCK..............EPFELST.........MAKEMP....CKHI
lcl|Glyma.14G067300_187-231   ...........AVCK..............EPFELCT.........MAKEMP....CKHI
lcl|Glyma.11G220400_185-229   ...........AVCK..............EAFETST.........AVREMP....CKHI
lcl|Glyma.18G037000_174-209   ................................IDDTH.........LAMEMP....CKHI
lcl|Glyma.07G134600_158-202   ...........AVCM..............ENFEINC.........DAREMP....CGHV
lcl|Glyma.18G184200_159-203   ...........AVCM..............ENFEINC.........DAREMP....CGHV
lcl|Glyma.11G137300_184-228   ...........AVCK..............EAFELHA.........EARELP....CKHI
lcl|Glyma.12G060700_182-226   ...........AVCK..............EVFELHA.........EARELP....CKHI
lcl|Glyma.13G338400_153-197   ...........AVCK..............EAFELGA.........LAREMP....CKHL
lcl|Glyma.15G036100_152-197   ..........CAVCK..............EAFELGE.........LAREMP....CKHL
lcl|Glyma.10G286700_262-306   ...........AICK..............DLLGVGD.........LAKRLP....CGHG
lcl|Glyma.20G102400_259-303   ...........AICK..............DLLGVGD.........AAKRLP....CGHR
```

```
lcl|Glyma.05G017500_280-324    ...........AVCK...............DEFGVGE.........GVKVLP....CSHR
lcl|Glyma.09G271200_369-413    ...........AICK...............DVLAPRT.........EVNQLP....CSHL
lcl|Glyma.18G218200_373-417    ...........AICK...............DVLTPGT.........EVNQLP....CSHL
lcl|Glyma.08G158300_95-136     ...........AVCK...............DQITPHA.........EAKQLP....CKHL
lcl|Glyma.15G268100_109-150    ...........AVCK...............DQITLNA.........QAKQLP....CQHL
lcl|Glyma.08G034000_52-100     ...........SVCM...............EGFITQRNTDG.....ENKRVP....CGHV
lcl|Glyma.06G272400_167-215    .......RKSCAICL...............EDFDPSE.........EVMLTP....CNHM
lcl|Glyma.12G131800_171-215    ...........AICL...............EDFEPSE.........EVMLTP....CNHM
lcl|Glyma.06G274300_168-216    .......SKSCAICL...............EDFDPSE.........EVMLTP....CNHM
lcl|Glyma.17G213300_14-59      ..........CAICL...............EDFEPSE.........EVMLTP....CNHT
lcl|Glyma.18G103300_98-136     ...........AICL...............EDFEPSE.........EVMLTP....CNHM
lcl|Glyma.12G223700_140-184    .........ECAICL...............EEFEVGQ.........LCQVFPE...CKHI
lcl|Glyma.13G277600_139-186    .........ECAICM...............EEFKVSQ.........LCQVFPE...CKHI
lcl|Glyma.12G151700_161-205    ..........CAICL...............EDFKKGE.........ECLVFSV...CGHT
lcl|Glyma.13G211100_115-163    ..........CPICM...............EDFKNGE.........LIQPFGV...CVHE
lcl|Glyma.17G211800_21-68      ..........CPICI...............EEFKNGE.........LIQPFGV...CVHE
lcl|Glyma.10G047200_194-237    .........ECVICK...............EEMGIGR.........DVCELP....CQHL
lcl|Glyma.13G135100_190-233    .........ECVICK...............EEMGIGR.........DVCELP....CQHL
lcl|Glyma.08G148600_91-141     .......DLMCSICL...............EEFLIGT.........ITIRLPHP..CYHI
lcl|Glyma.12G223800_101-147    ..........CVICL...............ESFITGE.........SCQILP.P..CNHL
lcl|Glyma.09G096300_141-186    ..........CAVCL...............ESFRVGE.........TLIHLP....CAHR
lcl|Glyma.15G201800_154-199    ..........CAVCL...............ESFRVGE.........TLIHLP....CAHR
lcl|Glyma.10G259300_189-234    ..........CSICL...............ESFTDGD.........ELIRLP....CGHK
lcl|Glyma.20G131600_187-232    ..........CSICL...............ESFTDGD.........ELIRLP....CGHK
lcl|Glyma.17G172100_265-310    ..........CPICL...............EEFEEGN.........L.IGKLHS..CIHK
lcl|Glyma.17G172200_262-307    ..........CPICL...............EEFEEGN.........L.IGKLHS..CIHK
lcl|Glyma.03G110700_123-171    .........DCPLCLFPLVP..........EDQESGT.........LPFMKLMS..CFHC
lcl|Glyma.10G289100_82-127     .........ICSICL...............VEYEGED.........A.VSKLGR..CGHV
lcl|Glyma.20G099800_77-122     .........ICSICL...............VEYEGED.........A.VSKLGR..CGHV
lcl|Glyma.16G006200_74-120     ..........CVICL...............TSFEEED.........S.VWKLHT..CRHI
lcl|Glyma.10G142800_16-53      ..........CPLIW.....................................GV..CNHA
lcl|Glyma.20G091400_16-53      ..........CPLIW.....................................GV..CNHA
lcl|Glyma.13G265400_16-53      ..........CPLIW.....................................GV..CNHA
lcl|Glyma.06G303600_75-104     ..........CTVAW.....................................GV..CNHA
lcl|Glyma.01G143600_200-252    .........KCNICM...............DEFYAEEGNEDDVKLLSSSSMP....CGHV
lcl|Glyma.17G108900_203-255    .........KCNICM...............DEFYAQEGNEDDVKLLSSSSMP....CGHV
lcl|Glyma.20G044300_97-142     ..........CPICL...............EELNINA.........ESYTMP....CHHL
lcl|Glyma.20G044600_112-155    .......DECCSICL...............EGLDIN...........VYTMP....CNHM
lcl|Glyma.09G257100_165-208    ...........PICL...............EEYDVEN..........PSNLT.K..CEHH
lcl|Glyma.18G235300_138-182    ...........PICL...............EEFDVEN..........PRNLT.K..CEHH
lcl|Glyma.08G016300_162-207    ..........CPICL...............EEYDAEN..........PKLAT.K..CDHH
lcl|Glyma.08G251300_174-216    .........VCPTCL...............EEYTEEN..........PKIVT.K..CSHH
lcl|Glyma.18G274000_173-215    .........VCPTCL...............EEYTEEN..........PKIVT.K..CSHH
lcl|Glyma.13G052000_177-221    ...........PTCL...............EEYTKEN..........PKIMT.K..CSHH
lcl|Glyma.19G034700_177-219    ...........PTCL...............EEYTKEN..........PKIMT.K..CSHH
lcl|Glyma.13G206800_34-78      ...........SICL...............EPFSVHD..........PSTVT.C..CKHE
lcl|Glyma.15G005300_166-208    .........TCPVCL...............ERLDQDT..........GGILTTI..CNHS
lcl|Glyma.01G019300_126-171    ...........GICMQSVRS.............GQGT.........AIFTAE....CSHT
lcl|Glyma.09G203400_19-64      ...........GICMQSARS.............GQGT.........AIFTAE....CSHT
lcl|Glyma.01G097900_111-159    ...........KICTRSVKT.............GEGK.........AIFTAE....CSHV
lcl|Glyma.08G273400_110-158    ...........EICMRSVKT.............GEGK.........AIFTAE....CSHV
lcl|Glyma.02G218200_86-131     .........NCGICSNSVKT.............GQGT.........AIYTAE....CGHA
lcl|Glyma.14G185600_109-152    .........SCGICSNSVKT.............GQGT.........AIYTAE....CGHA
lcl|Glyma.09G017200_92-144     ...........AICLDPLSYH............SKGSSP.....GQAIFTAQ....CSHT
lcl|Glyma.15G122900_92-144     ...........AICLDPLSYQ............SKGSSP.....GQAIFTAQ....CSHA
lcl|Glyma.07G270000_80-128     .........LCAICLDPLSHK............SKGS........KAIFTAQ....CSHA
lcl|Glyma.01G050400_262-297    ...................................MP.........TIGVLP....CCHA
lcl|Glyma.02G109200_262-297    ...................................MP.........TIGVLP....CCHA
lcl|Glyma.12G235700_273-332    .........CCGVCSKLLTEKSSWGTQ.KIIASNDLS.........VVSVLI....CGHV
lcl|Glyma.13G201200_274-333    .........CCGVCSKLLTEKSSWGTQ.KIIASNDLS.........VVSVLI....CGHV
lcl|Glyma.07G049700_1718-1767  .........CC.VCNCPLTKN............SVSS.........GIRIFN....CGHA
lcl|Glyma.16G018400_1710-1759  .........CC.VCNCPLTKN............SVSS.........GIRIFN....CGHA
lcl|Glyma.01G228500_290-332    ..........CIICREEMT...........................TAKKLV....CGHL
lcl|Glyma.11G011300_290-332    ..........CIICREEMT...........................TAKKLI....CGHL
lcl|Glyma.04G130500_337-383    ..........CAICREPMA...........................KAKRLN....CNHL
lcl|Glyma.06G316600_337-383    ..........CAICREPMA...........................KAKRLN....CNHL
lcl|Glyma.14G105900_232-269    ..........CAICRENLVLN........................DKMQELP....CKHT
lcl|Glyma.17G220500_232-277    ..........CAICRENLVLN........................DKMQELP....CKHT
lcl|Glyma.16G032000_12-61      ..........CSICYEPLN..........................PINEDLQSVSICGHV
lcl|Glyma.03G104400_860-896    ..........CTACT...............FTLDLPAV........HFMCMHS.......
lcl|Glyma.07G119500_861-897    ..........CTACT...............FTLDLPAV........HFMCMHS.......
lcl|Glyma.12G116100_524-572    ..........CVICM...............TAIDLSPR........SNDCMVTP..CDHF
lcl|Glyma.12G205300_519-567    ..........CVICM...............TAIDLSPR........SNDCMVTP..CDHF
lcl|Glyma.06G290800_524-572    ..........CVICM...............TAIDISPR........SNDCMVTP..CDHF
lcl|Glyma.13G295600_507-555    ..........CVICM...............TAIDLSQR........SNDCMVTP..CDHF
lcl|Glyma.01G195200_69-131     .........ICPISLEP.M...........QDPI...................TLCT.GQ
lcl|Glyma.11G046500_134-196    .........ICPISLEP.M...........QDPV...................TLCT.GQ
lcl|Glyma.03G202600_12-76      .........LCPISLEI.M...........KDPV...................TVST.GI
lcl|Glyma.10G262600_10-74      .........LCPISLEI.M...........KDPV...................TVST.GI
lcl|Glyma.19G200200_10-74      .........LCPISLDI.M...........KDPV...................TVST.GI
lcl|Glyma.04G212300_16-53      ...........PISLDL.F...........EDPV...................TLCT.GQ
lcl|Glyma.06G154100_14-76      .........RCPISLDL.F...........EDPV...................TLCT.GQ
lcl|Glyma.05G192100_12-74      .........RCPISLDL.L...........EDPV...................TLCT.GQ
lcl|Glyma.08G000400_12-74      .........RCPISLDL.F...........EDPV...................TLCT.GQ
lcl|Glyma.05G189600_16-52      ...........PISLDV.M...........KSPV...................SLCT.GV
lcl|Glyma.08G147200_16-52      ...........PISLDV.M...........KSPV...................SLCT.GV
lcl|Glyma.07G106000_30-93      ........FRCPISLDL.M...........KDPV...................TLST.GI
lcl|Glyma.15G064800_12-50      ..........CPISLQI.M...........SDPV...................ILSS.GH
lcl|Glyma.16G048700_180-229    ........VQCPICLEYPM...........CPQI...................TTC..GH
lcl|Glyma.19G103000_221-270    ........VQCPICLEYPL...........CPQI...................TSC..GH
lcl|Glyma.05G237500_1120-1165  ...........PICCDDLF...........TSSAT................VRALPC.GH
lcl|Glyma.08G044700_1118-1163  ...........PICCDDLF...........TSSAT................VRALPC.GH
lcl|Glyma.07G093700_1126-1171  ...........PICCDDLF...........TSSAT................VRALPC.GH
lcl|Glyma.09G182600_1122-1167  ...........PICCDDLF...........TSSAT................VRALPC.GH
lcl|Glyma.09G115100_1126-1168  ..........CPICCDFLF...........TSSES................VRALPC.GH
lcl|Glyma.17G096900_1156-1201  ...........PICHEYIF...........TSCSP................VKALPC.GH
lcl|Glyma.14G130700_200-247    ..........CPVCFEYLF...........ESRND................VTVMPC.GH
lcl|Glyma.17G202700_200-247    ..........CPVCFEYLF...........ESRND................VTVMPC.GH
lcl|Glyma.06G074300_199-246    ..........CPICFEYLF...........ESVND................VTVLLC.GH
lcl|Glyma.06G116800_4-50       .........ECPVCLQSFD...........ERDAI................PRVLSC.GH
lcl|Glyma.10G228500_5-52       ..........CPVFLQSFD...........ERDAI................PRVLSC.GH
lcl|Glyma.08G241700_44-77      ..........CPICWESFN...........IVENV................PYVLWC.GH
lcl|Glyma.04G134700_4-52       .........CCSVCQTRYN...........EEERV................PLLLQC.GH
```

```
lcl|Glyma.11G157400_5-54        ..........CSVCQTRYN..........EEERV...............PLLLQC.GH
lcl|Glyma.04G224200_401-445     .........MCAICQ..............EKMQA...............PILLSC.KH
lcl|Glyma.06G140600_410-454     .........LCAICQ..............EKMQA...............PILLSC.KH
lcl|Glyma.18G217900_377-421     .........LCAICQ..............EKMHA...............PILLRC.KH
lcl|Glyma.08G354400_21-65       .......SFVCCVCL..............DLLYK...............PIVLSC.GH
lcl|Glyma.18G171400_21-65       .......SFVCCVCL..............DLLYK...............PIVLSC.GH
lcl|Glyma.15G237300_55-95       ..........CPVCL..............NAMYP...............PIHQCSNGH
lcl|Glyma.13G198800_100-140     ..........CPVCL..............NAMYP...............PIHQCSNGH
lcl|Glyma.04G235700_61-101      ..........CPVCT..............NSMYP...............PIHQCHNGH
lcl|Glyma.06G129000_62-102      ..........CPVCT..............NSMYP...............PIHQCHNGH
lcl|Glyma.03G257700_48-88       ..........CPVCT..............NSMYP...............PIHQCHNGH
lcl|Glyma.13G035400_631-678     ...........KICKMAVD..........GEKV...................KICGHS
lcl|Glyma.14G156600_631-678     ...........KICKMAVD..........GEKV...................KICGHS
lcl|Glyma.12G192700_673-714     ...........GICHEPVE..........DVVV...................TTCEHA
lcl|Glyma.13G309700_670-711     ...........GICHEPVE..........DVVV...................TSCEHA
lcl|Glyma.07G148900_159-201     .......ELECGICLEINS..........KVVL...................PNCNHS
lcl|Glyma.18G200100_157-199     .......EEECGICLEMNS..........MVVL...................PNCNHS
lcl|Glyma.03G060000_142-184     .......EEECGICMEMNN..........KVVL...................PNCNHS
lcl|Glyma.07G195400_147-189     .......EDECGICMDMNS..........KIVL...................PNCNHA
lcl|Glyma.13G180900_147-189     .......EDECGICMDMNS..........KIVL...................PNCNHA
lcl|Glyma.13G240800_150-198     .......EEECGICMEMNS..........KIVL...................PDCNHV
lcl|Glyma.15G072700_147-189     .......EEECGICMEMNS..........KIVL...................PDCNHV
lcl|Glyma.18G063500_143-185     .......EEECGVCLEVKA..........KVVL...................PNCCHY
lcl|Glyma.05G144300_156-204     .......EDECGICLEPCT..........KMVL...................PNCCHA
lcl|Glyma.08G100700_156-204     .......EDECGICLEPCT..........KMVL...................PNCCHA
lcl|Glyma.11G246100_158-206     .......EDECGICLEPCT..........KMVL...................PGCCHA
lcl|Glyma.18G011100_158-206     .......EDECGICLEPCT..........KMVL...................PGCCHA
lcl|Glyma.05G044700_154-201     .......EHECGICLESCT..........KMVF...................PNCCHA
lcl|Glyma.11G246300_78-119      ........EECGICMENGM..........KMVL...................PNCGHS
lcl|Glyma.18G010900_145-192     ........EECGICMENGM..........KMVL...................PNCGHS
lcl|Glyma.02G309100_117-162     ........LNCSICMQLPE..........RPVT...................TPCGHN
lcl|Glyma.14G003700_117-162     ........LNCSICMQLPD..........RPVT...................TPCGHN
lcl|Glyma.12G001300_129-175     .......SLNCSICIQLPE..........RPVT...................SPCGHS
lcl|Glyma.05G198100_138-187     ..........CSICLNLWH..........DVVTV..................APCLHN
lcl|Glyma.04G165900_213-256     .......DFDCTLCLKLLY..........EPVT...................TPCGHS
lcl|Glyma.06G196600_193-236     .......DFDCTLCLKLLY..........EPVT...................TPCGHS
lcl|Glyma.16G063400_170-217     .......ELSCAICLEICF..........EPST...................TPCGHS
lcl|Glyma.19G082000_171-218     .......ELSCAICLEICF..........EPST...................TPCGHS
lcl|Glyma.12G180800_764-813     .......QGECPICLEVFE..........DAVL...................TPCAHR
lcl|Glyma.13G320000_790-839     .......QGECPICLEVFE..........DAVL...................TPCAHR
lcl|Glyma.20G087900_859-904     .......IIECSICMESPE..........DPVF...................TPCAHK
lcl|Glyma.02G149800_290-333     .........LCVICLEQEY..........NAVF...................VPCGHM
lcl|Glyma.10G024100_292-330     ...........VICLEQEY..........NAVF...................VPCGHM
lcl|Glyma.03G080300_332-382     .......GQLCVICLMRRR..........RSVF...................IPCGHL
lcl|Glyma.16G008300_327-377     .......GQLCVICLMRRR..........RSVF...................IPCGHL
lcl|Glyma.18G149800_331-381     .......GQLCVICLMRRR..........RSVF...................IPCGHL
lcl|Glyma.04G043900_344-393     .......EKLCCICYDEQR..........SSFF...................VPCGHC
lcl|Glyma.06G044100_357-407     .......EKLCCICYDEQR..........NSFF...................VPCGHC
lcl|Glyma.07G150500_385-433     ........HLCAICFDAPR..........DCFF...................LPCGHC
lcl|Glyma.18G201500_386-434     ........RLCAICFDAPR..........DCFF...................LPCGHC
lcl|Glyma.10G181100_405-450     ...........VICLDAPA..........EGAC...................IPCGHV
lcl|Glyma.20G209400_406-451     ...........VICLDAPA..........EGAC...................IPCGHV
lcl|Glyma.03G180500_289-337     .......QGKCVICLSEPR..........DTIV...................LPCRHM
lcl|Glyma.19G181100_312-356     ...........VICWSEPR..........DTIV...................HPCRHM
lcl|Glyma.10G052000_291-337     .........ECVICLSEPR..........DTTV...................LPCRHM
lcl|Glyma.13G139400_291-337     .........ECVICLSEPR..........DTTV...................LPCRHM
lcl|Glyma.13G326700_239-283     ...........VICLSEPP..........DITV...................LPCRHM
lcl|Glyma.07G257200_279-325     .........ECVICMTEPK..........DTAV...................LPCRHM
lcl|Glyma.17G016900_219-265     .........ECVICMTEPK..........DTAV...................LTCRHM
lcl|Glyma.13G208500_314-360     .........ECVICMTEPK..........DTAV...................LPCRHM
lcl|Glyma.15G104300_316-362     .........ECVICMTEPK..........DTAV...................LPCRHM
lcl|Glyma.09G048200_866-915     .......KGNCCICYEMKV..........DSVL...................YRCGHM
lcl|Glyma.15G155500_861-910     .......KGNCCICYEMKV..........DSVL...................YRCGHM
lcl|Glyma.10G198600_793-842     .......KGTCCVCCDNHI..........DSLL...................YRCGHM
lcl|Glyma.20G191400_792-841     .......KGTCCVCCDNHI..........DSLL...................YRCGHM
lcl|Glyma.11G191900_808-850     ...........CICCESNI..........DSLL...................YRCGHL
lcl|Glyma.12G082400_798-847     .......KGLCCICCESNI..........DSLL...................YRCGHL
lcl|Glyma.12G179300_740-789     .......KGLCCICCESNI..........NSLL...................YRCGHM
lcl|Glyma.13G321300_793-842     .......KGLCCICCESNI..........DSLL...................YRCGHM
lcl|Glyma.13G212200_825-875     .......ERECVMCLSEEM..........SVVF...................LPCAHQ
lcl|Glyma.15G100500_827-877     .......ERECVMCLSAEM..........SVVF...................LPCAHQ
lcl|Glyma.17G020500_819-869     .......ERECVMCLSEEM..........SVVF...................LPCAHQ
lcl|Glyma.07G253800_821-871     .......EQECVMCLSEEM..........SVLF...................MPCAHQ
lcl|Glyma.02G150400_776-826     .......EQECVMCLSEEM..........SVVF...................LPCAHQ
lcl|Glyma.05G112600_659-709     .......NRECIVCMKDEV..........SIVF...................LPCAHQ
lcl|Glyma.17G154400_621-671     .......NRECIVCMKDEV..........SIVF...................LPCAHQ
lcl|Glyma.11G042600_620-669     ........RECIICGKDEV..........SVIF...................LPCAHQ
lcl|Glyma.04G009100_1020-1065   .......SHVCKVCFESPT..........AAIL...................LPCRHF
lcl|Glyma.06G009000_1020-1065   .......SHVCKVCFESPT..........AAIL...................LPCRHF
lcl|Glyma.16G098500_18-63       .......SHVCKVCFESPT..........TAIM...................LPCRHF
lcl|Glyma.07G159000_32-77       .......SHVCKVCFESPT..........TVIL...................LSCRHF
lcl|Glyma.11G112700_1028-1064   ...........KVCFQSST..........AAIL...................LPCRHF
lcl|Glyma.12G038600_1038-1083   .......SHVCKVCFESST..........AAIL...................LPCRHF
lcl|Glyma.05G013200_415-460     .......KVLCRICFEGEI..........NVVL...................LPCRHR
lcl|Glyma.17G121200_415-460     .......KVLCRVCFEGEI..........NVVL...................LPCRHR
lcl|Glyma.10G204900_433-478     .......KILCRICFEEQI..........NVVL...................LPCRHH
lcl|Glyma.20G185600_422-467     .......KILCRICFEEQI..........NVVL...................LPCRHH
lcl|Glyma.01G029500_286-325     .......NLTCRACKTKTV..........SMLL...................MPCRHL
lcl|Glyma.02G035800_286-325     .......NLTCRACKVKTV..........SMLL...................MPCRHL
lcl|Glyma.14G083900_301-342     .......MMTCKACRVNEV..........TMVL...................LPCKHL
lcl|Glyma.17G241100_292-333     .......MMTCKACRVNEV..........TMVL...................LPCKHL
lcl|Glyma.08G170400_204-247     .........MCRNCGKEES..........CVLI...................LPCRHL
lcl|Glyma.15G256500_288-331     .........LCRNCRKEES..........CVLI...................LPCRHL
lcl|Glyma.09G019900_261-297     .........LCKNCGLRES..........VVLL...................LPCRHL
lcl|Glyma.15G126400_264-300     .........LCKNCGVRES..........VVLL...................LPCRHL
lcl|Glyma.01G203300_274-316     ..........CRGCAKRVA..........SVVV...................LPCRHL
lcl|Glyma.11G039700_281-323     ..........CRGCAKRVA..........SVVV...................LPCRHL
lcl|Glyma.05G068400_302-344     ..........CRGCAKRVA..........SVVV...................LPCRHL
lcl|Glyma.17G150900_309-344     ..........CRGCAKRVA..........SVVV...................LPCRHL
lcl|Glyma.09G282200_4713-4753   ..........CRVCLSSEV..........DITI...................VPCGHV
lcl|Glyma.20G003400_4712-4751   ...........RVCLSSEV..........DITI...................VPCGHV
lcl|Glyma.04G220000_1217-1265   .......DGLCCICYACEA..........NAQI...................APCSHR
lcl|Glyma.06G145900_1217-1265   .......DSLCCICYACEA..........DAQI...................APCSHR
```

```
lcl|Glyma.08G358300_319-375     .......MELCCICFDQAC...........TIEV....................RPCGHQ
lcl|Glyma.18G176600_318-374     .......MELCCICFDQAC...........TIEV....................RPCGHQ
lcl|Glyma.12G063600_316-372     .......TELCCICFEQVC...........TIEV....................QDCGHQ
lcl|Glyma.11G140000_316-372     .......TELCCICFEQVC...........TIEV....................QDCGHQ
lcl|Glyma.13G335300_323-381     .......SELCCICFEQVC...........TIEV....................QNCGHQ
lcl|Glyma.15G039100_316-374     .......SELCCICFEQVC...........TIEV....................QNCGHQ
lcl|Glyma.08G315500_281-340     .......KELCSICYDRVC...........TFVV....................RPCGHE
lcl|Glyma.03G079200_225-269     ...........CVCMVRHK...........GAAF....................IPCGHT
lcl|Glyma.16G094800_214-258     ...........CVCMVRHK...........GAAF....................IPCGHT
lcl|Glyma.08G350900_306-345     ..........CCVCMVRHK...........AAAF....................IPCGHT
lcl|Glyma.18G165000_306-353     ........HTCCVCMVKHK...........AAAF....................IPCGHT
lcl|Glyma.07G264100_155-203     .......DWVCCVCMGRKK...........GAAF....................IPCGHT
lcl|Glyma.17G009900_146-194     .......DWVCCVCMGRKK...........GAAF....................IPCGHT
lcl|Glyma.09G008200_144-192     .......DSVCCVCMGRKK...........GAAF....................IPCGHT
lcl|Glyma.15G112600_157-205     .......DSVCCVCMGRKK...........GAAL....................IPCGHT
lcl|Glyma.04G189900_380-423     .......NQVCPICLTDP............KDMA....................FGCGHQ
lcl|Glyma.06G175700_381-424     .......NQVCPICLTDP............KDMA....................FGCGHQ
lcl|Glyma.05G173500_413-456     .......SKVCPICLTND............KDMA....................FGCGHQ
lcl|Glyma.08G130800_413-456     .......NKVCPICLTNG............KDMA....................FGCGHQ
lcl|Glyma.11G244000_416-459     .......NKVCPICLTNA............KDMA....................FGCGHQ
lcl|Glyma.18G013100_412-455     .......NKVCPICLTNA............KDMA....................FGCGHQ
lcl|Glyma.05G115700_444-487     .......NQLCPICLTNS............KDMA....................FGCGHQ
lcl|Glyma.19G051500_449-492     .......NQLCPICLTNS............KDMA....................FGCGHQ
lcl|Glyma.19G078600_381-424     .......NQLCPICLSNA............KDMA....................FGCGHQ
lcl|Glyma.19G124300_227-270     .......NQLCPICLSNA............KDMA....................FGCGHQ
lcl|Glyma.16G068400_384-427     .......NQLCPICLSNA............KDMA....................FGCGHQ
lcl|Glyma.04G211000_388-427     ...........PICLTNP............KDMA....................FGCGHT
lcl|Glyma.05G193500_382-421     ...........PICLTNP............KDMA....................FGCGHT
lcl|Glyma.08G234200_376-419     .......QMACAICLTNK............KDLA....................FGCGHM
lcl|Glyma.15G219300_368-411     .......QMACAICLTNK............KDLA....................FGCGHM
lcl|Glyma.10G238000_315-375     .......IDFCAVCLERPC...........SVAA....................EGCGHE
lcl|Glyma.20G156600_315-375     .......IDFCAVCLERPC...........SVAA....................EGCGHE
lcl|Glyma.09G143600_314-374     .......VDACAVCLERQC...........SVAA....................EGCGHE
lcl|Glyma.01G178600_28-77       ........FECNICFDLAQ...........DPVI....................TLCGHL
lcl|Glyma.11G063500_28-77       ........FECNICFDLAQ...........DPVI....................TLCGHL
lcl|Glyma.04G034500_31-78       ..........CNICFELAQ...........DPII....................TLCGHL
lcl|Glyma.06G034500_29-78       ........FECNICFELAQ...........DPII....................TLCGHL
lcl|Glyma.03G145700_157-202     ...........NICLDLAR...........DPVV....................TCCGHL
lcl|Glyma.19G148900_156-201     ...........NICLDLAR...........DPVV....................TCCGHL
lcl|Glyma.08G157200_105-149     ...........NVCLDRAR...........DPVL....................TCCGHL
lcl|Glyma.15G269000_105-151     ...........NICLDKAR...........DPVL....................TSCGHL
lcl|Glyma.09G001100_123-170     ...........NICLDRAR...........DPVL....................ACCGHL
lcl|Glyma.12G240800_129-176     ...........NICLDRAR...........DPVL....................TCCGHL
lcl|Glyma.06G293400_33-91       ........FDCNICLECVQ...........DPVV....................TLCGHL
lcl|Glyma.12G112000_36-91       ...........NICLECVQ...........DPVV....................TLCGHL
lcl|Glyma.12G203900_46-99       ...........NICLECVQ...........DPVV....................TLCGHL
lcl|Glyma.13G297700_44-94       .........DCNICLECVQ...........DPVV....................TLCGHL
lcl|Glyma.12G151200_37-90       ...........NICMESAH...........DPVV....................TLCGHL
lcl|Glyma.07G196000_33-84       ..........CNICLDFAH...........EPVV....................TLCGHL
lcl|Glyma.13G179900_30-81       ..........CNICLDFAH...........EPVV....................TLCGHL
lcl|Glyma.13G239500_29-80       ..........CNICLDFAH...........EPVV....................TLCGHL
lcl|Glyma.15G073700_29-80       ..........CNICLDFAH...........EPVV....................TLCGHL
lcl|Glyma.14G099100_339-384     ..........CTLCLSNRQ...........HPTA....................TSCGHV
lcl|Glyma.17G225600_339-384     ..........CTLCLSNRQ...........HPTA....................TSCGHV
lcl|Glyma.01G074000_824-869     .......IIKCSICQDRA............KEVVI...................TKCYHL
lcl|Glyma.11G175700_818-875     ..EEYRDIIKCSICQDRA............KEVVI...................TKCYHL
lcl|Glyma.02G267300_828-871     ..........CTVCTDRP............KEVVI...................VKCYHL
lcl|Glyma.14G050200_828-871     ..........CTVCTDRP............KEVVI...................VKCYHL
lcl|Glyma.10G288200_718-762     ...........LVCNDPP............EEPVI...................TMCGHV
lcl|Glyma.20G100800_721-765     ...........LVCNDPP............EEPVI...................TMCGHV
lcl|Glyma.04G125600_69-110      ...........TVCQALLF...........DTSKC...................VPCSHV
lcl|Glyma.06G313000_69-110      ...........TVCQALLF...........DTSKC...................VPCSHV
lcl|Glyma.10G155500_28-80       ..........CPICLGPFL...........QLSYL...................DKCFHK
lcl|Glyma.20G232800_26-78       ..........CPICLGPFL...........QLSYL...................DKCFHK
lcl|Glyma.04G255000_11-57       ...........SVCHGNF............HIPCQ...................ANCSHW
lcl|Glyma.11G103800_150-191     ...........PICMGPMV...........HE....................MSTRCGHI
lcl|Glyma.12G028700_150-191     ...........PICMGPMV...........HE....................MSTRCGHI
lcl|Glyma.09G281300_151-194     ...........PICMSPLV...........EE....................MSTRCGHI
lcl|Glyma.20G004500_80-121      ...........PICMSALE...........EE....................TSTRCAHI
lcl|Glyma.03G124900_577-621     .......DFDCPICLSPPI...........EI....................VITRCAHI
lcl|Glyma.03G214100_219-270     ..........CSICLDTVF...........DP....................VSLTCGHI
lcl|Glyma.19G210900_219-269     ..........CSICLDTVF...........DP....................VSLTCGHI
lcl|Glyma.10G018800_210-261     ..........CSICLDTVF...........DS....................VSLTCGHI
lcl|Glyma.19G203000_218-270     ..........CAICLDFVF...........NP....................YALSCGHI
lcl|Glyma.08G153800_209-269     ..........CSVCLEHVL...........SKPTAAECK...........FGLLSECDHP
lcl|Glyma.15G272400_209-269     ..........CSVCLELVL...........SKPTAAECK...........FGLLSECDHP
lcl|Glyma.08G154100_188-251     ...........NVCLERVL...........SKPKPADCK...........FGLLPECDHA
lcl|Glyma.15G273000_188-250     ...........NVCLERVL...........SKPKPADCK...........FGLLPECDHA
lcl|Glyma.10G151500_34-80       ...........SICLEVVA...........DN...GDRS...........WSKL.QCGHQ
lcl|Glyma.20G236700_42-88       ...........SICLEVVA...........DN...GDRS...........WSKL.QCGHQ
lcl|Glyma.19G232800_18-67       ........VCCSICLEAVT...........DN...GDRS...........WAKL.QCGHQ
lcl|Glyma.02G219200_1354-1408   .......DETCPICQEKLG...........KQK...................MVFQCGHV
lcl|Glyma.14G186500_414-466     ..........CPICQEKLG...........KQK...................MVFQCGHV
lcl|Glyma.06G290400_82-126      ..........CPICLGIIK...........KTR...................TVMECLHR
lcl|Glyma.12G116500_60-104      ..........CPICLGIIK...........KTR...................TVMECLHR
lcl|Glyma.12G205500_121-165     ..........CPICLGIIK...........KTR...................TVMECLHR
lcl|Glyma.13G295300_124-168     ..........CPICLGIIK...........KTR...................TVMECLHR
lcl|Glyma.08G350700_40-90       ..DDIRKDVECPICLGIIR...........NTR...................VVMECMHR
lcl|Glyma.13G177200_138-181     ...........PICLGILR...........KTK...................AVMECMHR
lcl|Glyma.02G267800_40-81       .......DFLCPICMQIIK...........DP....................FLTACGHS
lcl|Glyma.14G049700_46-87       .......DFLCPICMQIIK...........DA....................FLTACGHS
lcl|Glyma.02G141500_20-65       ..........CPLCKKFLK...........E....................ATAISLCLH
lcl|Glyma.10G032500_19-64       ..........CPLCNKFLK...........E....................ATAISLCLH
lcl|Glyma.03G159500_34-79       ..........CPLCNKLFR...........E....................ATTISECLH
lcl|Glyma.19G161700_18-63       ..........CPLCGKLFR...........E....................ATTISECLH
lcl|Glyma.10G162800_19-64       ..........CPLCHNFFK...........D....................ATTISTCLH
lcl|Glyma.20G225400_20-65       ..........CPLCHKFFK...........D....................ATTISLCLH
lcl|Glyma.10G180300_32-77       ..........CPLCNKFFR...........N....................ATTISECCH
lcl|Glyma.03G165600_25-74       .........KCPLCLSLFK...........R....................PVLLP.CNH
lcl|Glyma.19G166900_24-66       ........LKCPLCLSLFK...........R....................PVLLP.CNH
lcl|Glyma.13G220400_468-515     ........LKCPICWSLLD...........S....................AVSLT.CNH
lcl|Glyma.15G092000_450-498     .......ELICPICWSLLD...........S....................AVSLT.CNH
lcl|Glyma.09G099300_2-43        ..........CNACWREVE...........G....................RAISTTCGH
```

```
lcl|Glyma.15G209600_2-43          ..........CNACWREVE...........G...................RAISTTCGH
lcl|Glyma.10G149400_424-470       ..........CGICLSEED...........KRRV................RGVLNCCTH
lcl|Glyma.20G238800_406-452       ..........CGICLSEED...........KRRV................RGVLNCCTH
lcl|Glyma.17G082300_27-72         ..........CGICYAESG............VSI................AGEIDCCSH
lcl|Glyma.19G042100_335-385       ........MSCVICLTEFS.............ST................RGILPCG.H
lcl|Glyma.09G206500_285-320       .........DCNICFSEYA.............GS................QFIRLPCEH
lcl|Glyma.02G103300_678-724       .......LEPCCICQEEFS...........DGEN...............VGSLD.CGHE
lcl|Glyma.07G214600_664-710       .......LEPCCICQDEFA...........DGEN...............VGSLD.CGHE
lcl|Glyma.04G039700_603-649       .......AEPCCVCQEDYG...........DGND...............IGTLD.CGHD
lcl|Glyma.04G039800_683-729       .......AEPCCVCQEDYG...........DGND...............IGTLD.CGHD
lcl|Glyma.06G041000_657-703       .......AEPCCVCQEDYG...........DEDD...............IGTLD.CGHD
lcl|Glyma.05G241900_382-427       ..........CSICQEEYE...........AGDE...............LGRLN.CEHS
lcl|Glyma.08G049400_328-373       ..........CSICQEEYE...........AGNE...............LGRLN.CEHI
lcl|Glyma.07G097700_305-350       ..........CSVCQEEYE...........SDDE...............LGRLK.CDHS
lcl|Glyma.09G180300_320-365       ..........CSVCQEEYE...........SDDE...............LGRLK.CDHS
lcl|Glyma.14G134600_501-543       ..........CCICQEEYV...........VGDE...............VGDLQ.CEHR
lcl|Glyma.17G198300_478-520       ..........CCICQEEYV...........VGDE...............VGDLQ.CEHR
lcl|Glyma.06G076600_488-533       ..........CSICQEEYV...........AAEE...............VGSLQ.CEHM
lcl|Glyma.03G179300_494-542       .......EGNCVICLEEYK...........NMDD...............VGTLKTCGHD
lcl|Glyma.19G180000_492-540       .......EGNCVICLEEYK...........NMDD...............VGTLQTCGHD
lcl|Glyma.10G051200_482-530       .......EEACAICLEEYK...........NMDD...............VGTLKACGHD
lcl|Glyma.13G138700_493-541       .......EETCAICLEEYK...........NMDD...............VGTLKACGHD
lcl|Glyma.06G229700_316-362       .......TDLCIICQDEYK...........NKEN...............IGILR.CGHE
lcl|Glyma.06G234500_258-302       .........LCIICQDEYK...........NKEN...............IGILR.CGHE
lcl|Glyma.11G156300_288-334       .......TDLCIICQDEYK...........NQEK...............IGILR.CGHE
lcl|Glyma.06G234300_402-449       .......TDSCIICQDEYK...........NQEK...............IGILQ.CGHE
lcl|Glyma.12G158500_402-449       .......NDSCIICQDEYK...........SQEK...............IGILQ.CGHE
lcl|Glyma.13G272700_474-517       ...........IICQDEFK...........NQEK...............IGVLQ.CEHE
lcl|Glyma.04G249100_149-193       ...........VICQVEYE...........EGEA...............LVALQ.CEHP
lcl|Glyma.06G113900_130-174       ...........VICQVEYE...........EGEA...............LVALQ.CEHP
lcl|Glyma.13G031100_152-196       ...........VICQVEYE...........EGEP...............LVAIQ.CEHP
lcl|Glyma.14G152800_154-198       ...........VICQVEYE...........EGES...............LVAIQ.CEHP
lcl|Glyma.10G284900_291-328       .........SCVICRVDYE...........DGES...............LTVLS.CKHL
lcl|Glyma.20G104300_280-331       .......NDSCVICRVDYE...........DDES...............LTVLS.CKHL
lcl|Glyma.11G132700_192-240       .......DERCVICQMEYR...........RGDK...............RITLP.CKHV
lcl|Glyma.12G057100_192-240       .......DERCVICQMEYK...........RGDK...............RITLP.CKHV
lcl|Glyma.12G237700_196-240       ...........VICQMTYR...........RGDQ...............QMKLP.CSHV
lcl|Glyma.13G203300_196-240       ...........VICQMTYR...........RGDQ...............QMKLP.CSHV
lcl|Glyma.03G184600_122-174       .......GLLCPICMDAWTN..........NGEHH..............ICCLP.CGHI
lcl|Glyma.14G147100_225-268       ..........CPICMDEFKV..........GGDK...............ACQLP.CTHT
lcl|Glyma.01G219800_243-291       ..........CEQCKS.GLHG.........ELMLLCDR.................CDKG
lcl|Glyma.11G023700_243-291       ..........CEQCKS.GLHG.........ELMLLCDR.................CDKG
lcl|Glyma.13G170000_1273-1321     ..........CKVCGI.DRDD.........DSVLLCDT.................CDAE
lcl|Glyma.19G010200_1251-1297     ..........CKVCGI.DRDD.........DSVLLCDT.................CDAE
lcl|Glyma.02G013500_1244-1297     ..........CKVCGS.RDRG.........DVMLICGDESGSV...........GCGIG
lcl|Glyma.10G014100_1243-1296     ..........CKVCGS.RDRG.........DVMLICGDESGSV...........GCGIG
lcl|Glyma.02G295400_391-439       ..........CQMCEL.SVNEV........DTVLLCDA.................CEKG
lcl|Glyma.14G018000_392-439       ...........QMCEL.SVNEV........DTVLLCDA.................CEKG
lcl|Glyma.09G059700_234-289       ..........CANCKLHEAFPD........NDIILCDG................TCNRA
lcl|Glyma.15G166200_251-303       ..........CANCKLHEAFPD........NDIILCDG................TCNRA
lcl|Glyma.17G059400_224-279       ..........CVKCTVREELPD........NDIILCNG................TCKRA
lcl|Glyma.10G260400_343-398       ..........CAKCQSKELSTN........NDIILCDG................VCDRG
lcl|Glyma.20G130800_344-399       ..........CAKCQSKELSTN........NDIILCDG................VCDRG
lcl|Glyma.01G234300_253-300       ..........CDICGD..AGRE........DLLAICS.................RCSDG
lcl|Glyma.11G008600_251-297       ..........CDICGD..AGRE........DLLAICS.................RCSDG
lcl|Glyma.12G014900_273-322       ..........CDICGD..AGRE........DLLAICS.................RCSDG
lcl|Glyma.11G109800_273-322       ..........CDICGD..AGRE........DLLAICS.................RCSDG
lcl|Glyma.17G155700_20-68         ..........CDICGD..QGFE........EYLAICN.................KCPDG
lcl|Glyma.04G163100_13-61         ..........CDICGA..SGFD........ETIVTCS.................KCNIN
lcl|Glyma.11G043500_25-80         ..........CDTCGA..SGFD........NELVVCH.................KCNEG
lcl|Glyma.06G230000_305-354       ..........CLQCGN..AGFP........ETLVFCN.................KCQVY
lcl|Glyma.09G125400_373-422       ..........CLQCGN..AGFP........ETLVFCN.................KCQVY
lcl|Glyma.10G227100_4-50          ..........CLHCGD..RGFP........ETLVFCT.................ECKAY
lcl|Glyma.20G165200_4-53          ..........CLHCGD..RGFP........ETLVFCS.................QCKAY
lcl|Glyma.04G214800_25-74         ..........CLICGD..KGDL........KSLIYCD.................QCKAC
lcl|Glyma.04G062400_53-97         .............CQA..CGEN........ENLVSCG.................TCTYA
lcl|Glyma.06G063400_53-97         .............CQA..CGEN........ENLVSCG.................TCTYA
lcl|Glyma.12G170900_115-166       ............LCGN..VLDT........EPLVKCEDT...............KCHVS
lcl|Glyma.13G328100_115-166       ............LCGN..VFDT........EPLVKCEDT...............RCHAS
lcl|Glyma.12G071300_116-167       ............LCGS..RLET........ENLVKCDDA...............RCHVW
lcl|Glyma.04G253800_296-345       ..........CHYCGRGDTGRDS.......NRLIVCAS.................CKVA
lcl|Glyma.06G108500_295-345       ..........CHYCGRGDTSRDS.......NRLIVCAS.................CKVV
lcl|Glyma.15G219200_660-708       ..........CEICT...TEEER.......KKLLQCSC.................CGKL
lcl|Glyma.01G238300_516-558       ...........GICGD..GG...........DLICCD..G...............CPST
lcl|Glyma.11G005500_722-764       ...........GICGD..GG...........DLICCD..G...............CPST
lcl|Glyma.01G238200_728-770       ...........GICGD..GG...........DLICCD..G...............CPST
lcl|Glyma.11G005300_720-760       ..........CGICGD..GG...........DLICCD..G...............CPST
lcl|Glyma.02G146500_568-610       ...........GVCGD..GG...........DLICCD..G...............CPST
lcl|Glyma.03G155500_313-355       ...........SLCGK..RG...........DLICCD..T...............CPST
lcl|Glyma.10G044200_547-587       ...........AVCGD..GG...........DLILCN..G...............CPRA
lcl|Glyma.13G131800_545-585       ...........AVCGD..GG...........DLILCN..G...............CPRA
lcl|Glyma.06G046500_300-343       ..........CAICYF..GG...........ELVLCD..R...............CPSS
lcl|Glyma.11G170900_521-564       ..........CSVCHY..GG...........ELILCD..K...............CPSS
lcl|Glyma.03G181800_43-91         .........VCSNCVR..GG...........VLLSCSGKG...............CQRR
lcl|Glyma.11G074400_81-126        ..........CFICFD..GG...........SLVLCDRRG...............CPKA
lcl|Glyma.13G320100_386-430       ...........RLCGM..DG...........TLLCCD..G...............CPAV
lcl|Glyma.02G296200_232-286       ..........CALCDN..GGNVT.....CCDGVCMRSFHAT..............VEAG
lcl|Glyma.18G086000_229-283       ..........CAICDN..GGQLL.....CCDGKCMRSFHAN..............EEDG
lcl|Glyma.13G197800_36-86         ..........CIHCNN..KGEEA.....KEDGLLICSGRG...............CPVA
lcl|Glyma.15G236400_75-123        ..........CIHCDN..KGEEA.......EGVLICGGRG...............CPVA
lcl|Glyma.13G057300_662-708       ..........CVCLGG..R..........VENLFIC..HG...............CQRL
lcl|Glyma.19G029100_661-707       ..........CVCLGG..R..........VENLFIC..HG...............CQRL
lcl|Glyma.02G227900_820-872       ...........GICGQ..TKIEG..TEDGD..LLACI..Q...............CEHK
lcl|Glyma.14G194800_734-787       ..........CGICRQ..TKIEG..TEDGD..LLACI..Q...............CEHK
lcl|Glyma.11G171200_781-832       ...........GICGQ..RKIDGD.DEVGQ..LLPCI..Q...............CEHK
lcl|Glyma.10G062700_503-554       ...........VLCRS..SDFSR..SGFGPRTIIICD..Q...............CEKE
lcl|Glyma.13G147100_501-557       ...........VLCRS..SDFSR..SGFGPRTIIICD..Q...............CEKE
lcl|Glyma.02G058300_166-214       ..........CGSCGGNYNKDE.........FWIGCDI.................CEWW
lcl|Glyma.06G319800_166-214       ..........CGSCGGNYNKDE.........FWIGCDI.................CEWW
lcl|Glyma.07G271400_198-247       ...........GACGDNYGTDE.........FWICCDM.................CERW
lcl|Glyma.17G002600_199-248       ...........GACGDNYGTDE.........FWICCDM.................CERW
lcl|Glyma.11G190000_1281-1329     ..........CHLCRKPYMSDL.........TYICCET.................CQNW
lcl|Glyma.12G084300_1287-1335     ..........CHLCRKPYMSDL.........TYICCET.................CRNW
```

```
lcl|Glyma.12G180700_1293-1341   ..........CRLCHKPYRSDL.........MYICCET..................CKHW
lcl|Glyma.07G056000_373-427     ..........CGICKRIWHHSDG.......GNWVCCDG..................CNVW
lcl|Glyma.16G024900_371-425     ..........CGICKRIWHHSDG.......GNWVCCDG..................CNVW
lcl|Glyma.03G215600_371-425     ..........CGICKKIWHHSDG.......GNWVCCDG..................CNVW
lcl|Glyma.19G212100_374-428     ..........CGICKKIWHHSDG.......GNWVCCDG..................CNVW
lcl|Glyma.04G236500_434-488     ..........CGICKKVWNHSDS.......GSWVRCDG..................CKVW
lcl|Glyma.06G127800_441-495     ..........CGICKKVWNHSDS.......GSWVRCDG..................CKVW
lcl|Glyma.19G066800_376-430     ..........CGICKKIWNYSDS.......GSWVRCDG..................CKVW
lcl|Glyma.01G179800_176-228     ..........CAGCK...............TEIGQGR................FLSCMGG
lcl|Glyma.11G062400_192-244     ..........CAGCK...............TEIGQGR................FLSCMGG
lcl|Glyma.02G059900_158-214     ...........SGCN...............AEIGHGR................FLSCMGG
lcl|Glyma.16G142700_157-213     ...........AGCN...............SEIGHGR................FLSCMGG
lcl|Glyma.12G160000_164-201     ..........CAGCN...............AEISHGR................FLSCMGG
lcl|Glyma.14G077800_125-177     ..........CAGCY...............TEIGYGR................YLNCLNA
lcl|Glyma.17G247700_120-172     ..........CAGCY...............TEIGFGR................YLNCLNA
lcl|Glyma.02G251800_176-228     ..........CGGCN...............QEIMYGN................CLGCMDT
lcl|Glyma.14G065000_141-193     ..........CGGCN...............QEIMYGN................CLGCMDT
lcl|Glyma.02G253700_47-99       ...........KACG...............KTVYPVD................QLS....
lcl|Glyma.18G030900_10-66       ...........KACE...............KTVYPVD................QLSADGT
lcl|Glyma.05G205800_10-66       ...........KACD...............KTVHFVE................GLSADGA
lcl|Glyma.08G012700_10-66       ...........KACD...............KTVHLVE................GLSVDGA
lcl|Glyma.03G013900_10-66       ...........TACD...............KTVYVVD................LLTLEGI
lcl|Glyma.07G073600_10-66       ...........TACD...............KTVYVVD................LLTLEGI
lcl|Glyma.09G261800_10-66       ...........KACD...............KTVYVVD................MLTLEGI
lcl|Glyma.18G230400_10-66       ...........KACD...............KTVYVVD................MLTLEGI
lcl|Glyma.04G179800_24-80       ...........KACE...............KTVYLVD................QLTADNK
lcl|Glyma.05G034700_11-67       ...........TACE...............KKVYWVE................QLTADNK
lcl|Glyma.10G114300_10-49       ..........CKACE...............KAVYLVD................QLTADNK
lcl|Glyma.20G085200_10-49       ..........CKACE...............KAVYLVD................QLTADNK
lcl|Glyma.04G046200_109-164     ...........AGCQ...............KTVYPTE................KVTVNGT
lcl|Glyma.06G047000_109-164     ...........AGCQ...............KTVYPTE................KVTVNGT
lcl|Glyma.17G234700_109-164     ...........AGCQ...............KTVYPTE................KVTVNGT
lcl|Glyma.10G256600_111-167     ...........VCCN...............KTVYPTE................RVTVNGT
lcl|Glyma.06G185000_110-162     ..........CVACK...............KTVYPIE................KVAVDGT
lcl|Glyma.17G092600_110-166     ...........VGCK...............KTVYPIE................KVAVDGK
lcl|Glyma.11G226400_108-164     ...........ATCG...............KTAYPLE................KVTVEGQ
lcl|Glyma.14G062700_108-164     ...........ATCG...............KTAYPLE................KVTVEGQ
lcl|Glyma.14G112700_1508-1555   ..........CCVCRSSSN...........DKINYLL..................ECSRC
lcl|Glyma.13G253900_326-371     .........VCPVSRD..............QGSEENP..............PMLLPCLHV
lcl|Glyma.13G273500_171-204     ...........ELAEP.............VRSME......................CRHI
```

```
                         β8           α14
Human_c-Cbl              .▶ ℓℓ......ℓℓℓℓℓ.....ℓ.ℓ          T...TT
                         20                      30
Human_c-Cbl              .MCTS.....CLTSW.....Q.ESEGQG...........CP...FC.............
consensus                TFCRS.....CIRKWL....ESG............NNTCP...IC.............
lcl|Glyma.09G103000_21-70      .VCRA.....CYEYERKD.................GNQSCP...QCKTRY..........
lcl|Glyma.15G275000_20-69      .VCRA.....CYEYERKD.................GNQSCP...QCKTRY..........
lcl|Glyma.04G067900_40-89      .VCRP.....CYEYERKD.................GNQSCP...QCKTRY..........
lcl|Glyma.06G069600_30-98      .VCRP.....CYEYERKD.................GNQSCP...QCKTRYKRHRGSPRV.
lcl|Glyma.13G202500_20-69      .VCRP.....CYEYERKD.................GNQSCP...QCKTRY..........
lcl|Glyma.02G080900_38-87      .VCRP.....CYEYERRE.................GNQVCP...QCKTRY..........
lcl|Glyma.16G165900_30-96      .VCRP.....CYEYERRE.................GNQVCP...QCKTRYKRIKGSPRV.
lcl|Glyma.05G187300_39-88      .VCRP.....CYEYERRE.................GIQACP...QCNTRY..........
lcl|Glyma.08G145600_39-88      .VCRP.....CYEYERRE.................GNQACP...QCKTRY..........
lcl|Glyma.10G223500_41-90      .VCRP.....CYEYERRE.................GNKICP...QCKTIY..........
lcl|Glyma.02G205800_37-86      .ACRP.....CYEYERRE.................GRQVCP...QCKTRY..........
lcl|Glyma.17G072200_27-95      .ACRP.....CYEYERRE.................GRQVCP...QCKTRYKRLKGSPRV.
lcl|Glyma.04G153700_37-86      .VCRP.....CYEYERRE.................GSQLCP...QCKTRY..........
lcl|Glyma.06G225500_37-86      .VCRP.....CYEYERRE.................GSHLCP...QCKTRY..........
lcl|Glyma.04G142700_37-86      .VCRP.....CYEYERRE.................GTQVCP...QCHTRY..........
lcl|Glyma.06G225400_46-117     .VCRP.....CYEYERRE.................GTQVCP...QCHTRYKRTKGSPRVL
lcl|Glyma.06G316800_40-89      .VCKS.....CYEYERRE.................GNQVCP...QCKTRF..........
lcl|Glyma.09G051100_38-87      .VCRP.....CYEYERSE.................GNQSCP...QCNTRY..........
lcl|Glyma.15G157100_38-87      .VCRP.....CYEYERSE.................GNQSCP...QCNTRY..........
lcl|Glyma.08G088400_32-81      .VCRP.....CYEYERSE.................GNHCCP...QCNTRY..........
lcl|Glyma.08G302500_39-88      .LCHS.....CYEYELKH.................MSQSCP...QCKTAF..........
lcl|Glyma.18G117100_39-88      .LCHS.....CYEYELKN.................VSQSCP...QCKTTF..........
lcl|Glyma.04G063800_9-58       .ICKA.....CFEYEINE.................GRKVCL...RCATPY..........
lcl|Glyma.06G065000_6-58       .ICKA.....CFEYEINE.................GRKVCL...RCATPY..........
lcl|Glyma.05G160000_385-436    .ICKD.....CFEHEINE.................DHRVCM...RCGTP...........
lcl|Glyma.08G117500_6-58       .ICKD.....CFEHEINE.................DHRVCM...RCGTPY..........
lcl|Glyma.12G017600_131-178    .ICRD.....CYKDAAKA.................GDGICP...GCKEPY..........
lcl|Glyma.02G090900_13-60      LKYAHKK...CVQRWCNEKG................DTICE...ICQ.............
lcl|Glyma.18G292200_14-61      LKYAHKK...CVQRWCNEKG................DTICE...ICQ.............
lcl|Glyma.18G242400_64-112     VKYVHKR...CIDQWYNSKGR...............MILCE...ICR.............
lcl|Glyma.02G103800_77-125     IKFVHQD...CLLQWLNHSN................ARQCE...VCK.............
lcl|Glyma.07G215200_80-128     IKFVHQD...CLLQWLNHSN................ARQCE...VCK.............
lcl|Glyma.13G372600_71-120     QKYVHRS...CLDNWRSTKEGF.............AFSHCT...ECR.............
lcl|Glyma.15G001100_69-118     QKYVHRS...CLDNWRSTKEGF.............AFSHCT...ECR.............
lcl|Glyma.11G236400_33-82      SKYVHRE...CLDHWRAIKEGF.............AFAHCT...TCK.............
lcl|Glyma.18G020800_33-82      SKYVHRE...CLDHWRAIKEGF.............AFAHCT...TCK.............
lcl|Glyma.05G182200_29-78      SKYVHRE...CLDHWRAVKEGF.............AFAHCT...TCK.............
lcl|Glyma.05G010700_54-89      QQFVHRS...CLDHW.............................................
lcl|Glyma.17G118400_55-107     QQFVHRS...CLDHWRSVKEGF.............AFSHCT...TCK.............
lcl|Glyma.02G310000_267-316    LALAHQE...CAVKWFSIKGN................RTCD...VCK.............
lcl|Glyma.14G002700_268-317    LALAHQE...CAVKWFSIKGN................RTCD...VCK.............
lcl|Glyma.03G180200_231-280    LALAHQE...CAVKWFSIKGN................RTCD...VCK.............
lcl|Glyma.19G180900_243-292    LALAHQE...CAVKWFSIKGN................RTCD...VCK.............
lcl|Glyma.13G005300_253-302    LALAHQD...CAVKWFSIKGN................KTCD...VCK.............
lcl|Glyma.20G064600_253-302    LALAHQD...CAVKWFSIKGN................KTCD...VCK.............
lcl|Glyma.11G147100_266-315    LALAHQE...CAIKWFSIKGN................KTCD...VCK.............
lcl|Glyma.12G068000_266-315    LALAHQE...CAIKWFSIKGN................KTCD...VCK.............
lcl|Glyma.12G173300_234-283    LALAHQE...CAIKWFSIKGN................KTCD...VCK.............
lcl|Glyma.13G326500_259-308    LALAHQE...CAIKWFSIKGN................KTCD...VCK.............
lcl|Glyma.10G051800_240-289    LSLAHRG...CAVKWFTIKGN................RTCD...VCK.............
lcl|Glyma.13G139200_260-309    LSLAHRE...CVVKWFTIKGN................RTCD...VCK.............
lcl|Glyma.13G213600_217-265    LRLVHEE...CLVKWFSTKGD................KKCD...VCR.............
lcl|Glyma.15G099100_214-262    LRLVHEE...CLIKWFSTKGD................KECD...VCR.............
lcl|Glyma.01G107700_99-151     LGAAHKN...CAEAWFKIKGN................RTCE...IC..............
lcl|Glyma.U027600_104-157      LGAAHKN...CAEAWFKIKGN................RTCE...IC..............
lcl|Glyma.07G142300_108-159    LAAAHKH...CAEAWFKIKGN................RTCE...IC..............
lcl|Glyma.02G253000_84-135     LAAAHKQ...CAEAWFKIKGN................KTCE...IC..............
lcl|Glyma.14G063500_84-135     LAAAHKQ...CAEVWFKIKGN................KTCE...IC..............
lcl|Glyma.13G227900_172-233    LGIAHAH...CAEAWFKLKGN................RSCE...IC..............
lcl|Glyma.15G084600_183-244    LGIAHVH...CAEAWFKLKGN................RLCE...IC..............
lcl|Glyma.04G063700_161-222    LGIAHGH...CAEAWFKLKGN................RLCE...IC..............
lcl|Glyma.06G064900_158-219    PGIAHVH...CALAWFKLKGN................MLCE...IC..............
lcl|Glyma.05G159600_181-243    LGIVHSH...CAEAWFKLKGN................RLCE...IC..............
lcl|Glyma.04G084500_138-184    ...IHEE...CLMKWKRTKGR...............SASCV...ICR.............
lcl|Glyma.06G086100_152-198    ...IHEE...CLIKWKRTKGR...............SASCV...ICR.............
lcl|Glyma.02G232900_1829-1880  ...FHSA...CLYKWFSTSHK...............S.SCP...LCQSPF..........
lcl|Glyma.01G095300_99-145     .FHQD.....CIDLWLR..................SHKTCP...VCRRDL..........
lcl|Glyma.08G270800_99-145     .FHQD.....CIDLWLR..................SHKTCP...VCRRHL..........
lcl|Glyma.01G017200_110-156    .FHQE.....CIDLWLR..................SHKTCP...VCRTDL..........
lcl|Glyma.02G210700_138-184    .FHPD.....CIDAWLV..................NHSTCP...VCRANL..........
lcl|Glyma.14G178400_137-183    .FHSD.....CIDAWLA..................NHSTCP...VCRANL..........
lcl|Glyma.04G098100_128-174    .FHSD.....CIDAWLA..................NHSTCP...VCRANL..........
lcl|Glyma.06G099800_131-177    .FHSE.....CIDAWLA..................NHSTCP...VCRANL..........
lcl|Glyma.10G156500_116-162    .FHPE.....CIDEWLA..................SHTTCP...VCRANL..........
lcl|Glyma.20G231900_117-163    .FHPE.....CIDEWLA..................SHTTCP...VCRANL..........
lcl|Glyma.19G236800_118-164    .FHPE.....CIDEWLG..................SHTTCP...VCRANL..........
lcl|Glyma.03G239900_110-156    .FHPE.....CIDEWLS..................SHTTCP...VCRANL..........
lcl|Glyma.09G140700_126-172    .FHPH.....CIDSWLA..................CHVTCP...VCRANL..........
lcl|Glyma.16G194600_116-162    .FHPH.....CIDSWLT..................SHVTCP...VCRANL..........
lcl|Glyma.09G140800_108-154    .FHAH.....CIDAWLA..................AHVTCP...VCRGEV..........
lcl|Glyma.11G242800_110-156    .FHQH.....CIDTWLP..................SRMTCP...ICRQKL..........
lcl|Glyma.14G037000_80-126     .FHQH.....CIDAWLP..................SHMNCP...ICRQKV..........
lcl|Glyma.06G118400_62-106     .FQQH.....CIDVWSS..................SHINCP...ICQR............
lcl|Glyma.01G017300_141-187    .FHLP.....CIDTWLK..................SHSSCP...LCRASI..........
lcl|Glyma.09G205600_131-177    .FHLP.....CIDTWLK..................SHSSCP...LCRASI..........
lcl|Glyma.01G095600_140-186    .FHAP.....CIDTWLK..................SHSSCP...LCRAGI..........
lcl|Glyma.08G271100_141-187    .FHAP.....CIDTWLK..................SHSSCP...LCRAGI..........
lcl|Glyma.04G128100_143-189    .FHLP.....CIDTWLK..................SHATCP...LCRASV..........
lcl|Glyma.06G310600_135-181    .FHLP.....CIDTWLK..................SHATCP...LCRSSV..........
lcl|Glyma.02G210400_152-198    .FHVP.....CIDTWLR..................SHTNCP...LCRAGI..........
lcl|Glyma.14G177900_153-199    .FHVP.....CIDTWLR..................SHTNCP...LCRAGI..........
lcl|Glyma.08G070100_155-204    .FHLP.....CIDTWLR..................SHTNCP...MCRAPI..........
lcl|Glyma.13G042300_156-201    .FHLP.....CIDTWLR..................SHTNCP...MCRAPI..........
lcl|Glyma.08G177000_180-226    .FHLP.....CIDTWLR..................SHTNCP...MCRAPI..........
lcl|Glyma.15G056000_166-212    .FHLP.....CIDTWLR..................SHTNCP...MCRAPI..........
lcl|Glyma.05G175400_149-200    .FHIP.....CIDTWLR..................SHKNCP...LCRAPVLR........
lcl|Glyma.18G014500_194-240    .FHIS.....CIDTWLR..................SHKSCP...LCRAPI..........
lcl|Glyma.18G014600_153-202    .FHIP.....CIDTWLR..................SHKSCP...LCRAPI..........
lcl|Glyma.11G242500_156-205    .FHVP.....CVDTWLR..................SHKTCP...LCRAPI..........
lcl|Glyma.11G122400_135-179    .FHVD.....CIDAWLR..................SHANCP...LCR.............
lcl|Glyma.12G047300_133-176    .FHVD.....CIDAWLR..................SHANCP...LCRA............
```

```
lcl|Glyma.01G032200_147-191   .FHIE......CIDTWLL..................SNSTCP...LCR.............
lcl|Glyma.02G033200_149-193   .FHIE......CIDTWLL..................SNSTCP...LCR.............
lcl|Glyma.13G071600_172-216   .FHIS......CIDTWLL..................SNSTCP...LCR.............
lcl|Glyma.19G011200_168-212   .FHIS......CIDTWLL..................SNSTCP...LCR.............
lcl|Glyma.08G289200_148-194   .FHID......CIDTWLL..................SNSTCP...LCRGSL..........
lcl|Glyma.18G135300_149-195   .FHID......CIDTWLL..................SNSTCP...LCRGSL..........
lcl|Glyma.05G035800_139-185   .FHMN......CLDMWLL..................SNSTCP...LCRASL..........
lcl|Glyma.17G091600_134-180   .FHMN......CLDTWLL..................SNSTCP...LCRASL..........
lcl|Glyma.13G092000_129-175   .FHME......CIDTWLL..................SHSTCP...LCRATL..........
lcl|Glyma.17G068300_125-171   .FHME......CIDTWLL..................SHSTCP...LCRASL..........
lcl|Glyma.03G103800_137-183   .FHAE......CIDTWLQ..................SNLSCP...LCRSAI..........
lcl|Glyma.07G120300_138-184   .FHAE......CIDTWLQ..................SNLSCP...LCRSTI..........
lcl|Glyma.15G183300_121-167   .FHAE......CIDTWLR..................SKLTCP...LCRSTV..........
lcl|Glyma.01G146500_110-156   .FHID......CIDHWLE..................KHSSCP...ICR.............
lcl|Glyma.09G194100_115-161   .FHID......CIDHWLE..................KHSTCP...ICR.............
lcl|Glyma.04G083400_113-140   ....................................NHSTCP...LCR.............
lcl|Glyma.06G084800_126-176   .FHMN......CIDKWFE..................SHSTCP...LCRRRVE.........
lcl|Glyma.14G146700_132-182   .FHMN......CIDKWLE..................SHSSCP...LCRNSID.........
lcl|Glyma.02G106400_97-139    .FHVE......CVDSWLD..................VHSMCP...LC..............
lcl|Glyma.04G091400_117-163   .FHVE......CVDTWLD..................AHSTCP...LCRYRV..........
lcl|Glyma.03G215500_93-139    .FHAH......CIDTWFG..................SHSKCP...LCRTPV..........
lcl|Glyma.19G212000_90-136    .FHAH......CIDTWIG..................SHSTCP...LCRTPV..........
lcl|Glyma.02G016800_86-132    .FHAH......CVDIWFH..................SHSNCP...LCRTPV..........
lcl|Glyma.07G046400_108-154   .FHVD......CIDMWFH..................SHSTCP...LCRNPV..........
lcl|Glyma.16G014600_107-153   .FHVD......CIDMWFH..................SHSTCP...LCRNPV..........
lcl|Glyma.03G263000_99-148    .FHVA......CIDMWFQ..................SHSTCP...LCRNPV..........
lcl|Glyma.07G245400_93-139    .FHTE......CIDVWFQ..................SHATCP...LCRETV..........
lcl|Glyma.17G028400_95-141    .FHTE......CIDMWFQ..................SHATCP...LCREPV..........
lcl|Glyma.09G042400_118-164   .FHIE......CIDMWFH..................SHDTCP...LCRAPV..........
lcl|Glyma.13G332800_88-134    .FHAS......CIDTWFR..................SHSNCP...LCRAYV..........
lcl|Glyma.06G284600_119-165   .FHVD......CIDTWLD..................SHSTCP...LCRAEV..........
lcl|Glyma.12G121400_124-171   .FHVD......CIDKWLG..................SHSTCP...ICRAEVK.........
lcl|Glyma.12G208600_102-147   .FHVS......CIDTWLS..................SHSTCP...ICRTK...........
lcl|Glyma.13G292600_94-142    .FHVG......CIDTWLA..................SHSTCP...ICRTK...........
lcl|Glyma.09G214600_99-144    .FHVA......CIDMWLS..................SHSSCP...ICRSS...........
lcl|Glyma.16G075300_86-130    .FHVA......CIDAWLY..................SHSNCP...VCR.............
lcl|Glyma.13G108500_112-158   .FHAP......CIDMWLY..................SHFDCP...ICRTPV..........
lcl|Glyma.17G050900_108-154   .FHAP......CIDMWLY..................SHLDCP...ICRTPV..........
lcl|Glyma.18G176200_157-203   .FHAS......CIDLWLS..................NHSNCP...ICRATI..........
lcl|Glyma.02G228800_114-163   .FHME......CIDMWLS..................SHCNCP...ICRAPI..........
lcl|Glyma.01G157400_104-150   .FHVP......CIDTWLG..................SHSSCP...SCRQIL..........
lcl|Glyma.11G087600_104-150   .FHVP......CIDTWLG..................SHSSCP...SCRQVL..........
lcl|Glyma.04G015200_96-142    .FHVS......CIDAWLR..................SHSSCP...SCRQIL..........
lcl|Glyma.06G015200_96-142    .FHVS......CIDAWLR..................SHSSCP...SCRQIL..........
lcl|Glyma.09G196700_100-146   .FHVA......CVDTWLA..................SHSSCP...SCRAPF..........
lcl|Glyma.16G112200_99-145    .FHVA......CVDTWLA..................SHSSCP...SCRAPF..........
lcl|Glyma.04G215600_139-185   .FHVR......CIDKWLS..................SHSSCP...KCRQCL..........
lcl|Glyma.06G150400_141-187   .FHIR......CIDKWLS..................SHSSCP...KCRQCL..........
lcl|Glyma.05G188900_144-190   .FHVC......CIDKWLS..................SHSSCP...KCRQCL..........
lcl|Glyma.08G146600_143-189   .FHVR......CIDKWLS..................SHSSCP...KCRQCL..........
lcl|Glyma.09G275300_113-159   .FHVR......CIDTWLL..................SHSSCP...NCRHSL..........
lcl|Glyma.18G214200_108-154   .FHVR......CIDTWLL..................SHSSCP...NCRHSL..........
lcl|Glyma.07G173600_79-113    .FHVR......CINMWLL..................SHSSCP...NCRHSL..........
lcl|Glyma.06G143100_111-157   .FHVR......CIDTWLL..................SHSSCP...NCRQSL..........
lcl|Glyma.08G088100_109-152   .FHVV......CIDKWLL..................SHSSCP...TCRH............
lcl|Glyma.15G157400_108-152   .FHVD......CIDKWLL..................SHSSCP...TCR.............
lcl|Glyma.04G182100_194-239   .FHVE......CIDEWLR..................LNVNCP...RCRCSV..........
lcl|Glyma.06G183300_233-278   .FHVE......CIDEWLR..................LNVNCP...RCRCSV..........
lcl|Glyma.05G037000_234-279   .FHVE......CIDEWLR..................LNVKCP...RCRCSV..........
lcl|Glyma.17G090200_234-279   .FHVE......CIDEWLR..................LNVKCP...RCRCSV..........
lcl|Glyma.11G191800_129-174   .FHPH......CIDTWLD..................KQVTCP...VCRCS...........
lcl|Glyma.01G151700_511-557   .FHTT......CVDKWLKE.................IHRVCP...LCRGDI..........
lcl|Glyma.09G216900_511-557   .FHTT......CVDKWLKE.................IHRVCP...LCRGDI..........
lcl|Glyma.16G076000_519-565   .FHRT......CIDKWLKE.................IHRVCP...LCRRDI..........
lcl|Glyma.16G107700_522-568   .FHRT......CIDKWLKE.................IHRVCP...LCRGDI..........
lcl|Glyma.13G165500_478-524   .YHMS......CVDKWLKE.................IHGVCP...LCRGNV..........
lcl|Glyma.17G105500_479-525   .YHMS......CVDKWLKE.................IHGVCP...LCRGNV..........
lcl|Glyma.01G217300_329-374   .FHCV......CVDKWLY..................INATCP...LCKYNI..........
lcl|Glyma.11G026000_332-377   .FHCV......CVDKWLY..................INATCP...LCKYNI..........
lcl|Glyma.05G048800_325-370   .FHCA......CVDKWLH..................INATCP...LCKYNI..........
lcl|Glyma.17G130700_323-368   .FHCA......CVDKWLH..................INATCP...LCKYNI..........
lcl|Glyma.09G265600_314-359   .FHCT......CIDKWLL..................INATCP...LCKFNI..........
lcl|Glyma.18G225500_320-365   .FHCT......CIDKWLL..................INATCP...LCKFNI..........
lcl|Glyma.02G085200_305-350   .FHSS......CIVKWLK..................MNATCP...LCKYNI..........
lcl|Glyma.07G170200_304-349   .FHSS......CIVKWLK..................MNATCP...LCKYNI..........
lcl|Glyma.05G238900_292-337   .FHST......CIVKWLK..................MNATCP...LCKYNI..........
lcl|Glyma.08G046000_293-338   .FHST......CIVKWLK..................MNATCP...LCKYNI..........
lcl|Glyma.03G120200_273-318   .FHCE......CIGRWLQ..................TKATCP...LCKFNI..........
lcl|Glyma.19G124800_275-320   .FHCG......CISRWLR..................TKATCP...LCKFNI..........
lcl|Glyma.02G302400_289-335   .FHLK......CVDQWLK..................IISCCP...ICKQ............
lcl|Glyma.14G011800_289-335   .FHLK......CVDQWLK..................ITSCCP...LCKQ............
lcl|Glyma.08G332300_279-325   .FHLK......CVDQWLR..................IISCCP...LCKQ............
lcl|Glyma.18G074600_278-324   .FHLK......CVDQWLR..................IISCCP...LCKQ............
lcl|Glyma.13G361000_365-409   .FHVE......CVDKWLK..................INATCP...LCKNEV..........
lcl|Glyma.15G012900_392-436   .FHVM......CVDKWLK..................INATCP...LCKNEV..........
lcl|Glyma.14G129100_343-387   .FHKD......CVDKWLK..................INALCP...LCKSDV..........
lcl|Glyma.17G203800_375-419   .FHKD......CVDKWLK..................INALCP...LCKSDV..........
lcl|Glyma.01G162900_187-232   .FHLP......CIDKWLFR..................HGSCP...LCRRDL..........
lcl|Glyma.11G080500_187-232   .FHLP......CIDKWLFR..................HGSCP...LCRRDL..........
lcl|Glyma.02G044300_186-231   .FHLP......CIDKWLIK..................HGSCP...LCRRDL..........
lcl|Glyma.04G143800_84-127    .FHLV......CIDKWLVQ..................QGSCP...MCK.............
lcl|Glyma.08G316400_182-225   .FHSV......CIDKWLVQ..................QGSCP...MCR.............
lcl|Glyma.18G096600_201-244   .FHLV......CIDKWLVQ..................RRSCP...MCR.............
lcl|Glyma.02G291200_187-233   .FHLE......CIDKWLVQ..................QGSCP...MCRTYV..........
lcl|Glyma.05G025200_170-216   .FHLI......CVDKWLVK..................NDSCP...VCRQNV..........
lcl|Glyma.17G101800_167-213   .FHLI......CVDKWLVK..................NDSCP...VCRQNV..........
lcl|Glyma.10G124600_688-735   .FHKD......CIDPWLQR..................KTSCP...VCKSSIT.........
lcl|Glyma.20G076700_718-765   .FHKD......CIDPWLQR..................KASCP...VCKSSIT.........
lcl|Glyma.10G217500_232-277   .FHAA......CVDSWLTS.................WRTFCP...VCKRD...........
lcl|Glyma.20G174500_247-292   .FHAA......CVDSWLTS.................WRTFCP...VCKRD...........
lcl|Glyma.11G137400_208-259   .FHAN......CIDPWLRQ..................QGTCP...VCKLRIGSV.......
lcl|Glyma.12G060800_236-287   .FHAN......CIDPWLRQ..................QGTCP...VCKLRIGSV.......
lcl|Glyma.11G220200_212-254   .FHAN......CIDPWLRQ..................QGTCP...VCK.............
```

```
lcl|Glyma.18G037200_212-254   .FHAN......CIDPWLRQ.................QGTCP...VCK.............
lcl|Glyma.02G194400_103-147   .FHLK......CIDPWLR.................LHPTCP...LCRTS...........
lcl|Glyma.10G083100_103-147   .FHLK......CIDPWLR.................LHPTCP...LCRTS...........
lcl|Glyma.03G203400_104-149   .FHLK......CIDPWLR.................LHPTCP...VCRTS...........
lcl|Glyma.10G121100_107-150   .FHRE......CIDRWLQ.................VNLSCP...MCR.............
lcl|Glyma.10G121500_113-160   .FHRD......CIDMWLQ.................LNLTCP...LCRTS...........
lcl|Glyma.03G017500_84-127    .FHLS......CIDMWLR.................KQSTCP...VCR.............
lcl|Glyma.07G078800_84-127    .FHLS......CIDMWLR.................KQSTCP...VCR.............
lcl|Glyma.09G264300_89-135    .FHLS......CIDIWLR.................KQSTCP...VCRLPL..........
lcl|Glyma.18G227700_89-135    .FHLS......CIDIWLR.................KQSTCP...VCRLPL..........
lcl|Glyma.10G202500_86-132    .FHVT......CIDLWLQ.................QNSTCP...VCRISL..........
lcl|Glyma.20G187900_86-132    .FHVT......CIDLWLQ.................QNSTCP...VCRISL..........
lcl|Glyma.09G001600_110-156   .FHMS......CIDLWLA.................THTTCP...LCRFSL..........
lcl|Glyma.05G217000_334-378   .FHAD......CIDEWLR.................LNATCP...LCR.............
lcl|Glyma.08G022900_332-375   .YHAH......CIDHWLK.................LNATCP...LCR.............
lcl|Glyma.07G056200_314-357   .FHAD......CIDEWLR.................INTTCP...VCR.............
lcl|Glyma.16G025100_320-363   .FHAD......CIDEWLR.................INTTCP...VCR.............
lcl|Glyma.19G255700_319-362   .FHAE......CIDEWLR.................MNSTCP...VCR.............
lcl|Glyma.06G127300_326-371   .FHAQ......CIDEWLP.................LNASCP...ICRTS...........
lcl|Glyma.07G062800_119-162   .FHLC......CLDPWLK.................LNGSCP...VCR.............
lcl|Glyma.16G030900_115-158   .FHLC......CLDPWLK.................LNGSCP...VCR.............
lcl|Glyma.09G253500_112-155   .FHLC......CLDSWLK.................LNGSCP...VCR.............
lcl|Glyma.18G239400_89-132    .FHLC......CLDSWLK.................LNGSCP...VCR.............
lcl|Glyma.09G253400_107-153   .FHKD......CVDAWLK.................VKTSCP...ICRNSL..........
lcl|Glyma.02G264800_105-151   .FHCE......CVDKWLA.................NHSNCP...LCRASL..........
lcl|Glyma.14G058000_100-146   .FHCE......CVDKWLT.................NHSNCP...LCRASL..........
lcl|Glyma.11G231400_107-153   .FHCD......CVDKWLT.................HHSSCP...LCRASL..........
lcl|Glyma.18G025700_107-153   .FHCD......CVDKWLT.................HHSSCP...LCRASL..........
lcl|Glyma.11G169500_92-138    .FHSK......CLGMWLS.................AHPSCP...LCRASL..........
lcl|Glyma.18G060400_108-154   .FHSE......CLDMWLS.................GHPSCP...LCRASL..........
lcl|Glyma.01G093800_57-96     .FHKG......CLDRWL.....................VSR...MPR.............
lcl|Glyma.04G153400_57-104    .FHKG......CLDRWVGFE............NA....TCP...LCRGSL..........
lcl|Glyma.04G153500_74-120    .FHRD......CLNTWVGFN............NA...TTCP...LCR.............
lcl|Glyma.13G115900_83-134    .FHRE......CIERWLEHE............HENHIPTCP...LCRAPL..........
lcl|Glyma.17G043900_85-135    .FHRE......CIDRWLEHE............HENHSATCP...ICRAP...........
lcl|Glyma.01G221300_81-127    .FHRG......CLDRWMGYD............QR....TCP...LCRTPF..........
lcl|Glyma.11G022400_87-133    .FHRG......CLDRWMGYD............QR....TCP...LCRTPF..........
lcl|Glyma.05G210000_86-133    .FHRG......CLDRWMGYD............QR....TCP...LCRTAF..........
lcl|Glyma.08G016700_85-132    .FHRG......CLDRWMGYD............QR....TCP...LCRMPF..........
lcl|Glyma.17G159400_72-117    .FHKR......CLDHWMGYD............MR....MCT...LCR.............
lcl|Glyma.02G223600_99-145    .FHKV......CLEKWLDYW............NI....TCP...LCRTPL..........
lcl|Glyma.14G190300_98-144    .FHKV......CLEKWLDYW............NI....TCP...LCRTPL..........
lcl|Glyma.13G009100_96-142    .FHKV......CMEKWLDYW............NI....TCP...LCRTSL..........
lcl|Glyma.05G182000_68-114    .FHKD......CLDQWL..Q............QYC..ATCP...LCRNKV..........
lcl|Glyma.08G139700_68-114    .FHKD......CLDQWL..Q............QYC..ATCP...LCRNKV..........
lcl|Glyma.11G236600_73-119    .FHRD......CLDKWL..Q............QYW..ATCP...LCRKQV..........
lcl|Glyma.18G020400_70-116    .FHRD......CLDKWL..Q............QYW..ATCP...LCRKQV..........
lcl|Glyma.05G218700_107-152   .FHIS......CISNWL..Q............SN...STCP...LCRCSI..........
lcl|Glyma.08G024800_108-153   .FHIS......CICNWL..Q............SN...STCP...LCRCSI..........
lcl|Glyma.13G002800_104-153   .FHLE......CIHHWL..Q............SN...STCP...LCRCSI..........
lcl|Glyma.20G066300_104-153   .FHFE......CIHHWL..Q............SN...STCP...LCRCSI..........
lcl|Glyma.10G007200_117-163   .FHID......CIDVWL..Q............NN...AHCP...LCRRTV..........
lcl|Glyma.20G088900_139-185   .FHID......CIDVWL..Q............NN...AYCP...LCRRTV..........
lcl|Glyma.10G187100_136-182   .FHID......CIDVWL..Q............SN...ANCP...LCRTSI..........
lcl|Glyma.20G203400_135-181   .FHID......CIDVWL..Q............SN...ANCP...LCRTTI..........
lcl|Glyma.19G163600_128-173   .FHLH......CIDIWL..Q............TN...ANCP...LCRSSI..........
lcl|Glyma.11G243400_93-139    .FHRG......CLDKWFEFD............NKH..STCP...LCRS............
lcl|Glyma.18G013800_88-132    .FHRG......CLDKW.FD.............NKH..TTCP...LCRS............
lcl|Glyma.13G232800_104-150   .FHVD......CVDKWF...............NSN..TTCP...ICRTVV..........
lcl|Glyma.13G232800_105-147   .FHVD......CVDKWF...............NSN..TTCP...ICR.............
lcl|Glyma.15G079900_101-151   .FHAD......CVDKWF...............NSN..TTCP...ICRTVVD.........
lcl|Glyma.04G021200_74-120    .FHVH......CADTWLS.................KHPLCP...VCRTKL..........
lcl|Glyma.06G021400_73-119    .FHVQ......CADTWLS.................KHPICP...VCRTKL..........
lcl|Glyma.14G221800_70-116    .FHLE......CADTWLS.................KHPLCP...LCRAKL..........
lcl|Glyma.17G260700_70-116    .FHLE......CADTWLS.................EHPLCP...LCRAKL..........
lcl|Glyma.04G181300_83-127    .FHVQ......CIDSWIL.................QTPVCP...ICRT............
lcl|Glyma.06G183600_83-127    .FHVQ......CIDSWIL.................QTPVCP...ICRT............
lcl|Glyma.13G170300_69-114    .FHAQ......CVDTWLL.................KTPICP...ICRCN...........
lcl|Glyma.19G010400_71-116    .FHAQ......CVDAWLL.................KTPICP...TCRCN...........
lcl|Glyma.13G092100_125-170   .FHRT......CVDTWLL.................KVAACP...TCRTPV..........
lcl|Glyma.17G068200_100-145   .FHRR......CVDTWLL.................KVAACP...TCRTPV..........
lcl|Glyma.10G244900_121-165   .YHSA......CLLRWLA.................SHPHCP...YCRTPV..........
lcl|Glyma.20G149700_120-164   .YHSA......CLLPWLA.................AHPHCP...YCRTPV..........
lcl|Glyma.01G048700_113-166   .FHVN......CIEKWLGM.................HGSCP...VCRYEMPVEEI.....
lcl|Glyma.02G108300_190-234   .FHGN......CIEKWLGM.................HGSCP...VCRYE...........
lcl|Glyma.02G071200_226-269   .FHGD......CIVSWLKL.................HGSCP...VCR.............
lcl|Glyma.16G152400_225-267   .FHGD......CIVSWLKL.................HGSCP...VCR.............
lcl|Glyma.18G000200_236-278   .FHSG......CILPWLEL.................HSSCP...VCR.............
lcl|Glyma.02G161600_186-230   .YHSD......CIVPWLVQ.................HNSCP...VCRQEL..........
lcl|Glyma.10G101400_186-230   .YHSD......CIVPWLVQ.................HNSCP...VCRQEL..........
lcl|Glyma.02G276700_252-296   .YHSD......CIVPWLVQ.................HNSCP...VCRVEL..........
lcl|Glyma.14G039000_202-246   .YHSD......CIVPWLVQ.................HNSCP...VCRVEL..........
lcl|Glyma.13G072800_206-250   .YHSD......CIVPWLVL.................HNSCP...VCRVEL..........
lcl|Glyma.13G072900_126-169   .YHSD......CIVPWLVH.................HNSCP...VCR.............
lcl|Glyma.04G251600_220-265   .YHSD......CIVPWLRL.................HNSCP...VCRHEV..........
lcl|Glyma.08G185400_202-246   .YHSD......CIIPWLRM.................HNTCP...VCRYEL..........
lcl|Glyma.15G047300_199-243   .YHSD......CIIPWLRM.................HNTCP...VCRYEL..........
lcl|Glyma.09G165200_203-248   .YHAD......CILPWLEL.................HNSCP...VCRYEL..........
lcl|Glyma.16G213500_202-247   .YHAD......CILPWLEL.................HNSCP...VCRYEL..........
lcl|Glyma.10G285300_179-224   .YHGD......CLIPWLRL.................HNSCP...VCRYEL..........
lcl|Glyma.20G103700_178-223   .YHGD......CLIPWLRL.................HNSCP...VCRYEL..........
lcl|Glyma.02G249100_188-232   .YHAE......CILPWLAI.................KNSCP...VCRHEL..........
lcl|Glyma.14G067300_187-231   .YHAE......CILPWLAI.................KNSCP...VCRHEL..........
lcl|Glyma.11G220400_185-229   .YHPE......CILPWLAL.................HNSCP...VCRHEL..........
lcl|Glyma.18G037000_174-209   .YHPE......CILPWLAL.................HNSCP...VCR.............
lcl|Glyma.07G134600_158-202   .YHSE......CIVPWLSV.................RNSCP...VCRHEV..........
lcl|Glyma.18G184200_159-203   .YHSE......CIVPWLSV.................RNSCP...VCRHEV..........
lcl|Glyma.11G137300_184-228   .YHSD......CILPWLSM.................RNSCP...VCRHEL..........
lcl|Glyma.12G060700_182-226   .YHSE......CILPWLSM.................RNSCP...VCRHEL..........
lcl|Glyma.13G338400_153-197   .YHSD......CILPWLSM.................RNSCP...VCRHEL..........
lcl|Glyma.15G036100_152-197   .YHSD......CILPWLSM.................RNSCP...VCRHEL..........
lcl|Glyma.10G286700_262-306   .YHGD......CIVPWLSS.................RNSCP...VCRYEL..........
lcl|Glyma.20G102400_259-303   .YHGD......CIVPWLSS.................RNSCP...VCRFEL..........
```

```
lcl|Glyma.05G017500_280-324    .YHED.....CIVPWLGI................RNTCP...VCRYEF..........
lcl|Glyma.09G271200_369-413    .YHIN.....CILPWLSA................RNSCP...LCRYEL..........
lcl|Glyma.18G218200_373-417    .YHNN.....CILPWLSA................RNSCP...LCRYEL..........
lcl|Glyma.08G158300_95-136     .YHSD.....CITPWLEL................HASCP...LCR.............
lcl|Glyma.15G268100_109-150    .YHSD.....CITPWIEL................NSSCP...LCR.............
lcl|Glyma.08G034000_52-100     .YHSN.....CITLWLEH................CNSCP...LCRCHI..........
lcl|Glyma.06G272400_167-215    .FHED.....CIVPWLTS................KGQCP...VCRFVI..........
lcl|Glyma.12G131800_171-215    .FHED.....CIVPWLTS................KGQCP...VCRFVI..........
lcl|Glyma.06G274300_168-216    .FHED.....CIVPWLTS................KGQCP...VCRFVI..........
lcl|Glyma.17G213300_14-59      .FHED.....CIVPWLTS................KGQCP...VCRFLI..........
lcl|Glyma.18G103300_98-136     .FHKD.....CIVPWLIK................MGNQS...................
lcl|Glyma.12G223700_140-184    .FHSD.....CIDHWLQK................KLTCP...ICR.............
lcl|Glyma.13G277600_139-186    .FHSD.....CIDHWLQK................KLTCP...ICRSCI..........
lcl|Glyma.12G151700_161-205    .FHCD.....CIKHWLEE................KPSCP...ICRH............
lcl|Glyma.13G211100_115-163    .FHLS.....CVNSWLLGG...........G...KTTCP...VCRKDL..........
lcl|Glyma.17G211800_21-68      .FHSS.....CINSWLLRG...............KTTCP...VCRKEL..........
lcl|Glyma.10G047200_194-237    .FHWM.....CILPWLGK................RNTCP...CCR.............
lcl|Glyma.13G135100_190-233    .FHWM.....CILPWLGK................RNTCP...CCR.............
lcl|Glyma.08G148600_91-141     .FHEH.....CITRWLNM................NNTCP...LCRRTI..........
lcl|Glyma.12G223800_101-147    .FHSY.....CIEHWLKD................NATCP...VCRNCL..........
lcl|Glyma.09G096300_141-186    .FHDR.....CLKPWLEN................NSYCP...CCRTTI..........
lcl|Glyma.15G201800_154-199    .FHDR.....CLKPWLEN................NSHCP...CCRTTI..........
lcl|Glyma.10G259300_189-234    .FHSV.....CLDPWIRC................CGDCP...YCRRCI..........
lcl|Glyma.20G131600_187-232    .FHSV.....CLDPWIRC................CGDCP...YCRRSI..........
lcl|Glyma.17G172100_265-310    .YHRH.....CIRQWLLC................RNFCP...VCKRV...........
lcl|Glyma.17G172200_262-307    .YHRH.....CIRQWLLC................RNFCP...VCKRV...........
lcl|Glyma.03G110700_123-171    .FHSE.....CIIRW...................WKWLQ...NSNK............
lcl|Glyma.10G289100_82-127     .FHLN.....CIEQWILRN...............QFSCP...LCR.............
lcl|Glyma.20G099800_77-122     .FHLN.....CIDQWILRN...............QFSCP...LCR.............
lcl|Glyma.16G006200_74-120     .FHTS.....CIYKWLAS................HFGCP...LCRTQI..........
lcl|Glyma.10G142800_16-53      .FHLH.....CILKWVNSQ...........T..SQAHCP...MCRREW..........
lcl|Glyma.20G091400_16-53      .FHLH.....CILKWVNSQ...........T..SQAHCP...MCRREW..........
lcl|Glyma.13G265400_16-53      .FHLH.....CILKWVHSQ...........T..SQAHCP...MCRREW..........
lcl|Glyma.06G303600_75-104     .FHFH.....CISRWLKTR................QVCP...L...............
lcl|Glyma.01G143600_200-252    .FHHQ.....CIVKWLQT................SHTCP...LCR.............
lcl|Glyma.17G108900_203-255    .FHHQ.....CIVKWLQT................SHTCP...LCR.............
lcl|Glyma.20G044300_97-142     .FHLK.....CIVSWLQT................SHVCP...LCRYPL..........
lcl|Glyma.20G044600_112-155    .FHHQ.....CIVTWLQN................SHMCP...LCR.............
lcl|Glyma.09G257100_165-208    .FHLS.....CILEWME................RSDSCP...ICDQE...........
lcl|Glyma.18G235300_138-182    .FHLS.....CILEWME................RSDSCP...ICDQKL..........
lcl|Glyma.08G016300_162-207    .FHLA.....CILEWME................RSETCP...VCDQDL..........
lcl|Glyma.08G251300_174-216    .FHLG.....CIYEWME................RSDSCP...VC..............
lcl|Glyma.18G274000_173-215    .FHLC.....CIYEWME................RSDNCP...VC..............
lcl|Glyma.13G052000_177-221    .FHLG.....CIYEWME................RSDNCP...VCGKVL..........
lcl|Glyma.19G034700_177-219    .FHLG.....CIYEWME................RSDNCP...VCGK............
lcl|Glyma.13G206800_34-78      .YHLH.....CIIEWSQ................RSKECP...ICWQSL..........
lcl|Glyma.15G005300_166-208    .FHCS.....CISKWAD................SS..CP...VCR.............
lcl|Glyma.01G019300_126-171    .FHFP.....CI......V...........KKHPIVTCP...VCNTSW..........
lcl|Glyma.09G203400_19-64      .FHFP.....CI......V...........KKHPIVTCP...VCNTSW..........
lcl|Glyma.01G097900_111-159    .FHFP.....CIAAH...V...........KKQRLVTCP...VCNANW..........
lcl|Glyma.08G273400_110-158    .FHFP.....CLAGH...V...........KKHRMVTCP...VCNANW..........
lcl|Glyma.02G218200_86-131     .FHFP.....CVVSV...S...........HARTNV.CP...VC..............
lcl|Glyma.14G185600_109-152    .FHFP.....CVVS................HAHIHV.CP...VC..............
lcl|Glyma.09G017200_92-144     .FHFA.....CISS....N...........VRHGSVTCP...ICRAHW..........
lcl|Glyma.15G122900_92-144     .FHFA.....CISS....N...........VRHGSVTCP...ICRAHW..........
lcl|Glyma.07G270000_80-128     .FHFA.....CISS....N...........VRHGNVTCP...ICR.............
lcl|Glyma.01G050400_262-297    .FHAE.....CLEQATPKT...........RKSDPP.CP...VC..............
lcl|Glyma.02G109200_262-297    .FHAE.....CLEQTTPKT...........QKSDPP.CP...VC..............
lcl|Glyma.12G235700_273-332    .YHAE.....CLESLTSEV...........NKYDPS.CP...VC..............
lcl|Glyma.13G201200_274-333    .YHAE.....CLESLTSEV...........NKYDPS.CP...VC..............
lcl|Glyma.07G049700_1718-1767  .IHLQ.....CEVSEIEGS...........SKTSSSGCP...VC..............
lcl|Glyma.16G018400_1710-1759  .IHLQ.....CEVSEIEES...........SKTSSSGCP...VC..............
lcl|Glyma.01G228500_290-332    .FHVH.....CLRSWLER................QHTCP...TCRALV..........
lcl|Glyma.11G011300_290-332    .FHVH.....CLRSWLER................QHTCP...TCRALV..........
lcl|Glyma.04G130500_337-383    .FHLA.....CLRSWLDQG...........LT.EMYTCP...TCRKPL..........
lcl|Glyma.06G316600_337-383    .FHLA.....CLRSWLDQG...........LA.EMYTCP...TCRKPL..........
lcl|Glyma.14G105900_232-269    .FHPP.....CLKPWL....................FYP...LC..............
lcl|Glyma.17G220500_232-277    .FHPP.....CLKPWLDE................HNSCP...ICRHEL..........
lcl|Glyma.16G032000_12-61      .FHEL.....CLQQWFEYS...........SKGKKHTCP...ICK.............
lcl|Glyma.03G104400_860-896    .FHLR.....CLG...DN................EKECP...EC..............
lcl|Glyma.07G119500_861-897    .FHLR.....CLG...DN................EKECP...QC..............
lcl|Glyma.12G116100_524-572    .FHSG.....CLQRWMDI................KMECP...TCRGPL..........
lcl|Glyma.12G205300_519-567    .FHSG.....CLQRWMDI................KMECP...TCRRPL..........
lcl|Glyma.06G290800_524-572    .FHSG.....CLQRWMDI................KMECP...TCRRPL..........
lcl|Glyma.13G295600_507-555    .FHSG.....CLQRWMDI................KMECP...TCRRPL..........
lcl|Glyma.01G195200_69-131     TYERS.....NILKWF...NLG............HFTCP...TTMQELWDD.SVTPNT
lcl|Glyma.11G046500_134-196    TYERS.....NILKWF...NLG............HFTCP...TTMQELWDD.SVTPNT
lcl|Glyma.03G202600_12-76      TYDRE.....SIETWL...FSKK...........NTTCP...MTKQPLIDYTDLTPNH
lcl|Glyma.10G262600_10-74      TYDRE.....SIETWL...FSKK...........NTTCP...ITKQPLIDYTDLTPNH
lcl|Glyma.19G200200_10-74      TYDRE.....SIETWL...FSKK...........NTTCP...ITKLPLIDYTDLTPNH
lcl|Glyma.04G212300_16-53      TYDRS.....SIEKWF...STG............NLTCP...V...............
lcl|Glyma.06G154100_14-76      TYDRS.....SIEKWF...SAG............NLTCP...VTMQKLHDP.SIVPNH
lcl|Glyma.05G192100_12-74      TYDRS.....NIEKWL...ALG............NLTCP...VTMQKLHDP.SIVPNH
lcl|Glyma.08G000400_12-74      TYDRS.....NIEKWL...AQG............NLTCP...VTMQKLHDP.SIVPNH
lcl|Glyma.05G189600_16-52      TYDRS.....SIQRWL...DNG............NNTCP...................
lcl|Glyma.08G147200_16-52      TYDRS.....SIQRWL...DNG............NNTCP...................
lcl|Glyma.07G106000_30-93      TYDRE.....SVERWF...DEG............NITCP...VTNQVVRNF.DMIPNH
lcl|Glyma.15G064800_12-50      TFDRS.....SIQRWL...DAG............HRTCP...I...............
lcl|Glyma.16G048700_180-229    IFCFP.....CILQYL...LMGEEDHKGDS....WKRCP...LC..............
lcl|Glyma.19G103000_221-270    IFCFP.....CILQYL...LMGEEDHKGDS....WKRCP...LC..............
lcl|Glyma.05G237500_1120-1165  YMHSA.....CFQAY....TCNHYT............CP...ICSKSL..........
lcl|Glyma.08G044700_1118-1163  YMHSA.....CFQAY....TCSHYT............CP...ICSKSL..........
lcl|Glyma.07G093700_1126-1171  YMHSS.....CFQAY....TCSHYT............CP...ICSKSL..........
lcl|Glyma.09G182600_1122-1167  YMHSS.....CFQAY....TCSHYT............CP...ICSKSL..........
lcl|Glyma.09G115100_1126-1168  FMHSA.....CFQAY....TCSHYI............CP...IC..............
lcl|Glyma.17G096900_1156-1201  VMHST.....CFQEY....TCFNYT............CP...ICSKSL..........
lcl|Glyma.14G130700_200-247    TIHKS.....CLNEMR...EHFQYA............CP...LCSKSV..........
lcl|Glyma.17G202700_200-247    TIHKS.....CLNEMR...EHFQYS............CP...LCLKSV..........
lcl|Glyma.06G074300_199-246    TIHKS.....CLKEMR...EHFQYA............CP...LCLKSV..........
lcl|Glyma.06G116800_4-50       SVCEA.....CLAELP...QRYQDTI..........RCP...AC..............
lcl|Glyma.10G228500_5-52       SVCEA.....CLAELP...QRYQDTI..........RCP...ACSQ............
lcl|Glyma.08G241700_44-77      TLCKN.....CILGLQ..........................................
lcl|Glyma.04G134700_4-52       GFCRE.....CLSRMF...SASSDAT.........LACP...RCR.............
```

```
lcl|Glyma.11G157400_5-54        GFCRE......CLSRMF....SASSDAT.........LACP...RCRHV..........
lcl|Glyma.04G224200_401-445     MFCEE......CVSEWF....ERER.............TCP...LCRALVK........
lcl|Glyma.06G140600_410-454     MFCEE......CVSEWF....ERER.............TCP...LCRALVK........
lcl|Glyma.18G217900_377-421     IFCED......CVSEWF....ERER.............TCP...LCRALVK........
lcl|Glyma.08G354400_21-65       MCCFW......CVYNSM....SCLRES...........QCP...VCR............
lcl|Glyma.18G171400_21-65       ICCFW......CVYNSM....NCLRES...........QCP...VCR............
lcl|Glyma.15G237300_55-95       TICSG......CKPRVH....N................RCP...TCRHEL.........
lcl|Glyma.13G198800_100-140     TICSG......CKPRVH....N................RCP...TCRHEL.........
lcl|Glyma.04G235700_61-101      TLCST......CKTRVH....N................RCP...TCRQEL.........
lcl|Glyma.06G129000_62-102      TLCST......CKTRVH....N................RCP...TCRQEL.........
lcl|Glyma.03G257700_48-88       TLCST......CKTRVH....N................RCP...TCRQEL.........
lcl|Glyma.13G035400_631-678     .FCPSKYYHVRCLSSK.....QLKSYGNCW......Y..CP...SC.............
lcl|Glyma.14G156600_631-678     .FCPSKYYHVSCLSSK.....QLKSYGHCW......Y..CP...SC.............
lcl|Glyma.12G192700_673-714     .FCKA......CLIDF.....S.ASLGRVS.........CP...TCSK...........
lcl|Glyma.13G309700_670-711     .FCKA......CLIDF.....S.SSLGRVS.........CP...TCSK...........
lcl|Glyma.07G148900_159-201     .MCMK......CYEDW.....H..ARSQS..........CP...FCR............
lcl|Glyma.18G200100_157-199     .MCMK......CYEDW.....H..ARSQS..........CP...FCR............
lcl|Glyma.03G060000_142-184     .LCMK......CYRNW.....H..ARSQS..........CP...FCR............
lcl|Glyma.07G195400_147-189     .MCLK......CYREW.....R..TISQS..........CP...FCR............
lcl|Glyma.13G180900_147-189     .MCLK......CYREW.....R..TISQS..........CP...FCR............
lcl|Glyma.13G240800_150-198     .MCLT......CYHEW.....R..TRSQS..........CP...FCRNSLKRV......
lcl|Glyma.15G072700_147-189     .MCLK......CYHEW.....R..TRSQS..........CP...FCR............
lcl|Glyma.18G063500_143-185     .MCLK......CYRDW.....C..QRSQS..........CP...FCR............
lcl|Glyma.05G144300_156-204     .MCIK......CYRKW.....N..TRSES..........CP...FCRGSLRRV......
lcl|Glyma.08G100700_156-204     .MCIK......CYRKW.....N..TRSES..........CP...FCRGSLRRV......
lcl|Glyma.11G246100_158-206     .MCIK......CYRKW.....N..RKSES..........CP...FCRGSLRRV......
lcl|Glyma.18G011100_158-206     .MCIK......CYRKW.....N..RKSES..........CP...FCRGSLRRV......
lcl|Glyma.05G044700_154-201     .MCIN......CYSDG.....N..TRSES..........CP...LCRGSIKS.......
lcl|Glyma.11G246300_78-119      .LCIS......CFHDW.....Y..MRSES..........CP...FCR............
lcl|Glyma.18G010900_145-192     .LCIS......CFHDW.....Y..MRSES..........CP...FCRGSLKRI......
lcl|Glyma.02G309100_117-162     .LCLR......CFEKW.....VGQGK.RT..........CA...NCRAQI.........
lcl|Glyma.14G003700_117-162     .FCLR......CFEKW.....IGQGK.RT..........CA...NCRAQI.........
lcl|Glyma.12G001300_129-175     .FCLK......CFEKW.....VRQGK.RN..........CA...KCRQII.........
lcl|Glyma.05G198100_138-187     .FCNG......CFSEW.....LRRSKERHS......AVLCP...QCRAVV.........
lcl|Glyma.04G165900_213-256     .FCCS......CLFQS.....MDR............GNKCP...LCRT...........
lcl|Glyma.06G196600_193-236     .FCRS......CLFQS.....MDR............GNRCP...LCRT...........
lcl|Glyma.16G063400_170-217     .FCRK......CLRSA.....ADKC...........GKKCP...KCRQLIS........
lcl|Glyma.19G082000_171-218     .FCRK......CLRSA.....ADKC...........GKKCP...KCRQLIS........
lcl|Glyma.12G180800_764-813     .LCRE......CLLSS.....WRNAT..........SGLCP...VCRKTISR.......
lcl|Glyma.13G320000_790-839     .LCRE......CLLSS.....WRNAT..........SGLCP...VCRKTISR.......
lcl|Glyma.20G087900_859-904     .FCRE......CLFSC.....WGTSV..........GGKCP...ICRQ...........
lcl|Glyma.02G149800_290-333     CCCTT......CSSHL.....T................NCP...LCRRQIEKV......
lcl|Glyma.10G024100_292-330     CCCTA......CSSHL.....T................NCP...LCRRQI.........
lcl|Glyma.03G080300_332-382     VCCQG......CAISV.....EREVA...........PKCP...VCRQEIRDS......
lcl|Glyma.16G008300_327-377     VCCQG......CAISV.....EREVA...........PKCP...VCRQEIRDS......
lcl|Glyma.18G149800_331-381     VCCQG......CAISV.....EREVA...........PKCP...VCRQEIRDS......
lcl|Glyma.04G043900_344-393     ATCYD......CAQRI.....LDEES...........IVCP...ICRRLIHK.......
lcl|Glyma.06G044100_357-407     ATCYD......CAERI.....VDGES...........KVCP...ICRRLIHKV......
lcl|Glyma.07G150500_385-433     VACFA......CGTRI.....AEAAG............TCP...VCRRNMKKV......
lcl|Glyma.18G201500_386-434     VACFA......CGTRI.....AEAAG............TCP...VCRRNMKKV......
lcl|Glyma.10G181100_405-450     AGCMS......CLNEV.....KSKK............WGCP...VCRAKIDQV......
lcl|Glyma.20G209400_406-451     AGCMS......CLNEV.....KSKK............WGCP...VCRAKIDQV......
lcl|Glyma.03G180500_289-337     CMCSG......CAKDS.....RFQTD............RCS...ICRQPVER.......
lcl|Glyma.19G181100_312-356     CMCSG......CAKVL.....RFQTD............RCP...ICRQPIER.......
lcl|Glyma.10G052000_291-337     CMCSG......CAKVL.....RFQTN............RCP...ICRQPVER.......
lcl|Glyma.13G139400_291-337     CMCSG......CAKVL.....RFQTN............RCP...ICRQPVER.......
lcl|Glyma.13G326700_239-283     CMCSG......CANLL.....KVHTA............NCP...ICRYPVER.......
lcl|Glyma.07G257200_279-325     CMCGD......CAKAL.....RLQSN............KCP...ICRQPIEE.......
lcl|Glyma.17G016900_219-265     CMCGD......CAKAL.....RPQSN............KCL...ICRQPIEE.......
lcl|Glyma.13G208500_314-360     CMCSE......CANAH.....RLQSN............KCP...ICRQSIEE.......
lcl|Glyma.15G104300_316-362     CMCSE......CANAL.....RQQSN............KCP...ICRQPIEE.......
lcl|Glyma.09G048200_866-915     CTCLK......CANEL.....QWNSG............KCP...ICRAKIVDV......
lcl|Glyma.15G155500_861-910     CTCLK......CANEL.....QWNSG............KCP...ICRAKIEDV......
lcl|Glyma.10G198600_793-842     CTCSK......CANEL.....IRGGG............KCP...LCRAPILEV......
lcl|Glyma.20G191400_792-841     CTCSK......CANEL.....IRGGG............KCP...LCRAPIVEV......
lcl|Glyma.11G191900_808-850     CTCSK......CANEL.....LQSRR............KCP...MCQAPV.........
lcl|Glyma.12G082400_798-847     CTCSK......CANEL.....LQSRR............NCP...MCQAPVVEV......
lcl|Glyma.12G179300_740-789     CTCSK......CANDL.....LQSRR............KCP...MCQAPVVEV......
lcl|Glyma.13G321300_793-842     CTCSK......CANDL.....LQSRR............KCP...MCQAPVVEV......
lcl|Glyma.13G212200_825-875     VVCTT......CNDLH.....EKQGM...........QDCP...SCRSPIQRR......
lcl|Glyma.15G100500_827-877     VVCTT......CNELH.....EKQGM...........QDCP...SCRSPIQRR......
lcl|Glyma.17G020500_819-869     VVCTP......CNELH.....EKQGM...........QDCP...SCRSPIQQR......
lcl|Glyma.07G253800_821-871     VVCKT......CNELH.....EKQGM...........QDCP...SCRSPIQQR......
lcl|Glyma.02G150400_776-826     VVCPE......CNELH.....EKQGM...........KECP...SCRAPIQRR......
lcl|Glyma.05G112600_659-709     VMCAS......CSDEY.....GRKGK...........AICP...CCRVQIQQR......
lcl|Glyma.17G154400_621-671     VMCAS......CSDEY.....GRKGK...........ATCP...CCRVQIQQR......
lcl|Glyma.11G042600_620-669     VMCAR......CGKEY.....GKKGK...........AVCP...CCRVPIEER......
lcl|Glyma.04G009100_1020-1065   CLCKS......CSLAC.....................SECP...ICRTNITDR......
lcl|Glyma.06G009000_1020-1065   CLCKS......CSLAC.....................SECP...ICRTSITDR......
lcl|Glyma.16G098500_18-63       CLCKS......CSLAC.....................SECP...ICRTSITDR......
lcl|Glyma.07G159000_32-77       CLCKS......CSLAC.....................FEGP...ICRTSITDR......
lcl|Glyma.11G112700_1028-1064   CLCKS......CSLAC.....................SECP...LCRT...........
lcl|Glyma.12G038600_1038-1083   CLCKS......CSLAC.....................SECP...ICRTNISDR......
lcl|Glyma.05G013200_415-460     VLCST......CSQKR.....................KKCP...ICRDSIAER......
lcl|Glyma.17G121200_415-460     VLCST......CSEKC.....................KKCP...ICRDSIAER......
lcl|Glyma.10G204900_433-478     ILCST......CCEKC.....................KRCP...VCRGPIEER......
lcl|Glyma.20G185600_422-467     ILCST......CCEKC.....................KRCP...VCRGSIEER......
lcl|Glyma.01G029500_286-325     CLCKD......CEGFI.....................NVCP...VCQ............
lcl|Glyma.02G035800_286-325     CLCKD......CEGFI.....................NVCP...ICQ............
lcl|Glyma.14G083900_301-342     CLCKD......CESKL.....................SFCP...LCQSS..........
lcl|Glyma.17G241100_292-333     CLCKD......CESKL.....................SFCP...LCQSS..........
lcl|Glyma.08G170400_204-247     CLCTA......CGSSL.....................HICP...ICKSFKTAS......
lcl|Glyma.15G256500_288-331     CLCTV......CGSSL.....................HICP...ICKSYKTAS......
lcl|Glyma.09G019900_261-297     CLCTM......CGSTV.....................RNCP...IC.............
lcl|Glyma.15G126400_264-300     CLCTM......CGTTI.....................RNCP...IC.............
lcl|Glyma.01G203300_274-316     CICAE......CDTHF.....................RACP...VCLTVKNST......
lcl|Glyma.11G039700_281-323     CICAE......CDGHF.....................RACP...VCLTVKNST......
lcl|Glyma.05G068400_302-344     CVCTE......CDAHF.....................RACP...VCLTPKNST......
lcl|Glyma.17G150900_309-344     CICTE......CDAHF.....................RACP...VC.............
lcl|Glyma.09G282200_4713-4753   .LCRR......CSS...........AV..........SRCP...FCRLQVTK.......
lcl|Glyma.20G003400_4712-4751   .LCRR......CSS...........AV..........SRCP...FCRLQVTK.......
lcl|Glyma.04G220000_1217-1265   .SCYG......CITRH.....LL.NC...........QRCF...FCNATVTDV......
lcl|Glyma.06G145900_1217-1265   .SCYG......CITRH.....LL.NC...........QRCF...FCNATVTDV......
```

```
lcl|Glyma.08G358300_319-375     .MCAH......CTLAL.....CCHKK.LDPATTGLSGPVCP...FCRGSI..........
lcl|Glyma.18G176600_318-374     .MCAH......CTLAL.....CCHKK.PDPATAGLSGPVCP...FCRGTI..........
lcl|Glyma.12G063600_316-372     .MCAQ......CTLAL.....CCHNK.PNPSTSRVIPPVCP...FCRSSI..........
lcl|Glyma.11G140000_316-372     .MCAQ......CTLAL.....CCHNK.PNPCTSRVIPPVCP...FCRSTI..........
lcl|Glyma.13G335300_323-381     .MCAQ......CTLAL.....CCHNK.PNPATACLTPPVCP...FCRSTITR........
lcl|Glyma.15G039100_316-374     .MCAQ......CTLAL.....CCHNK.PNPATACLTPPVCP...FCRSTITR........
lcl|Glyma.08G315500_281-340     .MCAH......CIMRL.....CQKKSDIDAPRSSNSKPVCP...FCRGDIVR........
lcl|Glyma.03G079200_225-269     .FCRT......CSREI.....WVSRG............NCP...LCNNLILEI.......
lcl|Glyma.16G094800_214-258     .FCRM......CSREI.....WVSRG............NCP...LCNNLILEI.......
lcl|Glyma.08G350900_306-345     .FCRM......CSREL.....MVSRG............NCP...RCN.............
lcl|Glyma.18G165000_306-353     .FCRM......CSREL.....MVSRG............NCP...LCNNFILEI.......
lcl|Glyma.07G264100_155-203     .FCRV......CSREL.....WLNRG............TCP...LCNRSILEI.......
lcl|Glyma.17G009900_146-194     .FCRV......CSREL.....WLNRG............TCP...LCNRSILEI.......
lcl|Glyma.09G008200_144-192     .FCRV......CSREL.....WLNRG............SCP...LCNRSILEI.......
lcl|Glyma.15G112600_157-205     .FCRV......CSREL.....WLNRG............SCP...LCNRSILEI.......
lcl|Glyma.04G189900_380-423     .TCCD......CGQDL.....EL................CP...ICRSTIDTR.......
lcl|Glyma.06G175700_381-424     .TCCE......CGQDL.....EL................CP...ICRSTIDTR.......
lcl|Glyma.05G173500_413-456     .TCCD......CGENL.....EC................CP...ICRSTITTR.......
lcl|Glyma.08G130800_413-456     .TCCD......CGENL.....EC................CP...ICRSTITTR.......
lcl|Glyma.11G244000_416-459     .TCCE......CGEDL.....QF................CP...ICRSTIHTR.......
lcl|Glyma.18G013100_412-455     .TCCE......CGEDL.....QF................CP...ICRSTIHTR.......
lcl|Glyma.05G115700_444-487     .TCCE......CGQDL.....QS................CP...ICRSPINTR.......
lcl|Glyma.19G051500_449-492     .TCCE......CGQDL.....QS................CP...ICRSPINTR.......
lcl|Glyma.19G078600_381-424     .TCCE......CGQDL.....QS................CP...MCRSPINTR.......
lcl|Glyma.19G124300_227-270     .TCCE......CGQDL.....QS................CP...MYRSPINTI.......
lcl|Glyma.16G068400_384-427     .TCCE......CGQDL.....QS................CP...MCRSPINAR.......
lcl|Glyma.04G211000_388-427     .TCKE......CGSTL.....SS................CP...MCRHQITTR.......
lcl|Glyma.05G193500_382-421     .TCKE......CGVTL.....SS................CP...MCRQEITTR.......
lcl|Glyma.08G234200_376-419     .TCRD......CGSRL.....TN................CP...ICRQRITNR.......
lcl|Glyma.15G219300_368-411     .TCRD......CGSRL.....TD................CP...ICRQRITNR.......
lcl|Glyma.10G238000_315-375     .LCVR......CALYL.....CSTNNVSSEMLGPPGSIPCP...LCRHGVVSF.......
lcl|Glyma.20G156600_315-375     .LCVR......CALYL.....CSTNNVSSEMVGPPGSIPCP...LCRHGVVSF.......
lcl|Glyma.09G143600_314-374     .LCVR......CALYL.....CSTSNVSSETGGPPGSIPCP...LCRHGIVSF.......
lcl|Glyma.01G178600_28-77       .FCWP......CLYRW.....LHHHSHSQE.........CP...VCKALVQE........
lcl|Glyma.11G063500_28-77       .FCWP......CLYRW.....LHHHSHSQE.........CP...VCKALVQE........
lcl|Glyma.04G034500_31-78       .FCWP......CLYKW.....LHFHSQSRE.........CP...VCKALVEE........
lcl|Glyma.06G034500_29-78       .FCWP......CLYKW.....LHFHSQSRE.........CP...VCKALVEE........
lcl|Glyma.03G145700_157-202     .FCWS......CLYRW.....LHLHSDAKE.........CP...VCKGEVT.........
lcl|Glyma.19G148900_156-201     .FCWP......CLYRW.....LHLHSDAKE.........CP...VCKGEVT.........
lcl|Glyma.08G157200_105-149     .FCWP......CFHKL.....SYAYSDVRE.........CP...VCKGDV..........
lcl|Glyma.15G269000_105-151     .FCWP......CFHKL.....SYAYSNVRE.........CP...VCKGDVTE........
lcl|Glyma.09G001100_123-170     .FCWQ......CFYQV.....QIVYSNARE.........CP...VCKGEVTET.......
lcl|Glyma.12G240800_129-176     .FCWP......CFYQV.....QIVYSNARE.........CP...VCKGEVTET.......
lcl|Glyma.06G293400_33-91       .YCWP......CIYKW.....LHFQSTSLDDEEQQR.PQCP...VCKSEVSQS.......
lcl|Glyma.12G112000_36-91       .YCWP......CIYKW.....LHFQSTSLDNEEQQK.PQCP...VCKSEVSQS.......
lcl|Glyma.12G203900_46-99       .YCWP......CIYKW.....LNFLSASCENEEKQ...QCP...VCKSEISQS.......
lcl|Glyma.13G297700_44-94       .YCWP......CIYKW.....LNLQTASSENEEEKQ..QCP...VCK.............
lcl|Glyma.12G151200_37-90       .YCWP......CIYKW.....LDVQSSSVEPYQQQ...TCP...VCKSEISHT.......
lcl|Glyma.07G196000_33-84       .YCWP......CIYKW.....LHVQSASLAPDEHP...QCP...VCKDDI..........
lcl|Glyma.13G179900_30-81       .YCWP......CIYKW.....LHVQSASLAPDEHP...QCP...VCKDDI..........
lcl|Glyma.13G239500_29-80       .YCWP......CIYKW.....LHVQSDSLPPDEHP...QCP...VCKADI..........
lcl|Glyma.15G073700_29-80       .YCWP......CIYKW.....LHVQSDSLAPDEHP...QCP...VCKADI..........
lcl|Glyma.14G099100_339-384     .FCWN......CITEW.....CNEKPE............CP...LCRTPITHS.......
lcl|Glyma.17G225600_339-384     .FCWN......CITEW.....CNEKPE............CP...LCRTPITHS.......
lcl|Glyma.01G074000_824-869     .FCYS......CIQ.............KVAGSRHR...KCP...QCGTS..........
lcl|Glyma.11G175700_818-875     .FCYS......CIQ.............KVAGSRHR...KCP...QCSTSFGANDVK....
lcl|Glyma.02G267300_828-871     .FCNP......CIQ.............RNLELRHR...KCP...ACGTAF.........
lcl|Glyma.14G050200_828-871     .FCNP......CIQ.............RNLELRHR...KCP...ACGTAF.........
lcl|Glyma.10G288200_718-762     .FCYQ......CVS.............EYLTGDDN...MCPS.VNCKELI..........
lcl|Glyma.20G100800_721-765     .FCYQ......CVS.............EYLTGDDN...TCPS.VNCKELI..........
lcl|Glyma.04G125600_69-110      .FCKA......CIS.............RFKD........CP...LCGADIVKI.......
lcl|Glyma.06G313000_69-110      .FCKA......CIL.............RFKD........CP...LCGADIVKI.......
lcl|Glyma.10G155500_28-80       .FCFN......CILRW.....TKVVAGKHRSPPSSV..KCP...LCKTE...........
lcl|Glyma.20G232800_26-78       .FCFN......CILRW.....TKVVASKHRSPPSSV..KCP...LCKTE...........
lcl|Glyma.04G255000_11-57       .FCGN......CIM..........LVWQHGSVGCSC..KCP...LCRRAI..........
lcl|Glyma.11G103800_150-191     .FCKD......CIKA......AISAQG...........KCP...TCRKKV..........
lcl|Glyma.12G028700_150-191     .FCKD......CIKA......AISAQG...........KCP...TCRKKV..........
lcl|Glyma.09G281300_151-194     .FCKN......CIRA......AISAQA...........KCP...TCRKKVTK........
lcl|Glyma.20G004500_80-121      .FCKN......CIRA......ALSAQA...........KCP...TCRKVT..........
lcl|Glyma.03G124900_577-621     .FCRI......CILR......ALQNKNP..........CCP...LCRR............
lcl|Glyma.03G214100_219-270     .FCYI......CACS......AASVSIVNGLKSADPKMKCP...LCRE............
lcl|Glyma.19G210900_219-269     .FCYI......CACS......AASVSIVNGLKSADPKMKCP...LCR.............
lcl|Glyma.10G018800_210-261     .FCYT......CACS......TASVTIVDGLKAANPKEKCP...LCRE............
lcl|Glyma.19G203000_218-270     .FCKS......CACS......AASVMIFQGLKAASPESKCP...ICREV...........
lcl|Glyma.08G153800_209-269     .FCIS......CIRNWRSS.NPTLGMDVN.....STLRACP...ICRK............
lcl|Glyma.15G272400_209-269     .FCIS......CIRNWRSS.NPTLGMDVN.....STLRACP...ICRK............
lcl|Glyma.08G154100_188-251     .FCLS......CIRNWRNSAAPTSGMDIGNAGTANTVRTCP...VC..............
lcl|Glyma.15G273000_188-250     .FCLS......CIRNWRNS.APTSGMDISNAGTANTVRTCP...VC..............
lcl|Glyma.10G151500_34-80       .FHLD......CIG.........SAFNIK......GAMQCP...NCRK............
lcl|Glyma.20G236700_42-88       .FHLD......CIG.........SAFNIK......GAMQCP...NCRK............
lcl|Glyma.19G232800_18-67       .FHLD......CIG.........SAFNIK......GAMQCP...NCRK............
lcl|Glyma.02G219200_1354-1408   .TCCK......CLFAMT..EKRLQNSKLHNW......VMCP...TCRQH...........
lcl|Glyma.14G186500_414-466     .TCCK......CLFAMT..EKRLQNNKLHNW......VMCP...TCRQHT..........
lcl|Glyma.06G290400_82-126      .FCRE......CIDKS....MRLGNNE............CP...ACRTHC..........
lcl|Glyma.12G116500_60-104      .FCRE......CIDKS....MRLGNNE............CP...ACRTHC..........
lcl|Glyma.12G205500_121-165     .FCRE......CIDKS....MRLGNNE............CP...ACRTHC..........
lcl|Glyma.13G295300_124-168     .FCRE......CIDKS....MRLGNNE............CP...ACRTHC..........
lcl|Glyma.08G350700_40-90       .FCRD......CIEKS....FRLGNNE............CP...ACRT............
lcl|Glyma.13G177200_138-181     .FCGK......CIEKS....MRLSNNE............CP...VCRVHC..........
lcl|Glyma.02G267800_40-81       .FCYM......CIITH......LRNKS...........DCP...CC..............
lcl|Glyma.14G049700_46-87       .FCYM......CIITH......LRNKS...........DCP...CC..............
lcl|Glyma.02G141500_20-65       TFCRK......CIYDKI....TDEEL...........ENCP...VCNIDL..........
lcl|Glyma.10G032500_19-64       TFCRK......CIYDKI....ADEEL...........ENCP...VCNIDL..........
lcl|Glyma.03G159500_34-79       TFCRK......CIYDKI....TDEEI...........ECCP...ICNIDL..........
lcl|Glyma.19G161700_18-63       TFCRK......CIYDKI....TDEEI...........ECCP...ICNIDL..........
lcl|Glyma.10G162800_19-64       TFCRK......CIYEKL....SDEET...........DCCP...VCNIDL..........
lcl|Glyma.20G225400_20-65       TFCRK......CIYEKL....SDEEM...........DCCP...VCHIDL..........
lcl|Glyma.10G180300_32-77       SFCRE......CVDKKL....IDEKL...........KHCP...ICNRDL..........
lcl|Glyma.03G165600_25-74       LFCNS......CLADCI....TAG.............SECA...VCKTTYAQTDVR....
lcl|Glyma.19G166900_24-66       LFCNS......CLADCI....TAG.............PGCA...VCKA............
lcl|Glyma.13G220400_468-515     LFCNS......CIFKSM....KSA.............SACP...VCKIPFTRR.......
lcl|Glyma.15G092000_450-498     LFCNS......CVFKSM....KSA.............SACP...VCKIPFTRR.......
lcl|Glyma.09G099300_2-43        LLCTD......DANKIL......SND...........GACP...ICDQ............
```

```
lcl|Glyma.15G209600_2-43        LLCTD......DANKIL......SND...........GACP...ICDQ............
lcl|Glyma.10G149400_424-470     FFCFA......CIMEWA......KVE...........SRCP...LCKQRF..........
lcl|Glyma.20G238800_406-452     FFCFA......CIMEWA......KVE...........SRCP...LCKQRF..........
lcl|Glyma.17G082300_27-72       HFCFV......CIMEWA......KHE...........SRCP...ICRQRF..........
lcl|Glyma.19G042100_335-385     RFCFP......CIQNWADHTTSMRKT...........STCP...LCKAS...........
lcl|Glyma.09G206500_285-320     FFCLK......CLQTFA......QIH..................................
lcl|Glyma.02G103300_678-724     .FHSG......CIKQWLM.................QKNLCP...ICKT............
lcl|Glyma.07G214600_664-710     .FHSG......CIKQWLM.................QKNLCP...ICKT............
lcl|Glyma.04G039700_603-649     .FHSS......CIKQWLM.................QKNLCP...ICKT............
lcl|Glyma.04G039800_683-729     .FHSS......CIKQWLM.................HKNLCP...ICKT............
lcl|Glyma.06G041000_657-703     .FHSS......CIKQWLM.................HKNLCP...ICKT............
lcl|Glyma.05G241900_382-427     .YHFQ......CIKQWVA.................QKNFCP...VCKQQV..........
lcl|Glyma.08G049400_328-373     .YHFQ......CIKQWAA.................QKNFCP...VCKQQV..........
lcl|Glyma.07G097700_305-350     .YHFQ......CIKQWLV.................HKNFCP...VCKQEV..........
lcl|Glyma.09G180300_320-365     .YHFQ......CIKHWLE.................HKNFCP...VCKQEV..........
lcl|Glyma.14G134600_501-543     .FHVV......CIQEWLR.................HKNWCP...ICK.............
lcl|Glyma.17G198300_478-520     .FHVV......CIQEWMR.................LKNWCP...VCK.............
lcl|Glyma.06G076600_488-533     .YHVA......CIQQWLQ.................LKNWCP...ICKASV..........
lcl|Glyma.03G179300_494-542     .YHVS......CIKKWLS.................LRKLCP...ICKVS...........
lcl|Glyma.19G180000_492-540     .YHVS......CIKKWLS.................MKKLCP...ICKVS...........
lcl|Glyma.10G051200_482-530     .YHVG......CIRKWLS.................MKKVCP...ICKAS...........
lcl|Glyma.13G138700_493-541     .YHVG......CIRKWLS.................MKKVCP...ICKVS...........
lcl|Glyma.06G229700_316-362     .YHAD......CLRRWLL.................EKNVCP...LCKS............
lcl|Glyma.06G234500_258-302     .YHAD......CLRRWLL.................EKNVCP...MCKS............
lcl|Glyma.11G156300_288-334     .YHTD......CLKKWLL.................EKNVCP...MCKS............
lcl|Glyma.06G234300_402-449     .YHAD......CLKKWLL.................VKNVCP...VCKSE...........
lcl|Glyma.12G158500_402-449     .YHAD......CLKKWLL.................VKNVCP...ICKSE...........
lcl|Glyma.13G272700_474-517     .YHAD......CLRTWLV.................VKNVCP...ICKSE...........
lcl|Glyma.04G249100_149-193     .YHSD......CIRKWLQ.................IKKVCP...ICGNEV..........
lcl|Glyma.06G113900_130-174     .YHSD......CISKWLQ.................IKKVCP...ICSNEV..........
lcl|Glyma.13G031100_152-196     .YHTD......CISKWLQ.................IKKVCP...ICNTEI..........
lcl|Glyma.14G152800_154-198     .YHTD......CISKWLQ.................IKKVCP...ICNIEI..........
lcl|Glyma.10G284900_291-328     .YHPE......CINNWLK.................INK......................
lcl|Glyma.20G104300_280-331     .YHPE......CINNWLK.................INKVCP...VCSTEVSAS.......
lcl|Glyma.11G132700_192-240     .YHAS......CGNKWLS.................INKACP...ICYTEV..........
lcl|Glyma.12G057100_192-240     .YHAS......CGNKWLS.................INKACP...ICYTEV..........
lcl|Glyma.12G237700_196-240     .YHGE......CITKWLS.................INKKCP...VCNTEV..........
lcl|Glyma.13G203300_196-240     .YHGE......CITKWLS.................INKKCP...VCNTEV..........
lcl|Glyma.03G184600_122-174     .YGMS......CIKKWLQH..............RRNSNKCP...QCKVK...........
lcl|Glyma.14G147100_225-268     .YCSE......CILRWLD.................NNKTCP...VCR.............
lcl|Glyma.01G219800_243-291     .WHTY......CLSPPLEH....IP..PG.......NWYCFN...CLNS...........
lcl|Glyma.11G023700_243-291     .WHTY......CLSPPLEK....IP..PG.......NWYCFN...CLNS...........
lcl|Glyma.13G170000_1273-1321   .YHTY......CLNPPLAR....IP..EG.......NWYCPS...CVVG...........
lcl|Glyma.19G010200_1251-1297   .YHTY......CLNPPLAR....IP..EG.......NWYCPS...CV.............
lcl|Glyma.02G013500_1244-1297   .THID......CCDPPLTA....VP..EE.......DWFCPK...CSS............
lcl|Glyma.10G014100_1243-1296   .THID......CCDPPLTH....VP..EE.......DWFCPK...CSS............
lcl|Glyma.02G295400_391-439     .FHLK......CLQPSVLRG...IHN.RV.......DWHCMR...C..............
lcl|Glyma.14G018000_392-439     .FHLK......CLQPSVLRG...IHN.RV.......DWHCMR...C..............
lcl|Glyma.09G059700_234-289     .FHQR......CLNPPLDTEN..IPPGDQ.......GWFCKF...CECK...........
lcl|Glyma.15G166200_251-303     .FHQR......CLNPPLDTEN..IPPGDQ.......GWFCKF...C..............
lcl|Glyma.17G059400_224-279     .FHQK......CLDPPLDTEN..IPPGEQ.......GWFCKF...CECK...........
lcl|Glyma.10G260400_343-398     .FHQL......CLDPPMLTED..IPPGDE.......GWLCPG...CDCK...........
lcl|Glyma.20G130800_344-399     .FHQL......CLDPPLLTED..IPPGDE.......GWLCPG...CDCK...........
lcl|Glyma.01G234300_253-300     AEHTY......CMREMLEK....VPEGD.........WLCEE...CK.............
lcl|Glyma.11G008600_251-297     AEHTY......CMREMLEK....VPEGD.........WLCEE...C..............
lcl|Glyma.12G014900_273-322     AEHTY......CMREMLEK....VPEGD.........WLCEE...CKCA...........
lcl|Glyma.11G109800_273-322     AEHTY......CMREMLEK....VPEGD.........WLCEE...CKCA...........
lcl|Glyma.17G155700_20-68       AEHIY......CNDEKLEK....VPDGDW........WTCED...CR.............
lcl|Glyma.04G163100_13-61       CEHSY......CMRFN.......TLIVP.......IDWICEP...CKSK...........
lcl|Glyma.11G043500_25-80       AEHTY......CMDPDEQS....KLEAKPEL...RKDWTCQE...CMPR...........
lcl|Glyma.06G230000_305-354     ARHRY......CLDGP.......VIFTD......DVTWFCED...CEAK...........
lcl|Glyma.09G125400_373-422     ALHRY......CLDGP.......VIFTD......DVTWFCED...CEAK...........
lcl|Glyma.10G227100_4-50        ALHRY......CLKGP.......VVFTD......AVTWFCED...C..............
lcl|Glyma.20G165200_4-53        ALHRY......CLKGP.......VIFTD......AVTWFCED...CATK...........
lcl|Glyma.04G214800_25-74       AEHSY......CLDKF.......HTEDDG.....TLIWRCED...CAP............
lcl|Glyma.04G062400_53-97       .YHPR......CLLPPLK.....GPLPD........NWRCPE...CVS............
lcl|Glyma.06G063400_53-97       .YHPK......CLLPPLK.....GPLPD........NWRCPE...CVS............
lcl|Glyma.12G170900_115-166     .QHIN......CVIIPEKPMDGIPPVPD........KFYCEI...CR.............
lcl|Glyma.13G328100_115-166     .QHIS......CVIIPEKPMDGIPPIPD........KFYCEI...CR.............
lcl|Glyma.12G071300_116-167     .QHIS......CVIIPEKPTEGIPLVPD........KFYCEL...CR.............
lcl|Glyma.04G253800_296-345     .VHRK......CYG.VHDD....IDE..........AWLCSW...CKQK...........
lcl|Glyma.06G108500_295-345     .VHRK......CYG.VYDDD...VDG..........TWMCSW...CKQK...........
lcl|Glyma.15G219200_660-708     .VHST......CLMPPIGDI...VPE..........EWSCHL...CKEK...........
lcl|Glyma.01G238300_516-558     .FHQS......CLD....I..QMLP.........PGEWHCPN...CTCK...........
lcl|Glyma.11G005500_722-764     .FHQS......CLD....I..QMLP.........PGEWRCMN...CTCK...........
lcl|Glyma.01G238200_728-770     .FHQS......CLD....I..QMLP.........LGEWHCPN...CTCK...........
lcl|Glyma.11G005300_720-760     .FHQS......CLD....I..QMLP.........PGEWHCPN...C..............
lcl|Glyma.02G146500_568-610     .FHQG......CLD....I..KKFP.........SGDWHCIY...CCCK...........
lcl|Glyma.03G155500_313-355     .FHQS......CLD....I..QTLP.........SGDWNCIY...CCCK...........
lcl|Glyma.10G044200_547-587     .FHAA......CLG....L..QCVP.........DSGWQCLN...CR.............
lcl|Glyma.13G131800_545-585     .FHAA......CLG....L..QCVP.........DSGWQCLN...CI.............
lcl|Glyma.06G046500_300-343     .FHLS......CLG....L..EHVP.........DGDWFCPA...CCCK...........
lcl|Glyma.11G170900_521-564     .FHKT......CLG....L..EDIP.........NGDWFCPS...CCCG...........
lcl|Glyma.03G181800_43-91       .YHPS......CVDP..PL..NYIP.........LGFWHCIW...CTKK...........
lcl|Glyma.11G074400_81-126      .YHPS......CIKR..DE..AFFRS........KAKWNCGW...H..............
lcl|Glyma.13G320100_386-430     .YHSR......CIGV..MK..MHIP.........EGAWYCPE...CKID...........
lcl|Glyma.02G296200_232-286     .RENS......CVSL..GFTQKEVDE........IQSFYCKN...C..............
lcl|Glyma.18G086000_229-283     .EEST......CASL..GFSRKEVDE........IQNFYCKN...C..............
lcl|Glyma.13G197800_36-86       .VHAT......CLAT..GP...KFDG........SGNFCCPY...C..............
lcl|Glyma.15G236400_75-123      .VHAT......CLGF..EP...EFDD........SGNFCCPY...C..............
lcl|Glyma.13G057300_662-708     .FHAD......CLGI..KEH..EVSS.........RNWSCQT...CICH...........
lcl|Glyma.19G029100_661-707     .FHAD......CLGI..KEH..EVSS.........RKWSCQT...CICH...........
lcl|Glyma.02G227900_820-872     .YHVG......CLKD.REKYESRIY.........MKNWLCGKE..C..............
lcl|Glyma.14G194800_734-787     .YHVE......CLKD.REKSESRRY.........MKNWLCGKE..C..............
lcl|Glyma.11G171200_781-832     .YHVR......CLEN.GAADISTRY.........LGNWFCGK..................
lcl|Glyma.10G062700_503-554     .YHVG......CLRDHKMAYLKELP.........EGDWFC....................
lcl|Glyma.13G147100_501-557     .YHVG......CLRDHKKAYLKELP.........EGDWFCCND..CT.............
lcl|Glyma.02G058300_166-214     .YHGK......CIMMTPTK....GETLKH........YKCAS...CS.............
lcl|Glyma.06G319800_166-214     .YHGK......CIMMTPTK....AETLKH........YKCAS...CS.............
lcl|Glyma.07G271400_198-247     .FHGK......CVKITPAK....AEHIKQ........YKCPS...CSNK...........
lcl|Glyma.17G002600_199-248     .FHGK......CVKITPAK....AEHIKQ........YKCPS...CSNK...........
lcl|Glyma.11G190000_1281-1329   .YHAE......AVELEESK....ISSVLG........FKCSK...CR.............
lcl|Glyma.12G084300_1287-1335   .YHAE......AVELEESK....ISSVLG........FKCCK...CR.............
```

```
lcl|Glyma.12G180700_1293-1341  .YHAE......AVELEESK.....LFDVLG........FKCCK...CR.............
lcl|Glyma.07G056000_373-427    .VHAE......CDKISSKV.....FKDLENT......DYYCPD...CKGK...........
lcl|Glyma.16G024900_371-425    .VHAE......CDKISSKL.....FKDLENT......DYYCPD...CKGK...........
lcl|Glyma.03G215600_371-425    .VHAE......CDKISSKH.....FKDLENT......DYYCPD...CKGK...........
lcl|Glyma.19G212100_374-428    .VHAE......CDKISSKL.....FKDLENA......DYYCPD...CKGK...........
lcl|Glyma.04G236500_434-488    .VHAE......CDKICSNL.....FKNLEGT......DYYCPT...CKAK...........
lcl|Glyma.06G127800_441-495    .VHAE......CDKISSNL.....FKNLEGT......DYYCPT...CKAK...........
lcl|Glyma.19G066800_376-430    .VHAE......CDKISSNL.....FKNLGGS......DYFCPT...CKIK...........
lcl|Glyma.01G179800_176-228    VWHPE......CFCCHACH...........LPITDYEFSMSSNRPYHKAC...........
lcl|Glyma.11G062400_192-244    VWHPE......CFCCHACH...........LPITDYEFSMSSNRPYHKSC...........
lcl|Glyma.02G059900_158-214    YWHPE......CFCCHACK...........LPITDYEFSMSGNRRYHKSCYKELH......
lcl|Glyma.16G142700_157-213    YWHPE......CFCCHACK...........LPITDYEFSMSGNRRYHKSCYKELH......
lcl|Glyma.12G160000_164-201    YWHPE......CFCCHACK...........LPITD..........................
lcl|Glyma.14G077800_125-177    FWHPE......CFRCRACN...........LPISDYEFSTSGNYPYHKSC...........
lcl|Glyma.17G247700_120-172    FWHPE......CFRCRACN...........LPISDYEFSTSGNYPYHKSC...........
lcl|Glyma.02G251800_176-228    YFHPN......CFRCHSCG...........YPITEREFSLSGKHPYHKSC...........
lcl|Glyma.14G065000_141-193    YFHPN......CFRCHSCG...........YPITEREFSLSGKHPYHKSC...........
lcl|Glyma.02G253700_47-99      AYHKA......CFKCSHCE...........GTLKLSNYSSMESVLYCKPHNEQLF......
lcl|Glyma.18G030900_10-66      AYHKA......CFRCSHCK...........GTLKLSNYSSMEGVLYCKPHYEQLF......
lcl|Glyma.05G205800_10-66      AYHKN......CFRCSHCN...........GLLAISNYSSTEGVLYCKVHFEQLF......
lcl|Glyma.08G012700_10-66      AYHKN......CFRCSHCN...........GLLAISNYSSTEGVLYCKVHFEQLF......
lcl|Glyma.03G013900_10-66      TYHKN......CFKCSHCK...........GCLTMSTYSSMDGILYCKTHFEQLF......
lcl|Glyma.07G073600_10-66      PYHKN......CFKCSHCK...........GCLTMCTYSSMDGILYCKTHFEQLF......
lcl|Glyma.09G261800_10-66      PYHKN......CFRCSHCK...........GCLTMSTYSSMDGVLYCKPHFEQLF......
lcl|Glyma.18G230400_10-66      PYHKN......CFRCSHCK...........GYLTMNTYSSMDGVLYCKPHFEQLF......
lcl|Glyma.04G179800_24-80      IYHKS......CFRCYHCK...........GTLKLSNYCSFEGVLYCKPHFDQLF......
lcl|Glyma.05G034700_11-67      VYHKS......CFRCHHCK...........GTLKLSNYCSFEGVLYCKPHFHQLF......
lcl|Glyma.10G114300_10-49      IYHKS......CFRCYHCK...........GTLKNT............P............
lcl|Glyma.20G085200_10-49      IYHKS......CFRCYHCK...........GTLKNN............P............
lcl|Glyma.04G046200_109-164    PYHKS......CFKCTHGG...........CVISPSNYIAHEGKLYCKHHHIQL.......
lcl|Glyma.06G047000_109-164    PYHKS......CFKCTHGG...........CVISPSNYIAHEGKLYCKHHHVQL.......
lcl|Glyma.17G234700_109-164    PYHKS......CFKCCHGG...........CVISPSNYIAHEGKLYCKHHHVQL.......
lcl|Glyma.10G256600_111-167    PYHKG......CFKCTYGG...........CTVSSSNFITHEGKLYCKHHHIQLF......
lcl|Glyma.06G185000_110-162    SYHKA......CFRCTHGG...........CVISPSNYVAHEHRLYCRHH...........
lcl|Glyma.17G092600_110-166    SYHKS......CFRCTHGG...........CVISPSNYVAHEHRLYCRHHHTQLF......
lcl|Glyma.11G226400_108-164    AYHKS......CFKCSHGG...........CPITPSNYAALEGILYCKHHFSQLF......
lcl|Glyma.14G062700_108-164    AYHKS......CFKCSHGS...........CPITPSNYAALEGVLYCKHHFSQLF......
lcl|Glyma.14G112700_1508-1555  LIRVH....QACYGVSSLPK...............KSSWCCR...PCR.............
lcl|Glyma.13G253900_326-371    .LCKQ......SIMKLSKNS...............TRTFKCP...YC..............
lcl|Glyma.13G273500_171-204    .YEKK......VIMQYLKS.................KQHQCP...I...............
```

Human_c-Cbl

```
Human_c-Cbl                       .........
consensus                         .........
lcl|Glyma.09G103000_21-70         .........
lcl|Glyma.15G275000_20-69         .........
lcl|Glyma.04G067900_40-89         .........
lcl|Glyma.06G069600_30-98         .........
lcl|Glyma.13G202500_20-69         .........
lcl|Glyma.02G080900_38-87         .........
lcl|Glyma.16G165900_30-96         .........
lcl|Glyma.05G187300_39-88         .........
lcl|Glyma.08G145600_39-88         .........
lcl|Glyma.10G223500_41-90         .........
lcl|Glyma.02G205800_37-86         .........
lcl|Glyma.17G072200_27-95         .........
lcl|Glyma.04G153700_37-86         .........
lcl|Glyma.06G225500_37-86         .........
lcl|Glyma.04G142700_37-86         .........
lcl|Glyma.06G225400_46-117        G........
lcl|Glyma.06G316800_40-89         .........
lcl|Glyma.09G051100_38-87         .........
lcl|Glyma.15G157100_38-87         .........
lcl|Glyma.08G088400_32-81         .........
lcl|Glyma.08G302500_39-88         .........
lcl|Glyma.18G117100_39-88         .........
lcl|Glyma.04G063800_9-58          .........
lcl|Glyma.06G065000_6-58          .........
lcl|Glyma.05G160000_385-436       .........
lcl|Glyma.08G117500_6-58          .........
lcl|Glyma.12G017600_131-178       .........
lcl|Glyma.02G090900_13-60         .........
lcl|Glyma.18G292200_14-61         .........
lcl|Glyma.18G242400_64-112        .........
lcl|Glyma.02G103800_77-125        .........
lcl|Glyma.07G215200_80-128        .........
lcl|Glyma.13G372600_71-120        .........
lcl|Glyma.15G001100_69-118        .........
lcl|Glyma.11G236400_33-82         .........
lcl|Glyma.18G020800_33-82         .........
lcl|Glyma.05G182200_29-78         .........
lcl|Glyma.05G010700_54-89         .........
lcl|Glyma.17G118400_55-107        .........
lcl|Glyma.02G310000_267-316       .........
lcl|Glyma.14G002700_268-317       .........
lcl|Glyma.03G180200_231-280       .........
lcl|Glyma.19G180900_243-292       .........
lcl|Glyma.13G005300_253-302       .........
lcl|Glyma.20G064600_253-302       .........
lcl|Glyma.11G147100_266-315       .........
lcl|Glyma.12G068000_266-315       .........
lcl|Glyma.12G173300_234-283       .........
lcl|Glyma.13G326500_259-308       .........
lcl|Glyma.10G051800_240-289       .........
lcl|Glyma.13G139200_260-309       .........
lcl|Glyma.13G213600_217-265       .........
lcl|Glyma.15G099100_214-262       .........
lcl|Glyma.01G107700_99-151        .........
lcl|Glyma.U027600_104-157         .........
lcl|Glyma.07G142300_108-159       .........
lcl|Glyma.02G253000_84-135        .........
lcl|Glyma.14G063500_84-135        .........
lcl|Glyma.13G227900_172-233       .........
lcl|Glyma.15G084600_183-244       .........
lcl|Glyma.04G063700_161-222       .........
lcl|Glyma.06G064900_158-219       .........
lcl|Glyma.05G159600_181-243       .........
lcl|Glyma.04G084500_138-184       .........
lcl|Glyma.06G086100_152-198       .........
lcl|Glyma.02G232900_1829-1880     .........
lcl|Glyma.01G095300_99-145        .........
lcl|Glyma.08G270800_99-145        .........
lcl|Glyma.01G017200_110-156       .........
lcl|Glyma.02G210700_138-184       .........
lcl|Glyma.14G178400_137-183       .........
lcl|Glyma.04G098100_128-174       .........
lcl|Glyma.06G099800_131-177       .........
lcl|Glyma.10G156500_116-162       .........
lcl|Glyma.20G231900_117-163       .........
lcl|Glyma.19G236800_118-164       .........
lcl|Glyma.03G239900_110-156       .........
lcl|Glyma.09G140700_126-172       .........
lcl|Glyma.16G194600_116-162       .........
lcl|Glyma.09G140800_108-154       .........
lcl|Glyma.11G242800_110-156       .........
lcl|Glyma.14G037000_80-126        .........
lcl|Glyma.06G118400_62-106        .........
lcl|Glyma.01G017300_141-187       .........
lcl|Glyma.09G205600_131-177       .........
lcl|Glyma.01G095600_140-186       .........
lcl|Glyma.08G271100_141-187       .........
lcl|Glyma.04G128100_143-189       .........
lcl|Glyma.06G310600_135-181       .........
lcl|Glyma.02G210400_152-198       .........
lcl|Glyma.14G177900_153-199       .........
lcl|Glyma.08G070100_155-204       .........
lcl|Glyma.13G042300_156-201       .........
lcl|Glyma.08G177000_180-226       .........
lcl|Glyma.15G056000_166-212       .........
lcl|Glyma.05G175400_149-200       .........
lcl|Glyma.18G014500_194-240       .........
lcl|Glyma.18G014600_153-202       .........
lcl|Glyma.11G242500_156-205       .........
lcl|Glyma.11G122400_135-179       .........
lcl|Glyma.12G047300_133-176       .........
```

```
lcl|Glyma.01G032200_147-191    .........
lcl|Glyma.02G033200_149-193    .........
lcl|Glyma.13G071600_172-216    .........
lcl|Glyma.19G011200_168-212    .........
lcl|Glyma.08G289200_148-194    .........
lcl|Glyma.18G135300_149-195    .........
lcl|Glyma.05G035800_139-185    .........
lcl|Glyma.17G091600_134-180    .........
lcl|Glyma.13G092000_129-175    .........
lcl|Glyma.17G068300_125-171    .........
lcl|Glyma.03G103800_137-183    .........
lcl|Glyma.07G120300_138-184    .........
lcl|Glyma.15G183300_121-167    .........
lcl|Glyma.01G146500_110-156    .........
lcl|Glyma.09G194100_115-161    .........
lcl|Glyma.04G083400_113-140    .........
lcl|Glyma.06G084800_126-176    .........
lcl|Glyma.14G146700_132-182    .........
lcl|Glyma.02G106400_97-139     .........
lcl|Glyma.04G091400_117-163    .........
lcl|Glyma.03G215500_93-139     .........
lcl|Glyma.19G212000_90-136     .........
lcl|Glyma.02G016800_86-132     .........
lcl|Glyma.07G046400_108-154    .........
lcl|Glyma.16G014600_107-153    .........
lcl|Glyma.03G263000_99-148     .........
lcl|Glyma.07G245400_93-139     .........
lcl|Glyma.17G028400_95-141     .........
lcl|Glyma.09G042400_118-164    .........
lcl|Glyma.13G332800_88-134     .........
lcl|Glyma.06G284600_119-165    .........
lcl|Glyma.12G121400_124-171    .........
lcl|Glyma.12G208600_102-147    .........
lcl|Glyma.13G292600_94-142     .........
lcl|Glyma.09G214600_99-144     .........
lcl|Glyma.16G075300_86-130     .........
lcl|Glyma.13G108500_112-158    .........
lcl|Glyma.17G050900_108-154    .........
lcl|Glyma.18G176200_157-203    .........
lcl|Glyma.02G228800_114-163    .........
lcl|Glyma.01G157400_104-150    .........
lcl|Glyma.11G087600_104-150    .........
lcl|Glyma.04G015200_96-142     .........
lcl|Glyma.06G015200_96-142     .........
lcl|Glyma.09G196700_100-146    .........
lcl|Glyma.16G112200_99-145     .........
lcl|Glyma.04G215600_139-185    .........
lcl|Glyma.06G150400_141-187    .........
lcl|Glyma.05G188900_144-190    .........
lcl|Glyma.08G146600_143-189    .........
lcl|Glyma.09G275300_113-159    .........
lcl|Glyma.18G214200_108-154    .........
lcl|Glyma.07G173600_79-113     .........
lcl|Glyma.06G143100_111-157    .........
lcl|Glyma.08G088100_109-152    .........
lcl|Glyma.15G157400_108-152    .........
lcl|Glyma.04G182100_194-239    .........
lcl|Glyma.06G183300_233-278    .........
lcl|Glyma.05G037000_234-279    .........
lcl|Glyma.17G090200_234-279    .........
lcl|Glyma.11G191800_129-174    .........
lcl|Glyma.01G151700_511-557    .........
lcl|Glyma.09G216900_511-557    .........
lcl|Glyma.16G076000_519-565    .........
lcl|Glyma.16G107700_522-568    .........
lcl|Glyma.13G165500_478-524    .........
lcl|Glyma.17G105500_479-525    .........
lcl|Glyma.01G217300_329-374    .........
lcl|Glyma.11G026000_332-377    .........
lcl|Glyma.05G048800_325-370    .........
lcl|Glyma.17G130700_323-368    .........
lcl|Glyma.09G265600_314-359    .........
lcl|Glyma.18G225500_320-365    .........
lcl|Glyma.02G085200_305-350    .........
lcl|Glyma.07G170200_304-349    .........
lcl|Glyma.05G238900_292-337    .........
lcl|Glyma.08G046000_293-338    .........
lcl|Glyma.03G120200_273-318    .........
lcl|Glyma.19G124800_275-320    .........
lcl|Glyma.02G302400_289-335    .........
lcl|Glyma.14G011800_289-335    .........
lcl|Glyma.08G332300_279-325    .........
lcl|Glyma.18G074600_278-324    .........
lcl|Glyma.13G361000_365-409    .........
lcl|Glyma.15G012900_392-436    .........
lcl|Glyma.14G129100_343-387    .........
lcl|Glyma.17G203800_375-419    .........
lcl|Glyma.01G162900_187-232    .........
lcl|Glyma.11G080500_187-232    .........
lcl|Glyma.02G044300_186-231    .........
lcl|Glyma.04G143800_84-127     .........
lcl|Glyma.08G316400_182-225    .........
lcl|Glyma.18G096600_201-244    .........
lcl|Glyma.02G291200_187-233    .........
lcl|Glyma.05G025200_170-216    .........
lcl|Glyma.17G101800_167-213    .........
lcl|Glyma.10G124600_688-735    .........
lcl|Glyma.20G076700_718-765    .........
lcl|Glyma.10G217500_232-277    .........
lcl|Glyma.20G174500_247-292    .........
lcl|Glyma.11G137400_208-259    .........
lcl|Glyma.12G060800_236-287    .........
lcl|Glyma.11G220200_212-254    .........
```

```
lcl|Glyma.18G037200_212-254     .........
lcl|Glyma.02G194400_103-147     .........
lcl|Glyma.10G083100_103-147     .........
lcl|Glyma.03G203400_104-149     .........
lcl|Glyma.10G121100_107-150     .........
lcl|Glyma.10G121500_113-160     .........
lcl|Glyma.03G017500_84-127      .........
lcl|Glyma.07G078800_84-127      .........
lcl|Glyma.09G264300_89-135      .........
lcl|Glyma.18G227700_89-135      .........
lcl|Glyma.10G202500_86-132      .........
lcl|Glyma.20G187900_86-132      .........
lcl|Glyma.09G001600_110-156     .........
lcl|Glyma.05G217000_334-378     .........
lcl|Glyma.08G022900_332-375     .........
lcl|Glyma.07G056200_314-357     .........
lcl|Glyma.16G025100_320-363     .........
lcl|Glyma.19G255700_319-362     .........
lcl|Glyma.06G127300_326-371     .........
lcl|Glyma.07G062800_119-162     .........
lcl|Glyma.16G030900_115-158     .........
lcl|Glyma.09G253500_112-155     .........
lcl|Glyma.18G239400_89-132      .........
lcl|Glyma.09G253400_107-153     .........
lcl|Glyma.02G264800_105-151     .........
lcl|Glyma.14G058000_100-146     .........
lcl|Glyma.11G231400_107-153     .........
lcl|Glyma.18G025700_107-153     .........
lcl|Glyma.11G169500_92-138      .........
lcl|Glyma.18G060400_108-154     .........
lcl|Glyma.01G093800_57-96       .........
lcl|Glyma.04G153400_57-104      .........
lcl|Glyma.04G153500_74-120      .........
lcl|Glyma.13G115900_83-134      .........
lcl|Glyma.17G043900_85-135      .........
lcl|Glyma.01G221300_81-127      .........
lcl|Glyma.11G022400_87-133      .........
lcl|Glyma.05G210000_86-133      .........
lcl|Glyma.08G016700_85-132      .........
lcl|Glyma.17G159400_72-117      .........
lcl|Glyma.02G223600_99-145      .........
lcl|Glyma.14G190300_98-144      .........
lcl|Glyma.13G009100_96-142      .........
lcl|Glyma.05G182000_68-114      .........
lcl|Glyma.08G139700_68-114      .........
lcl|Glyma.11G236600_73-119      .........
lcl|Glyma.18G020400_70-116      .........
lcl|Glyma.05G218700_107-152     .........
lcl|Glyma.08G024800_108-153     .........
lcl|Glyma.13G002800_104-153     .........
lcl|Glyma.20G066300_104-153     .........
lcl|Glyma.10G007200_117-163     .........
lcl|Glyma.20G088900_139-185     .........
lcl|Glyma.10G187100_136-182     .........
lcl|Glyma.20G203400_135-181     .........
lcl|Glyma.19G163600_128-173     .........
lcl|Glyma.11G243400_93-139      .........
lcl|Glyma.18G013800_88-132      .........
lcl|Glyma.13G232800_104-150     .........
lcl|Glyma.13G232800_105-147     .........
lcl|Glyma.15G079900_101-151     .........
lcl|Glyma.04G021200_74-120      .........
lcl|Glyma.06G021400_73-119      .........
lcl|Glyma.14G221800_70-116      .........
lcl|Glyma.17G260700_70-116      .........
lcl|Glyma.04G181300_83-127      .........
lcl|Glyma.06G183600_83-127      .........
lcl|Glyma.13G170300_69-114      .........
lcl|Glyma.19G010400_71-116      .........
lcl|Glyma.13G092100_125-170     .........
lcl|Glyma.17G068200_100-145     .........
lcl|Glyma.10G244900_121-165     .........
lcl|Glyma.20G149700_120-164     .........
lcl|Glyma.01G048700_113-166     .........
lcl|Glyma.02G108300_190-234     .........
lcl|Glyma.02G071200_226-269     .........
lcl|Glyma.16G152400_225-267     .........
lcl|Glyma.18G000200_236-278     .........
lcl|Glyma.02G161600_186-230     .........
lcl|Glyma.10G101400_186-230     .........
lcl|Glyma.02G276700_252-296     .........
lcl|Glyma.14G039000_202-246     .........
lcl|Glyma.13G072800_206-250     .........
lcl|Glyma.13G072900_126-169     .........
lcl|Glyma.04G251600_220-265     .........
lcl|Glyma.08G185400_202-246     .........
lcl|Glyma.15G047300_199-243     .........
lcl|Glyma.09G165200_203-248     .........
lcl|Glyma.16G213500_202-247     .........
lcl|Glyma.10G285300_179-224     .........
lcl|Glyma.20G103700_178-223     .........
lcl|Glyma.02G249100_188-232     .........
lcl|Glyma.14G067300_187-231     .........
lcl|Glyma.11G220400_185-229     .........
lcl|Glyma.18G037000_174-209     .........
lcl|Glyma.07G134600_158-202     .........
lcl|Glyma.18G184200_159-203     .........
lcl|Glyma.11G137300_184-228     .........
lcl|Glyma.12G060700_182-226     .........
lcl|Glyma.13G338400_153-197     .........
lcl|Glyma.15G036100_152-197     .........
lcl|Glyma.10G286700_262-306     .........
lcl|Glyma.20G102400_259-303     .........
```

```
lcl|Glyma.05G017500_280-324      .........
lcl|Glyma.09G271200_369-413      .........
lcl|Glyma.18G218200_373-417      .........
lcl|Glyma.08G158300_95-136       .........
lcl|Glyma.15G268100_109-150      .........
lcl|Glyma.08G034000_52-100       .........
lcl|Glyma.06G272400_167-215      .........
lcl|Glyma.12G131800_171-215      .........
lcl|Glyma.06G274300_168-216      .........
lcl|Glyma.17G213300_14-59        .........
lcl|Glyma.18G103300_98-136       .........
lcl|Glyma.12G223700_140-184      .........
lcl|Glyma.13G277600_139-186      .........
lcl|Glyma.12G151700_161-205      .........
lcl|Glyma.13G211100_115-163      .........
lcl|Glyma.17G211800_21-68        .........
lcl|Glyma.10G047200_194-237      .........
lcl|Glyma.13G135100_190-233      .........
lcl|Glyma.08G148600_91-141       .........
lcl|Glyma.12G223800_101-147      .........
lcl|Glyma.09G096300_141-186      .........
lcl|Glyma.15G201800_154-199      .........
lcl|Glyma.10G259300_189-234      .........
lcl|Glyma.20G131600_187-232      .........
lcl|Glyma.17G172100_265-310      .........
lcl|Glyma.17G172200_262-307      .........
lcl|Glyma.03G110700_123-171      .........
lcl|Glyma.10G289100_82-127       .........
lcl|Glyma.20G099800_77-122       .........
lcl|Glyma.16G006200_74-120       .........
lcl|Glyma.10G142800_16-53        .........
lcl|Glyma.20G091400_16-53        .........
lcl|Glyma.13G265400_16-53        .........
lcl|Glyma.06G303600_75-104       .........
lcl|Glyma.01G143600_200-252      .........
lcl|Glyma.17G108900_203-255      .........
lcl|Glyma.20G044300_97-142       .........
lcl|Glyma.20G044600_112-155      .........
lcl|Glyma.09G257100_165-208      .........
lcl|Glyma.18G235300_138-182      .........
lcl|Glyma.08G016300_162-207      .........
lcl|Glyma.08G251300_174-216      .........
lcl|Glyma.18G274000_173-215      .........
lcl|Glyma.13G052000_177-221      .........
lcl|Glyma.19G034700_177-219      .........
lcl|Glyma.13G206800_34-78        .........
lcl|Glyma.15G005300_166-208      .........
lcl|Glyma.01G019300_126-171      .........
lcl|Glyma.09G203400_19-64        .........
lcl|Glyma.01G097900_111-159      .........
lcl|Glyma.08G273400_110-158      .........
lcl|Glyma.02G218200_86-131       .........
lcl|Glyma.14G185600_109-152      .........
lcl|Glyma.09G017200_92-144       .........
lcl|Glyma.15G122900_92-144       .........
lcl|Glyma.07G270000_80-128       .........
lcl|Glyma.01G050400_262-297      .........
lcl|Glyma.02G109200_262-297      .........
lcl|Glyma.12G235700_273-332      .........
lcl|Glyma.13G201200_274-333      .........
lcl|Glyma.07G049700_1718-1767    .........
lcl|Glyma.16G018400_1710-1759    .........
lcl|Glyma.01G228500_290-332      .........
lcl|Glyma.11G011300_290-332      .........
lcl|Glyma.04G130500_337-383      .........
lcl|Glyma.06G316600_337-383      .........
lcl|Glyma.14G105900_232-269      .........
lcl|Glyma.17G220500_232-277      .........
lcl|Glyma.16G032000_12-61        .........
lcl|Glyma.03G104400_860-896      .........
lcl|Glyma.07G119500_861-897      .........
lcl|Glyma.12G116100_524-572      .........
lcl|Glyma.12G205300_519-567      .........
lcl|Glyma.06G290800_524-572      .........
lcl|Glyma.13G295600_507-555      .........
lcl|Glyma.01G195200_69-131       TLYRLIHTW
lcl|Glyma.11G046500_134-196      TLYRLIHMW
lcl|Glyma.03G202600_12-76        TLRRLIQAW
lcl|Glyma.10G262600_10-74        TLRRLIQSW
lcl|Glyma.19G200200_10-74        TLRRLIQAW
lcl|Glyma.04G212300_16-53        .........
lcl|Glyma.06G154100_14-76        TLRHLINQW
lcl|Glyma.05G192100_12-74        TLRHLIDQW
lcl|Glyma.08G000400_12-74        TLRHLIDQW
lcl|Glyma.05G189600_16-52        .........
lcl|Glyma.08G147200_16-52        .........
lcl|Glyma.07G106000_30-93        SLRIMIQDW
lcl|Glyma.15G064800_12-50        .........
lcl|Glyma.16G048700_180-229      .........
lcl|Glyma.19G103000_221-270      .........
lcl|Glyma.05G237500_1120-1165    .........
lcl|Glyma.08G044700_1118-1163    .........
lcl|Glyma.07G093700_1126-1171    .........
lcl|Glyma.09G182600_1122-1167    .........
lcl|Glyma.09G115100_1126-1168    .........
lcl|Glyma.17G096900_1156-1201    .........
lcl|Glyma.14G130700_200-247      .........
lcl|Glyma.17G202700_200-247      .........
lcl|Glyma.06G074300_199-246      .........
lcl|Glyma.06G116800_4-50         .........
lcl|Glyma.10G228500_5-52         .........
lcl|Glyma.08G241700_44-77        .........
lcl|Glyma.04G134700_4-52         .........
```

```
lcl|Glyma.11G157400_5-54        .........
lcl|Glyma.04G224200_401-445     .........
lcl|Glyma.06G140600_410-454     .........
lcl|Glyma.18G217900_377-421     .........
lcl|Glyma.08G354400_21-65       .........
lcl|Glyma.18G171400_21-65       .........
lcl|Glyma.15G237300_55-95       .........
lcl|Glyma.13G198800_100-140     .........
lcl|Glyma.04G235700_61-101      .........
lcl|Glyma.06G129000_62-102      .........
lcl|Glyma.03G257700_48-88       .........
lcl|Glyma.13G035400_631-678     .........
lcl|Glyma.14G156600_631-678     .........
lcl|Glyma.12G192700_673-714     .........
lcl|Glyma.13G309700_670-711     .........
lcl|Glyma.07G148900_159-201     .........
lcl|Glyma.18G200100_157-199     .........
lcl|Glyma.03G060000_142-184     .........
lcl|Glyma.07G195400_147-189     .........
lcl|Glyma.13G180900_147-189     .........
lcl|Glyma.13G240800_150-198     .........
lcl|Glyma.15G072700_147-189     .........
lcl|Glyma.18G063500_143-185     .........
lcl|Glyma.05G144300_156-204     .........
lcl|Glyma.08G100700_156-204     .........
lcl|Glyma.11G246100_158-206     .........
lcl|Glyma.18G011100_158-206     .........
lcl|Glyma.05G044700_154-201     .........
lcl|Glyma.11G246300_78-119      .........
lcl|Glyma.18G010900_145-192     .........
lcl|Glyma.02G309100_117-162     .........
lcl|Glyma.14G003700_117-162     .........
lcl|Glyma.12G001300_129-175     .........
lcl|Glyma.05G198100_138-187     .........
lcl|Glyma.04G165900_213-256     .........
lcl|Glyma.06G196600_193-236     .........
lcl|Glyma.16G063400_170-217     .........
lcl|Glyma.19G082000_171-218     .........
lcl|Glyma.12G180800_764-813     .........
lcl|Glyma.13G320000_790-839     .........
lcl|Glyma.20G087900_859-904     .........
lcl|Glyma.02G149800_290-333     .........
lcl|Glyma.10G024100_292-330     .........
lcl|Glyma.03G080300_332-382     .........
lcl|Glyma.16G008300_327-377     .........
lcl|Glyma.18G149800_331-381     .........
lcl|Glyma.04G043900_344-393     .........
lcl|Glyma.06G044100_357-407     .........
lcl|Glyma.07G150500_385-433     .........
lcl|Glyma.18G201500_386-434     .........
lcl|Glyma.10G181100_405-450     .........
lcl|Glyma.20G209400_406-451     .........
lcl|Glyma.03G180500_289-337     .........
lcl|Glyma.19G181100_312-356     .........
lcl|Glyma.10G052000_291-337     .........
lcl|Glyma.13G139400_291-337     .........
lcl|Glyma.13G326700_239-283     .........
lcl|Glyma.07G257200_279-325     .........
lcl|Glyma.17G016900_219-265     .........
lcl|Glyma.13G208500_314-360     .........
lcl|Glyma.15G104300_316-362     .........
lcl|Glyma.09G048200_866-915     .........
lcl|Glyma.15G155500_861-910     .........
lcl|Glyma.10G198600_793-842     .........
lcl|Glyma.20G191400_792-841     .........
lcl|Glyma.11G191900_808-850     .........
lcl|Glyma.12G082400_798-847     .........
lcl|Glyma.12G179300_740-789     .........
lcl|Glyma.13G321300_793-842     .........
lcl|Glyma.13G212200_825-875     .........
lcl|Glyma.15G100500_827-877     .........
lcl|Glyma.17G020500_819-869     .........
lcl|Glyma.07G253800_821-871     .........
lcl|Glyma.02G150400_776-826     .........
lcl|Glyma.05G112600_659-709     .........
lcl|Glyma.17G154400_621-671     .........
lcl|Glyma.11G042600_620-669     .........
lcl|Glyma.04G009100_1020-1065   .........
lcl|Glyma.06G009000_1020-1065   .........
lcl|Glyma.16G098500_18-63       .........
lcl|Glyma.07G159000_32-77       .........
lcl|Glyma.11G112700_1028-1064   .........
lcl|Glyma.12G038600_1038-1083   .........
lcl|Glyma.05G013200_415-460     .........
lcl|Glyma.17G121200_415-460     .........
lcl|Glyma.10G204900_433-478     .........
lcl|Glyma.20G185600_422-467     .........
lcl|Glyma.01G029500_286-325     .........
lcl|Glyma.02G035800_286-325     .........
lcl|Glyma.14G083900_301-342     .........
lcl|Glyma.17G241100_292-333     .........
lcl|Glyma.08G170400_204-247     .........
lcl|Glyma.15G256500_288-331     .........
lcl|Glyma.09G019900_261-297     .........
lcl|Glyma.15G126400_264-300     .........
lcl|Glyma.01G203300_274-316     .........
lcl|Glyma.11G039700_281-323     .........
lcl|Glyma.05G068400_302-344     .........
lcl|Glyma.17G150900_309-344     .........
lcl|Glyma.09G282200_4713-4753   .........
lcl|Glyma.20G003400_4712-4751   .........
lcl|Glyma.04G220000_1217-1265   .........
lcl|Glyma.06G145900_1217-1265   .........
```

```
lcl|Glyma.08G358300_319-375     .........
lcl|Glyma.18G176600_318-374     .........
lcl|Glyma.12G063600_316-372     .........
lcl|Glyma.11G140000_316-372     .........
lcl|Glyma.13G335300_323-381     .........
lcl|Glyma.15G039100_316-374     .........
lcl|Glyma.08G315500_281-340     .........
lcl|Glyma.03G079200_225-269     .........
lcl|Glyma.16G094800_214-258     .........
lcl|Glyma.08G350900_306-345     .........
lcl|Glyma.18G165000_306-353     .........
lcl|Glyma.07G264100_155-203     .........
lcl|Glyma.17G009900_146-194     .........
lcl|Glyma.09G008200_144-192     .........
lcl|Glyma.15G112600_157-205     .........
lcl|Glyma.04G189900_380-423     .........
lcl|Glyma.06G175700_381-424     .........
lcl|Glyma.05G173500_413-456     .........
lcl|Glyma.08G130800_413-456     .........
lcl|Glyma.11G244000_416-459     .........
lcl|Glyma.18G013100_412-455     .........
lcl|Glyma.05G115700_444-487     .........
lcl|Glyma.19G051500_449-492     .........
lcl|Glyma.19G078600_381-424     .........
lcl|Glyma.19G124300_227-270     .........
lcl|Glyma.16G068400_384-427     .........
lcl|Glyma.04G211000_388-427     .........
lcl|Glyma.05G193500_382-421     .........
lcl|Glyma.08G234200_376-419     .........
lcl|Glyma.15G219300_368-411     .........
lcl|Glyma.10G238000_315-375     .........
lcl|Glyma.20G156600_315-375     .........
lcl|Glyma.09G143600_314-374     .........
lcl|Glyma.01G178600_28-77       .........
lcl|Glyma.11G063500_28-77       .........
lcl|Glyma.04G034500_31-78       .........
lcl|Glyma.06G034500_29-78       .........
lcl|Glyma.03G145700_157-202     .........
lcl|Glyma.19G148900_156-201     .........
lcl|Glyma.08G157200_105-149     .........
lcl|Glyma.15G269000_105-151     .........
lcl|Glyma.09G001100_123-170     .........
lcl|Glyma.12G240800_129-176     .........
lcl|Glyma.06G293400_33-91       .........
lcl|Glyma.12G112000_36-91       .........
lcl|Glyma.12G203900_46-99       .........
lcl|Glyma.13G297700_44-94       .........
lcl|Glyma.12G151200_37-90       .........
lcl|Glyma.07G196000_33-84       .........
lcl|Glyma.13G179900_30-81       .........
lcl|Glyma.13G239500_29-80       .........
lcl|Glyma.15G073700_29-80       .........
lcl|Glyma.14G099100_339-384     .........
lcl|Glyma.17G225600_339-384     .........
lcl|Glyma.01G074000_824-869     .........
lcl|Glyma.11G175700_818-875     .........
lcl|Glyma.02G267300_828-871     .........
lcl|Glyma.14G050200_828-871     .........
lcl|Glyma.10G288200_718-762     .........
lcl|Glyma.20G100800_721-765     .........
lcl|Glyma.04G125600_69-110      .........
lcl|Glyma.06G313000_69-110      .........
lcl|Glyma.10G155500_28-80       .........
lcl|Glyma.20G232800_26-78       .........
lcl|Glyma.04G255000_11-57       .........
lcl|Glyma.11G103800_150-191     .........
lcl|Glyma.12G028700_150-191     .........
lcl|Glyma.09G281300_151-194     .........
lcl|Glyma.20G004500_80-121      .........
lcl|Glyma.03G124900_577-621     .........
lcl|Glyma.03G214100_219-270     .........
lcl|Glyma.19G210900_219-269     .........
lcl|Glyma.10G018800_210-261     .........
lcl|Glyma.19G203000_218-270     .........
lcl|Glyma.08G153800_209-269     .........
lcl|Glyma.15G272400_209-269     .........
lcl|Glyma.08G154100_188-251     .........
lcl|Glyma.15G273000_188-250     .........
lcl|Glyma.10G151500_34-80       .........
lcl|Glyma.20G236700_42-88       .........
lcl|Glyma.19G232800_18-67       .........
lcl|Glyma.02G219200_1354-1408   .........
lcl|Glyma.14G186500_414-466     .........
lcl|Glyma.06G290400_82-126      .........
lcl|Glyma.12G116500_60-104      .........
lcl|Glyma.12G205500_121-165     .........
lcl|Glyma.13G295300_124-168     .........
lcl|Glyma.08G350700_40-90       .........
lcl|Glyma.13G177200_138-181     .........
lcl|Glyma.02G267800_40-81       .........
lcl|Glyma.14G049700_46-87       .........
lcl|Glyma.02G141500_20-65       .........
lcl|Glyma.10G032500_19-64       .........
lcl|Glyma.03G159500_34-79       .........
lcl|Glyma.19G161700_18-63       .........
lcl|Glyma.10G162800_19-64       .........
lcl|Glyma.20G225400_20-65       .........
lcl|Glyma.10G180300_32-77       .........
lcl|Glyma.03G165600_25-74       .........
lcl|Glyma.19G166900_24-66       .........
lcl|Glyma.13G220400_468-515     .........
lcl|Glyma.15G092000_450-498     .........
lcl|Glyma.09G099300_2-43        .........
```

```
lcl|Glyma.15G209600_2-43         .........
lcl|Glyma.10G149400_424-470      .........
lcl|Glyma.20G238800_406-452      .........
lcl|Glyma.17G082300_27-72        .........
lcl|Glyma.19G042100_335-385      .........
lcl|Glyma.09G206500_285-320      .........
lcl|Glyma.02G103300_678-724      .........
lcl|Glyma.07G214600_664-710      .........
lcl|Glyma.04G039700_603-649      .........
lcl|Glyma.04G039800_683-729      .........
lcl|Glyma.06G041000_657-703      .........
lcl|Glyma.05G241900_382-427      .........
lcl|Glyma.08G049400_328-373      .........
lcl|Glyma.07G097700_305-350      .........
lcl|Glyma.09G180300_320-365      .........
lcl|Glyma.14G134600_501-543      .........
lcl|Glyma.17G198300_478-520      .........
lcl|Glyma.06G076600_488-533      .........
lcl|Glyma.03G179300_494-542      .........
lcl|Glyma.19G180000_492-540      .........
lcl|Glyma.10G051200_482-530      .........
lcl|Glyma.13G138700_493-541      .........
lcl|Glyma.06G229700_316-362      .........
lcl|Glyma.06G234500_258-302      .........
lcl|Glyma.11G156300_288-334      .........
lcl|Glyma.06G234300_402-449      .........
lcl|Glyma.12G158500_402-449      .........
lcl|Glyma.13G272700_474-517      .........
lcl|Glyma.04G249100_149-193      .........
lcl|Glyma.06G113900_130-174      .........
lcl|Glyma.13G031100_152-196      .........
lcl|Glyma.14G152800_154-198      .........
lcl|Glyma.10G284900_291-328      .........
lcl|Glyma.20G104300_280-331      .........
lcl|Glyma.11G132700_192-240      .........
lcl|Glyma.12G057100_192-240      .........
lcl|Glyma.12G237700_196-240      .........
lcl|Glyma.13G203300_196-240      .........
lcl|Glyma.03G184600_122-174      .........
lcl|Glyma.14G147100_225-268      .........
lcl|Glyma.01G219800_243-291      .........
lcl|Glyma.11G023700_243-291      .........
lcl|Glyma.13G170000_1273-1321    .........
lcl|Glyma.19G010200_1251-1297    .........
lcl|Glyma.02G013500_1244-1297    .........
lcl|Glyma.10G014100_1243-1296    .........
lcl|Glyma.02G295400_391-439      .........
lcl|Glyma.14G018000_392-439      .........
lcl|Glyma.09G059700_234-289      .........
lcl|Glyma.15G166200_251-303      .........
lcl|Glyma.17G059400_224-279      .........
lcl|Glyma.10G260400_343-398      .........
lcl|Glyma.20G130800_344-399      .........
lcl|Glyma.01G234300_253-300      .........
lcl|Glyma.11G008600_251-297      .........
lcl|Glyma.12G014900_273-322      .........
lcl|Glyma.11G109800_273-322      .........
lcl|Glyma.17G155700_20-68        .........
lcl|Glyma.04G163100_13-61        .........
lcl|Glyma.11G043500_25-80        .........
lcl|Glyma.06G230000_305-354      .........
lcl|Glyma.09G125400_373-422      .........
lcl|Glyma.10G227100_4-50         .........
lcl|Glyma.20G165200_4-53         .........
lcl|Glyma.04G214800_25-74        .........
lcl|Glyma.04G062400_53-97        .........
lcl|Glyma.06G063400_53-97        .........
lcl|Glyma.12G170900_115-166      .........
lcl|Glyma.13G328100_115-166      .........
lcl|Glyma.12G071300_116-167      .........
lcl|Glyma.04G253800_296-345      .........
lcl|Glyma.06G108500_295-345      .........
lcl|Glyma.15G219200_660-708      .........
lcl|Glyma.01G238300_516-558      .........
lcl|Glyma.11G005500_722-764      .........
lcl|Glyma.01G238200_728-770      .........
lcl|Glyma.11G005300_720-760      .........
lcl|Glyma.02G146500_568-610      .........
lcl|Glyma.03G155500_313-355      .........
lcl|Glyma.10G044200_547-587      .........
lcl|Glyma.13G131800_545-585      .........
lcl|Glyma.06G046500_300-343      .........
lcl|Glyma.11G170900_521-564      .........
lcl|Glyma.03G181800_43-91        .........
lcl|Glyma.11G074400_81-126       .........
lcl|Glyma.13G320100_386-430      .........
lcl|Glyma.02G296200_232-286      .........
lcl|Glyma.18G086000_229-283      .........
lcl|Glyma.13G197800_36-86        .........
lcl|Glyma.15G236400_75-123       .........
lcl|Glyma.13G057300_662-708      .........
lcl|Glyma.19G029100_661-707      .........
lcl|Glyma.02G227900_820-872      .........
lcl|Glyma.14G194800_734-787      .........
lcl|Glyma.11G171200_781-832      .........
lcl|Glyma.10G062700_503-554      .........
lcl|Glyma.13G147100_501-557      .........
lcl|Glyma.02G058300_166-214      .........
lcl|Glyma.06G319800_166-214      .........
lcl|Glyma.07G271400_198-247      .........
lcl|Glyma.17G002600_199-248      .........
lcl|Glyma.11G190000_1281-1329    .........
lcl|Glyma.12G084300_1287-1335    .........
```

```
lcl|Glyma.12G180700_1293-1341  .........
lcl|Glyma.07G056000_373-427    .........
lcl|Glyma.16G024900_371-425    .........
lcl|Glyma.03G215600_371-425    .........
lcl|Glyma.19G212100_374-428    .........
lcl|Glyma.04G236500_434-488    .........
lcl|Glyma.06G127800_441-495    .........
lcl|Glyma.19G066800_376-430    .........
lcl|Glyma.01G179800_176-228    .........
lcl|Glyma.11G062400_192-244    .........
lcl|Glyma.02G059900_158-214    .........
lcl|Glyma.16G142700_157-213    .........
lcl|Glyma.12G160000_164-201    .........
lcl|Glyma.14G077800_125-177    .........
lcl|Glyma.17G247700_120-172    .........
lcl|Glyma.02G251800_176-228    .........
lcl|Glyma.14G065000_141-193    .........
lcl|Glyma.02G253700_47-99      .........
lcl|Glyma.18G030900_10-66      .........
lcl|Glyma.05G205800_10-66      .........
lcl|Glyma.08G012700_10-66      .........
lcl|Glyma.03G013900_10-66      .........
lcl|Glyma.07G073600_10-66      .........
lcl|Glyma.09G261800_10-66      .........
lcl|Glyma.18G230400_10-66      .........
lcl|Glyma.04G179800_24-80      .........
lcl|Glyma.05G034700_11-67      .........
lcl|Glyma.10G114300_10-49      .........
lcl|Glyma.20G085200_10-49      .........
lcl|Glyma.04G046200_109-164    .........
lcl|Glyma.06G047000_109-164    .........
lcl|Glyma.17G234700_109-164    .........
lcl|Glyma.10G256600_111-167    .........
lcl|Glyma.06G185000_110-162    .........
lcl|Glyma.17G092600_110-166    .........
lcl|Glyma.11G226400_108-164    .........
lcl|Glyma.14G062700_108-164    .........
lcl|Glyma.14G112700_1508-1555  .........
lcl|Glyma.13G253900_326-371    .........
lcl|Glyma.13G273500_171-204    .........
```
