# Supplementary material for: Genome-wide analysis of genes encoding core components of the ubiquitin system in soybean (Glycine max) reveals a potential role for ubiquitination in host immunity against soybean cyst nematode
Source: BMC Plant Biol. 2018 Jul 18;18:149. doi: 10.1186/s12870-018-1365-7 (PMC6052599; doi:10.1186/s12870-018-1365-7)
Supplement: Supplementary file 8 — Figure S5. Alignment of sequences of the U-box domain from soybean U-box domain-containing proteins. (PDF 27 kb) [file 12870_2018_1365_MOESM8_ESM.pdf]

```
AtPUB14                                          β1      β2     α1
                                      TTT                                       TTT
                              1        10        20        30         40        50
AtPUB14                       IPEYFRCPISLELMKDP.VIVSTGQTYERSSIQKWLD....AGH...KTCPKSQETLL..
consensus                     ...EFLCPISLEVMKDPVILPSG.QTYERSAIEKWLL....SHG....TDPVTGQPLTHE
lcl|Glyma.13G059200_942-1013  .PDEFLDPIQYTLMKDPVILPSSRITVDRPVIQRHLL....SDS....TDPFNRSHLTAD
lcl|Glyma.19G027200_940-1011  .PDEFLDPIQYTLMKDPVILPSSRITVDRPVIQRHLL....SDS....TDPFNRSHLTAD
lcl|Glyma.10G208200_248-320   .PDDFRCPISLELMKDP.VIVSTGQTYERSCIQKWLD....AGH...KTCPKTQQTLV..
lcl|Glyma.20G182600_247-319   .PDDFRCPISLELMKDP.VIVSTGQTYERSCIQKWLD....AGH...KTCPKTQQTLV..
lcl|Glyma.11G140100_256-328   .PDDFRCPISLELMKDP.VIVSTGQTYERTCIEKWLQ....AGH...GTCPKTQQTLT..
lcl|Glyma.12G063700_257-329   .PDDFRCPISLELMKDP.VIVSTGQTYERTCIEKWLQ....AGH...GTCPKTQQTLT..
lcl|Glyma.18G176400_260-330   .PDEFRCPISLELMKDP.VIICTGQTYDRSCIKKWLE....AGH...RTCPMTQQILS..
lcl|Glyma.03G253100_49-114    ..PQFRCPISTQLMSDP.VILSTGQTYDRPFIQRWLN....EGH...RTCPQTQQVLS..
lcl|Glyma.19G250700_62-127    ..PQFRCPISTQLMSDP.VILSTGQTYDRPFIQRWLN....EGH...RTCPQTQQVLS..
lcl|Glyma.20G220600_60-130    .PPHFRCPLSGNLMTDP.VILASGQNFDRAFIQRWLN....EVR...RICPKTQQVLS..
lcl|Glyma.08G345800_78-143    ..DEFKCPLSKELMRDP.VIVASGQTYDRPFIQKWLN....AGN...RTCPRTHQVLS..
lcl|Glyma.18G155200_78-143    ..DEFKCPLSKELMRDP.VILASGQTYDRPFIQKWLN....AGN...RTCPRTHQVLS..
lcl|Glyma.07G188400_8-72      ....FKCPISLEIMSDP.VILSSGHTFDRSSIQRWLD....AGH...RTCPITKLPLPD.
lcl|Glyma.08G061100_8-72      ....FKCPISLEIMSDP.VILSSGHTFDRSSIQRWLD....AGH...RTCPITKLPLPD.
lcl|Glyma.13G249400_8-71      ..EYLKCPISLEIMSDP.VILSSGHTFDRSSIQRWLD....AGH...RTCPITKLPLPE.
lcl|Glyma.15G064800_8-71      ..DHFKCPISLQIMSDP.VILSSGHTFDRSSIQRWLD....AGH...RTCPITKLPLPA.
lcl|Glyma.07G216200_275-347   .PEDFLCPISLELMRDP.VIVATGQTYERSYIQRWID....CGN...TTCPKTQQKLQHL
lcl|Glyma.20G013200_272-344   .PEDFLCPISLELMRDP.VIVATGQTYERSYIQRWID....CGN...TTCPKTQQKLQHL
lcl|Glyma.01G131800_273-345   .PADYRCPISLELMRDP.VVVATGQTYDRASIKLWMD....SGH...NTCPKTGQTLSHT
lcl|Glyma.03G036700_271-343   .PADYRCPISLELMRDP.VVVATGQTYDRVSIKLWMD....SGH...NTCPKTGQTLSHS
lcl|Glyma.05G162100_287-357   .PKDFCCPISLDLMCDP.VIISTGQTYDRRSIWRWME....EGH...CTCPKTGQLLSHN
lcl|Glyma.08G119700_287-357   .PKEFCCPISLDLMCDP.VIISTGQTYDRRSIWRWME....EGH...CTCPKTGLLVSHN
lcl|Glyma.13G225900_291-361   .PKDFCCPISLDLMRDP.VIISTGQTYDRSSISRWME....EGH...TTCPKTGQMLAHT
lcl|Glyma.15G086200_291-361   .PKDFCCPISLDLMRDP.VIISTGQTYDRSSISRWME....EGH...TTCPKTGQILAHT
lcl|Glyma.01G195200_65-135    .PSVFICPISLEPMQDP.ITLCTGQTYERSNILKWFN....LGH...FTCPTTMQELWDD
lcl|Glyma.11G046500_130-200   .PSVFICPISLEPMQDP.VTLCTGQTYERSNILKWFN....LGH...FTCPTTMQELWDD
lcl|Glyma.05G107300_60-130    .PSVFICPISLEPMQDP.VTLCTGQTYERCNILKWFS....LGH...FTCPTTMQELWDG
lcl|Glyma.06G270100_64-125    .....ICPISLEPMHDL.ATLCIGQTYERCNILKWFS....LDH...FTCLTTMQELWDD
lcl|Glyma.04G047100_37-103    .PSVFICPISLEPMLDP.VTLCTGQTYDRSNILRWFS....LGH...NTCPTTMQELWDD
lcl|Glyma.06G047800_39-109    .PSVFICPISLEPMQDP.VTLCTGQTYDRSNILKWFS....LGH...NTCPTTMQELWDD
lcl|Glyma.17G233600_35-105    .PSVFICPISHEPMQDP.VTLCTGQTYDRSNILKWFS....LGH...KTCPTTMQELWDD
lcl|Glyma.U029700_45-114      .PSVFICPISLEPMQDP.VTLCTGQTYDRSNILKWFS....LGH...KTCPTTMQELWDD
lcl|Glyma.04G212300_10-78     .PHLFRCPISLDLFEDP.VTLCTGQTYDRSSIEKWFS....TGN...LTCPVTMQKLHDP
lcl|Glyma.06G154100_10-78     .PHLFRCPISLDLFEDP.VTLCTGQTYDRSSIEKWFS....AGN...LTCPVTMQKLHDP
lcl|Glyma.05G192100_8-76      .PHLFRCPISLDLLEDP.VTLCTGQTYDRSNIEKWLA....LGN...LTCPVTMQKLHDP
lcl|Glyma.08G000400_8-76      .PHLFRCPISLDLFEDP.VTLCTGQTYDRSNIEKWLA....QGN...LTCPVTMQKLHDP
lcl|Glyma.02G083900_13-82     .PSLFRCPISMDVMRSP.VSLCTGVTYDRASIQRWLD....SGH...DTCPATLQVLPSK
lcl|Glyma.16G170300_13-80     .PSLFRCPISMDVMRSP.VSLCTGVTYDRASIQHWLD....SGH...DTCPATMQVLPSK
lcl|Glyma.05G189600_10-76     .PSFFRCPISLDVMKSP.VSLCTGVTYDRSSIQRWLD....NGN...NTCPATMQVLQTR
lcl|Glyma.08G147200_10-76     .PSFFRCPISLDVMKSP.VSLCTGVTYDRSSIQRWLD....NGN...NTCPATMQVLQTT
lcl|Glyma.06G151200_12-78     .PSFFKCPISLDVMKSP.VSLCTGVTYDRSSIQRWLD....AGN...NTCPATMQLLHTK
lcl|Glyma.09G030600_12-76     .PSFFKCPISLDIMKSP.VNLCTELTYNRFNIQRWLD....DGN...NTCPATMQLLPTK
lcl|Glyma.02G102900_14-85     .PYHFRCPISLELMRDP.VTVCTGQTYDRASIEAWVS....TGN...STCPVTRATLTDF
lcl|Glyma.07G214300_14-85     .PYHFRCPISLELMRDP.VTVCTGQTYDRASIESWVS....TGN...TTCPVTRATLSDF
lcl|Glyma.02G242900_33-104    .PTHFRCPVTLDMMKDP.VTVSTGITYDRDSIEKWIE....SGN...RTCPVTKTELTTF
lcl|Glyma.14G212200_33-105    .PTHFRCPVTLDMMKDP.VTVSTGITYDRDSIEKWIE....SGN...RTCPVTKTELTSL
lcl|Glyma.11G214500_30-101    .PNHFRCPVSLELMTDP.VTLSTGITYDRVSIEKWIE....GEN...RTCPVTNQVLTTF
lcl|Glyma.18G042100_29-100    .PNHFHCPVSLELMTDP.VTLSTGITYDRVSIEKWIE....GGN...RTCPVTNQVLTTF
lcl|Glyma.07G106000_27-99     .PNHFRCPISLDLMKDP.VTLSTGITYDRESVERWFD....EGN...ITCPVTNQVVRNF
lcl|Glyma.09G172100_28-99     ..NHFRCPISLDLMKDP.VTLSTGITYDRESVEMWFD....EGN...ITCPVTNQVVRNF
lcl|Glyma.02G195900_6-73      .PPFFVCPISLELMKDP.VTVSTGITYDRDSIEKWLF....AEV.KNDTCPVTKQPLLP.
lcl|Glyma.10G081900_6-74      .PPFFVCPISLELMKDP.VTVSTGITYDRHSIEKWLF....AAVPKNNTCPVTKQPLLP.
lcl|Glyma.03G202600_8-76      .PSFFLCPISLEIMKDP.VTVSTGITYDRESIETWLF....SKK..NTTCPMTKQPLIDY
lcl|Glyma.10G262600_6-74      .PSFFLCPISLEIMKDP.VTVSTGITYDRESIETWLF....SKK..NTTCPITKQPLIDY
lcl|Glyma.19G199300_6-74      .PSFFLCPISLDIMKDP.VTVSTGITYDRESIETWLF....SKK..NTTCPITKLPLIDY
lcl|Glyma.19G200200_6-74      .PSFFLCPISLDIMKDP.VTVSTGITYDRESIETWLF....SKK..NTTCPITKLPLIDY
lcl|Glyma.02G196300_6-78      .PQYFICPISLQIMKDP.VTAITGITYDRESIEQWLF....TNK..NTTCPVSNQPLPRD
lcl|Glyma.03G202500_6-78      .PKYFICPISLQIMKDP.VTTITGITYDRDSIEHWLF....TNK..STTCPITRQPLPKH
lcl|Glyma.12G095000_6-79      .PQYFVCPISFQIMEDP.VTTVTGITYDRESIEQWLL....KAK..DCVCPVSKQPLPRS
lcl|Glyma.U009600_6-79        .PQYFVCPISFQIMEDP.VTTVTGITYDRESIEKWLL....KAK..DCVCPVSKQPLPRS
lcl|Glyma.12G188900_14-88     .PQFFLCPISLQIMKDP.VTTVTGITYDRESIEKWLL....KAK..DCTCPITKQPLPRS
lcl|Glyma.13G312800_14-87     ..QFFLCPISLQIMKDP.VTTVTGITYDRESIEQWLL....KAK..DCTCPITKQRLPRS
lcl|Glyma.12G189000_6-73      .PAHFLCPISLQLMRDP.VTVCTGITYDRENIERWLF....S.CKNN.TCPVTKQCLLD.
lcl|Glyma.13G312700_6-73      .PAHFLCPISLQLMRDP.VTVCTGITYDRENIERWLF....S.CKNN.TCPVTKQCLLN.
lcl|Glyma.12G095100_6-75      .PTLFLCPISLQLMSDP.VTCAQEITYDRENIERWLF....SSCKKNKTCPVTRQSLPH.
lcl|Glyma.06G183800_276-346   .PEDFRCPISLEIMTDP.VTISSGQTYNRASIQKWFN....SGN...LICPKTREKLAST
lcl|Glyma.17G090900_270-341   ..DDFRCPISLELMTDP.VTVSTGQTYDRASIQKWLK....AGN...TKCPKTGEKLTNT
lcl|Glyma.02G034200_308-370   ...DFRCPISLELMSDP.VTIETGHTYDRSSILKWFS....SGN...LMCPKTGKRLSST
lcl|Glyma.01G173800_272-340   ...YYKCPISSRLMYDP.VIIESGVTYERIWIKKWFD....EGN...DICPKTRKKLVNM
lcl|Glyma.11G069300_271-339   ...EYKCPISSRLMYDP.VIIDSGVTYERMWIKKWFD....EGN...DICPKTRKKLVHM
lcl|Glyma.02G055500_272-341   ..KEYTCPISLRLMYDP.VVIASGKTYERMWIQKWFD....EGN...TICPKTKKKLVHM
lcl|Glyma.16G138300_272-341   ..EEYTCPISLRLMYDP.VVIASGKTYERMWIQKWFD....EGN...TICPKTKKELAHM
lcl|Glyma.05G146300_281-350   ..EELRCPISLQLMYDP.VIIASGQTYERVCIEKWFS....DGH...NNCPKTQQKLSHL
lcl|Glyma.08G103100_282-351   ..EELRCPISLQLMYDP.VTIASGQTYERVWIEKWFS....DGH...NNCPKTQQKLSHL
lcl|Glyma.11G249000_280-349   ..EELRCPISLQLMSDP.VIIASGQTYERICIEKWFR....DGH...NTCPKTQQKLSHL
lcl|Glyma.18G008300_283-352   ..EELRCPISLQLMSDP.VIIASGQTYERVCIEKWFR....DGH...NTCPKTQQKLSHL
lcl|Glyma.02G234300_262-332   .PADFCCPLSLELMMDP.VIVASGQTYERAFIKNWID....LGL...TVCPKTRQTLVHT
lcl|Glyma.11G161500_236-306   .PADFCCPLSLELMTDP.VIVASGQTYERAFIKNWID....LGL...TVCAKTRQTLVHT
lcl|Glyma.18G055200_239-309   .PADFCCPLSLELMTDP.VIVASGQTYERAFIKNWID....LGL...TVCPKTRQTLVHT
lcl|Glyma.03G163900_240-310   .PPYFRCPLSLELMSDA.VIVASGQTYERQSIQKWLD....HGL...TVCPNTRQILVHT
lcl|Glyma.19G165200_258-328   .PPYFRCPLSLELMSDP.VIVASGQTYERQSIQKWLD....HGL...TVCPNTHHRLVHT
lcl|Glyma.10G037500_241-311   .PLYFRCPLSLELMLDP.VIVASGQTYERQSIQKWLD....HGL...TVCPKTRQRLTPT
lcl|Glyma.09G256200_262-332   .PHEFLCPITLEIMTDP.VIVTSGQTYERESIEKWFQ....SNH...NTCPKTRQPLEHL
lcl|Glyma.18G236600_262-332   .PHEFLCPITLEIMTDP.VIVTSGQTYERESIKKWFQ....SNH...NTCPKTRQPLEHL
lcl|Glyma.02G001400_306-372   .....LCPITGAVMVDP.VSLCTGTTCERSAIEAWFD....DGNR...IDPETKEVLEDT
lcl|Glyma.10G179500_308-374   .....HCSITRNVMVDP.VSLCTGTTCERSAIEAWFC....DGNR...TDPETKEVLEDT
lcl|Glyma.04G016500_31-105    ...AFVCPLTKQVMRDP.VTLENGQTFEREAIEKWFKECRESGRRL..LCPLTLQELRST
lcl|Glyma.18G061900_252-321   ....FLCPLTKEVMRDPVVVLESSQAYERTAIEYWFERCIQDGRDP..TCPVTGRVLKSL
lcl|Glyma.07G052900_261-324   ....FYCPITQDVMVDP.VEISSGQTFERSAIEKWFA....EGNK...LCPLTLIPLDTS
lcl|Glyma.16G022000_261-324   ....FYCPITQDVMVDP.VEISSGQTFERSAIEKWFA....EGNK...LCPLTLIPLDTS
lcl|Glyma.06G132100_264-327   ....FYCPISLAIMADP.VETSSGKTFERREIEKWFA....EGNT...LCPLTRLPLDTK
lcl|Glyma.05G229600_445-515   ..KDFVCPITSYIFDDP.VTLETGQTYERKAIEEWFN....RGNL...TCPITRQKLQNT
lcl|Glyma.08G037000_392-462   ..KDFVCPITSHIFDDP.VTLETGQTYERKAIEEWFN....RGNI...TCPITRQKLQNT
lcl|Glyma.10G194500_509-579   ..KDFVCPITGQIFCDP.VTLETGQTYERKAIQEWLR....TGNT...TCPITRQPLSAN
lcl|Glyma.08G361400_607-678   .PHEFICPLTGDLFEEP.VTLETGQTFEREAIKAWFE....KGNR...TCPVTGNNLECV
lcl|Glyma.18G300300_613-683   .PHEFICPLTGNLFEEP.VTLETGQTFEREAIKAWFE....KGNR...TCPVTGNTLECV
lcl|Glyma.01G022600_729-795   .PSVFLCPILQEVMKNP.HVAADGFSYELEAIEHWLQ....SGR...DTSPVTNLRLKHT
lcl|Glyma.09G199700_729-794   ..SVFLCPILQEAMTNP.HVAADGFSYELEAIEHWLQ....SGR...DTSPMTNLRLKHT
lcl|Glyma.18G147800_727-788   .....ICPILQRIMKNP.HIAADGFSYELEAIEEWLQ....SGH...DISPK.NLKLKHK
lcl|Glyma.03G008800_809-878   ..PYFICPIFLEVMQDP.HVASDGFTYEAEAIREWLE....SGR...DTSPRTNSKLAHR
lcl|Glyma.07G070300_813-882   ..PYFICPIFLEVMQDP.HVAADGFTYEAEAIREWLE....SGH...DTSPRTNSKLAHR
```

```
lcl|Glyma.09G259100_814-883   ..SYFICPIFQEVMRDP.HVAADGFTYEAEAIRGWLD....GGH...DNSPMTNSKLAHH
lcl|Glyma.18G233500_814-883   ..SYFICPIFQEVMRDP.HVAADGFTYEAEAIRGWLD....GGH...DNSPMTNSKLAHH
lcl|Glyma.13G335700_751-820   .PSFFSCPILQEIMHDP.QVAADGFTYEGDAIREWLE....NGH...DTSPMTNLKLSHL
lcl|Glyma.15G038600_747-816   .PSFFSCQILLEIMHDP.QVAADGFTYEGDAIREWLE....NGH...DTSPMTNLKLSHL
lcl|Glyma.11G139700_716-785   .PSFFLCPIFQEIMHDP.QVAADGFTYEGKAISEWLE....NGH...ETSPMTNLKLTHL
lcl|Glyma.10G233500_636-705   .PSHFVCPIVQEVMEDP.YIAADGFTYEEEAIRGWLN....SGH...DTSPMTNLKLDHT
lcl|Glyma.20G160900_669-738   .PSHFVCPIVQEVMEDP.YIAADGFTYEEEAIRGWLN....SGH...DTSPMTNLKLDHT
lcl|Glyma.13G106500_407-472   .PSQYYCPILQEIMDDP.YIAADGFTYEYIAIKAWLS....K.H...NVSPMTKLKLQYS
lcl|Glyma.17G052800_689-754   .PSQYYCPILQEIMDDP.YIAADGFTYEYVAIKAWLS....K.H...NVSPMTKLKLQHS
lcl|Glyma.04G121100_741-805   ..NHFICPILQDVMDDP.CVAADGYTYDRKAIEKWLE....E.N...DKSPMTNMALPHK
lcl|Glyma.06G317700_730-794   ..NHFICPILQDVMDDP.CVAADGYTYDRKAIEKWLE....E.N...HKSPMTNMALPHK
lcl|Glyma.04G179300_28-94     .PKEFTCPISGSLMSDP.VVVASGQTFERLAVQLCKD.....LN....FSPKLDDGTRPD
lcl|Glyma.06G185400_21-86     ..KEFTCPISGSLMSDP.VVVASGQTFERLAVQLCKD.....LN....FSPKLDDGTRPD
lcl|Glyma.13G069300_57-122    ..EEFLCPISRSLMFDP.VIVSSGHSYERSSVEACKN.....VN....FTPQLPDGTTPD
lcl|Glyma.19G013500_57-122    ..EEFLCPISHSLMFDP.VIVSSGHSFERSSVEACIN.....VN....FTPQLPDGTTPD
lcl|Glyma.04G222600_253-316   .......PVTGALMDDA.MILPCGHSFGGGGIEHAIR.....MK....ACCTCSQPTTEE
lcl|Glyma.06G142700_255-318   .......PVTGALMDDA.VILPCGHSFGGGGIQHVIR.....MK....ACCTCSQPTTEE
lcl|Glyma.01G194100_40-101    .....CCSLTFTPFEEP.VCTPDGSVFDNMNITPYIV....KYG...KH.PVNGAPLKHQ
lcl|Glyma.11G047800_40-101    .....CCSLTFTPFEEP.VCTPDGSVFDNMNITPYIV....KYG...KH.PVTGAPLKHQ
lcl|Glyma.03G088400_201-273   .PDYLCCRITLDIFHDP.VITPSGLTYERAVILEHLQ....KVG...KFDPITREPLDPS
lcl|Glyma.16G085300_110-182   .PDYLCCRITLDIFHDP.VITPSGLTYERAVILEHLQ....KVG...KFDPITREPLDPS
lcl|Glyma.U008400_249-321     .PDYLCCRITLDIFHDP.VITPSGLTYERAVILEHLQ....KVG...KFDPITREPLDPS
```

```
                                       β3          α2
AtPUB14                                →     ℓℓℓℓℓℓℓℓℓℓ
                                          60         70
                                           .          .
AtPUB14                          HA.GLTPNYVLKSLIALWCESN..
consensus                        D...LIPNLALKSAIQEW......
lcl|Glyma.13G059200_942-1013     M...LIPDDELKARIEEFVRSQE.
lcl|Glyma.19G027200_940-1011     M...LIPDDALKARIEEFVRSQE.
lcl|Glyma.10G208200_248-320      HT.ALTPNYVLKSLIALWCESNGI
lcl|Glyma.20G182600_247-319      HT.ALTPNYVLKSLIALWCESNGI
lcl|Glyma.11G140100_256-328      ST.VLTPNYVLRSLIAQWCEANGI
lcl|Glyma.12G063700_257-329      ST.VLTPNYVLRSLIAQWCEANGI
lcl|Glyma.18G176400_260-330      TS.ILIPNHALYGLISSWCEAN..
lcl|Glyma.03G253100_49-114       HT.ILTPNYLVRDMILQW......
lcl|Glyma.19G250700_62-127       HT.ILTPNYLVRDMILLW......
lcl|Glyma.20G220600_60-130       HS.ILTPNCFLQNMISLWCKEH..
lcl|Glyma.08G345800_78-143       HT.VLTPNHLIREMIEQW......
lcl|Glyma.18G155200_78-143       HT.VLTPNHLIREMIEQW......
lcl|Glyma.07G188400_8-72         HP.ALIPNHALRSLISNY......
lcl|Glyma.08G061100_8-72         HP.SLIPNHALRSLISNY......
lcl|Glyma.13G249400_8-71         HS.SLIPNHALRSLI.........
lcl|Glyma.15G064800_8-71         HS.SLIPNHALRSLI.........
lcl|Glyma.07G216200_275-347      T...LTPNYVLRSLISQWCIEHNI
lcl|Glyma.20G013200_272-344      T...LTPNYVLRSLISQWCIEHNI
lcl|Glyma.01G131800_273-345      E...LIPNRVLRNMIAAWCREQRI
lcl|Glyma.03G036700_271-343      D...LIPNRVLRNMITAWCREQRI
lcl|Glyma.05G162100_287-357      R...LVPNRALRNMIMQWCSAH..
lcl|Glyma.08G119700_287-357      R...LVPNRALRNLIMQWCSAH..
lcl|Glyma.13G225900_291-361      R...LVPNRALRNLIVKWCTAH..
lcl|Glyma.15G086200_291-361      R...LVLNRALRNLIVQWCTAH..
lcl|Glyma.01G195200_65-135       S...VTPNTTLYRLIHTWFSQK..
lcl|Glyma.11G046500_130-200      S...VTPNTTLYRLIHMWFSQK..
lcl|Glyma.05G107300_60-130       S...LTPNTTLHRLISTWFSQK..
lcl|Glyma.06G270100_64-125       S...LTPNTTLHCLIST.......
lcl|Glyma.04G047100_37-103       S...VTPNTTLHHFILSW......
lcl|Glyma.06G047800_39-109       S...VTPNTTLYHFILSWFSQK..
lcl|Glyma.17G233600_35-105       V...VTPNSTLSHLILTWFSQK..
lcl|Glyma.U029700_45-114         V...VTPNSTLSHLMLTWFSQ...
lcl|Glyma.04G212300_10-78        S...IVPNHTLRHLIDQWLQ....
lcl|Glyma.06G154100_10-78        S...IVPNHTLRHLINQWLQ....
lcl|Glyma.05G192100_8-76         S...IVPNHTLRHLIDQWLQ....
lcl|Glyma.08G000400_8-76         S...IVPNHTLRHLIDQWLQ....
lcl|Glyma.02G083900_13-82        D...FIPNLTLHRLIRLWLLS...
lcl|Glyma.16G170300_13-80        D...FIPNLTLHRLIRLWL.....
lcl|Glyma.05G189600_10-76        D...FVPNRTLQRLIQIW......
lcl|Glyma.08G147200_10-76        D...FVPNRTLQRLIQIW......
lcl|Glyma.06G151200_12-78        D...FIPNRTLQSLIQIW......
lcl|Glyma.09G030600_12-76        H...FIPNCTLQNLIQ........
lcl|Glyma.02G102900_14-85        T...LIPNHTLRRLIQEWCVANR.
lcl|Glyma.07G214300_14-85        T...LIPNHTLRRLIQEWCVANR.
lcl|Glyma.02G242900_33-104       D...MIPNHAIRRMIQDWCVEHR.
lcl|Glyma.14G212200_33-105       DD..MIPNHAIRRMIQDWCVEHR.
lcl|Glyma.11G214500_30-101       D...LIPNHAIRMMIQDWCVQNS.
lcl|Glyma.18G042100_29-100       D...IIPNHAIRRMIQDWCVENS.
lcl|Glyma.07G106000_27-99        D...MIPNHSLRIMIQDWCVENRQ
lcl|Glyma.09G172100_28-99        D...MIPNHSLRVMIQDWCVENRQ
lcl|Glyma.02G195900_6-73         .D..LTPNHTLRRLIQAW......
lcl|Glyma.10G081900_6-74         .D..LTPNHTLRRLIQAW......
lcl|Glyma.03G202600_8-76         TD..LTPNHTLRRLIQAW......
lcl|Glyma.10G262600_6-74         TD..LTPNHTLRRLIQSW......
lcl|Glyma.19G199300_6-74         TD..LTPNHTLRRLIQAW......
lcl|Glyma.19G200200_6-74         TD..LTPNHTLRRLIQAW......
lcl|Glyma.02G196300_6-78         SD..LTPNHTLRRLIQAWCTQN..
lcl|Glyma.03G202500_6-78         SD..LTPNHTLLRLIQFWCTQN..
lcl|Glyma.12G095000_6-79         SQ.YLTPNHTLRRLIQAWCSAN..
lcl|Glyma.U009600_6-79           SQ.YLTPNHTLRRLIQAWCSAN..
lcl|Glyma.12G188900_14-88        PE.FLTPNHTLRRLIQAWCSANE.
lcl|Glyma.13G312800_14-87        TE.FLTPNHTLRRLIQAWCSANE.
lcl|Glyma.12G189000_6-73         HG..LTPNHTLRRLIQSW......
lcl|Glyma.13G312700_6-73         HD..LTPNHTLRRLIQSW......
lcl|Glyma.12G095100_6-75         TD..LTPNHTLQRLIQAW......
lcl|Glyma.06G183800_276-346      E...LVPNTALKKLIQKFCSEN..
lcl|Glyma.17G090900_270-341      D...LVPNTTLKRLIQQFCADNGI
lcl|Glyma.02G034200_308-370      E...MVPNLVLRRLIQ........
lcl|Glyma.01G173800_272-340      G...LTPNMAMKDLISEWCKNN..
lcl|Glyma.11G069300_271-339      G...LTPNMAMKDLISKWCRNN..
lcl|Glyma.02G055500_272-341      A...LTPNIALKDLILKWCETN..
lcl|Glyma.16G138300_272-341      A...LTPNVALKDLILNWCKTN..
lcl|Glyma.05G146300_281-350      C...LTPNYCVKGLVSSWCEQN..
lcl|Glyma.08G103100_282-351      C...LTPNYCVKGLVASWCEQN..
lcl|Glyma.11G249000_280-349      C...LTPNYCVKGLVASWCEQN..
lcl|Glyma.18G008300_283-352      C...LTPNYCVKGLVASWCEQN..
lcl|Glyma.02G234300_262-332      N...LIPNYTVKALIANWCESN..
lcl|Glyma.11G161500_236-306      N...LIPNYTVKALIANWCESN..
lcl|Glyma.18G055200_239-309      H...LIPNYTVKALIANWCESN..
lcl|Glyma.03G163900_240-310      N...LIPNYTVKAMIANWCEEN..
lcl|Glyma.19G165200_258-328      N...LIPNYTVKAMIANWCEEN..
lcl|Glyma.10G037500_241-311      N...LIPNYTVKAMIATWCEEN..
lcl|Glyma.09G256200_262-332      S...LAPNCALKSLIEEWCENN..
lcl|Glyma.18G236600_262-332      S...LAPNRALKSLIEEWCENN..
lcl|Glyma.02G001400_306-372      TL...RSNVRLRESIEEWREVN..
lcl|Glyma.10G179500_308-374      TL...RSNIPLRQSIEEWRELN..
lcl|Glyma.04G016500_31-105       EL...NPSMALRNTIEEWTARNE.
lcl|Glyma.18G061900_252-321      EL...KPNIGLAGAIEEW......
lcl|Glyma.07G052900_261-324      IL...RPNKKLKQSIQEW......
lcl|Glyma.16G022000_261-324      IL...RPNKKLKQSIQEW......
lcl|Glyma.06G132100_264-327      IL...RPNKTLKQSIQEW......
lcl|Glyma.05G229600_445-515      QLP..KTNYVLKRLIASWKDRN..
lcl|Glyma.08G037000_392-462      QLP..KTNYVLKRLIASWKDRN..
lcl|Glyma.10G194500_509-579      TLP..KTNYVLKRLITSWKEQN..
lcl|Glyma.08G361400_607-678      TMP..FTNLILKRLIDNWKSER..
lcl|Glyma.18G300300_613-683      TMP..FTNLILKRLIDTWKSE...
lcl|Glyma.01G022600_729-795      F...LTPNHTLRSLIEDW......
lcl|Glyma.09G199700_729-794      F...LTPNHTLRSLIQDW......
lcl|Glyma.18G147800_727-788      L...LTPNHTLRSLIEDW......
lcl|Glyma.03G008800_809-878      N...LVPNHALRHAIQNWLQSH..
lcl|Glyma.07G070300_813-882      H...LVPNHTLRHAIQNWLQSH..
```

```
lcl|Glyma.09G259100_814-883   N...LVPNRALRSAIQDWLQNH..
lcl|Glyma.18G233500_814-883   N...LVPNRALRSAIQDWLQNH..
lcl|Glyma.13G335700_751-820   F...LTPNYALRLAIQDWLCK...
lcl|Glyma.15G038600_747-816   F...LTPNHALRLAIQDWLCK...
lcl|Glyma.11G139700_716-785   N...LTPNHALRLAIQGWLCK...
lcl|Glyma.10G233500_636-705   D...LVPNYALHNAILEWQQQ...
lcl|Glyma.20G160900_669-738   D...LVPNYALHNAILEWQQQ...
lcl|Glyma.13G106500_407-472   V...LTPNHTLRSAIQEW......
lcl|Glyma.17G052800_689-754   V...LTPNHTLRSAIQEW......
lcl|Glyma.04G121100_741-805   H...LIPNYTLLSAILEW......
lcl|Glyma.06G317700_730-794   H...LIPNYTLLSAILEW......
lcl|Glyma.04G179300_28-94     FS.TIIPNLAIKTTILHW......
lcl|Glyma.06G185400_21-86     FS.TLIPNLAIKTTILHW......
lcl|Glyma.13G069300_57-122    FS.TLIPNLALKSAILKW......
lcl|Glyma.19G013500_57-122    FS.TLIPNLALKSAILKW......
lcl|Glyma.04G222600_253-316   S...ISPNLSLRIAVQAYRREEE.
lcl|Glyma.06G142700_255-318   S...ISPNLSLRIAVQAYRREEE.
lcl|Glyma.01G194100_40-101    D...LIP.LTFHKNSEGEY.....
lcl|Glyma.11G047800_40-101    D...LIS.LTFHKNSEGEY.....
lcl|Glyma.03G088400_201-273   Q...LVPNLAIKEAVEAFLDKHGW
lcl|Glyma.16G085300_110-182   Q...LVPNLAIKEAVEAFLDKHGW
lcl|Glyma.U008400_249-321     Q...LVPNLAIKEAVEAFLDKHGW
```
