# Supplementary material for: Genome-wide analysis of genes encoding core components of the ubiquitin system in soybean (Glycine max) reveals a potential role for ubiquitination in host immunity against soybean cyst nematode
Source: BMC Plant Biol. 2018 Jul 18;18:149. doi: 10.1186/s12870-018-1365-7 (PMC6052599; doi:10.1186/s12870-018-1365-7)
Supplement: Supplementary file 9 — Figure S6. Multiple sequence alignments of the F-box domain from the soybean F-box domain-containing proteins. (PDF 52 kb) [file 12870_2018_1365_MOESM9_ESM.pdf]

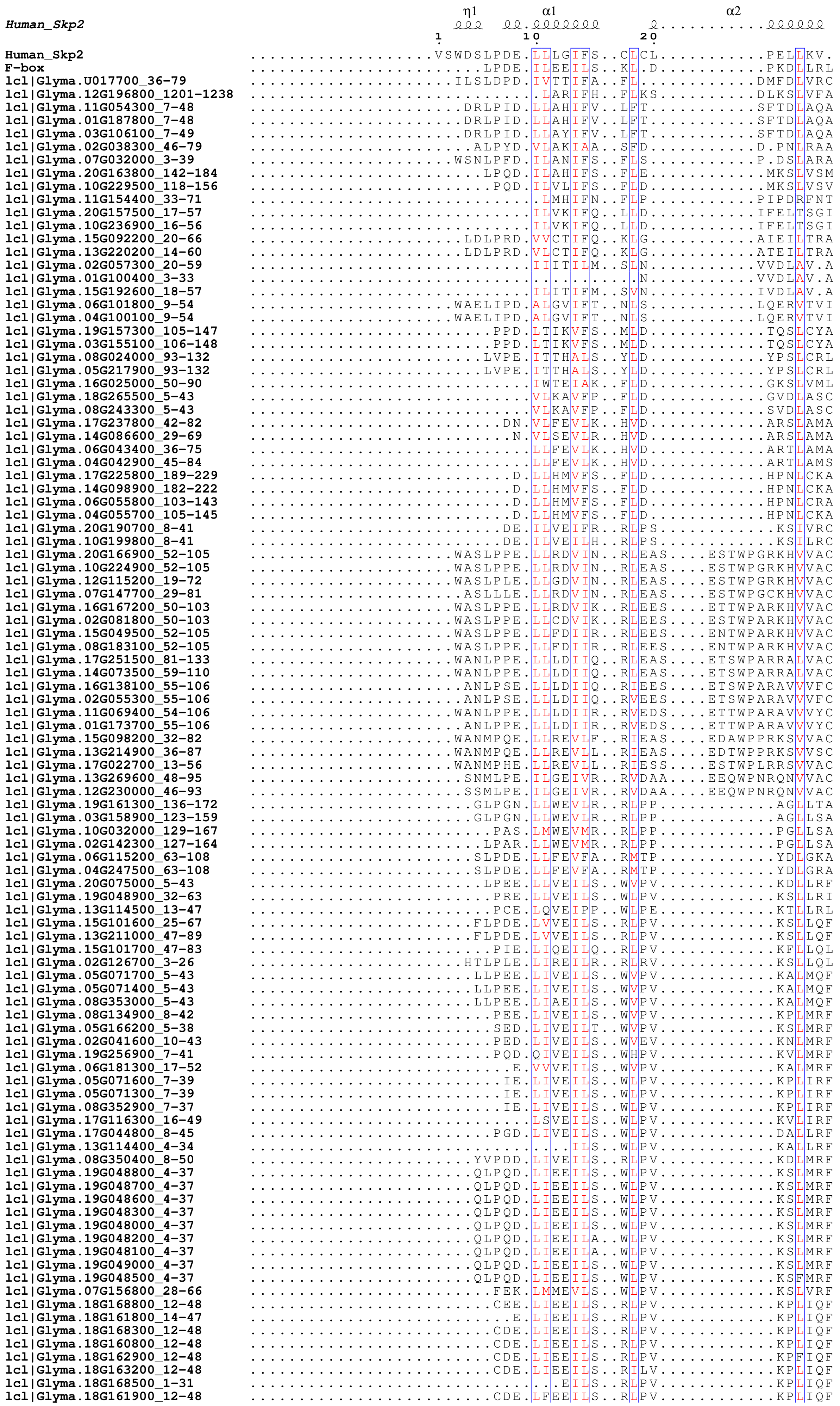
Human_Skp2
η1
α1
α2
1
10
20
Human_Skp2 VSWDSLPDE.LLLGIFS..CLCL...........PELLKV.
F-box LPDE.ILEEILS..KL.D...........PKDLLRL
lcl|Glyma.U017700_36-79 ILSLDPD.IVTTIFA..FL............DMFDLVRC
lcl|Glyma.12G196800_1201-1238 .LARIFH..FLKS..........DLKSLVFA
lcl|Glyma.11G054300_7-48 DRLPID.LLAHIFV..LFT..........SFTDLAQA
lcl|Glyma.01G187800_7-48 DRLPID.LLAHIFV..LFT..........SFTDLAQA
lcl|Glyma.03G106100_7-49 DRLPID.LLAYIFV..LFT..........SFTDLAQA
lcl|Glyma.02G038300_46-79 ALPYD.VLAKIAA..SFD..........D.PNLRAA
lcl|Glyma.07G032000_3-39 WSNLPFD.ILANIFS..FLS..........P.DSLARA
lcl|Glyma.20G163800_142-184 LPQD.ILAHIFS..FLE...........MKSLVSM
lcl|Glyma.10G229500_118-156 PQD.ILVLIFS..FLD...........MKSLVSV
lcl|Glyma.11G154400_33-71 .LMHIFN..FLP..........PIPDRFNT
lcl|Glyma.20G157500_17-57 ILVKIFQ..LLD..........IFELTSGI
lcl|Glyma.10G236900_16-56 ILVKIFQ..LLD..........IFELTSGI
lcl|Glyma.15G092200_20-66 LDLPRD.VVCTIFQ..KLG.........AIEILTRA
lcl|Glyma.13G220200_14-60 LDLPRD.VLCTIFQ..KLG.........ATEILTRA
lcl|Glyma.02G057300_20-59 IIITILM..SLN.........VVDLAV.A
lcl|Glyma.01G100400_3-33 ..........N.........VVDLAV.A
lcl|Glyma.15G192600_18-57 ILITIFM..SVN.........IVDLAV.A
lcl|Glyma.06G101800_9-54 WAELIPD.ALGVIFT..NLS..........LQERVTVI
lcl|Glyma.04G100100_9-54 WAELIPD.ALGVIFT..NLS..........LQERVTVI
lcl|Glyma.19G157300_105-147 PPD.LTIKVFS..MLD...........TQSLCYA
lcl|Glyma.03G155100_106-148 PPD.LTIKVFS..MLD...........TQSLCYA
lcl|Glyma.08G024000_93-132 LVPE.ITTHALS..YLD...........YPSLCRL
lcl|Glyma.05G217900_93-132 LVPE.ITTHALS..YLD...........YPSLCRL
lcl|Glyma.16G025000_50-90 IWTEIAK..FLD...........GKSLVML
lcl|Glyma.18G265500_5-43 VLKAVFP..FLD...........GVDLASC
lcl|Glyma.08G243300_5-43 VLKAVFP..FLD...........SVDLASC
lcl|Glyma.17G237800_42-82 DN.VLFEVLK..HVD...........ARSLAMA
lcl|Glyma.14G086600_29-69 N.VLSEVLR..HVD...........ARSLAMA
lcl|Glyma.06G043400_36-75 LLFEVLK..HVD...........ARTLAMA
lcl|Glyma.04G042900_45-84 LLFEVLK..HVD...........ARTLAMS
lcl|Glyma.17G225800_189-229 D.LLHMVFS..FLD...........HPNLCKA
lcl|Glyma.14G098900_182-222 D.LLHMVFS..FLD...........HPNLCKA
lcl|Glyma.06G055800_103-143 D.LLHMVFS..FLD...........HPNLCRA
lcl|Glyma.04G055700_105-145 D.LLHMVFS..FLD...........HPNLCKA
lcl|Glyma.20G190700_8-41 DE.ILVEIFR..RLPS..........KSIVRC
lcl|Glyma.10G199800_8-41 DE.ILVEILH..RLPS..........KSILRC
lcl|Glyma.20G166900_52-105 WASLPPE.LLRDVIN..RLEAS....ESTWPGRKHVVAC
lcl|Glyma.10G224900_52-105 WASLPPE.LLRDVIN..RLEAS....ESTWPGRKHVVAC
lcl|Glyma.12G115200_19-72 WASLPLE.LLGDVIN..RLEAS....ESTWPGRKHVVAC
lcl|Glyma.07G147700_29-81 ASLLLE.LLRDVIN..RLEAS....ESTWPGCKHVVAC
lcl|Glyma.16G167200_50-103 WASLPPE.LLRDVIK..RLEES....ETTWPARKHVVAC
lcl|Glyma.02G081800_50-103 WASLPPE.LLCDVIK..RLEES....ESTWPARKHVVAC
lcl|Glyma.15G049500_52-105 WASLPPE.LLFDIIR..RLEES....ENTWPARKHVVAC
lcl|Glyma.08G183100_52-105 WASLPPE.LLFDIIR..RLEES....ENTWPARKHVVAC
lcl|Glyma.17G251500_81-133 WANLPPE.LLLDIIQ..RLEAS....ETSWPARRALVAC
lcl|Glyma.14G073500_59-110 WANLPPE.LLLDIIQ..RLEAS....ETSWPARRALVAC
lcl|Glyma.16G138100_55-106 ANLPSE.LLLDIIQ..RIEES....ETSWPARAVVFC
lcl|Glyma.02G055300_55-106 ANLPSE.LLLDIIQ..RVEES....ETSWPARAVVFC
lcl|Glyma.11G069400_54-106 WANLPPE.LLLDIIR..RVEDS....ETTWPARAVVYC
lcl|Glyma.01G173700_55-106 ANLPPE.LLLDIIR..RVEDS....ETTWPARAVVYC
lcl|Glyma.15G098200_32-82 WANMPQE.LLREVLF..RIEAS....EDAWPPRKSVVAC
lcl|Glyma.13G214900_36-87 WANMPQE.LLREVLL..RIEAS....EDTWPPRKSVVSC
lcl|Glyma.17G022700_13-56 WANMPHE.LLREVLL..RIESS....ESTWPLRRSVVAC
lcl|Glyma.13G269600_48-95 SNMLPE.ILGEIVR..RVDAA....EEQWPNRQNVVAC
lcl|Glyma.12G230000_46-93 SSMLPE.ILGEIVR..RVDAA....EEQWPNRQNVVAC
lcl|Glyma.19G161300_136-172 GLPGN.LLWEVLR..RLPP...........AGLLTA
lcl|Glyma.03G158900_123-159 GLPGN.LLWEVLR..RLPP...........AGLLSA
lcl|Glyma.10G032000_129-167 PAS.LMWEVMR..RLPP...........PGLLSA
lcl|Glyma.02G142300_127-164 LPAR.LLWEVMR..RLPP...........PGLLSA
lcl|Glyma.06G115200_63-108 SLPDE.LLFEVFA..RMTP...........YDLGKA
lcl|Glyma.04G247500_63-108 SLPDE.LLFEVFA..RMTP...........YDLGRA
lcl|Glyma.20G075000_5-43 LPEE.LLVEILS..WVPV...........KDLLRF
lcl|Glyma.19G048900_32-63 PRE.LLVEILS..WLPV...........KSLLRI
lcl|Glyma.13G114500_13-47 PCE.LQVEIPP..WLPE...........KTLLRL
lcl|Glyma.15G101600_25-67 FLPDE.LVVEILS..RLPV...........KSLLQF
lcl|Glyma.13G211000_47-89 FLPDE.LVVEILS..RLPV...........KSLLQF
lcl|Glyma.15G101700_47-83 PIE.LIQEILQ..RLPV...........KFLLQL
lcl|Glyma.02G126700_3-26 HTLPLE.LIREILR..RLRV...........KSLLQL
lcl|Glyma.05G071700_5-43 LLPEE.LIVEILS..WVPV...........KALMQF
lcl|Glyma.05G071400_5-43 LLPEE.LIVEILS..WVPV...........KALMQF
lcl|Glyma.08G353000_5-43 LLPEE.LIAEILS..WVPV...........KALMQF
lcl|Glyma.08G134900_8-42 PEE.LIVEILS..WVPV...........KPLMRF
lcl|Glyma.05G166200_5-38 SED.LIVEILT..WVPV...........KSLMRF
lcl|Glyma.02G041600_10-43 PED.LIVEILS..WVEV...........KNLMRF
lcl|Glyma.19G256900_7-41 PQD.QIVEILS..WHPV...........KVLMRF
lcl|Glyma.06G181300_17-52 E.VVVEILS..WVPV...........KALMRF
lcl|Glyma.05G071600_7-39 IE.LIVEILS..WLPV...........KPLIRF
lcl|Glyma.05G071300_7-39 IE.LIVEILS..WLPV...........KPLIRF
lcl|Glyma.08G352900_7-37 IE.LIVEILS..WLPV...........KPLIRF
lcl|Glyma.17G116300_16-49 LSVEILS..WLPV...........KVLIRF
lcl|Glyma.17G044800_8-45 PGD.LIVEILS..WLPV...........DALLRF
lcl|Glyma.13G114400_4-34 ....ILS..WLPV...........KALLRF
lcl|Glyma.08G350400_8-50 YVPDD.LIVEILS..RLPV...........KDLMRF
lcl|Glyma.19G048800_4-37 QLPQD.LIEEILS..WLPV...........KSLMRF
lcl|Glyma.19G048700_4-37 QLPQD.LIEEILS..WLPV...........KSLMRF
lcl|Glyma.19G048600_4-37 QLPQD.LIEEILS..WLPV...........KSLMRF
lcl|Glyma.19G048300_4-37 QLPQD.LIEEILS..WLPV...........KSLMRF
lcl|Glyma.19G048000_4-37 QLPQD.LIEEILS..WLPV...........KSLMRF
lcl|Glyma.19G048200_4-37 QLPQD.LIEEILA..WLPV...........KSLMRF
lcl|Glyma.19G048100_4-37 QLPQD.LIEEILA..WLPV...........KSLMRF
lcl|Glyma.19G049000_4-37 QLPQD.LIEEILS..WLPV...........KSLMRF
lcl|Glyma.19G048500_4-37 QLPQD.LIEEILS..WLPV...........KSFMRF
lcl|Glyma.07G156800_28-66 FEK.LMMEVLS..WLPV...........KSLVRF
lcl|Glyma.18G168800_12-48 CEE.LIEEILS..RLPV...........KPLIQF
lcl|Glyma.18G161800_14-47 E.LIEEILS..RLPV...........KPLIQF
lcl|Glyma.18G168300_12-48 CDE.LIEEILS..RLPV...........KPLIQF
lcl|Glyma.18G160800_12-48 CDE.LIEEILS..RLPV...........KPLIQF
lcl|Glyma.18G162900_12-48 CDE.LIEEILS..RLPV...........KPFIQF
lcl|Glyma.18G163200_12-48 CDE.LIEEILS..RILV...........KPLIQF
lcl|Glyma.18G168500_1-31 ...EILS..RLPV...........KPLIQF
lcl|Glyma.18G161900_12-48 CDE.LFEEILS..RLPV...........KPLIQF

```
lcl|Glyma.18G162500_12-48     ..........................CDE.LIKEILS..RLPV.............KPLIQF
lcl|Glyma.18G160700_1-30      .................................EILS..RLPV.............KPLIQF
lcl|Glyma.18G162400_12-48     ..........................CDE.LIKEILS..RLPV.............KPLIQF
lcl|Glyma.18G161500_14-48     ............................E.LIKEILS..CLPV.............KTLIQF
lcl|Glyma.18G162700_12-49     ..........................CDE.LFEEILS..RLPV.............KPLMQF
lcl|Glyma.18G162600_11-49     ........................LLCNE.IIKEILS..RLPV.............KPLIQF
lcl|Glyma.18G162300_14-50     ..........................CNE.IIKEILS..RLPV.............KPLIQF
lcl|Glyma.18G168000_8-37      ..................................ILS..RLPM.............KPLIQF
lcl|Glyma.18G163100_10-47     ........................LLCNK.IIEEILS..RFPV.............KPLIQF
lcl|Glyma.14G160200_8-46      ......................LSLLCNE.IIEEILS..RLPV.............KPLIQF
lcl|Glyma.18G162800_10-46     ........................LLCNE.IIEEILS..RLPV.............KPLIPF
lcl|Glyma.18G276400_6-41      ...........................RE.LTEKILI..KLPV.............KSLVSF
lcl|Glyma.20G073200_7-44      ..........................PFD.LIVEILL..RLSV.............RSLLRF
lcl|Glyma.07G187200_13-47     ............................D.LTIEILL..RLPV.............RCLLRF
lcl|Glyma.16G203000_9-45      ..........................PED.LITEILM..MLPV.............RSILRF
lcl|Glyma.16G202600_12-48     ..........................PED.LITEILM..MLPV.............RSILRF
lcl|Glyma.16G202900_12-48     ..........................PED.LITEILM..MLPV.............RSILRF
lcl|Glyma.16G202800_1-34      ..............................LITEILM..MLPV.............RSILRF
lcl|Glyma.01G232700_12-48     ..........................PED.LITEILM..MLPV.............RSILRF
lcl|Glyma.10G117700_28-64     ..........................PLE.LIREILL..RLPV.............RSVLRF
lcl|Glyma.08G252000_10-33     ..........................PLE.LIREILL..RSPV.............RSVLRF
lcl|Glyma.08G252300_8-45      ..........................PLE.LIREVLL..RLPV.............RSVLRF
lcl|Glyma.18G274900_8-44      ..........................PLE.LIIEILM..RLPV.............RSVLGF
lcl|Glyma.07G148300_5-41      .........................LPLE.LIEEILL..RLPV.............RSILRF
lcl|Glyma.18G274700_8-46      ..........................PLD.LIELILL..KLPV.............KSVTRF
lcl|Glyma.03G116000_13-48     ...........................GE.LIGAILL..WLPV.............RSVLRF
lcl|Glyma.18G274500_6-42      ..........................PME.LMREILL..RLPV.............RSVSRC
lcl|Glyma.08G251800_6-42      ..........................PMD.LMREILL..RLPV.............RSVSRF
lcl|Glyma.17G162200_4-40      .........................LPDD.QIVEILL..RLPV.............RTLLRF
lcl|Glyma.10G132100_7-44      ..........................PDE.LIVEILL..RLPV.............RTLLRF
lcl|Glyma.06G197100_7-43      ..........................PDD.MMEEILL..RLPV.............KCLLRF
lcl|Glyma.06G197000_14-50     ..........................PDE.LMEEILL..RLPV.............RCLVRF
lcl|Glyma.10G220200_8-46      ......................LWLLPEE.LISEILF..RVPV.............RSLLQF
lcl|Glyma.02G188000_34-67     ............................E.LISNILH..RVPV.............RSLLQF
lcl|Glyma.10G220500_40-78     ........................VLPYD.LIWEILL..RVPV.............RSLILF
lcl|Glyma.06G016300_7-47      ........................MLPDD.LIVNILS..RLRV.............RSLMRS
lcl|Glyma.08G252200_30-68     ..........................PPE.LIREILL..SLPV.............NSLLQC
lcl|Glyma.06G197200_5-41      ..........................PEE.LIQVILL..RLPL.............RNLLHL
lcl|Glyma.18G274800_22-60     .........................LPQE.LIREILL..RLPV.............KSLLRF
lcl|Glyma.13G109000_20-53     ..........................PEE.LIIEILL..RLPV.............KSLLRF
lcl|Glyma.17G017600_31-68     .........................LPQE.LIHEILL..RLPV.............KSLIRF
lcl|Glyma.16G164000_14-50     ..........................PQE.LIIEILL..RLPV.............KSLVRF
lcl|Glyma.02G079600_14-50     ..........................PQE.LIIEILL..RLPV.............KSLVRF
lcl|Glyma.06G126800_15-54     .......................AILPWE.LIIEILL..RLPV.............KSLVRF
lcl|Glyma.17G027000_18-43     ..........................PQE.LIIQILL..RLPV.............KSLIRF
lcl|Glyma.07G247000_18-54     ..........................PQE.LIIQILL..RLPV.............KSLLRF
lcl|Glyma.20G014700_28-52     ..........................PLE.LITEIFL..RLPL.............KSLIRF
lcl|Glyma.17G021300_21-61     ..........................PQD.LVIQILL..RLPV.............KSLVRF
lcl|Glyma.08G098000_3-39      ..........................PQD.LITEILL..RLPV.............KSLVRF
lcl|Glyma.08G251700_6-43      ...........................HD.LIVEILL..RLPI.............KSLLRF
lcl|Glyma.18G256100_8-31      ..........................PIE.LIELILL..RLSV.............RHVIRF
lcl|Glyma.15G055200_11-42     ..........................PYD.VIINILK..RLPV.............KSLIRF
lcl|Glyma.09G032600_10-43     ...........................DE.VVIQILA..RLPV.............KSLFRF
lcl|Glyma.02G128400_30-69     .........................LPDEIMLFKILP..LLPS.............KTLIRF
lcl|Glyma.17G008200_11-50     .......................ANLPVE.VVTEILS..RLPV.............KSVIRL
lcl|Glyma.07G265800_3-43      ........................NLPVE.VVTEILS..RLPV.............KSVIRL
lcl|Glyma.15G115100_4-44      .........................LPRE.VLTEILS..RLPV.............RSLLRF
lcl|Glyma.09G010600_4-42      .........................LPRE.VVTDILS..RLPA.............KSLLRF
lcl|Glyma.06G045700_5-48      ......................WSKLPPD.VVEHILL..LLPL.............KTLLNL
lcl|Glyma.04G045200_5-49      ......................WSKLPPD.VVEHILL..LLPL.............KTLLNL
lcl|Glyma.14G088700_35-79     .......................SKLPPE.ILEYILS..FLPL.............KTFLNL
lcl|Glyma.19G081700_4-43      .........................LPQE.LVSNILS..RLPA.............IDLVKC
lcl|Glyma.16G063500_47-81     .........................LPQE.LVSNILS..RLPA.............KDLVKC
lcl|Glyma.19G081500_1-45      .....................SMEHLPRE.LVSNVLS..RLPA.............KVLLLC
lcl|Glyma.16G063600_1-45      .....................SMEHLPGE.LVSNVLS..RLPS.............KVLLLC
lcl|Glyma.15G220600_3-41      ........................YLPEA.LVLQILY..RLPP.............TTLVKC
lcl|Glyma.16G161700_9-36      ................................FEVFS..CLPA.............KAIHRF
lcl|Glyma.15G092300_5-37      ..........................DEL.LLFKICS..YLPA.............KAIYRF
lcl|Glyma.03G020200_4-34      ..........................SHD.ELLEIFS..RLPA.............KAIYRF
lcl|Glyma.04G198800_33-68     .........................LCND.ALFEIFL..RLPP.............EVLPRF
lcl|Glyma.09G075900_20-65     ......................INNLFDD.SLTEIFC..KLPC.............KSLFTC
lcl|Glyma.07G022100_3-47      ......................INNLSDD.VMGEIFI..RLPF.............RSTVKC
lcl|Glyma.02G083400_9-46      .........................LAED.SLRQIFC..RLPL.............REIMIC
lcl|Glyma.19G252100_40-81     .......................DVLPDD.LLERILA..YLPI.............ASIFRA
lcl|Glyma.03G254500_40-81     .......................DVLPDD.LLERILA..YLPI.............ASIFRA
lcl|Glyma.08G088700_46-81     ......................WSKLPQR.LLDRVLA..FLPP.............PAFFRA
lcl|Glyma.08G104800_104-143   .......................KKLPED.LFEPVIA..RLPI.............ATFFCF
lcl|Glyma.05G147900_105-145   .......................KNLPED.LFEPVIA..RLPI.............ATFFRF
lcl|Glyma.18G005400_113-153   .......................KDFPED.LFEAVIA..RLPI.............ATFFRF
lcl|Glyma.17G220600_21-64     .....................SLDDLNED.LLERVLS..WLPT.............SSFFRL
lcl|Glyma.14G105800_21-64     .....................SLDDLNED.LLERVLS..WLPT.............SSFFRL
lcl|Glyma.06G058900_28-68     .....................SLDDLNED.LFERILS..WLQT.............STFFRL
lcl|Glyma.04G058200_28-68     .....................SLDDLNED.LFERILS..WLPT.............STFFRL
lcl|Glyma.18G275900_100-142   .......................AMLPED.LLHEILA..RVPP.............FLIFRL
lcl|Glyma.08G253600_95-137    .......................AMLPED.LLHEILA..RVPP.............FLIFRL
lcl|Glyma.13G209700_23-63     ..........................PED.LMIEILA..RVRV.............SNPLQL
lcl|Glyma.06G168700_28-71     ......................LSMLNED.LLQNILA..RLPS.............LHFASA
lcl|Glyma.04G196900_27-69     ......................LSMLNED.LLQNILA..RLPA.............LHFASA
lcl|Glyma.19G162400_18-54     .......................PGLPND.VAASILS..MVPY.............SHHGRL
lcl|Glyma.03G160600_17-56     .......................PGLPND.VAASILS..KVPY.............SHHGRL
lcl|Glyma.19G158300_6-45      ........................GLPDA.VAIRCLA..RVPF.............YLHPVL
lcl|Glyma.03G156000_5-45      .......................ERLPDA.VAIRCLA..RVPF.............YFHPVL
lcl|Glyma.10G028000_6-45      ........................GLPDA.VAIRCLA..WVPF.............YLHPKL
lcl|Glyma.06G146500_19-60     .......................PSLPDD.VALNCLG..RIPR.............SQHPTL
lcl|Glyma.04G219400_21-60     ..........................PND.VALNCLA..RIPR.............SHHPTL
lcl|Glyma.07G071600_25-62     .......................CGLPDD.ISLMCLA..RIPR.............KYHSVL
lcl|Glyma.03G010000_2-33      ...............................SLMCLA..RIPR.............KYHSVL
lcl|Glyma.07G071500_32-72     .......................CGLPDD.ISLMCLA..RIPR.............KYHSVM
lcl|Glyma.07G071400_25-62     .......................CGLPDD.LSLMCLA..RVPR.............KYHSVL
lcl|Glyma.08G112900_78-117    .......................PGLPDD.LAIACLI..RVPR.............VEHGKL
lcl|Glyma.05G155000_78-117    .......................PGLPDD.LAIACLI..RVPR.............VEHSKL
lcl|Glyma.19G005000_68-104    .......................PGLPDD.LAIACLI..RVPR.............VEHRKL
lcl|Glyma.09G019400_18-59     ......................LSGLPDD.LAIACLI..RVPR.............IEHRKL
```

```
lcl|Glyma.15G093300_53-91    ......................PGLPDD.VALNCLL..RLPV...........QSHSSC
lcl|Glyma.13G218800_53-91    ......................PGLPDD.VALNCLL..RLPV...........QSHSSC
lcl|Glyma.18G007900_34-73    ......................PGLPDD.VAEYCLA..LVPR...........SNFPAM
lcl|Glyma.11G249300_49-87    ......................PGLPDD.VAEYCLA..LVPR...........SNFPAM
lcl|Glyma.08G103400_52-91    ......................PGLPDD.VSKHCLA..LVPR...........SNFPAM
lcl|Glyma.15G013300_4-46     ......................SGLPED.VARDCLI..RVPY...........DQFPAV
lcl|Glyma.13G360600_4-47     ......................SGLPED.VARDCLI..RIPY...........EQFPAV
lcl|Glyma.08G207700_4-46     ......................SGLPED.VARDCLI..RVSY...........QQFPTV
lcl|Glyma.07G034400_4-36     ......................SGLPED.VARDCLI..RVSY...........QQFPTV
lcl|Glyma.20G045100_49-89    .....................ISSLPDD.IVLDCLS..RVPT...........SSLPAL
lcl|Glyma.05G155600_55-96    ......................PKLPSE.LGLECLT..RLPH...........SAHRVA
lcl|Glyma.06G016400_142-179  .........................PDD.ILEMCLV..RLPL...........TSLMNA
lcl|Glyma.04G016300_139-176  .........................PDD.ILEMCLV..RLPL...........TSLMNA
lcl|Glyma.19G246800_38-73    ...........................D.ILHEILL..RIPP...........PTISKL
lcl|Glyma.03G249300_21-56    ...........................D.ILHEILL..RIPP...........PTISKL
lcl|Glyma.06G076900_64-102   ......................PGLPYE.IAELCLL..HVPY...........PYQALS
lcl|Glyma.02G036400_11-44    .............................ILEAIFS..HVPL...........IHLVPA
lcl|Glyma.01G028800_12-45    .............................ILEAIFS..HVPL...........IHLVPA
lcl|Glyma.08G284600_18-52    .............................ILEAIFS..HVPL...........IHLVPA
lcl|Glyma.18G141000_17-47    .............................ILEAIFS..HVPL...........IHLVSA
lcl|Glyma.08G259900_8-42     .........................NSD.ILIEILS..HVPA...........KDLLSL
lcl|Glyma.01G083900_11-43    .............................ILIEILS..HVPA...........KDLLSL
lcl|Glyma.14G167900_43-84    ......................PNIPDE.LSLQIIA..RLPR...........ICYYHV
lcl|Glyma.13G086000_43-84    ......................PNLPDE.LSLQIIA..RLPR...........ICYYHV
lcl|Glyma.06G106800_43-84    ......................PNLPDE.LSLQIIA..RLPR...........ICYFNV
lcl|Glyma.04G107000_43-84    ......................PNLPDE.LSLQIIA..RLPR...........ICYFNV
lcl|Glyma.15G214000_43-89    ......................PNLPDD.LSLQIIA..RLPR...........ICYYHV
lcl|Glyma.08G074400_43-84    ......................PSLPDE.ISIQILA..RVPR...........IYYLNL
lcl|Glyma.05G119400_43-84    ......................PSLPDE.ISIQILA..RVPR...........IYYLNL
lcl|Glyma.08G298900_194-236  .......................RMNRD.SSIACLS..RCSR...........SDYGSL
lcl|Glyma.02G105600_194-236  .......................RMNRD.SSITCLS..RCSR...........SDYGSL
lcl|Glyma.13G326800_49-82    ........................ID.D.VALNCLA..WVSG...........SDYAVL
lcl|Glyma.20G215600_15-54    .....................LSGLPDE.ILLIIMS..FI.MI.........KDAVQT.
lcl|Glyma.10G175100_15-54    .....................LSGLPDE.ILFIIMS..FI.MI.........KDAVKT.
lcl|Glyma.17G078800_21-60    .....................LSNLPDD.IIDRVLY..FL.DA.........VSAVQT.
lcl|Glyma.07G004400_30-68    ......................SNLPDE.VLHRILS..TL.DA.........KSAVQT.
lcl|Glyma.16G177700_131-169  .....................ISELPDN.VLLHIMN..FV.DT.........KDAVKT.
lcl|Glyma.09G139100_17-56    .....................ISEFPDH.VLLHIMS..LM.DT.........KSAVRT.
lcl|Glyma.09G140100_13-51    .....................ISELPIS.VLLHILE..FM.NT.........KDAVQT.
lcl|Glyma.09G130200_31-69    .....................ISELPDS.ILLHILN..FM.NT.........ESAVQT.
lcl|Glyma.09G140200_31-69    .....................ISELPDS.VLLHIIE..FM.DT.........KSGVQT.
lcl|Glyma.13G224200_116-152  .....................ISALPDS.LLFHMIN..FM.DT.........KSAVQT.
lcl|Glyma.09G139800_14-52    .....................ISELPDN.ILLHMMN..FM.DT.........REAVQT.
lcl|Glyma.09G139700_14-52    .....................ISEMPDN.ILLHMMN..FM.DT.........REAVQT.
lcl|Glyma.09G139900_29-68    .....................ISELPDN.ILLHMMD..FM.DT.........REAVQT.
lcl|Glyma.10G136700_25-60    ........................LPEN.VLLHIMN..FM.ET.........RHAVQT.
lcl|Glyma.09G141700_24-62    .....................LSELPDC.VVLHIME..FM.DT.........KYAVQT.
lcl|Glyma.09G141600_32-70    .....................LSELPDC.VVLHIME..FM.DT.........KYAVQT.
lcl|Glyma.09G141500_32-70    .....................LSELPDC.VVLHIME..FM.DT.........KYAVQT.
lcl|Glyma.09G141400_32-70    .....................LSELPDC.VVLHIME..FM.DT.........KYAVQT.
lcl|Glyma.09G141300_32-70    .....................LSELPDC.VVLHIME..FM.DT.........KYAVQT.
lcl|Glyma.09G141200_32-70    .....................LSELPDC.VVLHIME..FM.DT.........KYAVQT.
lcl|Glyma.09G141100_32-70    .....................LSELPDC.VVLHIME..FM.DT.........KYAVQT.
lcl|Glyma.10G134800_20-58    .....................LSELPDC.ILLYIMK..FM.NT.........KYAVQT.
lcl|Glyma.09G142000_40-78    .....................LSDLPDF.VLLHIMK..FM.SM.........KHAVQT.
lcl|Glyma.10G135600_12-49    .....................LSELPDF.VLLHIMN..FI.DT.........KDALRT.
lcl|Glyma.10G135200_27-64    .....................LSELPDF.VLLHIMN..FI.DT.........KDALRT.
lcl|Glyma.10G135500_27-64    .....................LSELPDF.VLLHIMN..FI.YT.........KDALRT.
lcl|Glyma.09G130100_116-155  .....................ISELPDT.VLLHILN..FM.DT.........KDAVKT.
lcl|Glyma.06G098100_17-56    .....................LSDLPEC.VLLHILT..FL.NA.........KHAVRT.
lcl|Glyma.15G227900_18-56    .....................ISELPIH.VFLRILE..FM.NT.........RDAVRL.
lcl|Glyma.18G164400_4-43     .....................ISSLPDT.LLCHILS..FL.PT.........IESVAT.
lcl|Glyma.08G351300_7-40     .....................ISSLPDT.LLCHILS..FL.PT.........KEAIATT
lcl|Glyma.18G164500_7-43     .....................ISSLPNE.LLCHILS..FL.PT.........KQAVAT.
lcl|Glyma.13G353900_4-42     .....................FSSLTES.VLCYILS..FL.PT.........KDAVAT.
lcl|Glyma.18G164300_4-42     .....................ISNLPDV.VLSHILS..LV.PT.........NVAVAT.
lcl|Glyma.08G351400_4-43     .....................ISNLPDA.VLCHILS..FL.PT.........KQSIVT.
lcl|Glyma.18G164600_21-53    .....................ISHLPDV.LLLQILS..LL.PT.........KQAVIT.
lcl|Glyma.12G103400_25-61    .....................ISDLPDA.VLHQILF..LL.PI.........K.CVAQM
lcl|Glyma.13G283500_19-52    ......................STLPDS.VLVSIIS..LL.PC.........NEGVRT.
lcl|Glyma.15G241900_46-76    ......................SYLPEV.IIGRILF..FL.PN.........KVAVRT.
lcl|Glyma.15G241800_37-67    ......................SYIPDV.IIGRILF..FL.PN.........KDAVRT.
lcl|Glyma.15G241500_46-77    .....................ISYLSDV.IIGRILF..FL.PN.........KDTVRT.
lcl|Glyma.13G262900_18-55    .....................FSDLPDV.IIGRILS..IL.PT.........KEAVRT.
lcl|Glyma.17G193700_22-57    .....................ISNLPDF.IIGLILS..LL.PT.........KDAFRT.
lcl|Glyma.15G242400_9-60     ........LPPEQTVVEGEGIISKLHES.ILGKILS..FL.PT.........TDAVHT.
lcl|Glyma.15G242200_29-66    ......................SKLHES.ILGHILS..FL.PT.........MESVHT.
lcl|Glyma.15G242000_23-61    ......................SQIHDS.ILGHILS..FL.PT.........MEAVQT.
lcl|Glyma.13G262800_23-60    ......................SQIHDS.ILGHILS..FL.PT.........MEAVQT.
lcl|Glyma.20G142600_29-63    ......................GNLPNE.IIQHILS..LL.PT.........KDAVKT.
lcl|Glyma.15G022500_46-82    .....................ISQFPDH.VIHHILS..HLRNV.........NDAIRT.
lcl|Glyma.08G192100_57-96    .....................LSDMPDC.IIHHILS..FM.ET.........KDAIQT.
lcl|Glyma.13G278200_43-78    .....................ISSLPDE.IICHILS..FL.PT.........IEAVTT.
lcl|Glyma.07G072400_15-54    .....................ISELPDD.VVYHILS..FL.TI.........KEAIAT.
lcl|Glyma.08G348900_6-44     .....................ISALPDE.VLGHILS..FL.ST.........QEAIST.
lcl|Glyma.20G098700_4-40     .....................FSNLPDE.VLSCIVS..FL.PN.........ESALET.
lcl|Glyma.10G290500_5-34     ......................SNLPDE.ILSCIVS..FL.P.............LET.
lcl|Glyma.18G286800_5-41     .....................FSNLPDQ.ILCRIVS..FL.PN.........ESSLET.
lcl|Glyma.02G094600_5-41     .....................FSNLPDE.ILGRIVS..FL.PN.........ESSLET.
lcl|Glyma.02G127700_9-44     .....................ISDLPQS.IIESILV..QL.PI.........RDAVRT.
lcl|Glyma.01G072000_9-46     .....................ISDLPQS.IIESILV..QL.PI.........RDAVRT.
lcl|Glyma.02G127100_16-54    .....................ISDLPSH.LIDFILQ..RL.QL.........QDVVRT.
lcl|Glyma.02G126900_16-55    .....................ISDLPSH.LIDFILQ..RL.PL.........QDVVRT.
lcl|Glyma.17G247500_18-51    .....................ISCLPGH.VIDQVLS..HL.SI.........REAVRT.
lcl|Glyma.17G128800_29-62    ......................NKLPDG.IPVAILS..KL.PI.........NEAART.
lcl|Glyma.12G068200_27-62    ......................STMPDD.ILGDILS..RL.TL.........KEAART.
lcl|Glyma.08G195600_6-40     ......................STLPKT.ILHDILS..RM.PE.........EDAVRT.
lcl|Glyma.08G195400_12-48    ......................ENLPDI.VLHDILS..RL.PE.........KDAART.
lcl|Glyma.13G261600_7-44     ......................SNLPDE.IKTNILS..KL.CI.........DEAVRC.
lcl|Glyma.09G102300_7-43     .....................LSSLPEL.LCLFIIS..LL.PF.........KDAVRT.
lcl|Glyma.08G312700_7-45     .....................LSSLPEL.LCLFIIS..LL.PF.........KDAVRT.
lcl|Glyma.17G194900_31-63    .....................LSKLPEP.LVSHILS..FL.PT.........KDAVRT.
lcl|Glyma.10G200400_12-47    .......................LLPEI.VLITIVS..FL.PF.........KEAVRT.
```

```
lcl|Glyma.02G289100_7-44     .....................FCLLPIE.VVLTILS..LL.PF...........KEVVRT.
lcl|Glyma.02G289200_6-45     .....................ISSLPIQ.LLMTIVS..LL.PF...........KESMRA.
lcl|Glyma.06G120600_42-76    ......................KDIPVE.LLMQILS...LVDD...........QTVIIA.
lcl|Glyma.04G242700_1-26     ................................QILS...LVDD...........QTVITA.
lcl|Glyma.13G036600_43-77    ......................KDIPVE.LLMQILS...LVDD...........QTVMIA.
lcl|Glyma.12G088900_2-39     .......................QLPVE.VIGNILS..HLRAA...........RDVVIA.
lcl|Glyma.15G081900_5-39     ......................SNLPHD.LLSRISS..GLGL............IDFLSF.
lcl|Glyma.13G230700_4-39     .....................WSNLPRD.LVSQIVS..GLGL............IDFLSF.
lcl|Glyma.04G019900_5-47     ......................GELPPE.LLESISK..TLTIY...........VDYLRF.
lcl|Glyma.09G116200_72-107   .....................WSSLPRG.ILEMVAE..RLTF............IDCLSI.
lcl|Glyma.10G154000_52-81    .............................ILRLIFD..NLPI............PDLARA.
lcl|Glyma.08G356100_5-44     ......................TNLSTD.LIELILS..LLPI............PTLIRA.
lcl|Glyma.13G154400_35-73    .....................WSDLPTE.LLELILS..RLSL............DDNVRA.
lcl|Glyma.06G213000_16-54    .......................LLPQE.LIQNIFL..SLVL............PEIIRL.
lcl|Glyma.04G150400_60-99    .......................LLPQE.LIQNIFL..SLVL............PEIIRL.
lcl|Glyma.18G227100_18-56    ................................KILM..SLDD............LADLVRA
lcl|Glyma.16G197900_17-58    .............................MSIKILT..HLDD............PCDLIRV
lcl|Glyma.14G044300_96-140   .....................ITDLPPA.LISEILN..CLD.............PKDLGVV
lcl|Glyma.02G273700_95-139   .....................ITDLPPA.LISEILN..CLD.............PKDLGVV
lcl|Glyma.15G046900_4-35     .........................PDA.ILQYILS..RINN............ARDVAAC
lcl|Glyma.08G185900_4-35     .........................PDA.ILQYILS..HINN............ARDVAAC
lcl|Glyma.08G127900_2-36     .......................SLPDA.ILQCILS..RITN............ARDVSSC
lcl|Glyma.05G169600_2-36     .......................SMPDA.ILQCILS..RITN............ARDVSSC
lcl|Glyma.13G164200_19-62    ........................LPDE.ILCSILE..GLT.............PRDAARV
lcl|Glyma.06G101600_19-63    ........................LPDE.ILCSILE..RLT.............PRDAARV
lcl|Glyma.12G051100_14-48    ..................................LK..KLG.............AQDIARV
lcl|Glyma.15G092600_22-56    .......................RLPDS.LLLLVFN..KIGD............VKALGRC
lcl|Glyma.13G219800_25-59    .......................RLPDS.LLLLVFN..KIGD............VKALGRC
lcl|Glyma.17G251700_4-43     .......................DLPEG.CIAKILS..YT.............TPVDACRL
lcl|Glyma.14G073300_3-44     ......................QDLPEG.CIAKILS..YT.............TPVDVCRL
lcl|Glyma.20G220300_18-63    .....................INLLPEG.CISYILS..YT.............TPVDACRL
lcl|Glyma.20G220200_12-43    ................................KILS..YT.............TRADVCRL
lcl|Glyma.10G169300_5-46     ........................LPEG.CVAHILS..YIC............TPEDIVRL
lcl|Glyma.10G169600_3-46     ......................QGLPEG.CIASILS..RT.............TPADVCRF
lcl|Glyma.20G220100_9-52     ......................NNLPEG.CIANILS..FT.............SPRDVCRL
lcl|Glyma.10G169700_11-37    .............................CMVNILS..FT.............SPRDVRRL
lcl|Glyma.03G233900_4-44     .........................PYD.CFAHILS..FT.............STQDVCRS
lcl|Glyma.03G253900_24-65    .......................HLPEG.CIANIVS..FT.............TPPDACVL
lcl|Glyma.19G231000_9-54     ....................NIESLPHD.CVSEILS..HT.............SPLVACIV
lcl|Glyma.03G233800_10-55    ....................NIESLPHD.CVSEILS..HT.............SPLEACMV
lcl|Glyma.10G150400_4-45     .........................PED.CVSKILS..YT.............SPPDACRF
lcl|Glyma.06G271000_8-48     .........................PEE.CVATIIS..LT.............SAKDACQL
lcl|Glyma.06G270800_8-48     .........................PEE.CVATIIS..LT.............SPKDACQL
lcl|Glyma.06G271100_7-47     .........................PEE.CISMIVS..FT.............SPEDACRL
lcl|Glyma.13G064500_14-60    .....................ITDLNED.CIAHCAG..HL.............SLGDVCNM
lcl|Glyma.17G131400_23-62    ........................LPES.CVAQIMT..YM.............DPPQICKL
lcl|Glyma.05G049400_78-117   ........................LPES.CVAQIMT..YM.............DPPQICKL
lcl|Glyma.07G077600_23-55    ........................IPES.CVALVLM..YL.............DPPDICKL
lcl|Glyma.03G017000_23-61    ........................IPES.CVALVLM..YL.............DPPDICKL
lcl|Glyma.13G149600_18-56    ........................IPES.CVACVFL..HL.............TPPEICNL
lcl|Glyma.10G064800_18-56    ........................IPES.CVACVFL..HL.............TPPEICNL
lcl|Glyma.03G189500_21-61    ........................IPEN.CVARVFL..HL.............TPPEICNL
lcl|Glyma.10G238700_32-71    ........................IPES.CISSLFM..NL.............DPPDICKL
lcl|Glyma.09G145700_29-67    .........................PEN.CISSMMM..NF.............DPQEICSL
lcl|Glyma.17G022900_12-56    ......................SSLPED.VALKIAS..LL.............QVRDLCAL
lcl|Glyma.07G251400_13-56    .......................SLPED.VALKIAS..LL.............QVRDLCAL
lcl|Glyma.16G123900_129-161  .......................SLPMD.LLVKILC..HL.............HHDQLRAV
lcl|Glyma.02G045900_131-163  .......................SLPMD.LLVKILC..HL.............HHDQLRAV
lcl|Glyma.13G246200_46-82    ....................LLDALPQD.VLVQVLC..GV.............DHEDLKQL
lcl|Glyma.07G190100_53-90    ....................RLEALPLD.VLIRVLC..GV.............DHEDLEQL
lcl|Glyma.20G135900_323-367  .....................FTRLPME.LKLLILE..R.............LPGVDLAKV
lcl|Glyma.10G255600_327-371  .....................FMRLPME.LKLLILE..R.............LPGVDLAKV
lcl|Glyma.19G154800_16-53    ..............................FYDIFR..R.............LDGATLASA
lcl|Glyma.03G152300_16-53    ..............................FYDIFR..R.............LDGATLASA
lcl|Glyma.10G025700_15-53    .............................LLYDILR..R.............LDGPTLASA
lcl|Glyma.02G148100_28-63    ................................DILR..R.............LDGPTLASA
lcl|Glyma.13G314700_20-55    ................................HILN..R.............LDGPTLASA
lcl|Glyma.11G181200_32-69    ...............................AHILT..R.............LDGATLASA
lcl|Glyma.10G075800_22-60    ..........................DI.IQTHILT..R.............LPGPALASA
lcl|Glyma.03G198600_27-68    ..........................DI.IQTHILT..C.............LDGRALASV
lcl|Glyma.17G062000_214-251  ...............................LKILA..R.............LTPRDIASV
lcl|Glyma.13G097600_214-251  ...............................LKILA..Q.............LTPRDIASV
lcl|Glyma.09G056100_208-245  ...............................LKILA..R.............LTPRDIASV
lcl|Glyma.08G046500_233-272  ....................ILQLSDEV.LAHNILS..R.............LTPRDVASI
lcl|Glyma.05G239400_207-245  .....................LQLSDEV.LAHNILS..R.............LTPRDVASI
lcl|Glyma.11G090700_79-123   .......................QIPPE.LFNHILK..F.............LSSEDLVSC
lcl|Glyma.01G154100_79-123   .......................QIPPE.LFHHILK..F.............LSSEDLVSC
lcl|Glyma.19G119400_4-54     .......................LLPDE.LWRRILE..IGIES.....N..GFSYKDLCCV
lcl|Glyma.08G002000_12-56    ......................LCLPDD.VFGMVSR..F.............LLPRDVCNL
lcl|Glyma.05G194000_12-56    ......................LCLPDD.VFGLVSR..F.............LLPRDVCNL
lcl|Glyma.06G155700_5-46     .........................PDD.VFAIVSR..F.............MSPRDVCNL
lcl|Glyma.18G006600_20-60    .......................SLPDD.LVLSILC..KLSST........ATSPSDFISV
lcl|Glyma.11G250600_31-71    .......................SLPDD.LVLSILC..KLSST........ATSPSDFISV
lcl|Glyma.08G104400_65-106   .......................SLPDD.LVISIFC..KLSST........ATKPSDFVNV
lcl|Glyma.05G147400_69-110   .......................SLPDD.LVISIFC..KLSST........ATKPSDFVNI
lcl|Glyma.04G195700_16-55    .......................CLPDD.LLVLILS..KLSST........ASSPSDFINI
lcl|Glyma.11G154800_241-282  .......................ELSDD.ILTKILA..SLG.............PMDLTRV
lcl|Glyma.04G138300_237-278  .......................ELSDD.ILTKILA..SLG.............PMDLTRV
lcl|Glyma.20G042200_86-132   ....................SVLDLPEL.ALDCILE..R.............LPPSSLCRI
lcl|Glyma.07G235500_86-132   ....................SVLDLPEL.ALDCILE..R.............LPPSALCRM
lcl|Glyma.14G036300_85-130   ....................SVLDLPEL.VLECILE..K.............LPPASLCQM
lcl|Glyma.02G278300_85-130   ....................SVLDLPEL.VLECILE..K.............LPPPSLCQM
lcl|Glyma.19G129600_88-132   .....................LLDLPDL.PLECILE..H.............LSPAELCRV
lcl|Glyma.03G126600_90-134   .....................LLDLPDL.PLECILE..H.............LSPAELCRV
lcl|Glyma.03G126700_21-66    .....................LLDLPEW.TLDCILE..C.............LPPQDLCRV
lcl|Glyma.03G126900_1-44     ....................SLLNLPEP.ILDCILK..L.............LSPMELVSM
lcl|Glyma.03G126800_1-45     ....................SLLNLPYP.ILDCILK..L.............LSPMDLTRM
lcl|Glyma.19G129700_11-53    .....................LFNLQEP.VLDCILK..L.............LSPMGLIRM
lcl|Glyma.10G102300_11-56    .....................LLDLPEP.TLDLILK..C.............FSPNELCTV
lcl|Glyma.19G077200_211-253  ..........................HD.ALFFVLG..Y.............LGVRDLLSV
lcl|Glyma.05G073500_216-258  ..........................HD.ALFFVLG..Y.............LGVRDLLSV
lcl|Glyma.08G101200_189-221  ......................................Y.............LGLSDLLVV
lcl|Glyma.05G144800_201-233  ......................................Y.............LGLSDLLVV
```

```
lcl|Glyma.08G043200_22-57     ..........................HE.AMFLVLA..Y............LPVYEVVVM
lcl|Glyma.05G235800_20-55     ..........................HE.ALFLVLS..Y............LPVYEVVVM
lcl|Glyma.18G253400_43-88     .......................CLPDD.TVIQLLS..C............LSYRDRASL
lcl|Glyma.09G242200_43-88     .......................CLPDD.TVIQLLS..C............LSYQDRASL
lcl|Glyma.08G221300_34-71     .............................FTILLL.................LPIDAILSL
lcl|Glyma.07G020700_29-66     .............................FTILLL.................LPIDAILSL
lcl|Glyma.06G132400_2-45      .......................RLPLE.ICMKIFY..L............LDYQHLAVA
lcl|Glyma.04G232600_3-45      ........................LPLE.ICMKIFC..L............LDYQHLAVA
lcl|Glyma.14G009200_85-113    .......................................................ALAIL
lcl|Glyma.16G096700_44-84     .........................PAD.ILMQIVR..L............LGPKHAARL
lcl|Glyma.04G112600_47-88     ........................LPSD.ILMHILR..L............LGPKEAAKL
lcl|Glyma.14G179500_64-91     ..............................LENVLH..FLS...........SRRDRNAA
lcl|Glyma.02G211800_64-91     ..............................LENVLH..FLP...........SRRDRNAA
lcl|Glyma.06G095400_17-52     .....................PEQVLEN.VLESVLH..FLT...........SRRDRNAA
lcl|Glyma.04G093500_13-48     .....................PDQVLEN.VLESVLH..FLT...........SRRDRNAA
lcl|Glyma.19G206800_11-41     ..........................EE.VLEHVFS..FIW...........NERDRNAI
lcl|Glyma.19G100200_4-36      ........................FPDE.VIEHIFD..YVV...........SHSDRNAL
lcl|Glyma.16G050500_4-36      ........................FPDE.VIEHIFD..YVV...........SHSDRNAL
lcl|Glyma.02G065300_5-35      ..........................DE.VIEHIFD..YVT...........SQRDRNDL
lcl|Glyma.12G128600_9-41      ........................LPEE.ILLNVFS..AVS...........DTRTRNAL
lcl|Glyma.08G216300_8-40      ..........................EE.CWELVFR..FIG...........HGRHLESL
lcl|Glyma.07G026200_6-40      ........................LPEE.CWELVFR..FIG...........HGRHLESL
lcl|Glyma.19G068600_7-36      ...........................E.LLQEILQ..KLP...........SSSS.SSV
lcl|Glyma.16G071200_7-36      ...........................E.LLQEIFQ..KLP...........SSSS.SSV
lcl|Glyma.17G019800_12-42     ..........................DE.LIVEIFS..RLH...........SKSTRDAC
lcl|Glyma.07G254400_12-42     ..........................DD.LIVEIFS..RLH...........SMSTRDAC
lcl|Glyma.15G101100_11-42     .........................PDE.LLIEIFR..RLD...........SKSSRDAG
lcl|Glyma.13G211600_11-42     .........................PDE.LLIEIFR..RLD...........SKSNRDAS
lcl|Glyma.06G318100_54-84     ...........................E.LLLRILS..KLPD..........SSQQRNSN
lcl|Glyma.04G120000_114-144   ...........................E.LLLRILS..KLPN..........SQQQRNSN
lcl|Glyma.09G229800_64-94     ...........................E.LLLKIIA..LVSD..........SSTQRNSN
lcl|Glyma.11G227300_23-43     .......................................ID...........DPKDRDAV
lcl|Glyma.02G254300_17-37     .......................................IH...........DSKDRDAV
lcl|Glyma.15G004900_2-35      .......................NIPEH.IVWEILS..RLK...........KTSDRNSV
lcl|Glyma.08G059500_18-48     ..........................DE.VLERISG..MLK...........SRKDKSTV
lcl|Glyma.07G189800_17-46     ..........................DE.VLERILG..MLK...........SRKDKSTV
lcl|Glyma.14G136200_20-60     ...........................D.VLHLILG..RVE...........RQEDKEVF
lcl|Glyma.17G167800_40-72     .......................DIPDE.CLAGIFQ..FLS...........S.VDRKTC
lcl|Glyma.05G097100_59-91     .......................DIPDE.CLAGIFQ..FLS...........S.VDRKTC
lcl|Glyma.17G238700_64-96     ......................SDLPNE.CLASVFQ..FLS...........S.ADRSRC
lcl|Glyma.11G052600_36-77     .......................RLSDD.CLAAIFH..FLS...........T.ADRKRC
lcl|Glyma.01G189600_36-77     .......................RLSDD.CLAAIFH..FLN...........T.ADRKRC
lcl|Glyma.06G042800_9-51      ......................DDIPDN.CLACIFQ..LFP...........P.ADQKKL
lcl|Glyma.04G041800_24-67     ......................DEIPDE.CLGCIFQ..LFS...........P.GERKMF
lcl|Glyma.07G060100_13-46     .......................HLPDD.CLVIIFH..GLD...........SRIDRDSF
lcl|Glyma.17G211000_69-101    .........................LPDE.CLFEIFR..RLP...........SGEDRSAC
lcl|Glyma.14G116800_66-106    .....................IEALPDE.CLFEIFR..RLP...........AGEDRSAC
lcl|Glyma.06G068400_68-108    .....................IESLPDE.CLFEILR..RLP...........AGQDRSVC
lcl|Glyma.04G066900_66-109    .....................IESLPDE.CLFEILR..RLP...........AGQDRSVC
lcl|Glyma.13G166200_66-97     .......................VLPDE.CLFEIFR..RLP...........SGKERSSC
lcl|Glyma.04G147000_69-100    .......................VLPDE.CLFEVFR..WLS...........SGKERSSC
lcl|Glyma.17G026300_26-71     .......................FLPKE.LQVEIFA..KVATR.........SVFDHCMI
lcl|Glyma.07G247800_23-68     .......................SLPKE.LQVEIFA..KVATR.........SIFDHCMI
lcl|Glyma.02G238900_22-70     ......................LSLPSD.LLEEIIA..KAASK.........SSIDLVNI
lcl|Glyma.20G105000_9-46      ......................DQLPSA.LVATIMS..KLDI............ASICSL
lcl|Glyma.17G035100_46-127    EVPMNSSTISDTLYANRSEDATISLVEEGLKETLLPSDKIPSEKVIDEVGIVSIKCLYAY
lcl|Glyma.13G246100_26-61     .......................ELPLE.ILLHVFS..FLPI............KVAAQN
lcl|Glyma.07G162700_19-49     ........................LTIVQVRRKLIE....................DWLPV
lcl|Glyma.03G214300_17-57     ...........................D.IVLNIFY..KLEDD..........PRHWARL
lcl|Glyma.13G353900_191-234   .......................CVPKC.ISSHLRTCCVYKYG.........GYETVFEF
lcl|Glyma.07G111600_14-69     .................NKVDKKSQNEANKVENIID...LPMN..........IVRKWNI
lcl|Glyma.01G094300_169-235   .............RKERGENNFKSFSANNIYEWNID.AQMEYN.........IMNTLQHM
```

```
                                              α3
Human_Skp2                                  ℓℓℓℓℓℓℓ
                                  30
                                   .
Human_Skp2                        SGVCKRWYRLASD...............
F-box                             RKVSRKWRSLIDSHDFWFKL........
lcl|Glyma.U017700_36-79           SLVCKLWNAIVESRSLREF.........
lcl|Glyma.12G196800_1201-1238     SMTCKRWRAAV..RFYKEVSI.......
lcl|Glyma.11G054300_7-48          SGVCKKWKQGVKESLAR...........
lcl|Glyma.01G187800_7-48          SGVCKKWKQGVKESLAR...........
lcl|Glyma.03G106100_7-49          SGVCKKWKQGVKESLARR..........
lcl|Glyma.02G038300_46-79         SLVCRAWCEAL.................
lcl|Glyma.07G032000_3-39          RSVCKNWHTCSK................
lcl|Glyma.20G163800_142-184       GLVCWSWNIAANDNHLWEKQY.......
lcl|Glyma.10G229500_118-156       GLVCRSWNIAANDNHLWE..........
lcl|Glyma.11G154400_33-71         ALVCHRWNYLACHPRLWLRVD.......
lcl|Glyma.20G157500_17-57         SRVCSAWRMACCDPLLWKTLDL......
lcl|Glyma.10G236900_16-56         SRVCSAWRLACCDPLLWKTLDL......
lcl|Glyma.15G092200_20-66         QRVCSVWRGISKEPLLWRTIDM......
lcl|Glyma.13G220200_14-60         QGVCSVWRAISKEPLLWRTIDM......
lcl|Glyma.02G057300_20-59         SLVCKMWNKACRDPSLWRKIDL......
lcl|Glyma.01G100400_3-33          SLVCKMWNKACHDPSLWRKIDL......
lcl|Glyma.15G192600_18-57         SLVCKMWNTACRDPSLWGKIDL......
lcl|Glyma.06G101800_9-54          PRVCKSWANAVTGPYCWQEI........
lcl|Glyma.04G100100_9-54          PRVCKSWANAVTGPYCWQEI........
lcl|Glyma.19G157300_105-147       SAACSLFSKCAKDPMCYANLDL......
lcl|Glyma.03G155100_106-148       SATCLLFSKCAKDPMCYANLDL......
lcl|Glyma.08G024000_93-132        SMTNSLMRKAANDDNAWK..........
lcl|Glyma.05G217900_93-132        SMTNSLMRKAANDDNAWK..........
lcl|Glyma.16G025000_50-90         AATSRWFRRAIMDDGIWKFVCLR.....
lcl|Glyma.18G265500_5-43          MAVDKQWKDIASDDFLWKCLC.......
lcl|Glyma.08G243300_5-43          MGVCTQWKDIASDDFFWKCLC.......
lcl|Glyma.17G237800_42-82         GCVSKQWQKAARDERLWELIC.......
lcl|Glyma.14G086600_29-69         GCVSKQWQKMARDERLWELICT......
lcl|Glyma.06G043400_36-75         GCVNKQWHKTAQDERLWELICT......
lcl|Glyma.04G042900_45-84         GCVNKQWHKTAQDERLWELICT......
lcl|Glyma.17G225800_189-229       ARVCKQWRGASAHEDFWKSLNF......
lcl|Glyma.14G098900_182-222       ARICKQWRGASAHEDFWKSLNF......
lcl|Glyma.06G055800_103-143       ARVCKQWRTASAHEDFWKSLNF......
lcl|Glyma.04G055700_105-145       ARVCKQWWTASAHEVFWKSLNF......
lcl|Glyma.20G190700_8-41          SAVCKLWRSLVTNE..............
lcl|Glyma.10G199800_8-41          SAVCKSWRSLISNE..............
lcl|Glyma.20G166900_52-105        AAVCKSWREMCKEIVSSPEFC.......
lcl|Glyma.10G224900_52-105        AAVCKSWREMCKEIVSSPEFC.......
lcl|Glyma.12G115200_19-72         VAMCKSWREMCKEIVSSPEFC.......
lcl|Glyma.07G147700_29-81         AAMCKSWREMCKEIVSSPEFC.......
lcl|Glyma.16G167200_50-103        AAVCRSWRGMCKEIVTSPEFC.......
lcl|Glyma.02G081800_50-103        AAVCKSWREMCKEIVTSPEFC.......
lcl|Glyma.15G049500_52-105        AAVCQSWRNMCKDIVKSPEFC.......
lcl|Glyma.08G183100_52-105        AAVCQSWRNMCKDIVKSPEFC.......
lcl|Glyma.17G251500_81-133        ASVCKLWREITKDVVKTPEQ........
lcl|Glyma.14G073500_59-110        ASVCRLWREITKDVIKTPE.........
lcl|Glyma.16G138100_55-106        ASVCKSWRSITREIVKTPEQ........
lcl|Glyma.02G055300_55-106        ASVCKSWRSITREIVKTPEQ........
lcl|Glyma.11G069400_54-106        GSVCKSWRAVTKEIVKTPEQ........
lcl|Glyma.01G173700_55-106        GSVCKSWRAVTKEIVKTPEQ........
lcl|Glyma.15G098200_32-82         AGVCRSWRQITIDIVKTP..........
lcl|Glyma.13G214900_36-87         AGVCRSWRHITKDIVKTPE.........
lcl|Glyma.17G022700_13-56         GGVCRTWRRLI.................
lcl|Glyma.13G269600_48-95         ACVCKRWRDITREVVR............
lcl|Glyma.12G230000_46-93         ACVCKRWRDITREVVR............
lcl|Glyma.19G161300_136-172       AMVSRGWREMTRS..L............
lcl|Glyma.03G158900_123-159       AKVSRGWREMTRS..L............
lcl|Glyma.10G032000_129-167       AKVCKGWRDTTKR..LWRAV........
lcl|Glyma.02G142300_127-164       AKVCKGWRDTAKR..MWR..........
lcl|Glyma.06G115200_63-108        SCVCRKWKYTIRNPVFWRNACLK.....
lcl|Glyma.04G247500_63-108        SCVCRKWKYTIRNPVFWRNACLK.....
lcl|Glyma.20G075000_5-43          RCVAKWLRALISDPTFV...........
lcl|Glyma.19G048900_32-63         RCVFKAWNSFS.................
lcl|Glyma.13G114500_13-47         RCVCKFWKTLVFDP..............
lcl|Glyma.15G101600_25-67         RCVCKSWMSLIYDPYFMKKH........
lcl|Glyma.13G211000_47-89         RCVCKSWMSLISDPYFMKKH........
lcl|Glyma.15G101700_47-83         RCVCKSWKSLISHPQF............
lcl|Glyma.02G126700_3-26          ............................
lcl|Glyma.05G071700_5-43          RCVSKTWNSLILHPTF............
lcl|Glyma.05G071400_5-43          RCISKTWNSLILHPTF............
lcl|Glyma.08G353000_5-43          RCVSKTWNSLILHPTF............
lcl|Glyma.08G134900_8-42          KCVSKTWNSLIFHP..............
lcl|Glyma.05G166200_5-38          RCVSKSWNSLIFH...............
lcl|Glyma.02G041600_10-43         RCVSKSWNSLIFN...............
lcl|Glyma.19G256900_7-41          RCVSKTWNSLILNP..............
lcl|Glyma.06G181300_17-52         RCVSKSWNSLILDPTFV...........
lcl|Glyma.05G071600_7-39          RCVSKTWKSLISH...............
lcl|Glyma.05G071300_7-39          RCVSKTWKSLISH...............
lcl|Glyma.08G352900_7-37          RCVSKTWKSLI.................
lcl|Glyma.17G116300_16-49         KCVSKTWNSLIFHPML............
lcl|Glyma.17G044800_8-45          RCVCKSWKSLIFDPSFK...........
lcl|Glyma.13G114400_4-34          RCVCKSWKSLMLDLSFV...........
lcl|Glyma.08G350400_8-50          RCVCKTWKSIIFDPSFVKKH........
lcl|Glyma.19G048800_4-37          RCVSRTWNSLI.................
lcl|Glyma.19G048700_4-37          RCVSRTWNSLI.................
lcl|Glyma.19G048600_4-37          RCVSRTWNSLI.................
lcl|Glyma.19G048300_4-37          RCVSRTWNSLI.................
lcl|Glyma.19G048000_4-37          RCVSRTWNSLI.................
lcl|Glyma.19G048200_4-37          RCVSRTWNSLI.................
lcl|Glyma.19G048100_4-37          RCVSRTWNSLI.................
lcl|Glyma.19G049000_4-37          RCVSSTWNSLI.................
lcl|Glyma.19G048500_4-37          RCISRTWNSLI.................
lcl|Glyma.07G156800_28-66         TCASKWFQSLISDSSFVK..........
lcl|Glyma.18G168800_12-48         KCVCKGWNSLMSDPYF............
lcl|Glyma.18G161800_14-47         KCVCKGWNSLMSDPY.............
lcl|Glyma.18G168300_12-48         KCVCKGWNSLMSDPYF............
lcl|Glyma.18G160800_12-48         KCVCKGWNSLMSDPYF............
lcl|Glyma.18G162900_12-48         KCVCKGWNSLMSDPYF............
lcl|Glyma.18G163200_12-48         KCVCKGWNSLMSDPYF............
lcl|Glyma.18G168500_1-31          KYVCKGWNSLMSDPYF............
lcl|Glyma.18G161900_12-48         KCVCKGWNSLMSDPYF............
```

```
lcl|Glyma.18G162500_12-48      KCVYKGWNSLMSDPYF.............
lcl|Glyma.18G160700_1-30       KCVYKGWNSLMLDPY..............
lcl|Glyma.18G162400_12-48      KCVCKGWNSLMSDPYF.............
lcl|Glyma.18G161500_14-48      KCVYKGWNSLMSDPYF.............
lcl|Glyma.18G162700_12-49      KCVCKGWNSLISDPYFI............
lcl|Glyma.18G162600_11-49      KCMCKEWNSLISEPYF.............
lcl|Glyma.18G162300_14-50      KCVCKEWNSLISEPYF.............
lcl|Glyma.18G168000_8-37       KCVCKEWNSLISEPYF.............
lcl|Glyma.18G163100_10-47      KCVCKEWNSLMSEPY..............
lcl|Glyma.14G160200_8-46       MCVCKEWNSLMSEP...............
lcl|Glyma.18G162800_10-46      KCVCKGWNSLMSEP...............
lcl|Glyma.18G276400_6-41       KCVRKEWNNLISDPEF.............
lcl|Glyma.20G073200_7-44       KCVSKSWCALISDPEFA............
lcl|Glyma.07G187200_13-47      KCVCKSWFSLISNPEF.............
lcl|Glyma.16G203000_9-45       KCMCKSWFFLISHPEF.............
lcl|Glyma.16G202600_12-48      KYMCKSWFSLISHPEF.............
lcl|Glyma.16G202900_12-48      KCMCKLWFSLISDPEF.............
lcl|Glyma.16G202800_1-34       KCMCKLWFSLISHPEF.............
lcl|Glyma.01G232700_12-48      KCMCKSWFSLISDPEF.............
lcl|Glyma.10G117700_28-64      KCVCKSWLSLISDPQF.............
lcl|Glyma.08G252000_10-33      KCV..........................
lcl|Glyma.08G252300_8-45       RCVCKSWLSLISDPQFR............
lcl|Glyma.18G274900_8-44       KCVCKSWFSLISDPQF.............
lcl|Glyma.07G148300_5-41       KCVCKSWFSLISEPQ..............
lcl|Glyma.18G274700_8-46       KCVCKSWLSLISDPQFGF...........
lcl|Glyma.03G116000_13-48      KCVCKSWLSVISDPHF.............
lcl|Glyma.18G274500_6-42       KCVCKSWNFIISNPQF.............
lcl|Glyma.08G251800_6-42       KCVCKSWLSIISDPQF.............
lcl|Glyma.17G162200_4-40       KCVRKSWLFLISDPQ..............
lcl|Glyma.10G132100_7-44       KCVRKSWLFLISDPQFT............
lcl|Glyma.06G197100_7-43       KYVCKSWLSLISDPHF.............
lcl|Glyma.06G197000_14-50      KCVCKSWLSLISDPQF.............
lcl|Glyma.10G220200_8-46       RCVCKSWKTLISHP...............
lcl|Glyma.02G188000_34-67      KCVCKSWNSLISDPL..............
lcl|Glyma.10G220500_40-78      KCVCKSWKTLISDPQF.............
lcl|Glyma.06G016300_7-47       KCVCKSWLSLISDPQFVK...........
lcl|Glyma.08G252200_30-68      KRVCKAWLSLISDPQFFI...........
lcl|Glyma.06G197200_5-41       KRVCKSWLSLISDPQF.............
lcl|Glyma.18G274800_22-60      KCVCKSFLSLISDPQFV............
lcl|Glyma.13G109000_20-53      KRVCNSWLSLISD................
lcl|Glyma.17G017600_31-68      KTVCKSWLSHISDPHF.............
lcl|Glyma.16G164000_14-50      KCVCKLWLSLISDPHF.............
lcl|Glyma.02G079600_14-50      KCVCRLWLSLISDPSF.............
lcl|Glyma.06G126800_15-54      KCVCKSWLCLLSDPHF.............
lcl|Glyma.17G027000_18-43      KCISN........................
lcl|Glyma.07G247000_18-54      KCVSKSWLSLITDPHF.............
lcl|Glyma.20G014700_28-52      KCVC.........................
lcl|Glyma.17G021300_21-61      KSVCKSWLSFISDSHLWLSH.........
lcl|Glyma.08G098000_3-39       KSVCKSWLFLISDPRF.............
lcl|Glyma.08G251700_6-43       KCVCKSWLSFISDPHFVK...........
lcl|Glyma.18G256100_8-31       KRV..........................
lcl|Glyma.15G055200_11-42      KCVSKDWFNLF..................
lcl|Glyma.09G032600_10-43      KTVCKLWYRLSLDK...............
lcl|Glyma.02G128400_30-69      RCVCKLWDCFIRDPSFL............
lcl|Glyma.17G008200_11-50      RSTCKWWRSIIDSRHF.............
lcl|Glyma.07G265800_3-43       RSTCKWWRSIIDSRHFVL...........
lcl|Glyma.15G115100_4-44       RSTSKSWKSLIDSQHLNWL..........
lcl|Glyma.09G010600_4-42       RSTSKSWKSLIDSQHFN............
lcl|Glyma.06G045700_5-48       RPTCKAFTCLLFSPSFVSK..........
lcl|Glyma.04G045200_5-49       RPTCKAFTSLLFSPSFVSKH.........
lcl|Glyma.14G088700_35-79      RSTCKGFWSLIFSPPFISKHC........
lcl|Glyma.19G081700_4-43       KSVCKSWFDLITDSHFVS...........
lcl|Glyma.16G063500_47-81      KRVCKSWFDLITD................
lcl|Glyma.19G081500_1-45       KCVCNSWFDLITDPHFVSN..........
lcl|Glyma.16G063600_1-45       KCVCKSWFDLITDPHFVSN..........
lcl|Glyma.15G220600_3-41       TSVCKAWNKIIRSHDF.............
lcl|Glyma.16G161700_9-36       KCTSKTLAKLLE.................
lcl|Glyma.15G092300_5-37       KCTCKTVSNIVE.................
lcl|Glyma.03G020200_4-34       TSTSKSFSKL...................
lcl|Glyma.04G198800_33-68      STLNKQSNEIINSP...............
lcl|Glyma.09G075900_20-65      KSVSKRWLTLISNPNFHSLFL........
lcl|Glyma.07G022100_3-47       KCVCKRWLGLISSPSFPTEF.........
lcl|Glyma.02G083400_9-46       RSVSKFFLQLLSTPSF.............
lcl|Glyma.19G252100_40-81      GCVSKRWHEIVNSERFVW...........
lcl|Glyma.03G254500_40-81      GCVSKRWHEIVNSERFVW...........
lcl|Glyma.08G088700_46-81      RCVCKRWYALL..................
lcl|Glyma.08G104800_104-143    RSVCQRWNSLLTSQSF.............
lcl|Glyma.05G147900_105-145    RSVCQRWNSLLSSQSFS............
lcl|Glyma.18G005400_113-153    RSVCRQWNSMLTSQSFS............
lcl|Glyma.17G220600_21-64      TSVCKRWKSAAASVSFKL...........
lcl|Glyma.14G105800_21-64      TSVCKRWKSAAASVSFKL...........
lcl|Glyma.06G058900_28-68      NSVCKRWKSVAASAS..............
lcl|Glyma.04G058200_28-68      NSVCKRWKSVAASAS..............
lcl|Glyma.18G275900_100-142    RLVCKRWNSLLQDSSFLKF..........
lcl|Glyma.08G253600_95-137     RLVCKRWNSLLQDSSFLKF..........
lcl|Glyma.13G209700_23-63      RCVCKRWKSLVLDPQFVKKH.........
lcl|Glyma.06G168700_28-71      ACVSKSWNSLCSRILSRPK..........
lcl|Glyma.04G196900_27-69      ACVSKSWNSLCNRILTRP...........
lcl|Glyma.19G162400_18-54      KATCKSWKLLLSS................
lcl|Glyma.03G160600_17-56      KATCKSWKLLLSSKSF.............
lcl|Glyma.19G158300_6-45       ELVSRSWQAAICSPELF............
lcl|Glyma.03G156000_5-45       ELVSRSWQAAIRSPELF............
lcl|Glyma.10G028000_6-45       ELVSRAWRAVVRGPELF............
lcl|Glyma.06G146500_19-60      SLVSKPIRTLLSSPILFT...........
lcl|Glyma.04G219400_21-60      SLVSKPIRSLLYSPLLFTT..........
lcl|Glyma.07G071600_25-62      KCVSKRWRDLICSE...............
lcl|Glyma.03G010000_2-33       KCVSKRWRDLICSEE..............
lcl|Glyma.07G071500_32-72      KCVSKRWRNLICSEEWF............
lcl|Glyma.07G071400_25-62      KCVSKRWRDLICSE...............
lcl|Glyma.08G112900_78-117     RLVCKRWYHLLSGNFF.............
lcl|Glyma.05G155000_78-117     RLVCKRWYRLLSGNFF.............
lcl|Glyma.19G005000_68-104     RLVCKRWYRLLVG................
lcl|Glyma.09G019400_18-59      HLVCKRWHRLLSEDFFY............
```

```
lcl|Glyma.15G093300_53-91      RAVCKRWHMLLGNKE..............
lcl|Glyma.13G218800_53-91      RAVCKRWHMLLGNKE..............
lcl|Glyma.18G007900_34-73      GVVCKGWRSFIQSKEF.............
lcl|Glyma.11G249300_49-87      GGVCKIWRSFIQSKE..............
lcl|Glyma.08G103400_52-91      GGVCKRWRGFIRSKEF.............
lcl|Glyma.15G013300_4-46       ASVCKGWSAEIHSPDFHRR..........
lcl|Glyma.13G360600_4-47       ASVCKGWNTEIHSPDFHRRR.........
lcl|Glyma.08G207700_4-46       ASVCKLWKSEIHAPEFHRQ..........
lcl|Glyma.07G034400_4-36       ASVCKLWKS....................
lcl|Glyma.20G045100_49-89      SLVCRRWSRLLSSPDF.............
lcl|Glyma.05G155600_55-96      LRVCSQWHCLLQSDAFYS...........
lcl|Glyma.06G016400_142-179    RLVCKKWRSLTTTPRFL............
lcl|Glyma.04G016300_139-176    RLVCKKWRSLTTTPRFL............
lcl|Glyma.19G246800_38-73      IIVSKIWLRVICSPSFR............
lcl|Glyma.03G249300_21-56      IIVSKIWLRVICSPSFR............
lcl|Glyma.06G076900_64-102     RSVSSTWNRAITHPS..............
lcl|Glyma.02G036400_11-44      SHVSNSWKRAVSSSLA.............
lcl|Glyma.01G028800_12-45      SHVSNSWKRAVSSSLA.............
lcl|Glyma.08G284600_18-52      SHVSNSWKRAVSTSLRH............
lcl|Glyma.18G141000_17-47      SHVSHEWKRAIST................
lcl|Glyma.08G259900_8-42       KLVSKEWHRLISSR...............
lcl|Glyma.01G083900_11-43      KRVCKEWHHVISSRC..............
lcl|Glyma.14G167900_43-84      RLVSRRWKTTITSLELYK...........
lcl|Glyma.13G086000_43-84      RLVSRKWKATITSSELYK...........
lcl|Glyma.06G106800_43-84      RLVSKKWKSTIMSSELYK...........
lcl|Glyma.04G107000_43-84      RLVSKRWKSTIMSSELYK...........
lcl|Glyma.15G214000_43-89      RLVSRRWKATIKFNLIYDRLCIK......
lcl|Glyma.08G074400_43-84      KLVCRAWKETFVSSELFC...........
lcl|Glyma.05G119400_43-84      KLVCRAWKETLVSSELFC...........
lcl|Glyma.08G298900_194-236    ASLNRSFRNTIRSGELYRWR.........
lcl|Glyma.02G105600_194-236    ASLNRSFRNIIRSGELYQWR.........
lcl|Glyma.13G326800_49-82      SCINKRFNKLINS................
lcl|Glyma.20G215600_15-54      CILSKRWRNLWKFLP..............
lcl|Glyma.10G175100_15-54      CILSKRWRNLWKFLP..............
lcl|Glyma.17G078800_21-60      SVLSKRFIYLWTSLP..............
lcl|Glyma.07G004400_30-68      CVLSKRWRHVWTSLP..............
lcl|Glyma.16G177700_131-169    CVLSKRWKDLGKGL...............
lcl|Glyma.09G139100_17-56      CVLSKRWKDLCKRLT..............
lcl|Glyma.09G140100_13-51      CVLSKPWKDLCKRL...............
lcl|Glyma.09G130200_31-69      CVLSKRWKDLCKRL...............
lcl|Glyma.09G140200_31-69      CVLSKRWKDLWKSL...............
lcl|Glyma.13G224200_116-152    CVLSKRWNDLSK.................
lcl|Glyma.09G139800_14-52      CVLSKRWNNLWKRL...............
lcl|Glyma.09G139700_14-52      CVLSKRWNNLWKRL...............
lcl|Glyma.09G139900_29-68      CVLSKRWNNLWKRLS..............
lcl|Glyma.10G136700_25-60      CVLSKRWNNLWKSL...............
lcl|Glyma.09G141700_24-62      CVLSKRWKDLWKRL...............
lcl|Glyma.09G141600_32-70      CVLSKRWKDLWKRL...............
lcl|Glyma.09G141500_32-70      CVLSKRWKDLWKRL...............
lcl|Glyma.09G141400_32-70      CVLSKRWKDLWKRL...............
lcl|Glyma.09G141300_32-70      CVLSKRWKDLWKRL...............
lcl|Glyma.09G141200_32-70      CVLSKRWKDLWKRL...............
lcl|Glyma.09G141100_32-70      CVLSKRWKDLWKRL...............
lcl|Glyma.10G134800_20-58      CILSKRWKNLWKRL...............
lcl|Glyma.09G142000_40-78      CVLSKRWKELWKRL...............
lcl|Glyma.10G135600_12-49      CILSKRWKDLWKH................
lcl|Glyma.10G135200_27-64      CILSKRWKDLWKH................
lcl|Glyma.10G135500_27-64      CILSKRWKDLWKH................
lcl|Glyma.09G130100_116-155    SVLFNRWKNFCKALS..............
lcl|Glyma.06G098100_17-56      CVLSTRWKDLWKRLP..............
lcl|Glyma.15G227900_18-56      CALSKSWKDFWKRL...............
lcl|Glyma.18G164400_4-43       SVLSKRWRPLWRSVP..............
lcl|Glyma.08G351300_7-40       SLLSKRWS.....................
lcl|Glyma.18G164500_7-43       GILSKRWGPLWR.................
lcl|Glyma.13G353900_4-42       SVLSKRWKPLWRSV...............
lcl|Glyma.18G164300_4-42       SVLSKRWKLLWRSV...............
lcl|Glyma.08G351400_4-43       SILSKRWKALWRSVP..............
lcl|Glyma.18G164600_21-53      GILSKRWR.....................
lcl|Glyma.12G103400_25-61      SILSKRWKFLWS.................
lcl|Glyma.13G283500_19-52      CVLSNRWKTM...................
lcl|Glyma.15G241900_46-76      SVLSKRW......................
lcl|Glyma.15G241800_37-67      SVLSKSW......................
lcl|Glyma.15G241500_46-77      SILSKRW......................
lcl|Glyma.13G262900_18-55      SILSKRWRNLWKF................
lcl|Glyma.17G193700_22-57      SVLSKRWINLW..................
lcl|Glyma.15G242400_9-60       SVLSKGWIHVWKSI...............
lcl|Glyma.15G242200_29-66      SVLSKRWVDAWKSI...............
lcl|Glyma.15G242000_23-61      SVLSTRWINVWTSIT..............
lcl|Glyma.13G262800_23-60      SVLSTRWIDVWTSI...............
lcl|Glyma.20G142600_29-63      SVLSRRWHSQW..................
lcl|Glyma.15G022500_46-82      SVLSKRWRELW..................
lcl|Glyma.08G192100_57-96      CVLSKRWRYLWASVP..............
lcl|Glyma.13G278200_43-78      SVLSTRWRSLW..................
lcl|Glyma.07G072400_15-54      SLLSTRWRFLWTMLP..............
lcl|Glyma.08G348900_6-44       SLVSKRWQPLWLSI...............
lcl|Glyma.20G098700_4-40       SLISTRWRDLWN.................
lcl|Glyma.10G290500_5-34       SLISTRWRDL...................
lcl|Glyma.18G286800_5-41       SLLSTRWRDLWN.................
lcl|Glyma.02G094600_5-41       SLLSTRWRDLWN.................
lcl|Glyma.02G127700_9-44       SILSSKWRYKW..................
lcl|Glyma.01G072000_9-46       SILSSKWRYKWAS................
lcl|Glyma.02G127100_16-54      SLLSSKWRYKWTSV...............
lcl|Glyma.02G126900_16-55      SLLSSKWRYKWTSIP..............
lcl|Glyma.17G247500_18-51      SVLSSKWRY....................
lcl|Glyma.17G128800_29-62      SILSRKWRYL...................
lcl|Glyma.12G068200_27-62      SVLATKWRYHWT.................
lcl|Glyma.08G195600_6-40       SVLSKSWAETW..................
lcl|Glyma.08G195400_12-48      SVLSKKWAEIWST................
lcl|Glyma.13G261600_7-44       SVLSKTWKGLWKGM...............
lcl|Glyma.09G102300_7-43       SILSKHWLHIWK.................
lcl|Glyma.08G312700_7-45       CILSKYWLHIWKNS...............
lcl|Glyma.17G194900_31-63      SVLSKKWQ.....................
lcl|Glyma.10G200400_12-47      SILSKRWSKIWLS................
```

```
lcl|Glyma.02G289100_7-44       CVLSKDWLDICKS................
lcl|Glyma.02G289200_6-45       SIISSKWLKACKLTK..............
lcl|Glyma.06G120600_42-76      SGVCRGWRDAI..................
lcl|Glyma.04G242700_1-26       SGVCRGWRDAI..................
lcl|Glyma.13G036600_43-77      SEVCRGWREAI..................
lcl|Glyma.12G088900_2-39       SASCRKWRLACCKH...............
lcl|Glyma.15G081900_5-39       RGVCKDWR..VVS................
lcl|Glyma.13G230700_4-39       RGVCEDWR..VAS................
lcl|Glyma.04G019900_5-47       RSVCRSWRSSVPKIPLHL...........
lcl|Glyma.09G116200_72-107     SKVCTSWN.RVL.................
lcl|Glyma.10G154000_52-81      SCVCRLWNSVAS.................
lcl|Glyma.08G356100_5-44       STVCKLWHSIISSSSF.............
lcl|Glyma.13G154400_35-73      SVVCKRWHSVATSV...............
lcl|Glyma.06G213000_16-54      KLLNKSFSRIISDNTF.............
lcl|Glyma.04G150400_60-99      KLVNKSFSRIISDHAFV............
lcl|Glyma.18G227100_18-56      TCVSRSWRDIVIANGLCKQLCLR......
lcl|Glyma.16G197900_17-58      SSVSSSWHRFVIEHGLCKQLCLK......
lcl|Glyma.14G044300_96-140     SCVSPILQRVASEHHAWKQF.........
lcl|Glyma.02G273700_95-139     SCVSTIFQRVASEHGAWKQF.........
lcl|Glyma.15G046900_4-35       NCVSKR..........WKDS.........
lcl|Glyma.08G185900_4-35       NCVSKR..........WKDS.........
lcl|Glyma.08G127900_2-36       NCVSKR..........WKDST........
lcl|Glyma.05G169600_2-36       NCVSKR..........WKDST........
lcl|Glyma.13G164200_19-62      ACVIGVMYILYNEDPLWMSLCR.......
lcl|Glyma.06G101600_19-63      SCVSSVMYILCNEDPLWMSLCLK......
lcl|Glyma.12G051100_14-48      ACVSKRFSSSASDDTLWINLCF.......
lcl|Glyma.15G092600_22-56      CVVSRRFHSLV..................
lcl|Glyma.13G219800_25-59      CVVSRRFHSLV..................
lcl|Glyma.17G251700_4-43       S.VSIAFRSAAESDTV.WD..........
lcl|Glyma.14G073300_3-44       SLVSKAFRSAAESDTV.WD..........
lcl|Glyma.20G220300_18-63      SLVSKAFRSAAQSDTL.WDRFI.......
lcl|Glyma.20G220200_12-43      SLVSKAFHSAAEANTV.W...........
lcl|Glyma.10G169300_5-46       SLVSKAFYSAADYDTV.WDR.........
lcl|Glyma.10G169600_3-46       SVVSKIFRSAAESDAV.WKRF........
lcl|Glyma.20G220100_9-52       SLLSSTFRSAAQSDAV.WNKF........
lcl|Glyma.10G169700_11-37      SLVSSIFRS....................
lcl|Glyma.03G233900_4-44       SLVSSIVQSMADSDAV.WEKF........
lcl|Glyma.03G253900_24-65      SLVSSSFRSASVTDFV.WER.........
lcl|Glyma.19G231000_9-54       SLVSPSLCSCANSDTV.WRSF........
lcl|Glyma.03G233800_10-55      SLVSPTLRSCANSDTV.WRSF........
lcl|Glyma.10G150400_4-45       SMVSSTLRSSADSDLL.WRTFF.......
lcl|Glyma.06G271000_8-48       SPVSPSLKAIADSDAV.WANF........
lcl|Glyma.06G270800_8-48       SLVSPSFKEIADSDAV.WANF........
lcl|Glyma.06G271100_7-47       SLVSPFFKEIADSDAV.WENF........
lcl|Glyma.13G064500_14-60      AMTSSALKRLAYSDFI.WQRFFR......
lcl|Glyma.17G131400_23-62      ATLNRAFRGASSADFV.WE..........
lcl|Glyma.05G049400_78-117     ATLNRAFRGASSADFV.WE..........
lcl|Glyma.07G077600_23-55      ARLNRAFRDAS..................
lcl|Glyma.03G017000_23-61      ARLNRAFRDASSADFI.W...........
lcl|Glyma.13G149600_18-56      ARLNRAFRGAASSDSV.W...........
lcl|Glyma.10G064800_18-56      ARLNRAFRGAASSDSV.W...........
lcl|Glyma.03G189500_21-61      ARLNRAFRGAASADSV.WQT.........
lcl|Glyma.10G238700_32-71      ARVNRAFHRASSADFV.WE..........
lcl|Glyma.09G145700_29-67      ARVNKTFHRASSANFV.WE..........
lcl|Glyma.17G022900_12-56      GCCSMFWKELCFSDCI.WESLV.......
lcl|Glyma.07G251400_13-56      GCCSRFWRELCFSDCI.WESLV.......
lcl|Glyma.16G123900_129-161    FHVSQKIRKA...................
lcl|Glyma.02G045900_131-163    FHVSQRIRKA...................
lcl|Glyma.13G246200_46-82      FHVSKTIREAT..................
lcl|Glyma.07G190100_53-90      VRVSKTVREAAE.................
lcl|Glyma.20G135900_323-367    ACTCSELRYLSTSNEL.WKKK........
lcl|Glyma.10G255600_327-371    ACTCSELRYLSTSNEL.WKKK........
lcl|Glyma.19G154800_16-53      ACTCAALCSISKEENL.WENVC.......
lcl|Glyma.03G152300_16-53      ACTCAALCSISKEESL.WENVC.......
lcl|Glyma.10G025700_15-53      ACTCASFSSISKEESL.WENVC.......
lcl|Glyma.02G148100_28-63      ACTCASFSSISKEESL.WENVC.......
lcl|Glyma.13G314700_20-55      ASSTSHLRRLCTEHHL.WRNIS.......
lcl|Glyma.11G181200_32-69      ASVSSLMHRLCTQDDL.WREIST......
lcl|Glyma.10G075800_22-60      TATCSQLHSLSSHDPL.WLN.........
lcl|Glyma.03G198600_27-68      ASTCSQLHALSDHEPL.WENICH......
lcl|Glyma.17G062000_214-251    SSVCRRLYELTKNEDL.WRMVCQ......
lcl|Glyma.13G097600_214-251    SSVCRRLYELTKNEDL.WRMVCQ......
lcl|Glyma.09G056100_208-245    GSVCRHLYELTKNEDL.WRMVCQ......
lcl|Glyma.08G046500_233-272    GSVCRRVRQLTKNE...............
lcl|Glyma.05G239400_207-245    GSVCRRIRQLTKNE...............
lcl|Glyma.11G090700_79-123     SLVCRFLNYAASDEAL.WRRLYC......
lcl|Glyma.01G154100_79-123     SLVCRFLNYAASDEAL.WRRLYC......
lcl|Glyma.19G119400_4-54       SISCRRLHRLSSEEPL.WNRLLF......
lcl|Glyma.08G002000_12-56      SLCCKSLYALSSSEKV.WFPQC.......
lcl|Glyma.05G194000_12-56      SLCCKSLYALGSSEKV.WFTQC.......
lcl|Glyma.06G155700_5-46       CLCCKSLNALVASEKL.WLTQC.......
lcl|Glyma.18G006600_20-60      LITCKRLNSLGLH................
lcl|Glyma.11G250600_31-71      LITCKRLNSLGLH................
lcl|Glyma.08G104400_65-106     LITSKRLNRLALHS...............
lcl|Glyma.05G147400_69-110     LITCKRLNRLALHS...............
lcl|Glyma.04G195700_16-55      ILTCKRLNRLGL.................
lcl|Glyma.11G154800_241-282    SATCHHLRSLAASVMP.YTK.........
lcl|Glyma.04G138300_237-278    SATCHHLRSLAASVMP.YTK.........
lcl|Glyma.20G042200_86-132     AAVCRTLRERCVSDHL.WEKHM.......
lcl|Glyma.07G235500_86-132     AAVCRSLRERCVSDHL.WERHM.......
lcl|Glyma.14G036300_85-130     AGVCRSLRESCVSDHL.WERH........
lcl|Glyma.02G278300_85-130     AGVCRSLRESCVSDHL.WERH........
lcl|Glyma.19G129600_88-132     ATVCTSLRDRCRSDHL.WKKH........
lcl|Glyma.03G126600_90-134     ATVCTSLRDRCRSDHL.WKKH........
lcl|Glyma.03G126700_21-66      AQVCTSLRDRIRSDAL.WEKKI.......
lcl|Glyma.03G126900_1-44       SEVCTCLRDRCRSDPL.WE..........
lcl|Glyma.03G126800_1-45       SEVCTFLRDRCRSDPL.WEL.........
lcl|Glyma.19G129700_11-53      PEVCTFFRDRCGSDPL.WE..........
lcl|Glyma.10G102300_11-56      SEVCVSLKDKCQSDHF.WEHHI.......
lcl|Glyma.19G077200_211-253    EGVCKSLCDAVRGDPLLWRTMHI......
lcl|Glyma.05G073500_216-258    EGVCRSLCDAVRGDPLLWRTMHI......
lcl|Glyma.08G101200_189-221    ERVCKSLHSTVRGDPLLWRSIH.......
lcl|Glyma.05G144800_201-233    ERVCKSLHSTVCGDPLLWRSIH.......
```

```
lcl|Glyma.08G043200_22-57      SQVCTSLRDAVNNDIL.............
lcl|Glyma.05G235800_20-55      SQVCTSLRDAVNNDIL.............
lcl|Glyma.18G253400_43-88      SSTCKTWRSLGSLPCL.WSSLDLR.....
lcl|Glyma.09G242200_43-88      SSTCKTWRSLGSSLCL.WSSLDLR.....
lcl|Glyma.08G221300_34-71      SMTCKRFRALSSSHTL.WRSLCK......
lcl|Glyma.07G020700_29-66      SITCKRFRALTSSHTL.WKSLCK......
lcl|Glyma.06G132400_2-45       QQVCRKWKLVASENAL.WSDLF.......
lcl|Glyma.04G232600_3-45       QQVCRKWKLVASDNTL.WSNLF.......
lcl|Glyma.14G009200_85-113     FQVCSTWREVSRSDLL.WQRLTRR.....
lcl|Glyma.16G096700_44-84      CLVSKSWRSLVSDNAL.WAHF........
lcl|Glyma.04G112600_47-88      SVVCKALRSLVSDNRL.WIHF........
lcl|Glyma.14G179500_64-91      SLVCRSWYRA...................
lcl|Glyma.02G211800_64-91      SLVCRSWYRA...................
lcl|Glyma.06G095400_17-52      SLVCKSWYRA...................
lcl|Glyma.04G093500_13-48      SLVCKSWYRA...................
lcl|Glyma.19G206800_11-41      SLVCKSWYEI...................
lcl|Glyma.19G100200_4-36       SLVCKSWYRI...................
lcl|Glyma.16G050500_4-36       SLVCKSWYRI...................
lcl|Glyma.02G065300_5-35       SLVCKNWHRL...................
lcl|Glyma.12G128600_9-41       SLVSWSFYHL...................
lcl|Glyma.08G216300_8-40       SLVCKQFLSITN.................
lcl|Glyma.07G026200_6-40       SLVCKQFLSITN.................
lcl|Glyma.19G068600_7-36       SLVCKRWLRLH..................
lcl|Glyma.16G071200_7-36       SLVCKRWLRLH..................
lcl|Glyma.17G019800_12-42      SLVCRRWFRL...................
lcl|Glyma.07G254400_12-42      SLVCRRWFRL...................
lcl|Glyma.15G101100_11-42      SLVCTRWLRL...................
lcl|Glyma.13G211600_11-42      SLVCTRWLRL...................
lcl|Glyma.06G318100_54-84      SLVCKRWLNL...................
lcl|Glyma.04G120000_114-144    SLVCKRWLNL...................
lcl|Glyma.09G229800_64-94      SLVCKRWLNL...................
lcl|Glyma.11G227300_23-43      SQVCRRWYEL...................
lcl|Glyma.02G254300_17-37      SQVCRRWYEL...................
lcl|Glyma.15G004900_2-35       SLVCKRLYYL...................
lcl|Glyma.08G059500_18-48      SLVCKEWYNA...................
lcl|Glyma.07G189800_17-46      SLVCKEWFN....................
lcl|Glyma.14G136200_20-60      RLVCKRWLQLLNIENYFREFC........
lcl|Glyma.17G167800_40-72      SAVCRRWLRV...................
lcl|Glyma.05G097100_59-91      SAVCRRWLRV...................
lcl|Glyma.17G238700_64-96      SLVCRRWLQ....................
lcl|Glyma.11G052600_36-77      SLVCRRWLRVDGQRRHRLS..........
lcl|Glyma.01G189600_36-77      SLVCLRWRLVDGQRRHRLS..........
lcl|Glyma.06G042800_9-51       SLVCRRWLKVEGHTHHRLC..........
lcl|Glyma.04G041800_24-67      SLVCSRWLKIEGQTYQRLSL.........
lcl|Glyma.07G060100_13-46      GLTCRRWLHV...................
lcl|Glyma.17G211000_69-101     ACVSKRWLML...................
lcl|Glyma.14G116800_66-106     ACVSKRWLMLLSSIC..............
lcl|Glyma.06G068400_68-108     ASVSKRWLMLLSSIC..............
lcl|Glyma.04G066900_66-109     ASVSKRWLMLLSSICKNE...........
lcl|Glyma.13G166200_66-97      ACVSKRWL.....................
lcl|Glyma.04G147000_69-100     AYVSKRWL.....................
lcl|Glyma.17G026300_26-71      KLCCKEFLRAAEDNYVYRHA.........
lcl|Glyma.07G247800_23-68      KLCCKEFLHAAEDDYVYRHA.........
lcl|Glyma.02G238900_22-70      KLSCKDFLHASEANNVWKNVSL.......
lcl|Glyma.20G105000_9-46       ASTSSTFRSCARHI...............
lcl|Glyma.17G035100_46-127     ELESSMFPSTESEIAAIDLTN........
lcl|Glyma.13G246100_26-61      CTQGSKFRNCKQF................
lcl|Glyma.07G162700_19-49      LVVCKDWLANAVD................
lcl|Glyma.03G214300_17-57      ACVCTKFSSLVRDFCWKTKC.........
lcl|Glyma.13G353900_191-234    ERYIVQNARLLQDMTI.............
lcl|Glyma.07G111600_14-69      SVVSQCHQRQSRPISLAMDLDLAKK....
lcl|Glyma.01G094300_169-235    TMVATAYQTSHECSKETIIDILVTGLSGQ
```
