# Supplementary material for: Genome-wide analysis of genes encoding core components of the ubiquitin system in soybean (Glycine max) reveals a potential role for ubiquitination in host immunity against soybean cyst nematode
Source: BMC Plant Biol. 2018 Jul 18;18:149. doi: 10.1186/s12870-018-1365-7 (PMC6052599; doi:10.1186/s12870-018-1365-7)
Supplement: Supplementary file 23 — Table S8. PCR primers used in this study. (DOCX 18 kb) [file 12870_2018_1365_MOESM23_ESM.docx]

**Supplemental Table 8. PCR primers used in this study.**

| **Experiment** | **Primer name** | **Sequence** |
| --- | --- | --- |
| **Gene cloning** | GmUBA1-F | 5' CACCATGCTTCCTAGAAAGAGAGTGAGGG 3' |
|  | GmUBA1-R | 5' TTAACGGAAATAGATGGATA 3' |
|  | GmUBC2-F | 5' CACCATGTCGACTCCTGCTAGGAAG 3' |
|  | GmUBC2-R | 5' TTAGTCAGCTGTCCAACTTTGCTCC 3' |
|  | GmUBC8-F | 5' CACCATGGCTTCAAAGCGCATC 3' |
|  | GmUBC8-R | 5' TCAGCCCATGGCATACTTC 3' |
|  | GmUBC19-F | 5' CACCATGGCTGCCACTAATAACATTC 3' |
|  | GmUBC19-R | 5' TCAATTTGCAGATTTGTACAACTTCTCCACC 3' |
|  | GmUBC21-F | 5' CACCATGCAGGCATCGCGGG 3' |
|  | GmUBC21-R | 5' TCAGCCTTTCTTTGGCATAGCCGC 3' |
|  | Glyma.04G235700-F | 5' CACCATGGACTTGATGAGCGTTG 3' |
|  | Glyma.04G235700-R | 5' TCAACTACAGAGATTTGGCATG 3' |
|  | Glyma.17G094000-F | 5' CACCATGGTTTCCGATTCAATCGC 3' |
|  | Glyma.17G094000-R | 5' TCAGGACCTCTCGATCATGCT 3' |
|  | Glyma.15G001100-F | 5' CACCATGCAATTAGCGTCAAATGGTG 3' |
|  | Glyma.15G001100-R | 5' CTAGTAAAGGCCCAACATCCTTAG 3' |
|  | Glyma.10G024100-F | 5' CACCATGATTCCTTGGGGTGGCC 3' |
|  | Glyma.10G024100-R | 5' TCAATGACGGAAAGTCTTCACAAC 3' |
|  | GmPUB10-F | 5' CACCATGGCCGGCGAGGAGCT 3' |
|  | GmPUB10-R | 5' TCAAAGTTGCTGCAACTTATGAATAT 3' |
|  | GmPUB13-F | 5' CACCATGGAGGGTGAGAACGCGAG 3' |
|  | GmPUB13-R | 5' TTAGGAATCATCGGGGTTAGTAATCAAAGG 3' |
|  | GmPUB22-F | 5' CACCATGAACGAAATCGATGTTCCTTCG 3' |
|  | GmPUB22-R | 5' TCACACATAATTCGGGTATGAATTAAGC 3' |
|  | GmPUB38-F | 5' CACCATGGGTGGCAACGGCAAG 3' |
|  | GmPUB38-R | 5' CTAAAACGTGGTCGTGTTGACGAG 3' |
| **qRT-PCR** | Glyma.05G048800-QF | 5' TCGTTGGTATTGCTGTTTGC 3' |
|  | Glyma.05G048800-QR | 5' ATCAGCATCGCATTCAATCA 3' |
|  | Glyma.17G098000-QF | 5' TCCTGCTAGGAAGAGGCTCA 3' |
|  | Glyma.17G098000-QR | 5' AGTTGGTGGCTTGTTTGGAT 3' |
|  | Glyma.01G131800-QF | 5' ACCTCACAACCACCCTCTTG 3' |
|  | Glyma.01G131800-QR | 5' ACTTGCTTCCGTTGGAGAAA 3' |
|  | Glyma.20G220100-QF | 5' GCTTCCAGATGCCAGGTTTT 3' |
|  | Glyma.20G220100-QR | 5' GCCGTAGAATCCAGAAGGGTT 3' |
|  | Glyma.10G150400-QF | 5' GTGAAGCAGAGTGGGAGGGTAT 3' |
|  | Glyma.10G150400-QR | 5' TTCACCACAGAAGAACTCACCC 3' |
|  | Glyma.15G101600-QF | 5' ACAACCGCTGAGTTTCACCT 3' |
|  | Glyma.15G101600-QR | 5' TTTTGGAGACCCTGATGGAG 3' |
|  | Glyma.13G326500-QF | 5' CTGCTGCTCAAGAAGGTTCC 3' |
|  | Glyma.13G326500-QR | 5' TCCCCTGAGTCTCTCTTCCA 3' |
|  | Glyma.08G046500-QF | 5' AACTTCATGGGCTCCTCCTT 3' |
|  | Glyma.08G046500-QR | 5' ACTGGCTAAACCCGTGAATG 3' |
|  | Glyma.10G179500-QF | 5' CGGGTGGAATGAATGTCTCT 3' |
|  | Glyma.10G179500-QR | 5' TCATGCGATCAACAAGTGGT 3' |
|  | Glyma.11G107800-QF | 5' GCAGGCCCTGGTTAATTATACTT 3' |
|  | Glyma.11G107800-QR | 5' GTGGGCACAGTATCTATTCCTCAT 3' |
|  | GmEF1A-QF | 5' TTTCGAGGGTGACAACATGA 3' |
|  | GmEF1A-QR | 5' GCTTCACGACACCAGTCTCA 3' |
