# Supplementary material for: Genome-wide analysis of genes encoding core components of the ubiquitin system in soybean (Glycine max) reveals a potential role for ubiquitination in host immunity against soybean cyst nematode
Source: BMC Plant Biol. 2018 Jul 18;18:149. doi: 10.1186/s12870-018-1365-7 (PMC6052599; doi:10.1186/s12870-018-1365-7)

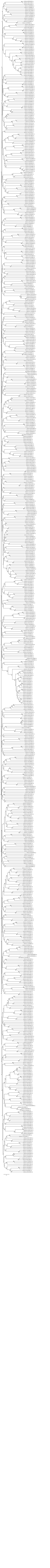

Supplement: Supplementary file 10 — Figure S7. Phylogenetic analysis of soybean RING domain-containing proteins. (PDF 187 kb) [file 12870_2018_1365_MOESM10_ESM.pdf]
